# Excess Copper-Induced Alterations of Protein Profiles and Related Physiological Parameters in *Citrus* Leaves

**DOI:** 10.3390/plants9030291

**Published:** 2020-02-28

**Authors:** Wei-Lin Huang, Feng-Lin Wu, Hui-Yu Huang, Wei-Tao Huang, Chong-Ling Deng, Lin-Tong Yang, Zeng-Rong Huang, Li-Song Chen

**Affiliations:** 1 Institute of Plant Nutritional Physiology and Molecular Biology, College of Resources and Environment, Fujian Agriculture and Forestry University, Fuzhou 350002, China; 1170807014@fafu.edu.cn (W.-L.H.); 1180807019@fafu.edu.cn (F.-L.W.); 1180807011@fafu.edu.cn (H.-Y.H.); 1190807012@fafu.edu.cn (W.-T.H.); talstoy@fafu.edu.cn (L.-T.Y.); 2 Guangxi Key Laboratory of Citrus Biology, Guangxi Academy of Specialty Crops, Guilin 541004, China; cldeng88168@126.com (C.-L.D.); huangzengrong@fafu.edu.cn (Z.-R.H.); 3 Fujian Provincial Key Laboratory of Soil Environmental Health and Regulation, College of Resources and Environment, Fujian Agriculture and Forestry University, Fuzhou 350002, China; 4 The Higher Education Key Laboratory of Fujian Province for Soil Ecosystem Health and Regulation, College of Resources and Environment, Fujian Agriculture and Forestry University, Fuzhou 350002, China

**Keywords:** *Citrus grandis*, *Citrus sinensis*, CO_2_ assimilation, copper-toxicity, 2-DE, leaves

## Abstract

This present study examined excess copper (Cu) effects on seedling growth, leaf Cu concentration, gas exchange, and protein profiles identified by a two-dimensional electrophoresis (2-DE) based mass spectrometry (MS) approach after *Citrus sinensis* and *Citrus grandis* seedlings were treated for six months with 0.5 (control), 200, 300, or 400 μM CuCl_2_. Forty-one and 37 differentially abundant protein (DAP) spots were identified in Cu-treated *C. grandis* and *C. sinensis* leaves, respectively, including some novel DAPs that were not reported in leaves and/or roots. Most of these DAPs were identified only in *C.* *grandis* or *C. sinensis* leaves. More DAPs increased in abundances than DAPs decreased in abundances were observed in Cu-treated *C. grandis* leaves, but the opposite was true in Cu-treated *C. sinensis* leaves. Over 50% of DAPs were associated with photosynthesis, carbohydrate, and energy metabolism. Cu-toxicity-induced reduction in leaf CO_2_ assimilation might be caused by decreased abundances of proteins related to photosynthetic electron transport chain (PETC) and CO_2_ assimilation. Cu-effects on PETC were more pronounced in *C. sinensis* leaves than in *C. grandis* leaves. DAPs related to antioxidation and detoxification, protein folding and assembly (viz., chaperones and folding catalysts), and signal transduction might be involved in *Citrus* Cu-toxicity and Cu-tolerance.

## 1. Introduction

Microelement copper (Cu) is highly toxic to plants when in excess. Cu-containing fungicides and bactericides are widely used in agriculture to control fungal and bacterial diseases in crops including *Citrus* in order to improve crop production and quality. Cu contamination in agriculture soils is on the rise all over the world [1,2]. Cu accumulation in soils can cause Cu-toxicity and related nutritional disorders, resulting in a series of adverse effects on plants ranging from morphological and physiological to molecular levels [1,3]. In old *Citrus* orchards, the excess accumulation of Cu in soils is a common phenomenon because of the extensive and continued use of Cu-containing agricultural chemicals against fruit and foliar diseases such as anthracnose and canker [3,4]. Cu concentration and availability in soils under continuous *Citrus* production orchards increase with increasing production period [2]. In *Citrus*, the common Cu-toxic symptoms include leaf iron (Fe) chlorosis, poor growth, and stunted, and discolored root systems [3,5].

Cu, which can act as a cofactor for over 100 proteins including plastocyanin, laccase, cytochrome *c* oxidase, Cu/zinc (Zn) superoxide dismutase (SOD), ethylene receptors, amino oxidase, polyphenol oxidases, ascorbate (ASC) oxidase, diamine oxidases, and phytocyanin, is involved in photosynthesis, respiration, ATP biosynthesis, ethylene reception, reactive oxygen species (ROS) metabolism, cell wall formation, and carbon, lipid, and nitrogen metabolisms [6]. Accordingly, a lot of researchers have examined the toxic effects of Cu on the uptake of nutrients and water [1,5], growth [1,3], photosynthetic pigment production [7], photosynthetic electron transport [5,8], CO_2_ assimilation [8], carbohydrate and nitrogen (N) metabolism [7,9], respiration [10], hormonal status [11], cell wall metabolism [12], phenolic metabolism [13], as well as ROS generation and detoxification [8].

Although Cu-toxic effects on plant growth and physiology have been investigated in some details [2,14], little is known about Cu-toxicity-induced alteration of protein profiles in plants. Proteomics is a powerful approach to elucidate the complicated responses of plants to unfavorable environments [15,16]. Recently, there have been several reports investigating Cu toxicity responsive proteins. Most reports, however, have focused on herbaceous plants, including rice [17,18,19], *Allium cepa* [20], *Oenothera glazioviana* [21], *Arabidopsis* [22], *Cannabis sativa* [23], *Agrostis capillaris* [24], *Elsholtzia splendens* [25,26], sorghum [27,28] and wheat [29], while only one study investigated Cu-toxic effects on protein profiles in leaves of woody plant *Eucalyptus camaldulensis* [30]. Also, most of the above studies mainly focused on Cu-toxicity-responsive proteins occurring in roots because Cu is preferentially accumulated in Cu-stressed roots, while only few studies investigated Cu-toxic effects on protein profiles in leaves [25,27,29,30]. Evidence shows that the toxic effects of Cu on plant proteomics vary with Cu concentration, plant species, populations and/or cultivars, and plant tissues [17,18,24,25,27,28,29]. Therefore, more extensive proteomic research on the leaves of woody plants is needed to elucidate the molecular mechanisms of plants under Cu-toxicity.

Here, a two-dimensional electrophoresis (2-DE) based mass spectrometry (MS) approach was used to examine Cu-toxicity-responsive proteins in *Citrus grandis* and *Citrus sinensis* leaves. Meanwhile, we examined excess Cu effects on seedling growth, and leaf Cu concentration and gas exchange. The objectives were (a) to identify Cu toxicity responsive proteins in *Citrus* leaves and (b) to screen the candidate proteins possibly responsible for Cu tolerance in *Citrus*.

## 2. Results

### 2.1. Excess Cu-Effects on Seedling Growth, Leaf Cu and Gas Exchange

As shown in Supplementary Figures S1 and S2, *C. sinensis* (*C. grandis*) biomass remained little changed as Cu concentration in the nutrient solution elevated from 0.5 to 300 (200) μM, then declined at 400 (300–400) μM Cu. Biomass was lower in *C. sinensis* seedlings than that in *C. grandis* seedlings at each given Cu supply.

**Figure 1:**
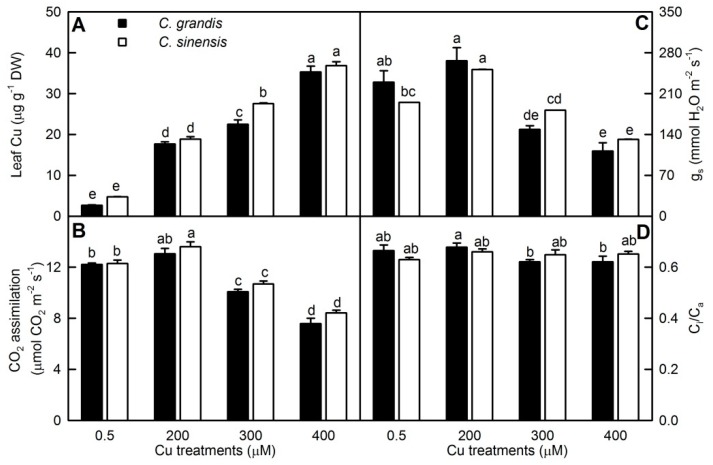
Cu-effects on Cu concentration (**A**), CO_2_ assimilation (**B**), stomatal conductance (g_s_, **C**) and ratio of intercellular to ambient CO_2_ concentration (C_i_/C_a_, **D**) in *Citrus grandis* and *Citrus sinensis* leaves. Bars represent means ± SE (*n* = 8 except for 4 for leaf Cu). Different letters above the bars indicate significant differences at *p* < 0.05.

Leaf Cu concentration increased with Cu supply and did not differ between the two *Citrus* species with the exception that its concentration in leaves was higher in *C. sinensis* than that in *C. grandis* at 300 μM (Figure 1A).

Leaf CO_2_ concentration and stomatal conductance (g_s_) kept unchanged or increased as Cu concentration in the nutrient solution rose from 0.5 to 200 μM, then declined with further rise in Cu concentration. Cu supply had little influence on the ratio of intercellular to ambient CO_2_ concentration (C_i_/C_a_) except for that C_i_/C_a_ in *C. grandis* leaves was slightly higher at 200 μM Cu than that at 300–400 μM Cu. No significant differences were observed in the three parameters between the two *Citrus* species over the range of Cu supply (Figure 1B–D).

Based on these results, seedlings that received 300–400 μM Cu were regarded as Cu excess.

### 2.2. Protein Yield and Cu-responsive Proteins in Leaves

**Figure 2:**
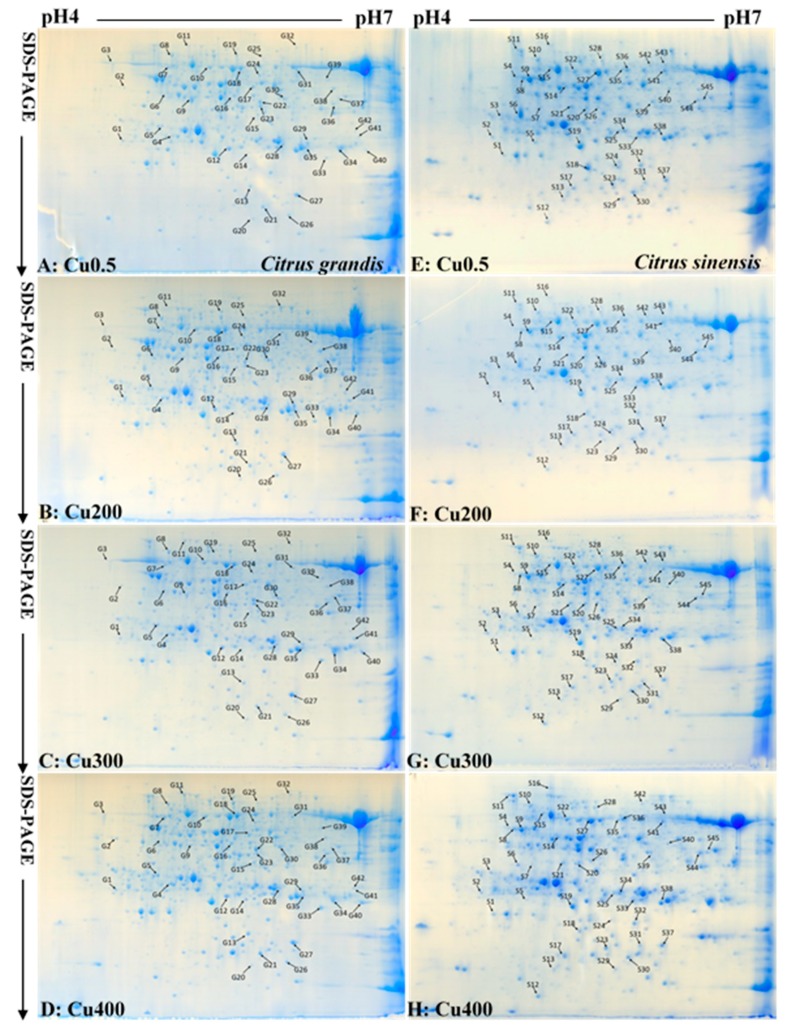
Representative 2-DE images of proteins extracted from 0.5 (**A**,**E**), 200 (**B**,**F**) 300 (**C**,**G**) and 400 (**D**,**H**) Cu-treated *Citrus grandis* (**A**–**D**) and *Citrus sinensis* (**E**–**H**) leaves.

Three biological replicates were performed in order to obtain reliable data. No significant differences were observed in protein yields and the number of protein spots per gel among eight means (Table 1, Figure 2, Supplementary Figures S3 and S4).

As shown in Table 1 and Table 2, Supplementary Table S1 and Figure S5, a total of 42 and 45 differentially abundant protein (DAP) spots were obtained from Cu-treated *C. grandis* and *C. sinensis* leaves, respectively. All of these DAP spots were submitted to matrix-assisted laser desorption/ionization tandem time-of-flight mass spectrometry (MALDI-TOF/TOF-MS) based identification. In total, 41 and 37 DAP spots were identified in 200, 300, and/or 400 μM Cu-treated *C. sinensis* and *C. grandis* leaves, responsively. Most of these DAP spots only presented in Cu-treated *C. sinensis* or *C. grandis* leaves. Only seven DAPs with the same accession number [viz., Orange1.1t05091.1, S-adenosyl-L-homocysteine (AdoHcy) hydrolase (Orange1.1t01892.1), chaperonin CPN60-1, (Orange1.1t01459.2), major allergen Pru ar 1 (Cs9g03630.1), ribulose bisphosphate carboxylase/oxygenase (Rubisco) activase 1 (Cs7g31800.3), sedoheptulose-1,7-bisphosphatase (Cs7g31640.4), and 29 kDa ribonucleoprotein A (CP29A; Cs6g11900.1)] presented in the two *Citrus* species. Fifteen, 12 and 12 (2, 4, and 5) spots increased in abundances and 4, 2, and 9 (9, 12, and 23) spots decreased (including disappeared) in abundances were identified in 200, 300, or 400 μM Cu-treated *C. grandis* (*C. sinensis*) leaves, responsively. Obviously, more (less) DAPs increased in abundances than DAPs decreased in abundances were obtained in 200, 300, or 400 μM Cu-treated *C. grandis* (*C. sinensis*) leaves. For *C. grandis*, 10, 6, or 14 DAP spots were identified only in 200, 300 or 400 μM Cu-treated leaves, respectively, only 2 DAP spots with the same accession number (viz., malate dehydrogenase (MDH, Cs9g10470.1) and glutathione S-transferase (GST, Cs5g32800.1)) were shared by the three. For *C. sinensis*, 3, 3, or 15 DAP spots were identified only in 200, 300, or 400 μM Cu-treated leaves, respectively, only 2 DAP spots with the same accession number (viz., ferritin-3 (Cs6g09150.2) and enolase (Cs6g15540.1)) were shared by the three.

**Figure 3:**
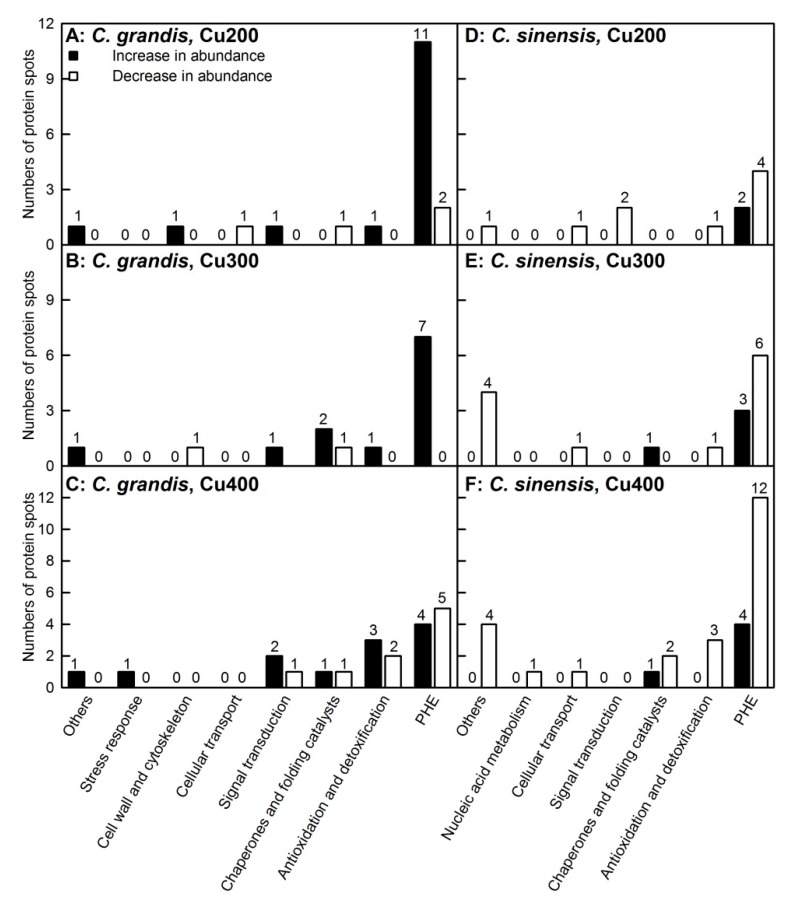
Differentially abundant proteins (DAPs) in 200, 300 and 400 μM Cu-treated *Citrus grandis* (**A**–**C**) and *Citrus sinensis* (**D**–**F**) leaves. PHE: photosynthesis, carbohydrate and energy metabolism.

DAPs were mainly involved in photosynthesis, carbohydrate and energy metabolism, antioxidation and detoxification, protein folding and assembly (viz., chaperones and folding catalysts), and others. Cell wall, cytoskeleton (G7 and G2), and stress response (G14) related DAPs were obtained only in Cu-treated *C. grandis* leaves, but nucleic acid metabolism related DAP (S42) was identified only in Cu-treated *C. sinensis* leaves (Table 2 and Figure 3).

### 2.3. KEGG Pathway Analysis of DAPs

For total DAPs in *C. grandis* leaves, there were eight significantly enriched KEGG pathways-namely carbon fixation in photosynthetic organisms (ko00710), exosome (ko04147), glycolysis/gluconeogenesis (ko00010), fructose and mannose metabolism (ko00051), photosynthesis (ko00195), chaperones and folding catalysts (ko03110), photosynthesis proteins (ko00194) and inositol phosphate metabolism (ko00562). Four, six, and ten KEGG pathways were significantly enriched by DAPs in 200, 300, and 400 μM Cu-treated *C. grandis* leaves, respectively. For total DAPs in *C. sinensis* leaves, carbon fixation in photosynthetic organisms, photosynthesis proteins, exosome, tricarboxylic acid (TCA) cycle (ko00020) and photosynthesis were the significantly enriched KEGG pathways. One [photosynthesis-antenna proteins (ko00196)], one (exosome) and five KEGG pathways were significantly enriched by DAPs in 200, 300 and 400 μM Cu-treated *C. sinensis* leaves, respectively (Supplementary Figure S6).

### 2.4. PCA Loading Plots and Correlation Matrices of DAPs

**Figure 4:**
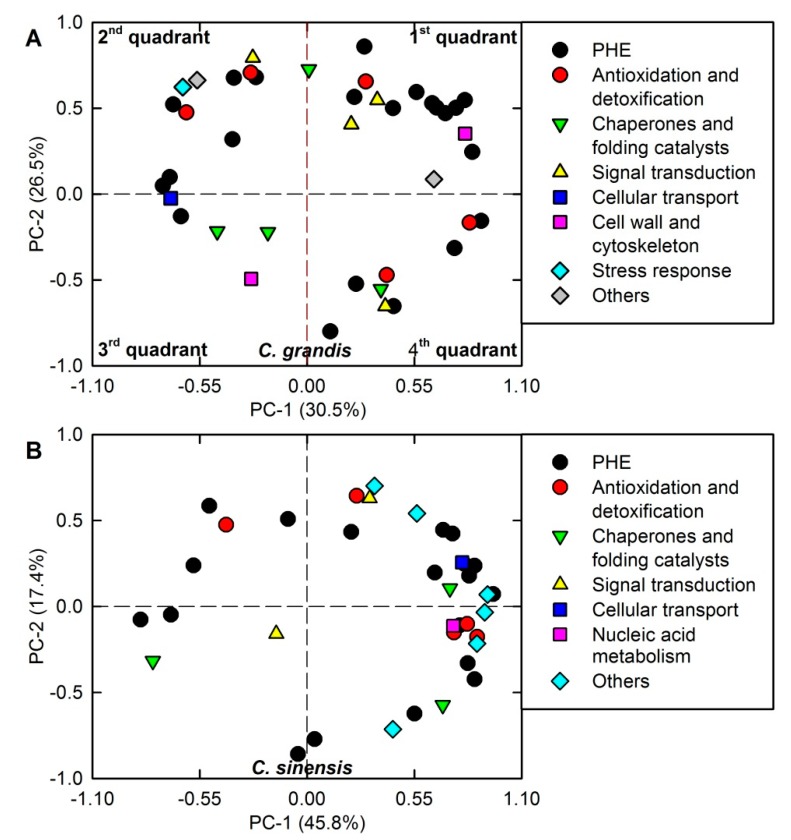
Principal component analysis (PCA) of differentially abundant proteins (DAPs) in Cu-treated *Citrus grandis* (**A**) and *Citrus sinensis* (**B**) leaves. PHE: photosynthesis, carbohydrate and energy metabolism.

As shown in Figure 4 and Supplementary Tables S2 and S3, PC1 and PC2 accounted for 30.5% and 26.5%, and 45.8% and 17.4% of the total variation in *C. grandis* and *C. sinensis* leaves, respectively. The association patterns of DAPs were more obvious in *C. sinensis* leaves than those in *C. grandis* leaves. Similarly, more positive and negative relationships between DAP spots existed in *C. sinensis* leaves than those in *C. grandis* leaves (Supplementary Figure S7).

### 2.5. qRT-PCR Analysis of Genes for DAPs

The expression levels of genes for 22 DAPs from 400 μM Cu-treated *C. grandis* (viz., G3, G9, G10, G11, G14, G26, G29, G33, G34, and G35) and *C. sinensis* (viz., S2, S5, S9, S16, S17, S23, S24, S30, S32, S33, S37, and S43) leaves were analyzed by qRT-PCR. With the exceptions of G26, G33, S9, S16, S23, S32, and S33, the abundances of the other 16 DAPs matched well with the expression levels of the corresponding genes regardless of whether *PRPF31* or *actin* served as an internal standard (Table 2 and Supplementary Figure S8).

## 3. Discussion

### 3.1. DAPs Related to Photosynthesis, Carbohydrate and Energy Metabolism

Excess Cu-treated *C. grandis* and *C. sinensis* leaves had lower CO_2_ assimilation (Figure 1) and higher concentrations of nonstructural carbohydrates relative to controls [5]. Accordingly, many Cu-toxicity-responsive proteins related to photosynthesis, carbohydrate and energy were identified in these leaves (Table 2 and Figure 3). Damkjær et al. reported that *Arabidopsis* mutants lacking light-harvesting chlorophyll (Chl) a/b binding protein *Lhcb3* had a lower maximum photosystem (PSII) efficiency of dark-adapted leaves (F_v_/F_m_) than wild type under high light condition and still displayed a lower F_v_/F_m_ after 7 d of recovery under normal light, implying that PSII in these plants suffered from photoinhibition under high light [31]. The abundance of Chl a-b binding protein 8 (Lhca3; S19) was increased and decreased in 200 and 400 μM Cu-treated *C. sinensis* leaves, respectively. Thus, the decreased abundance of Lhca3 in 400 μM Cu-treated *C. sinensis* leaves might contribute to the Cu-induced photoinhibition. This could explain why photoinhibition was slightly greater in 400 μM Cu-treated *C. sinensis* leaves than that in 400 μM Cu-treated *C. grandis* leaves [5]. Also, the abundance of protease Do-like 1 (DEGP1; S41) was decreased in 200 and 400 μM Cu-treated *C. sinensis* leaves. DEGP1, an enzyme responsible for the degradation of damaged proteins, plays a role in photoinhibition repair of PSII in *Arabidopsis* [32]. Also, the abundance of PsbP domain-containing protein 3 (PPD3, S13) involved in PSII light reaction was decreased in 300 and 400 μM Cu-treated *C. sinensis* leaves.

Phosphorylation of PSII antenna protein RNA-binding protein CP29, localized in chloroplasts, was induced under conditions of decreased photosynthetic capacity and excess light. Maize plants lacking the ability to perform the phosphorylation of CP29 were more sensitive to cold-induced photoinhibition [33]. CP29 phosphorylation has been indicated to play a role in lowering ^1^O_2_ generation and improving excess energy dissipation [34]. The abundance of CP29A (G1) was increased in 200 and 400 μM Cu-treated *C. grandis* leaves, while the abundances of CP29A (S2 and S32) were decreased in 300 and 400 μM Cu-treated *C. sinensis* leaves. The different response of CP20A to excess Cu between the two agreed with the report that excess Cu had less influence on Chl a fluorescence (OJIP) transients in *C. grandis* leaves than those in *C. sinensis* leaves [5]. Similarly, the abundance of PSII stability/assembly factor HCF136, an essential protein for the stability/assembly of PSII, was increased in 300 (G31) and 400 (G30) μM Cu-treated *C. grandis* leaves, but not in Cu-treated *C. sinensis* leaves. Increased abundance of HCF136 has been obtained in cadmium (Cd) treated *Arabidopsis* shoots [35]. However, the abundances of oxygen-evolving enhancer protein 1 (PSBO2, S17) were enhanced significantly in 400 μM Cu-treated *C. sinensis* leaves. PSBO2 is required for the stability of the photosynthetic water-splitting complex [36]. Interestingly, the damage of the oxygen evolving complexes (OEC) was greater in *C. sinensis* leaves than that in *C. grandis* leaves under 400 μM Cu [5]. Evidently, other factors play a role in stabilizing the water-splitting complex.

The abundance of G10 (ferredoxin-NADP reductase, leaf-type isozyme (LFNR2)) was decreased in 200 and 400 μM Cu-treated *C. grandis* leaves, and of G42 (LFNR2) was increased in 200 μM Cu-treated *C. grandis* leaves. LFNR oxidizes ferredoxin (Fd) to yield NADPH, which is utilized in various reactions such as lipid and Chl biosynthesis, CO_2_ fixation and stromal redox regulation. *Arabidopsis fnr2* RNAi mutants had decreased concentrations of photosynthetic thylakoid proteins and Chls, and rate of carbon fixation relative to the wild type plants [37]. The abundances of Rubisco subunit binding-protein α subunit [chaperonin 60 subunit α 1 (Cpn60α1); S11] involved in protein folding and Rubisco activase 1 (G4, S4, and S14) involved in the activation of Rubisco were decreased in 400 μM Cu-treated *C. sinensis* and/or *C. grandis* leaves. The abundance of Rubisco activase 1 (S14) was also decreased in 200 and 300 μM Cu-treated *C. sinensis* leaves. Cpn60α1 is necessary for the folding of Rubisco large subunit (rbcL) and proper chloroplast development [38]. Rubisco activase-deficient transgenic tobacco plants had decreased Rubisco carbamylation and CO_2_ assimilation [39]. Also, the abundances of sedoheptulose-1,7-bisphosphatas (SBPase; S33) and phosphoribulokinase (PRK; S21) involved in Calvin cycle were decreased in 400 μM Cu-treated *C. granids* leaves. The lower abundances of LFNR2 (G10), Cpn60α1 (S11), Rubisco activase 1 (G4, S4, S9 and S14), PRK (S21) and SBPase (S33) agreed with our finding and previous report that excess Cu-treated *Citrus* leaves had reduced CO_2_ assimilation and Chl concentrations (Figure 1) [5]. However, the abundances of LFNR2 (G42), SBPase (G6) and glyceraldehyde-3-phosphate dehydrogenase B (GAPB, G38) were increased in 200 μM Cu-treated *C. grandis* leaves. This was agreement with the finding that CO_2_ assimilation displayed an upward trend in 200 μM Cu-treated *C. grandis* leaves relative to controls (Figure 1). Similarly, the abundance of fructose-1,6-bisphosphatase, cytosolic (cyFBPase; G36), a major site for controlling sucrose synthesis, was increased in 200 μM Cu-treated *C. grandis*. Strand et al. reported that photosynthesis was inhibited in antisense *cyFBPase Arabidopsis* mutants [40]. Also, the abundance of Cpn60α1 (G8) in *C. grandis* leaves was decreased and increased at 200 and 300 μM Cu, respectively, and the abundance of Rubisco activase 1 (S10) was increased in 300 μM Cu-treated *C. sinensis* leaves.

Carbonic anhydrase (CA, a Zn-metalloenzyme) is required for CO_2_ assimilation in cotyledons. The abundance of CA (S3) was increased or unaffected by Cu supply in *C. sinensis* leaves. However, CA activity was reduced in Cu excess *Brassica juncea* [41]. The difference between CA abundance and activity could be explained by the Cu-induced decrease in Zn level in *C. sinensis* [5], because its activity is regulated by Zn availability.

Mitochondrial MDH (mMDH) is necessary for CO_2_ and energy partitioning in leaves. Antisense *mMDH* tomato plants displayed increased photosynthetic electron transport rate, CO_2_ assimilation, g_s_ and growth rate, but decreased respiration rate [42]. The increased abundances of mMDH (S44 and S45) in 400 μM Cu-treated *C. sinensis* leaves agreed with the report that 400 μM Cu-treated *C. sinensis* seedlings had decreased growth, leaf CO_2_ assimilation and g_s_, and impaired photosynthetic electron transport chain (PETC) [5]. Chloroplastic NADP-MDH, which catalyzes the excess NADPH produced through PETC and oxaloacetate to malate and NADP^+^, plays a key role in counteracting PETC over-reduction and in H_2_O_2_ signaling by exporting chloroplast NADPH to other cell compartments. *Arabidopsis nadp-mdh* mutants lacked the reversible inactivation of catalase activity and the concomitant accumulation of H_2_O_2_, but had a higher reduction state of the plastoquinone (PQ) pool when exposed to high light [43]. The decreased abundance of NADP-MDH (G17) in 400 μM Cu-treated *C. grandis* leaves might contribute to the Cu-induced inhibition of photosynthesis and the increased reduction of the PSII acceptor side, as indicated by the positive ΔJ- and ΔI-bands in 400 μM Cu-treated *C. grandis* leaves [5]. However, Cu treatments increased or did not alter the abundance of cytosolic MDH (cyMDH; G37 and G39) in *C. grandis* leaves. cyMDH plays a key role in the transport of chloroplast or mitochondria NADPH to other cell compartments. Transgenic apple plants overexpressing an apple cyMDH gene displayed a higher stress-tolerance accompanied by increased reducing power, as indicated by increased concentrations of ASC and reduced glutathione (GSH) and ratios of ASC/dehydroascorbate (DHA), GSH/GSSG and NAD(P)H/NAD(P)^+^ [44]. Thus, the Cu-toxicity-induced increases of cyMDH abundances in *C. grandis* leaves might be an adaptive strategy.

Pentose phosphate pathway (PPP) provides NADPH for biosynthesis of GSH and maintenance of cellular redox state necessary to deal with oxidative stress. *Arabidopsis PGL3* T-DNA insertion mutants with decreased flux through the plastidial PPP displayed a decrease in plant size and a lower cellular redox potential [45]. The increased abundance of probable 6-phosphogluconolactonase 4 (PGL4, an enzyme involved in PPP; G4) in 200 and 300 μM Cu-treated *C. grandis* leaves agreed with the increased needs for ROS scavenging [5].

Triosephosphate isomerase (TPI), which catalyzing the reversible interconversion of glyceraldehydes 3-phosphate (GAP) and dihydroxyacetone phosphate (DHAP), may prevent the spontaneous degradation of DHAP into methylglyoxal (MG, a cytotoxic metabolite). TPI-deficiency led to increased generation of MG in red blood cells [46]. The decreased abundances of TPI (G29) in 400 μM Cu-treated *C. grandis* leaves implied that MG formation was increased in these leaves, thus increasing ROS generation and lipid peroxidation [5].

The increased abundances of glucose-1-phosphate adenylyltransferase (APS, G24, and G25) in 200 and 300 μM Cu-treated *C. grandis* leaves implied that starch biosynthesis was enhanced in these leaves. However, this way could not explain starch accumulation in 400 μM Cu-treated *C. grandis* leaves, because APS abundance was not increased in these leaves. A weaker sink for the photosynthetic requirement due to Cu toxicity-induced inhibition of growth has been suggested to be responsible for the accumulation of nonstructural carbohydrates including starch in Cu-toxic *Citrus* leaves [5].

There is a close relation between energy availability and stress-tolerance [47]. An extra energy supply is necessary for stressed plants to fortify their tolerance. The increased abundances of ATP synthase subunit β (G12) and ATP synthase γ chain (G23) 300 μM Cu-treated *C. grandis* leaves and bis(5’-adenosyl)-triphosphatase (Ap3A, G26) in 400 μM Cu-treated *C. grandis* leaves suggested that ATP biosynthesis was enhanced in these leaves to meet the increased energy needs. Similar result has been obtained in Cu-stressed *Elsholtzia splendens* leaves [25].

To conclude, Cu-toxicity might affect the abundances of proteins involved in PETC and CO_2_ assimilation, thus decreasing electron transport rate and CO_2_ assimilation. Cu-toxic effects on PETC were more pronounced in *C. sinensis* leaves than those in *C. grandis* leaves.

### 3.2. DAPs Related to Antioxidation and Detoxification

Five (five) DAP spots involved in antioxidation and detoxification were identified in Cu-treated *C. sinensis* (*C. grandis*) leaves (Table 2). The striking Cu-mediated alteration was the big increase in GST (G40) abundance in Cu-treated *C. grandis* leaves. *Dianthus superbus* plants overexpressing *GST* were observed to biosynthesize phytochelatins (PCs), thus sequestering and detoxifying excess Cu [48]. Lambda class of GSTs could be used to enhance plant tolerance against various stresses including heavy metals [49]. However, the abundance of GST DHAR1 (G34), an enzyme having glutathione-dependent thiol transferase and DHA reductase (DHAR) activities, was decreased in 400 μM Cu-treated *C. grandis* leaves. SOD can rapidly dismutate O_2_^−^ to H_2_O_2_ and protect organisms against oxidative damage. The increased abundances of Cu/Zn SOD (G21) and manganese (Mn) SOD (G33) in 400 μM Cu-treated *C. grandis* leaves agreed with the report that Cu stress increased Cu/Zn SOD and Mn SOD activity in *Arabidopsis* leaves [50]. Cu/Zn SOD abundance increased and Fe SOD abundance decreased in Cu-sufficient *Arabidopsis* leaves, but the reverse was true in Cu-limited leaves, which could save Cu for the biosynthesis of plastocyanin necessary for photosynthesis [51]. Thus, excess Cu increased the biosynthesis of Cu/Zn SOD by a direct effect of Cu on the gene for SOD, hence preventing a Cu-toxic effect on photosynthesis. Methyl viologen (mainly to enhance PSI-originated ROS formation) induced decrease of F_v_/F_m_ was more severe in *aor* [a chloroplastic NADPH-dependent alkenal/one oxidoreductase (AOR, At1g23740)] *Arabidopsis* mutants than in Col-0 plants, concluding that AOR played a role in the scavenging of stromal reactive carbonyls (RCs) generated under oxidative stress [52]. Therefore, the decreased abundance of quinone oxidoreductase-like protein At1g23740 (G16) in 400 μM Cu-treated *C. grandis* might contribute to the Cu-induced inhibition of photosynthesis by lowering the photosynthetic electron transport rate.

The abundances of all the five DAP spots were decreased in Cu-treated *C. sinensis* leaves. The decreased abundances of three H_2_O_2_ detoxifying enzymes in 300 (S1) and 400 (S24 and S34) μM Cu-treated *C. sinensis* leaves agreed with the report that H_2_O_2_ production was increased in these leaves [5]. Cysteine (Cys) synthase (CS) catalyzes the final step for Cys biosynthesis in plants. The overexpression of *CS* conferred tolerance to Cd and selenium (Se) by over-production of Cys, GSH and presumably PCs, but not to Cu in transgenic tobacco plants [53]. PCs have been proven not to be the major factor responsible for plant Cu-tolerance [54]. Thus, the Cu-induced decrease of CS abundance (S20 and S39) might not lower the tolerance of *C. sinensis* seedlings to Cu.

To conclude, the antioxidation and detoxification system as a whole could not effectively protect *Citrus* leaves from Cu-toxicity-induced oxidative stress, as indicated by the increased H_2_O_2_ production and electrolyte leakage [5].

### 3.3. Chaperones and Folding Catalysts

Luminal binding protein (BiP) functions in both protein folding and endoplasmic reticulum (ER) quality control mechanism. Heterologous expression of an ER BiP gene alleviated Cd-induced ER stress and programmed cell death in transgenic tobacco BY-2 cells [55]. Transgenic tobacco plants overexpressing an ER chaperone BiP gene had enhanced Cd-tolerance accompanied by decreased level of ROS and increased level of GSH [56]. Thus, the increased abundance of luminal-binding protein 5 (BiP5, S16) might contribute to Cu-tolerance of *C. sinensis*. Protein disulfide isomerase (PDI), which catalyzes thiol-disulfide interchange, is the most abundant oxidative protein folding catalyst and a multifunctional protein chaperone. PDI could serve as a Cu chelator or Cu delivering protein to protect cells against Cu-toxicity [57]. The increased abundance of probable PDI A6 (G15) in 400 μM Cu-treated *C. grandis* leaves might play a role in preventing these leaves from Cu-toxicity by binding Cu and/or decreasing oxidative damage. Like Cpn60α1 (G8), the abundance of 20 kDa chaperonin (Cpn20, a co-chaperonin of CPN60; G12) was increased in 300 μM Cu-treated *C. grandis* leaves. Cpn20 played a role in oxidative stress protection and chloroplast development via positively regulating the activation of Fe SOD [58]. Interestingly, the abundance of chaperonin CPN60-1 (G19 and S26) involved in the correct folding of imported proteins was decreased and increased in 200 and 300 μM Cu-treated *C. grandis* leaves, respectively, but was decreased in 400 μM Cu-treated *C. sinensis* leaves. Also, the abundance of heat shock cognate 70 kDa protein 2 (HSP70-2, G11) involved in the folding of de novo translocation of precursor proteins into organelles, and degradation of damaged protein under disadvantaged conditions was decreased in 300 and 400 μM Cu-treated *C. grandis* leaves. These results demonstrate the involvement of chaperones and folding catalysts in the Cu tolerance and Cu toxicity of *Citrus*.

### 3.4. DAPs Related to Signal Transduction

Plant plasma membrane (PM) H^+^-ATPase activity can be regulated by 14-3-3 proteins involved in brassinosteroid (BR)-mediated signaling pathway [59]. The increased abundance of 14-3-3 protein 7 (G5) in 400 μM Cu-treated *C. grandis* leaves agreed with the increased expression of a 14-3-3 gene in *Fucus vesiculosus* in response to moderately elevated level of Cu [60], and the increased activity of PM H^+^-ATPase in Cu-treated cucumber roots [61]. However, the abundance of 14-3-3 protein 6 (S7) was reduced in 200 μM Cu-treated *C. sinensis* leaves.

Major pollen allergen, which is involved in abscisic acid (ABA)-activated signaling pathway, have high sequence homology to pathogenesis related (PR) proteins. The increased or unaltered abundance of major allergen Pru ar 1 (G27) in 200–400 μM *C. grandis* leaves agreed with the elevated abundances of Bet v 1-Sc3 (PR-10c) and PvPR1 in Cu-stressed *Betula pendula* and bean leaves, respectively [62,63]. Annexins, a key element of Ca^2+^-signaling pathways, are involved in counteracting oxidative stress. Transgenic tobacco plants overexpressing an annexin displayed elevated total peroxidase activity, improved tolerance/resistance to Cd, oxidative stress and diseases, and increased message levels for several PR proteins [64]. The increased or unchanged abundance of annexin D1 (G22) in Cu-treated *C. grandis* leaves agreed with the increased abundance of annexin D1 in Cu-stressed *Allium cepa* roots [20]. Thus, Cu supply might enhance the resistance of *C. grandis* to diseases [65]. However, the abundance of major allergen Pru ar 1 (S35) was decreased in 200 μM Cu-treated *C. sinensis* leaves.

Calreticulin (CRT), a crucial Ca^2+^-binding protein mainly in the ER, functions in Ca^2+^ signaling in response to stress in plants. The decreased abundance of CRT-1 (G3) agreed the decreased abundance of CRT in excess Cu-treated *Ectocarpus siliculosus* [66] and the decreased expression level of *CRT* in Mg-deficient *Citrus reticulata* leaves [67], because Mg concentration was decreased in Cu-stressed *C. grandis* leaves [5].

To conclude, hormone (ABA and BR)- and Ca^2+^-mediated signaling pathways might function in *Citrus* Cu-tolerance and Cu-toxicity. This was also supported by data suggesting that 28-homobrassinolide [41] and Ca [68] could alleviate plant Cu-toxicity, and that a reciprocal cross-talk existed between Cu status and ABA metabolism and signaling in *Arabidopsis* [69].

### 3.5. DAPs Related to Cellular Transport, Nucleic Acid and Cell Wall Metabolisms, and Cytoskeleton

Ferritin can protect plant cells from Fe-toxicity by storing excess Fe in a non-toxic form in plant cells [70]. A characteristic of Cu-toxicity in *Citrus* leaves is Fe chlorosis [5,71]. The decreased abundance of ferritin-3 (S5) in 200–400 μM Cu-treated *C. sinensis* leaves agreed with the report that ferritin accumulation in plant cells increased under high Fe concentrations [72]. The decreased abundance of ferritin-3 might contribute to Fe homeostasis by lowering the chelation of Fe to ferritin.

Both α- and β-tubulins are the primary constituents of microtubules (MTs), one of the cytoskeletal components. MTs have been proposed to function in plant Cu-toxicity and Cu-tolerance. Song et al. found that the abundances of three protein spots-namely tubulin α-1 chain, putative tubulin α-1 chain and tubulin α-2 chain, were decreased in excess Cu-treated rice roots, concluding that the decreased accumulation of α-tubulin might impair MT polymerization and alignment, thus influencing MT functions [18]. However, the abundance of tubulin β-6 chain (G7) in *C. grandis* leaves increased or did not alter in response to Cu supply, implying that MTs might be not impaired in these leaves. This might be related to the preferential accumulation of most Cu in the roots under Cu-stress [5].

DNA helicases, which are ATP-dependent DNA unwinding enzymes, are involved in DNA repair, replication and recombination. Ectopic expression of a *Medicago sativa* helicase 1 (a homolog of the pea DNA helicase 4) gene conferred *Arabidopsis* tolerance to drought, salt and oxidative stress [73]. The decreased abundance of RuvB-like helicase 1 (S42) in 400 μM Cu-treated *C. sinensis* leaves implied that DNA repair was impaired in these leaves.

The decreased or unaltered abundance of endochitinase 1 (G2) related to cell wall polysaccharide (macromolecule) catabolic process in Cu-treated *C. grandis* leaves implied that the level of cell wall polysaccharides might be increased in these leaves because of decreased degradation. This agreed with the increased concentration of total polysaccharide in the cell walls of Cu-treated *Elsholtzia splendens* roots [26]. However, the abundance of chitinase was enhanced in rice leaves treated with 100 μM Cu for 72 h [74]. Chitinase activity was not altered in pepper roots, stems, and cotyledons after 28 days of treatment with 50 μM Cu [65]. Thus, it seems that the effects of Cu on chitinase vary with plant species, Cu concentration, and time of exposure to Cu.

### 3.6. Other DAPs

AdoHcy hydrolase, which catalyzes the reversible hydrolysis of AdoHcy to L-homocysteine and adenosine, plays a crucial role in maintaining methyl cycling via the removal of AdoHcy. Taddei et al. observed that AdoHcy hydrolase was induced by Cu stress in in vitro-cultured pith explants of *Nicotiana glauca*, suggesting that AdoHcy hydrolase played a crucial role in regulating Cu level and intracellular distribution [75]. B-induced alleviation of *C. grandis* Al-toxicity was accompanied by increased root expression of *adenosylhomocysteinase-like* [76]. The increased abundance of AdoHcy hydrolase (G32) in 200 μM Cu-treated *C. grandis* leaves might contribute to their Cu-tolerance. However, its (S37) abundance was decreased in 400 μM Cu-treated *C. sinensis* leaves.

Flavonoids can act as ROS scavengers, and inhibit ROS production by chelating metals. The decreased abundance of dihydroflavonol-4-reductase (DFR; S38) in 400 μM Cu-treated *C. sinensis* leaves suggested that anthocyanin biosynthesis might be decreased in these leaves. This disagreed with the increased expression level of *DFR* in Cu-stressed rice leaves [77].

## 4. Materials and Methods

### 4.1. Plant Materials

Seedling culture and Cu treatments were made according to Li et al. [5]. Briefly, 6-week-old uniform seedlings of ‘Xuegan’ (*Citrus sinensis*) and ‘Shatian pummelo’ (*Citrus grandis*) were transported to 6 L pots (two plants per pot) filled with sand thoroughly washed with tap water, then grown in a greenhouse under natural conditions at Fujian Agriculture and Forestry University. Six weeks after transporting, seedlings were watered daily with freshly papered nutrient solution at a Cu concentration of 0.5 (Cu0.5, control), 200 (Cu200), 300 (Cu300), or 400 (Cu400) μM from CuCl_2_ until nutrients begin to flow out of the bottom hole of the pot (~500 mL per pot). Nutrient solution pH was adjusted to 4.8 with 1 M HCl before supply. Six months after Cu treatments, the fully expanded (about 7-week-old) leaves were used for all measurements. Firstly, leaf gas exchange was measured. Then, leaves (winged leaves, petioles and midribs removed) were taken at a sunny noon and immediately frozen in liquid N_2_. All samples were stored at −80 °C until extraction of proteins and total RNA. These seedlings unused for the collection of leaves were used for the measurements of plant dry weight (DW) and leaf Cu.

### 4.2. Measurements of Plant DW, and Leaf Gas Exchange and Cu Concentration

Root, stem, and leaf DW were weighted after being washed with tap water and dried to a constant weight at 70 °C (~48 h) [78].

Gas exchange was measured with a CIARS-2 portable photosynthesis system (PP systems, Herts, UK) at a controlled light intensity of ~1000 μmol m^−2^ s^−1^ and a controlled CO_2_ concentration of ~380 μmol mol^−1^ between 9:30 and 12:30 a.m. on a sunny day [79].

Leaf Cu was determined with a NexION 300X Inductively Coupled Plasma Mass Spectrometer (ICP-MS, PerkinElmer, Shelton, CT, USA).

### 4.3. Leaf Protein Extraction, 2-DE and Image Analysis

About 1 g of frozen leaves harvested equally from four seedlings (one seedling per pot) was mixed as one biological replicate. There were three biological replicates per treatment (a total of 12 seedlings from 12 pots). Proteins were extracted using a phenol extraction procedure [80] and their concentration was measured as described by Bradford [81]. 2-DE was performed according to Sang et al. [82]. Stained gels were scanned with an Epson Scanner (Seiko Epson Corporation, Japan) at a resolution of 300 dpi. Images were analyzed with PDQuest version 8.0.1 (BioRad, Hercules, CA, USA), including background subtraction, normalization, spot detection, matching, Gaussian fitting and gel alignment [83]. A fold change of >1.5 or <0.67 was set to determine DAP spots in addition to a *p*-value < 0.05. After being visually checked and manually excised from gels, all DAP spots were submitted to MALDI-TOF/TOF-MS-based identification.

### 4.4. MALDI-TOF/TOF-MS-Based Protein Identification and Bioinformatic Analysis

Peptide identification was carried out on an AB SCIEX 5800 TOF/TOF plus MS (AB SCIEX, Shanghai, China) as described by Peng et al. [83]. After being processed with TOF/TOF Explorer™ Software (AB SCIEX, Shanghai, China) in a default mode, all acquired spectra were submitted to MASCOT (Version 2.3, Matrix Science Inc., Boston, MA) by GPS Explorer (Version 3.6) for the search of *C. sinensis* databases (http://citrus.hzau.edu.cn/orange/index.php) using following search parameters: trypsin cleavage with one missed, MS tolerance of 100 ppm and MS/MS tolerance of 0.6 Da. At least two of matched peptides were necessary for each protein. Protein identifications were accepted if MASCOT score was ≥ 70, and the sequence coverage was ≥ 20% or the number of matched peptides (NMP) was ≥ five [84,85]. DAPs were classified according to KEGG (http://www.kegg.jp/), GO (http://www.geneontology.org/) and Uniprot (http://www.uniprot.org/) databases [86,87].

### 4.5. KEGG Pathway Analysis of DAPs

KEGG pathway was analyzed using KOBAS 3.0 (Peking University, Beijing, China). Pathways were considered as significantly enriched if the corrected *p*-value was less than 0.05

### 4.6. qRT-PCR Analysis

Total RNA were extracted from ~300 mg frozen of leaves (mixed sample from four seedlings, one seedling per pot) using Recalcirtant Plant Total RNA Extraction Kit (Bioteke Corporation, Beijing, China). There were three biological replicates per treatment (a total of 12 seedlings from 12 pots). The sequences of specific primers designed using Primer Primier Version 5.0 (PREMIER Biosoft International, CA, USA), were listed in Table S4. qRT-PCR was performed with three biological and two technical replicates [86]. Two *Citrus* genes: *U4/U6 small nuclear ribonucleoprotein PRP31* (*PRP31*, Cs7g08440.1) and *actin* (Cs1g05000.1) were used as internal standards and 0.5 μM Cu-treated leaves were used as reference (set as 1).

### 4.7. Data Analysis

There were 15 pots (30 seedlings) per treatment in a completely randomized design. Results were presented as the mean ± SE for *n* = 3–10. Eight means [two (species) × four (Cu levels)] were tested by two ANOVA followed by the least significant difference at *p* < 0.05 level.

Pearson correlation analysis and principal component analysis (PCA) for all identified DAP spots were made using SPSS (version 17.0, IBM, NY, USA) [88].

### 4.8. Data Deposit

The mass spectrometry proteomics data have been deposited to the ProteomeXchange Consortium via the PRIDE partner repository with the dataset identifier PXD017049.

## 5. Conclusions

**Figure 5:**
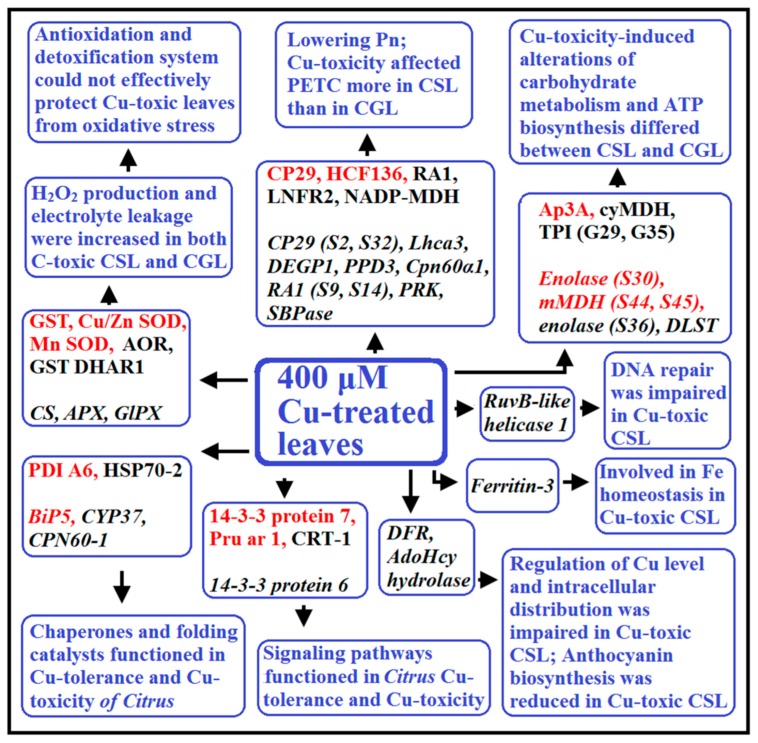
A possible model displaying the differentially abundant proteins (DAPs) in 400 μM Cu-treated *Citrus grandis* leaves (CGL) and *Citrus sinensis* leaves (CSL). In this Figure, plain format and italics were used for Cu-toxic CGL and CSL, respectively. Red: DAPs increased in abundance; Black: DAPs decreased in abundances; APX, L-ascorbate peroxidase; DLST: dihydrolipoyllysine-residue succinyltransferase component of 2-oxoglutarate dehydrogenase complex 2; GlPX: glutathione peroxidase; Pn: photosynthesis; RA1: Rubisco activase A1.

In this study, a 2-DE based MS approach was used to investigate Cu-toxicity-responsive proteins in *Citrus* leaves. Forty-one and 37 DAP spots were identified in 200, 300 and/or 400 μM Cu-treated *C. grandis* and *C. sinensis* leaves, respectively. Over 50% of these DAPs were involved in photosynthesis, carbohydrate, and energy metabolism, followed by antioxidation and detoxification, protein folding and assembly (viz., chaperones and folding catalysts), and signal transduction. More than 80% of these DAPs were identified only in *C. grandis* or *C. sinensis leaves*. More (Less) DAPs increased in abundances than DAPs decreased in abundances were identified in Cu-treated *C. grandis* (*C. sinensis*) leaves. Impaired PETC and decreased abundances of proteins involved in CO_2_ assimilation might be responsible for the Cu-induced inhibition of photosynthesis. Cu-toxicity affected the PETC more in *C. sinensis* leaves than in *C. grandis* leaves. DAPs related to antioxidation and detoxification, protein folding and assembly (viz., chaperones and folding catalysts), and signal transduction might be involved in *Citrus* Cu-toxicity and Cu-tolerance. Also, we identified some new DAPs (viz., LFNR2, SBPase, probable PGL4, ferritin, AdoHcy hydrolase and abscisic stress-ripening protein 1-like) that were not reported in leaves and/or roots (Figure 5). In conclusion, this study revealed some novel mechanisms on Cu-toxicity and Cu-tolerance in plants.

## Figures and Tables

**Table 1:** Protein yield, number of differentially abundant protein (DAP) spots and number of identified DAP spots in 0.5 (control), 200 (Cu200), 300 (Cu300) or 400 (Cu400) Cu-treated *Citrus grandis* and *C. sinensis* leaves.

	*Citrus grandis*					*Citrus sinensis*				
	Control	Cu200	Cu300	Cu400	Total	Control	Cu200	Cu300	Cu400	Total
Protein yield (mg g^−1^DW)	49.4 ± 5.1a	45.3 ± 0.7a	47.6 ± 1.2a	44.7 ± 8.3a		44.4 ± 5.0a	43.8 ± 5.4a	40.9 ± 5.4a	35.6 ± 3.1a	
Number of spots per gel	613 ± 4a	627 ± 8a	621 ± 12a	621 ± 22a		614 ± 7a	625 ± 15a	617 ± 12a	618 ± 9a	
Number of DAP spots										
Increased in abundances		15	12	13			2	4	5	
Decreased in abundances		4	2	7			8	12	18	
Disappeared				2			2	1	13	
Total		19	14	22	42		12	17	36	45
Number of identified DAP spots										
Increased in abundances		15	12	12			2	4	5	
Decreased in abundances		4	2	7			7	11	12	
Disappeared				2			2	1	11	
Total		19	14	21	41		11	16	28	37

Note: Means (±SE, *n* = 3) with a row followed by the same letter are not significant different at *p* < 0.05.

**Table 2:** Differentially abundant protein (DAP) spots and their identification by MALDI-TOF/TOF-MS in 0.5 (control), 200 (Cu200), 300 (Cu300) or 400 (Cu400) Cu-treated *Citrus grandis* and *C. sinensis* leaves.

Spot No.	Protein Identity	Accession No	Mr(kDa)/PI Exp.	Mr(kDa)/PI Theor.	Protein Score	Peptide Ions	NMP	Ratio				CS (%)	*Charge*
								Cu0.5	Cu200	Cu300	Cu400		
***Citrus Grandis***													
**Photosynthesis, Carbohydrate and Energy Metabolism**													
G1	29 kDa ribonucleoprotein A, chloroplastic; Ribonucleoprotein At2g37220, chloroplastic	Cs6g11900.1	30.37/5.17	36.20/4.33	358	186	9	1.00 ± 0.11c	1.68 ± 0.19ab	1.20 ± 0.14bc	2.24 ± 0.26a	8	1
G30	Photosystem II stability/assembly factor HCF136, chloroplast, putative	Cs7g13970.1	45.06/8.46	48.11/5.91	902	42	22	1.00 ± 0.11b	1.19 ± 0.20b	1.17 ± 0.13b	1.91 ± 0.12a	19	1
G31	Photosystem II stability/assembly factor HCF136, chloroplast, putative	Cs7g13970.1	53.11/5.75	59.08/5.94	684	172	28	1.00 ± 0.23b	1.23 ± 0.25b	2.40 ± 0.19a	1.58 ± 0.21b	24	1
G10	Ferredoxin—NADP reductase, leaf-type isozyme, chloroplastic	Cs1g25510.4	55.49/5.09	62.32/4.92	74	47	14	1.00 ± 0.11a	0.53 ± 0.08b	0.69 ± 0.10ab	0.36 ± 0.16b	12	1
G42	Ferredoxin—NADP reductase, leaf-type isozyme, chloroplastic	Cs1g25510.4	40.48/8.68	35.76/6.62	458	122	26	1.00 ± 0.23b	3.66 ± 0.24a	1.64 ± 0.10b	1.54 ± 0.32b	23	1
G8	RuBisCO subunit binding-protein alpha subunit, chloroplast, putative, expressed; Chaperonin 60 subunit alpha 1, chloroplastic	Cs8g16040.3	61.50/5.23	68.61/4.72	1350	169	47	1.00 ± 0.22b	0.31 ± 0.09c	1.86 ± 0.24a	1.06 ± 0.13b	41	1
G9	Ribulose bisphosphate carboxylase/oxygenase activase 1, chloroplastic	Cs7g31800.3	47.86/6.29	53.65/5.10	617	119	17	1.00 ± 0.20ab	1.38 ± 0.05a	0.80 ± 0.08bc	0.48 ± 0.08c	24	1
G6	Sedoheptulose-1,7-bisphosphatase, chloroplastic	Cs7g31640.4	42.40/5.82	48.51/4.64	489	84	29	1.00 ± 0.22b	1.66 ± 0.20a	1.09 ± 0.18ab	1.32 ± 0.10ab	25	1
G38	Glyceraldehyde-3-phosphate dehydrogenase B, chloroplastic	Cs3g27520.2	48.00/7.10	52.45/6.38	515	104	30	1.00 ± 0.27b	2.43 ± 0.31a	1.42 ± 0.08b	1.34 ± 0.14b	26	1
G29	Triosephosphate isomerase, cytosolic (Fragment)	Cs5g16495.1	26.96/5.73	32.66/6.15	326	129	7	1.00 ± 0.04ab	1.58 ± 0.36a	0.63 ± 0.12b	0.18 ± 0.10c	6	1
G35	Triosephosphate isomerase, cytosolic	Cs7g32500.1	26.96/5.73	31.99/6.16	236	125	14	1.00 ± 0.34a	0.69 ± 0.14a	0.51 ± 0.24a	0	11	1
G41	Triosephosphate isomerase, cytosolic	Cs8g18560.2	27.24/5.75	33.66/6.67	428	102	17	1.00 ± 0.14b	1.97 ± 0.27a	1.49 ± 0.05ab	1.16 ± 0.19b	15	1
G4	Probable 6-phosphogluconolactonase 4, chloroplastic	Orange1.1t02542.1	35.38/6.24	34.54/4.72	1050	179	25	1.00 ± 0.10b	2.05 ± 0.35a	2.04 ± 0.28a	1.30 ± 0.20ab	20	1
G36	Fructose-1,6-bisphosphatase, cytosolic	Cs3g21280.1	37.65/5.95	47.35/6.37	408	80	22	1.00 ± 0.42b	4.05 ± 1.70a	2.31 ± 0.11ab	1.63 ± 0.18ab	19	1
G17	Malate dehydrogenase [NADP], chloroplastic	Cs7g21820.2	47.97/6.37	52.65/5.59	654	104	29	1.00 ± 0.28a	0.71 ± 0.05ab	1.01 ± 0.02a	0.32 ± 0.15b	25	1
G37	Malate dehydrogenase, cytoplasmic	Cs9g10470.1	35.54/6.10	48.01/6.44	212	63	15	1.00 ± 0.28b	1.66 ± 0.13a	1.01 ± 0.01b	0.82 ± 0.15b	13	1
G39	Malate dehydrogenase, cytoplasmic	Cs9g10470.1	42.35/5.94	56.49/6.33	193	144	11	1.00 ± 0.21b	2.76 ± 0.46a	2.11 ± 0.20a	2.35 ± 0.48a	10	1
G18	ATP synthase subunit beta, mitochondrial	Cs2g13550.1	59.85/6.06	61.56/5.45	1240	203	21	1.00 ± 0.11bc	0.73 ± 0.20c	1.66 ± 0.12a	1.40 ± 0.11ab	18	1
G23	ATP synthase gamma chain, chloroplastic	Cs2g03080.1	40.62/6.08	45.19/5.67	594	115	25	1.00 ± 0.08bc	0.64 ± 0.20c	1.65 ± 0.09a	1.38 ± 0.04ab	22	1
G26	Bis(5’-adenosy l)-triphosphatase	Cs9g13060.1	17.37/5.94	18.09/5.99	216	105	7	1.00 ± 0.07b	1.03 ± 0.37b	1.47 ± 0.09ab	1.86 ± 0.14a	14	1
G24	Glucose-1-phosphate adenylyltransferase small subunit 2, chloroplastic	Cs2g18800.1	57.08/6.74	58.03/5.66	1120	121	45	1.00 ± 0.12b	1.97 ± 0.42a	2.24 ± 0.27a	1.64 ± 0.31ab	39	1
G25	Glucose-1-phosphate adenylyltransferase small subunit 2, chloroplastic	Cs2g18800.1	65.86/8.50	68.69/5.67	751	139	37	1.00 ± 0.18b	2.11 ± 0.43a	1.09 ± 0.08b	1.36 ± 0.19ab	32	1
**Antioxidation and Detoxification**													
G40	Glutathione S-transferase	Cs5g32800.1	23.83/6.17	30.07/6.75	389	100	20	1.00 ± 0.13b	78.9 ± 28.10a	93.20 ± 15.40a	47.10 ± 8.39a	17	1
G34	Glutathione S-transferase DHAR1, mitochondrial	Cs7g28340.4	23.85/6.18	30.34/6.46	544	127	21	1.00 ± 0.41a	0.57 ± 0.33a	0.26 ± 0.06a	0	18	1
G21	Copper/zinc superoxide dismutase (Fragment)	Cs3g12000.1	15.09/5.47	19.86/5.80	83	46	7	1.00 ± 0.07b	1.03 ± 0.41b	1.18 ± 0.16b	2.43 ± 0.51a	12	1
G33	Manganese superoxide dismutase (Fragment)	Cs7g29850.1	25.29/6.79	28.64/6.34	520	107	19	1.00 ± 0.08b	1.51 ± 0.15ab	1.47 ± 0.16ab	2.02 ± 0.47a	17	1
G16	Quinone oxidoreductase-like protein At1g23740, chloroplastic	Cs7g08640.2	41.88/8.77	48.90/5.38	728	189	27	1.00 ± 0.12a	1.17 ± 0.08a	1.01 ± 0.04a	0.66 ± 0.03b	23	1
**Chaperones and Folding Catalysts**													
G15	Probable protein disulfide-isomerase A6	Cs5g33860.2	41.75/6.91	44.67/5.58	522	130	28	1.00 ± 0.11b	1.45 ± 0.18ab	1.49 ± 0.03ab	1.60 ± 0.21a	24	1
G12	20 kDa chaperonin, chloroplastic	Cs4g07030.2	26.59/8.89	30.93/5.32	874	186	27	1.00 ± 0.18b	0.69 ± 0.13b	1.66 ± 0.08a	0.73 ± 0.19b	23	1
G11	Heat shock cognate 70 kDa protein 2	Cs7g29010.1	70.99/5.09	74.6/4.89	794	113	5	1.00 ± 0.18a	0.81 ± 0.12ab	0.52 ± 0.12b	0.56 ± 0.04b	4	1
G19	Chaperonin CPN60-1, mitochondrial, putative, expressed	Orange1.1t01459.2	61.73/5.85	68.57/5.40	632	60	46	1.00 ± 0.23b	0.46 ± 0.04c	1.76 ± 0.06a	0.81 ± 0.22bc	40	1
**Signal Transduction**													
G3	Calreticulin-1	Cs3g15060.3	52.52/6.29	62.84/4.12	122	99	11	1.00 ± 0.33a	0.72 ± 0.17ab	0.54 ± 0.08ab	0.19 ± 0.02b	15	1
G27	Major allergen Pru ar 1 (Major pollen allergen Bet v 1-D/H; Major pollen allergen Bet v 1-A)	Cs9g03630.1	17.60/5.67	21.51/6.05	253	104	19	1.00 ± 0.09c	3.02 ± 0.42a	1.66 ± 0.28bc	2.17 ± 0.49ab	17	1
G5	14-3-3 protein 7 (14-3-3-like protein GF14 epsilon)	Cs3g18200.2	28.86/4.92	37.48/4.61	670	174	25	1.00 ± 0.20b	1.36 ± 0.08ab	1.39 ± 0.13ab	1.83 ± 0.16a	21	1
G22	Annexin D1	Cs3g18360.1	35.88/5.17	46.63/5.66	784	138	31	1.00 ± 0.72b	3.18 ± 1.21ab	4.77 ± 0.99a	2.03 ± 0.43ab	27	1
**Cellular Transport**													
G28	Ferritin-2, chloroplastic	Cs7g30630.1	29.47/5.41	32.63/5.90	499	155	27	1.00 ± 0.12a	0.55 ± 0.12b	0.88 ± 0.05ab	1.21 ± 0.16a	23	1
Cell Wall and Cytoskeleton													
G7	Tubulin beta-6 chain	Cs3g26180.1	50.38/4.75	62.28/4.68	775	162	30	1.00 ± 0.10b	3.48 ± 0.55a	1.59 ± 0.08b	1.23 ± 0.39b	26	1
G2	Endochitinase 1	Cs8g01850.1	35.39/4.85	52.52/4.16	75	33	9	1.00 ± 0.16a	0.55 ± 0.27ab	0.44 ± 0.05b	0.75 ± 0.09ab	8	1
**Stress Response**													
G14	Abscisic stress-ripening protein 1-like	Cs3g21500.1	17.88/6.00	30.30/5.56	323	123	8	1.00 ± 0.35b	1.75 ± 0.48b	1.97 ± 0.10b	5.75 ± 0.59a	29	1
**Others**													
G20		Orange1.1t05091.1	157.30/6.83	19.29/5.70	161	12	31	1.00 ± 0.16c	1.38 ± 0.32bc	1.80 ± 0.26ab	2.31 ± 0.19a	30	1
G32	S-adenosyl-L-homocysteine hydrolase (adenosylhomocysteinase)	Orange1.1t01892.1	80.71/6.26	77.69/6.12	577	97	35	1.00 ± 0.18b	2.72 ± 0.72a	0.87 ± 0.09b	0.79 ± 0.10b	3	1
*Unidentified Protein Spots*													
G13	Receptor serine-threonine protein kinase, putative	Cs9g04750.2	25.57/8.87	23.24/5.56	45	109	13	1.00 ± 0.15b	1.06 ± 0.28b	1.13 ± 0.25b	2.12 ± 0.35a	22	1
***Citrus Sinensis***													
**Photosynthesis, Carbohydrate and Energy Metabolism**													
S19	Chlorophyll a-b binding protein 8,chloroplastic	Cs3g06180.2	29.52/6.84	32.89/5.42	222	95	12	1.00 ± 0.25b	1.80 ± 0.27a	1.28 ± 0.05ab	0.44 ± 0.09c	10	1
S41	Protease Do-like 1, chloroplastic	Cs2g28080.1	53.11/5.75	95.23/4.75	553	139	17	1.00 ± 0.15a	0.12 ± 0.05c	0.68 ± 0.04ab	0.51 ± 0.10bc	15	1
S13	PsbP domain-containing protein 3, chloroplastic	Cs3g27720.1	27.63/8.28	21.66/5.68	366	126	9	1.00 ± 0.17a	0.81 ± 0.11ab	0.55 ± 0.10bc	0.36 ± 0.11c	12	1
S2	29 kDa ribonucleoprotein A, chloroplastic; Ribonucleoprotein At2g37220, chloroplastic	Cs6g11900.1	30.37/5.17	45.1/6.21	418	195	13	1.00 ± 0.08a	0.82 ± 0.16ab	0.43 ± 0.09c	0.53 ± 0.06bc	11	1
S32	29 kDa ribonucleoprotein A, chloroplastic; Ribonucleoprotein At2g37220, chloroplastic	Cs7g01430.1	28.53/7.78	33.75/5.11	392	114	19	1.00 ± 0.20a	0.76 ± 0.02ab	0.49 ± 0.14b	0	23	1
S17	Oxygen-evolving enhancer protein 1, chloroplastic	Cs1g23450.1	35.38/5.83	24.74/5.56	261	116	7	1.00 ± 0.05c	1.06 ± 0.01bc	1.34 ± 0.10ab	1.54 ± 0.14a	19	1
S3	Carbonic anhydrase, chloroplastic	Cs2g28060.4	36.77/6.66	53.09/6.25	171	162	5	1.00 ± 0.26c	3.02 ± 0.38ab	3.14 ± 0.23a	1.18 ± 0.35bc	5	1
S11	Rubisco subunit binding-protein alpha subunit, chloroplast, putative, expressed; Chaperonin 60 subunit alpha 1, chloroplastic	Cs8g16040.1	61.50/5.23	99.02/5.94	1250	182	39	1.00 ± 0.1a	0.70 ± 0.16a	0.80 ± 0.17a	0.27 ± 0.04b	34	1
S9	Ribulose bisphosphate carboxylase/oxygenase activase 1, chloroplastic	Cs7g31800.3	50.90/5.33	81.78/6.10	505	107	21	1.00 ± 0.23a	0.75 ± 0.10ab	0.66 ± 0.10ab	0.51 ± 0.04b	28	1
S14	Ribulose bisphosphate carboxylase/oxygenase activase 1, chloroplastic	Cs7g31800.3	46.96/5.94	75.7/5.65	505	107	21	1.00 ± 0.11a	0.65 ± 0.02b	0.48 ± 0.05b	0.45 ± 0.05b	28	1
S4	Ribulose bisphosphate carboxylase/oxygenase activase 1, chloroplastic	Cs7g31800.3	46.96/5.94	83.26/6.21	579	139	19	1.00 ± 0.46a	0	0.38 ± 0.03a	0.77 ± 0.06a	17	1
S10	Ribulose bisphosphate carboxylase/oxygenase activase 1, chloroplastic	Cs7g31800.3	46.96/5.94	79.78/6.02	641	175	24	1.00 ± 0.16b	1.38 ± 0.30b	2.16 ± 0.23a	1.05 ± 0.04b	21	1
S21	Phosphoribulokinase, chloroplastic	Cs3g08480.1	45.19/5.97	67.41/5.58	686	137	31	1.00 ± 0.05a	1.01 ± 0.04a	1.02 ± 0.15a	0	27	1
S33	Sedoheptulose-1,7-bisphosphatase, chloroplastic	Cs7g31640.4	36.77/6.66	43.56/4.96	576	101	27	1.00 ± 0.06a	1.02 ± 0.06a	0.75 ± 0.24a	0	23	1
S44	Malate dehydrogenase, mitochondrial	Cs7g25390.1	35.48/8.52	66.81/4.32	613	144	24	1.00 ± 0.02b	1.18 ± 0.21ab	0.95 ± 0.15b	1.64 ± 0.26a	21	1
S45	Malate dehydrogenase, mitochondrial	Cs7g25390.3	37.65/5.95	63.69/4.53	287	171	9	1.00 ± 0.16bc	1.15 ± 0.2b	0.46 ± 0.21c	1.85 ± 0.22a	8	1
S30	Enolase	Cs6g15540.1	15.09/5.47	18.14/4.99	928	154	30	1.00 ± 0.12a	0.53 ± 0.07b	0.63 ± 0.09b	0.22 ± 0.08c	26	1
S36	Enolase	Cs6g15540.1	47.79/5.54	89.59/5.04	531	136	15	1.00 ± 0.25b	1.04 ± 0.22b	1.78 ± 0.13a	1.92 ± 0.11a	47	1
S43	Dihydrolipoyllysine-residue succinyltransferase component of 2-oxoglutarate dehydrogenase complex 2, mitochondrial	Cs2g21190.3	40.39/6.95	87.84/4.79	268	144	8	1.00 ± 0.26a	0.56 ± 0.20ab	0.30 ± 0.06b	0	25	1
**Antioxidation and Detoxification**													
S1	2-Cys peroxiredoxin BAS1, chloroplastic	Cs6g13880.1	29.49/7.65	30.56/6.56	465	161	17	1.00 ± 0.10a	0.89 ± 0.11a	0	0.81 ± 0.19a	15	1
S20	Cysteine synthase, chloroplastic/chromoplastic	Orange1.1t02144.1	41.35/8.29	56.87/5.55	880	197	28	1.00 ± 0.14a	0	0.91 ± 0.09a	1.17 ± 0.06a	21	1
S39	Cysteine synthase	Cs9g06970.1	29.29/6.78	60.56/4.69	114	49	6	1.00 ± 0.28a	1.04 ± 0.12a	0.78 ± 0.15a	0	17	1
S34	L-ascorbate peroxidase 1, cytosolic	Cs8g17370.1	28.68/5.42	46.45/5.05	392	114	19	1.00 ± 0.05a	0.82 ± 0.18a	0.77 ± 0.14a	0.35 ± 0.01b	23	1
S24	Glutathione peroxidase (Fragment)	Cs5g03830.1	18.58/5.72	25.08/5.15	646	135	21	1.00 ± 0.14a	1.02 ± 0.07a	0.88 ± 0.16ab	0.55 ± 0.12b	30	1
**Chaperones and Folding Catalysts**													
S16	Luminal-binding protein 5	Cs5g01840.2	73.56/5.09	108.09/5.72	578	112	27	1.00 ± 0.18b	1.07 ± 0.21b	2.57 ± 0.19a	2.39 ± 0.23a	23	1
S8	Peptidyl-prolylcis-transisomerase CYP37, chloroplastic	Cs1g06710.1	50.39/6.42	58.95/5.92	109	92	5	1.00 ± 0.13a	1.03 ± 0.07a	0.73 ± 0.05ab	0.66 ± 0.12b	2	1
S26	Chaperonin CPN60-1, mitochondrial, putative, expressed	Orange1.1t01459.2	46.12/8.24	52.38/5.28	727	152	39	1.00 ± 0.13a	0.90 ± 0.14a	1.16 ± 0.12a	0	34	1
**Signal Transduction**													
S35	Major allergen Pru ar 1 (Major pollen allergen Bet v 1-D/H; Major pollen allergen Bet v 1-A)	Cs9g03630.1	48.33/6.19	89.59/5.09	230	94	15	1.00 ± 0.07a	0.57 ± 0.15b	1.20 ± 0.13a	0.91 ± 0.05ab	13	1
S7	14-3-3 protein 6	Orange1.1t01991.1	29.44/4.84	45.11/6.09	439	137	18	1.00 ± 0.18a	0.28 ± 0.02b	0.51 ± 0.13ab	0.54 ± 0.04ab	16	1
**Cellular Transport**													
S5	Ferritin-3, chloroplastic	Cs6g09150.2	28.97/5.46	41.03/5.97	406	129	13	1.00 ± 0.03a	0.65 ± 0.03b	0.61 ± 0.12b	0.46 ± 0.03b	33	1
**Nucleic acid Metabolism**													
S42	RuvB-like helicase 1	Cs6g16920.1	38.08/6.90	90.61/4.73	268	99	16	1.00 ± 0.17a	0.82 ± 0.33a	0.56 ± 0.08a	0	25	1
**Others**													
S27		Orange1.1t05091.1	53.64/5.26	85.72/5.32	246	73	19	1.00 ± 0.38a	0.15 ± 0.01b	0.23 ± 0.04b	0.44 ± 0.18ab	17	1
S28		Orange1.1t05091.1	61.73/5.85	95.17/5.28	212	70	16	1.00 ± 0.24a	0.91 ± 0.15a	0.32 ± 0.05b	0.60 ± 0.07ab	14	1
S31		Orange1.1t05091.1	177.77/7.11	24.86/4.86	170	45	15	1.00 ± 0.07a	0.75 ± 0.15ab	0.53 ± 0.12b	0	14	1
S23	Anthranilate N-methyltransferase	Cs5g24940.1	39.48/5.20	23.56/5.23	300	146	17	1.00 ± 0.03a	0.93 ± 0.02a	0.27 ± 0.10b	0	15	1
S37	S-adenosyl-L-homocysteine hydrolase (adenosylhomocysteinase)	Orange1.1t01892.1	17.60/5.67	26.91/4.83	776	172	24	1.00 ± 0.12a	1.18 ± 0.07a	0.73 ± 0.24a	0	21	1
S38	Dihydroflavonol-4-reductase	Cs3g01140.1	15.15/4.94	43.76/4.84	396	152	14	1.00 ± 0.07a	1.28 ± 0.35a	2.04 ± 0.45a	0	12	1
***Unidentified Protein Spots***													
S6	Light-harvesting chlorophyll-a/b binding protein Lhca6 (Fragment)	Cs7g27290.1	26.56/5.43	35.60/5.91	105	50	4	1.00 ± 0.24a	0.87 ± 0.06ab	0.71 ± 0.09ab	0.49 ± 0.02b	31	1
S12	Thioredoxin M-type, chloroplastic	Cs3g20630.1	19.91/8.83	16.79/5.85	60	43	4	1.00 ± 0.18a	0.95 ± 0.13a	0.80 ± 0.08ab	0.40 ± 0.17b	7	1
S15	Nicotinate-nucleotide pyrophosphorylase [carboxylating], putative	Orange1.1t04780.1	55.49/5.09	91.64/5.82	50	131	26	1.00 ± 0.26a	0.79 ± 0.18ab	0.59 ± 0.04ab	0.30 ± 0.02b	58	1
S18	Disease resistance protein RFL1, putative	Cs3g08210.1	49.77/9.44	31.35/5.42	50	18	10	1.00 ± 0.20a	0.39 ± 0.13b	0.33 ± 0.04b	0.19 ± 0.1b	17	1
S22	Dehydration-responsive family protein, putative, expressed	Orange1.1t00308.3	49.93/5.04	88.43/5.57	57	117	24	1.00 ± 0.19a	0.94 ± 0.06ab	0.87 ± 0.02ab	0.65 ± 0.07b	57	1
S25	Transducin/WD40 domain-containing protein-like protein	Cs9g09840.1	29.76/6.18	31.98/5.16	67	41	9	1.00 ± 0.14a	0.83 ± 0.15a	0.51 ± 0.12a	0	8	1
S29	ATPase 8, plasma membrane-type	Cs4g01370.1	14.72/5.41	22.10/5.07	68	98	11	1.00 ± 0.09a	0.90 ± 0.07ab	0.72 ± 0.05bc	0.50 ± 0.09c	21	1
S40	4-hydroxy-3-methylbut-2-enyl diphosphate reductase	Cs5g28200.1	52.22/6.60	81.69/4.72	63	49	5	1.00 ± 0.16a	0.68 ± 0.06a	0.66 ± 0.14a	0	17	1

Note: Spot number corresponds to the 2-DE imagines in Figure 2. Ratio means the ratio of 0.5, 200, 300 or 400 μM Cu-treated leaves to 0.5 μM Cu-treated leaves. NMP: the number of matched peptides; CS: covered sequence. Means (±SE, *n* = 3) with a row followed by different letters are significant different at *p* < 0.05.

## Supplementary Materials

The following are available online at https://www.mdpi.com/2223-7747/9/3/291/s1, Figure S1: Excess Cu effects on growth of *Citrus grandis* (A) and *Citrus sinensis* (B), Figure S2: Excess Cu effects on root (A), stem (B), leaf (C) and whole plant (D) dry weight (DW) in *Citrus grandis* and *Citrus sinensis* seedlings, Figure S3: 2-DE images of proteins extracted from 0.5 (A, E, I, M), 200 (B, F, J, N) 300 (C, G, K, O) and 400 (D, H, L, P) Cu-treated *Citrus grandis* (A-D and I-L) and *Citrus sinensis* (E-H and M-P) leaves for the other two replicates, Figure S4: Close-up views of 24 DAP spots in 200, 300 and 400 μM Cu-treated *Citrus sinensis* and *Citrus grandis* leaves, Figure S5: Venn diagram analysis of Cu-responsive proteins in *Citrus grandis* and *Citrus sinensis* leaves, Figure S6: Significantly enriched (A-E and H) and the most enriched (F-G) KEGG pathways for annotated differentially abundant proteins (DAPs) in Cu-treated *Citrus grandis* (A-D) and *Citrus sinensis* (E-H) leaves, Figure S7: Matrices of Pearson correlation coefficients for differentially abundant proteins (DAPs) in *Citrus grandis* (A) and *Citrus sinensis* (B) leaves, Figure S8: Relative expression levels of genes encoding 22 differentially abundant proteins (DAPs) identified in 400 μM Cu-treated *Citrus grandis* (G3, G9, G10, G11, G14, G26, G29, G33, G34 and G35) and *Citrus sinensis* (S2, S5, S9, S16, S17, S23, S24, S30, S32, S33, S37 and S43) leaves using *PRPF31* (A) and *actin* (B) as internal standards, Table S1: Master list of proteins identified in MALDI TOF/TOF MS from 200, 300 and or 400 μM Cu-treated *Citrus sinensis* and *Citrus grandis* leaves using 2DE and DIGE experiments, Table S2: Principal component analysis (PCA) for copper-responsive proteins in *Citrus sinensis* leaves, Table S3: Principal component analysis (PCA) for copper-responsive proteins in *Citrus grandis* leaves, Table S4: Specific primer pairs used for qRT-PCR analysis.
