# Supplementary material for: Excess Copper-Induced Alterations of Protein Profiles and Related Physiological Parameters in Citrus Leaves
Source: Plants (Basel). 2020 Feb 28;9(3):291. doi: 10.3390/plants9030291 (PMC7154894; doi:10.3390/plants9030291)
Supplement: Supplementary file 1 [file plants-09-00291-s001.doc]

Supplementary Data


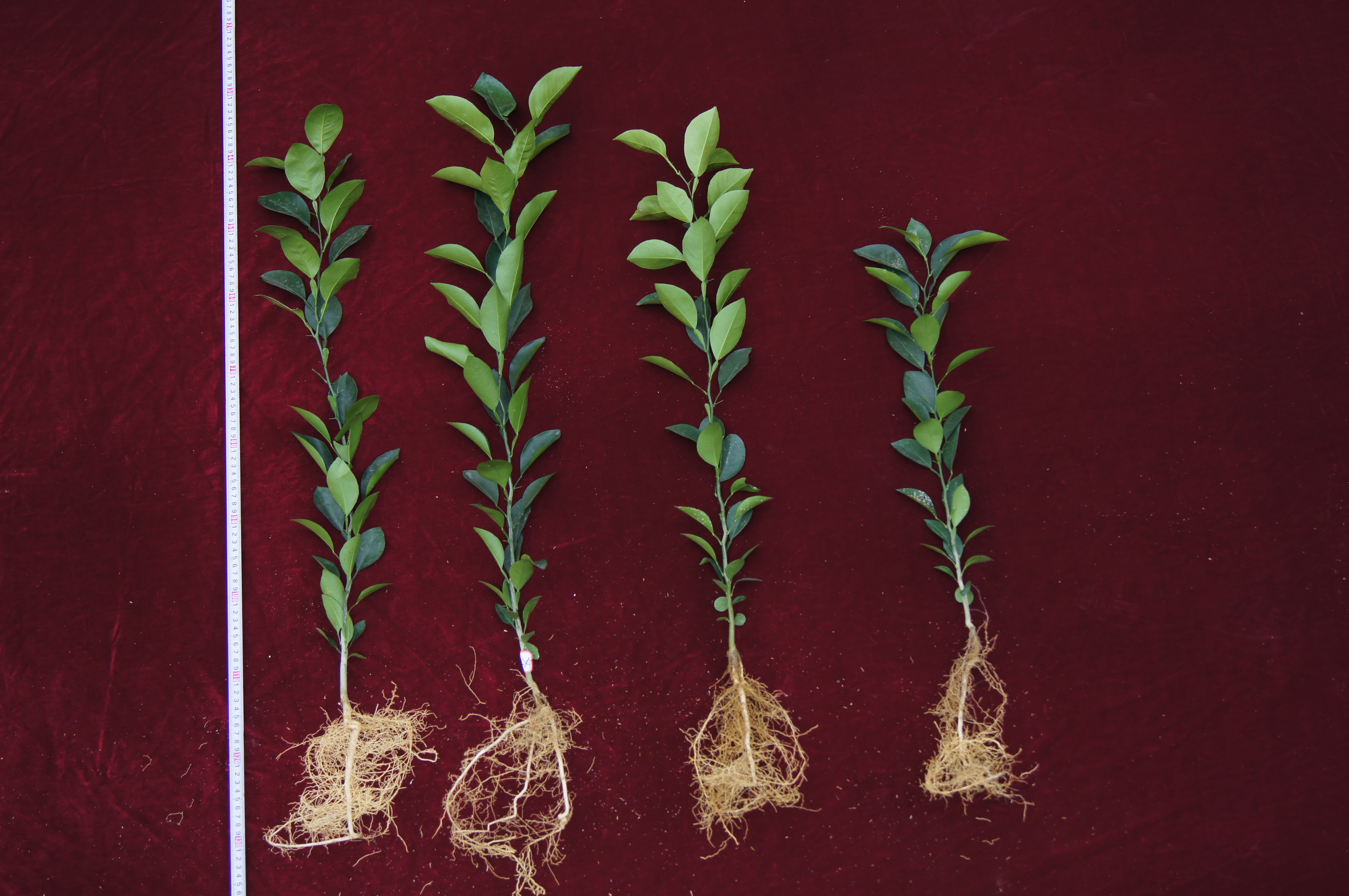

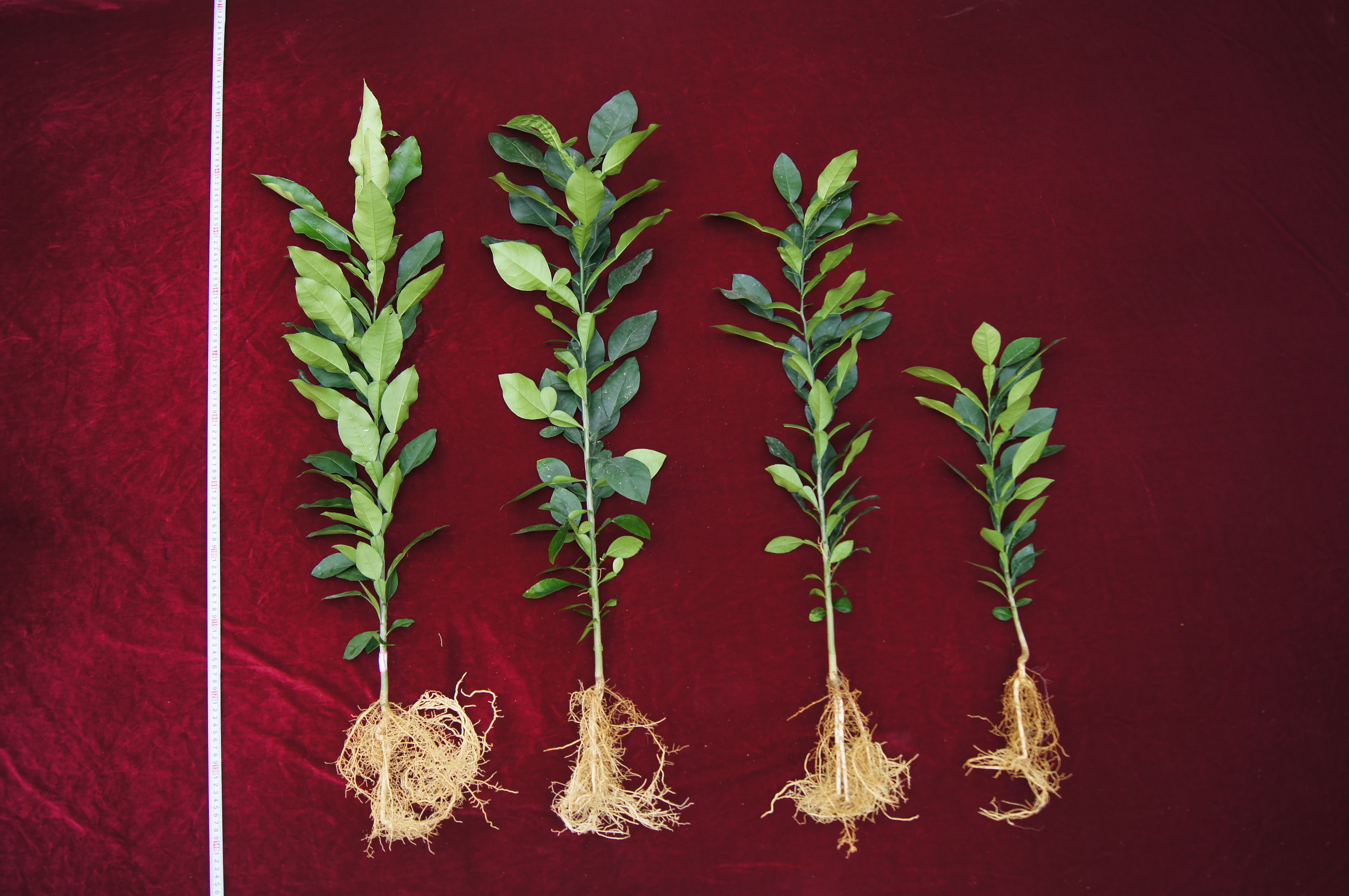


**0.5 200 300 400**

**A Cu treatments (μM)**

**0.5 200 300 400**

**B Cu treatments (μM)**

**Figure S1.** Excess Cu effects on growth of *Citrus grandis* (A) and *Citrus sinensis* (B)

**Figure S2.** Excess Cu effects on root (A), stem (B), leaf (C) and whole plant (D) dry weight (DW) in *Citrus grandis* and *Citrus sinensis* seedlings. Bars represent the means ± SE (*n* = 10). Different letters above the bars indicate a significant difference at *P* < 0.05.


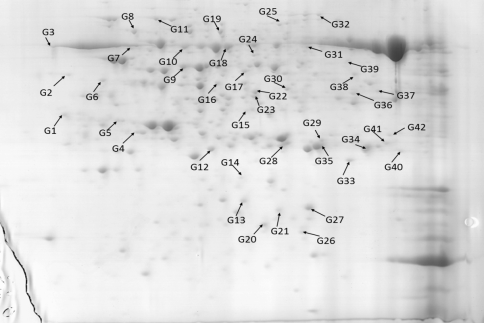

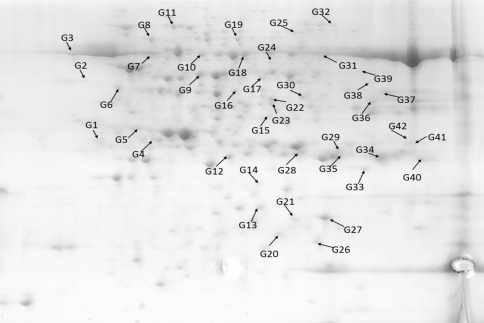

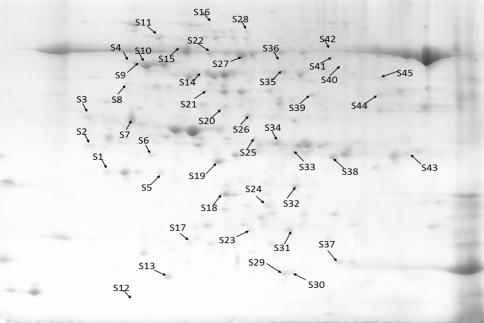

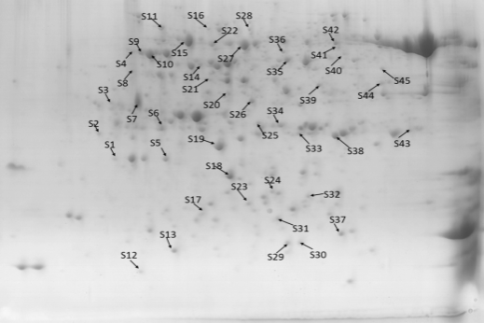

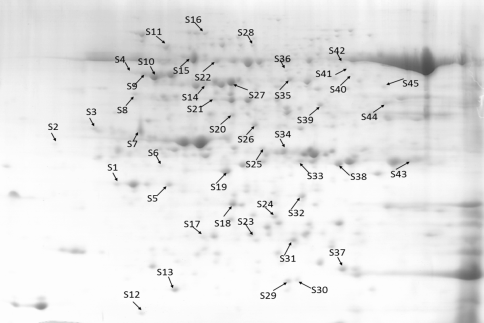

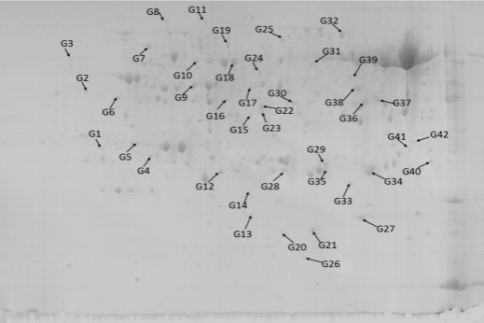

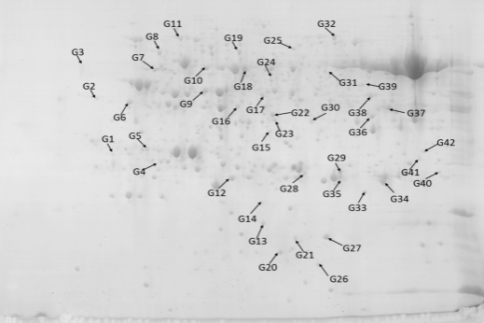

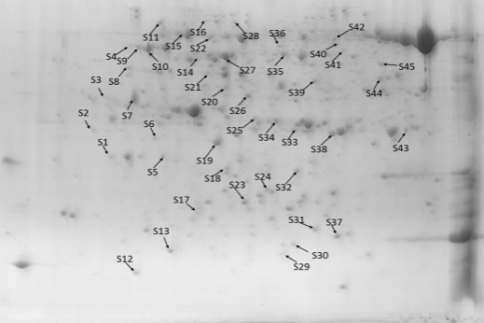

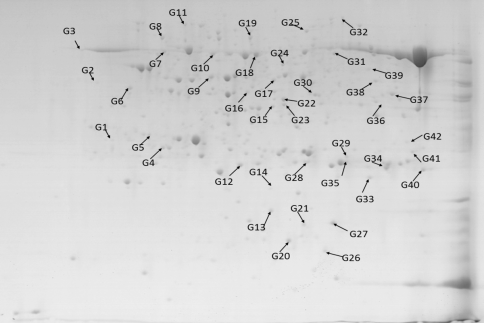

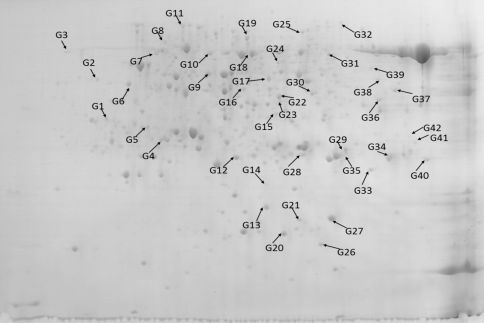

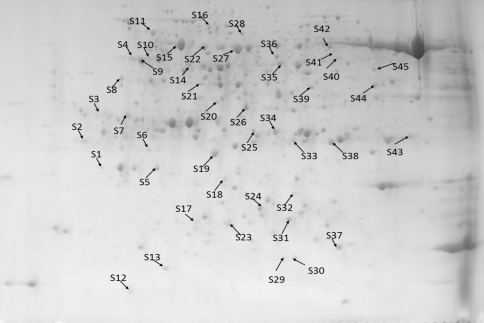

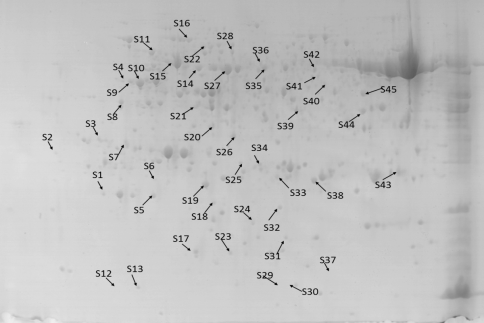

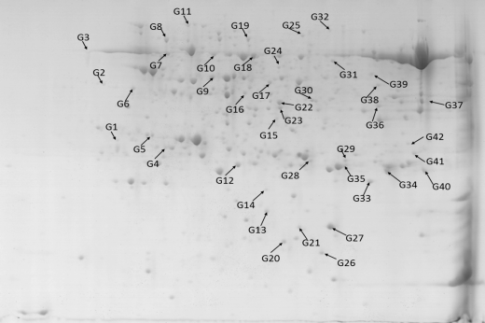

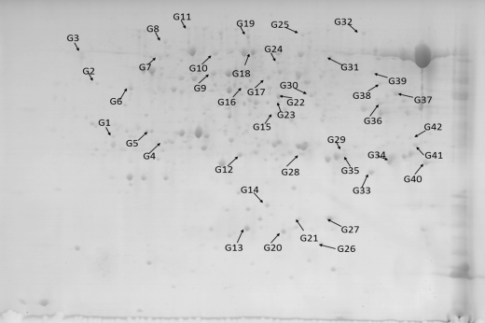

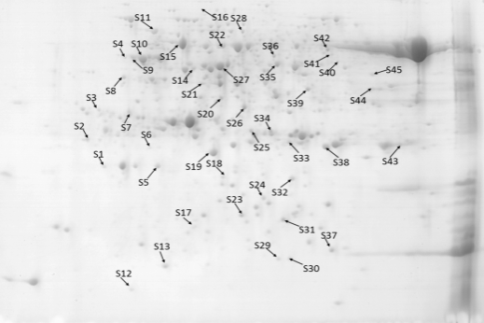

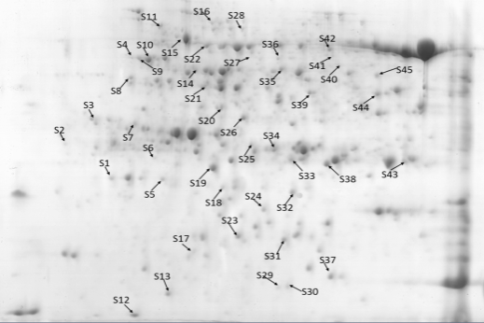


**SDS-PAGE**GE

**SDS-PAGE**

**SDS-PAGE**

**SDS-PAGE**

**pH4**

**pH7**

**pH4**

**pH7**

**pH4**

**pH7**

**pH4**

**pH7**

**A: Cu0.5**

***Citrus grandis***

***Citrus grandis***

***Citrus sinensis***

**B: Cu200**

**C: Cu300**

**D: Cu400**

**E: Cu0.5**

**I: Cu0.5**

**M: Cu0.5**

**F: Cu200**

**J: Cu200**

**N: Cu200**

**G: Cu300**

**K: Cu300**

**O: Cu300**

**H: Cu400**

**L: Cu400**

**P: Cu400**

**Figure S3.** 2-DE images of proteins extracted from 0.5 (A, E, I, M), 200 (B, F, J, N) 300 (C, G, K, O) and 400 (D, H, L, P) Cu-treated *Citrus grandis* (A-D and I-L) and *Citrus sinensis* (E-H and M-P) leaves for the other two replicates.


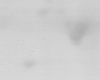

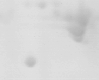

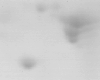

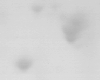

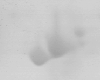

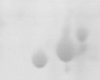

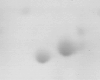

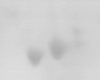

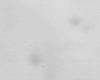

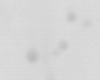

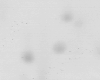

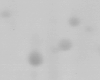

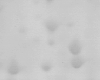

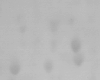

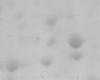

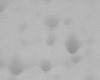

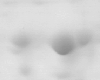

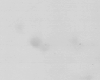

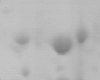

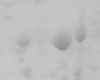

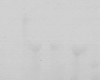

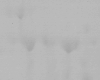

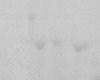

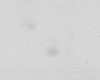

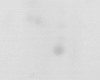

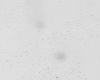

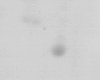

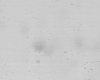

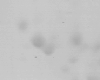

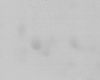

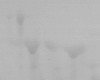

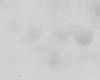

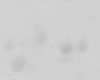

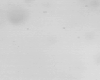

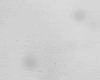

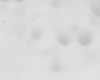

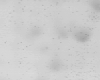

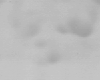

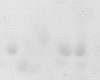

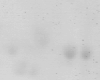

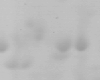

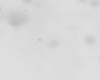

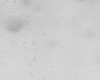

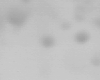

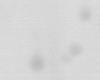

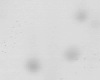

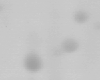

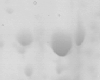

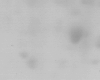

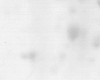

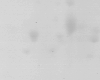

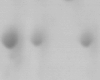

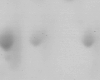

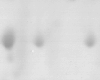

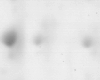

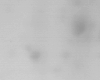

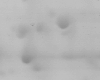

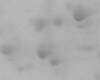

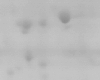

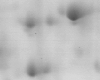

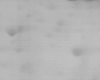

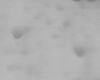

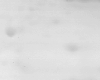

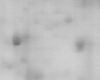

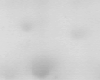

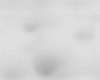

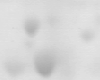

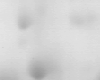

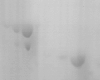

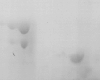

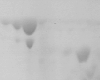

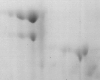

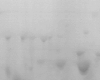

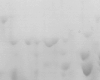

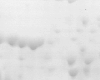

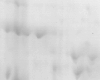

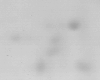

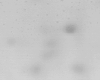

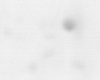

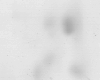

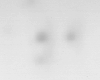

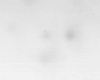


G1

G1

G1

G1

G4

G4

G4

G4

G5

G5

G5

G5

G9

G9

G9

G9

G11

G11

G11

G11

G14

G14

G14

G14

G15

G15

G15

G15

G20

G20

G20

G20

G21

G21

G21

G21

G23

G23

G23

G23

G29

G29

G29

G29

G33

G33

G33

G33

S2

S2

S2

S2

S5

S5

S5

S5

S9

S9

S9

S9

S11

S11

S11

S11

S13

S13

S13

S13

S16

S16

S16

S16

S17

S17

S17

S17

S30

S30

S30

S30

S32

S32

S32

S32

S36

S36

S36

S36

S44

S44

S44

S44

S45

S45

S45

S45

***Citrus grandis Citrus sinensis***

**Cu0.5 Cu200 Cu300 Cu400 Cu0.5 Cu200 Cu300 Cu 400**

**Figure S4.** Close-up views of 24 DAP spots in 200, 300 and 400 μM Cu-treated *Citrus sinensis* and *Citrus grandis* leaves

**E: Cu300 F: Cu400**

**15**

**1**

**13**

***C. grandis* *C. sinensis***

**26**

**2**

**19**

***C. grandis* *C. sinensis***

**C: Total D: Cu200**

**10**

**1**

**18**

***C. grandis* *C. sinensis***

**30**

**7**

**34**

***C. grandis* *C. sinensis***

**2**

**2**

**14**

**Cu400**

**Cu300**

**2**

**3**

**10**

**6**

**Cu200**

**15**

**Cu400**

**Cu300**

**8**

**3**

**3**

**3**

**3**

**Cu200**

**A: *C.* *grandis* B: *C. sinensis***

**4**

**Figure S5.** Venn diagram analysis of Cu-responsive proteins in *Citrus grandis* and *Citrus sinensis* leaves. (A) Differentially expressed protein (DAP) spots in 200, 300 and 400 μM Cu-treated *Citrus grandis* leaves; (B) DAP spots in 200, 300 and 400 μM Cu-treated *C. sinensis* leaves; (C) Total DAPs in *C. grandis* and *C. sinensis* leaves; (D) DAPs in 200 μM Cu-treated *C. grandis* and *C. sinensis* leaves; (E) DAPs in 300 μM Cu-treated *C. grandis* and *C. sinensis* leaves; and (F) DAPs in 400 μM Cu-treated *C. grandis* and *C. sinensis* leaves.

**A: Cu0.5**

**E: Cu0.5**

**B: Cu200**

**F: Cu200**

**C: Cu300**

**G: Cu300**

**D: Cu400**

**H: Cu400**

**Figure S6.** Significantly enriched (A-E and H) and the most enriched (F-G) KEGG pathways for annotated differentially abundant proteins (DAPs) in Cu-treated *Citrus grandis* (A-D) and *Citrus sinensis* (E-H) leaves. (A, E) Total DAPs; (B, F) DAPs in 200 μM Cu-treated leaves; (C, G) DAPs in 300 μM Cu-treated leaves; and (D, H) DAPs in 400 μM Cu-treated leaves.

**Figure S7.** Matrices of Pearson correlation coefficients for differentially abundant proteins (DAPs) in *Citrus grandis* (A) and *Citrus sinensis* (B) leaves

**Figure S8.** Relative expression levels of genes encoding 22 differentially abundant proteins (DAPs) identified in 400 μM Cu-treated *Citrus grandis* (G3, G9, G10, G11, G14, G26, G29, G33, G34 and G35) and *Citrus sinensis* (S2, S5, S9, S16, S17, S23, S24, S30, S32, S33, S37 and S43) leaves using *PRPF31* (A) and *actin* (B) as internal standards, Bars represent means ± SE of three biological replicates and two technique replicates for dependent variables. For the same genes, different letters above the bars indicate a significant difference at *P* < 0.05.

**Table S1.** Master list of proteins identified in MALDI TOF/TOF MS from 200, 300 and or 400 μM Cu-treated *Citrus sinensis* and *Citrus grandis* leaves using 2DE and DIGE experiments.

| **Proteins identified in *Citrus sinensis***  **Photosynthesis, carbohydrate and energy metabolism**   | **S19.** | [Cs3g06180.2](http://localhost/mascot/cgi/protein_view.pl?file=../data/20140808/F003845.dat&hit=1)    **Mass:** 23059    **Score:** 222    **Expect:** 1.8e-018  **Matches:** 12 | | --- | --- |  | **Observed** | **Mr(expt)** | **Mr(calc)** | **ppm** | **Start** |  | **End** | **Miss** | **Ions** | **Peptide** | | --- | --- | --- | --- | --- | --- | --- | --- | --- | --- | | 1031.4973 | 1030.4900 | 1030.5124 | -21.69 | 121 | - | 128 | 0 | --- | R.QYFLGFEK.Y | | 1277.6180 | 1276.6107 | 1276.6564 | -35.78 | 33 | - | 43 | 0 | --- | K.WLAYGEVINGR.Y | | 1277.6180 | 1276.6107 | 1276.6564 | -35.78 | 33 | - | 43 | 0 | 95 | K.WLAYGEVINGR.Y | | 1365.5874 | 1364.5801 | 1364.5932 | -9.56 | 109 | - | 120 | 0 | --- | R.FQDWANPGSMGR.Q | | 1381.5652 | 1380.5579 | 1380.5881 | -21.85 | 109 | - | 120 | 0 | --- | R.FQDWANPGSMGR.Q + Oxidation (M) | | 1381.5652 | 1380.5579 | 1380.5881 | -21.85 | 109 | - | 120 | 0 | 4 | R.FQDWANPGSMGR.Q + Oxidation (M) | | 1521.6901 | 1520.6828 | 1520.6943 | -7.53 | 108 | - | 120 | 1 | --- | R.RFQDWANPGSMGR.Q | | 1537.6665 | 1536.6592 | 1536.6892 | -19.50 | 108 | - | 120 | 1 | --- | R.RFQDWANPGSMGR.Q + Oxidation (M) | | 1689.8812 | 1688.8739 | 1688.9171 | -25.56 | 44 | - | 60 | 0 | --- | R.YAMLGAVGAIAPEILGK.A + Oxidation (M) | | 2247.0925 | 2246.0852 | 2246.1372 | -23.13 | 129 | - | 151 | 0 | --- | K.YLGGSGDPAYPGGPLFNPLGLGK.D | | 2619.2498 | 2618.2425 | 2618.3017 | -22.59 | 129 | - | 154 | 1 | --- | K.YLGGSGDPAYPGGPLFNPLGLGKDEK.S | | 2619.2498 | 2618.2425 | 2618.3017 | -22.59 | 129 | - | 154 | 1 | 91 | K.YLGGSGDPAYPGGPLFNPLGLGKDEK.S |   **No match to:** 712.2439, 738.3864, 804.2685, 832.2852, 832.2852, 842.4905, 1004.5463, 1014.4643, 1014.4643, 1028.4883, 1032.4950, 1036.4619, 1131.5540, 1145.5659, 1146.5635, 1147.5664, 1259.6193, 1260.6080, 1260.6080, 1263.6036, 1264.6033, 1274.6187, 1275.6211, 1276.6157, 1281.6233, 1285.6141, 1287.6133, 1289.6282, 1291.6377, 1292.6312, 1293.6213, 1293.6213, 1299.6161, 1309.6230, 1315.5804, 1316.5831, 1317.5684, 1317.5684, 1321.5773, 1328.6011, 1328.6011, 1332.6318, 1333.6198, 1358.6432, 1358.6432, 1362.6437, 1372.6389, 1373.6462, 1374.6423, 1375.6569, 1379.6151, 1385.5609, 1390.6411, 1391.6547, 1397.5565, 1397.5565, 1407.6561, 1413.5621, 1413.5621, 1428.7157, 1430.7302, 1430.7302, 1431.7278, 1441.5765, 1457.5742, 1457.5742, 1472.7159, 1484.7627, 1486.7885, 1486.7885, 1500.7479, 1502.7295, 1511.6926, 1518.7025, 1520.6873, 1525.6915, 1528.6926, 1534.6788, 1535.6642, 1536.6625, 1541.6674, 1543.6591, 1545.6896, 1551.6681, 1552.6696, 1553.6649, 1555.6859, 1557.6898, 1567.7219, 1569.6981, 1571.7228, 1575.6775, 1588.7919, 1589.7905, 1597.7815, 1613.6862, 1625.8873, 1638.8208, 1711.8586, 1727.8434, 1969.9888, 1991.9821, 2641.2212   | **S41.** | [**Cs2g28080.1**](http://localhost/mascot/cgi/protein_view.pl?file=../data/20140808/F003852.dat&hit=1)**Mass:**46090**Score:**553 **Expect:**2.2e-051**Matches: 17** | | --- | --- |  | **Observed** | **Mr(expt)** | **Mr(calc)** | **ppm** | **Start** |  | **End** | **Miss** | **Ions** | **Peptide** | | --- | --- | --- | --- | --- | --- | --- | --- | --- | --- | | 996.6203 | 995.6130 | 995.6491 | -36.27 | 329 | - | 337 | 0 | --- | K.VTRPILGIK.F | | 1010.4643 | 1009.4570 | 1009.4829 | -25.60 | 393 | - | 401 | 0 | --- | K.VSNGSDLYR.I | | 1145.5513 | 1144.5440 | 1144.6088 | -56.59 | 111 | - | 120 | 0 | --- | K.LQTDELATVR.L | | 1227.6556 | 1226.6483 | 1226.6871 | -31.58 | 408 | - | 418 | 0 | --- | K.VGDEVIVEVLR.G | | 1227.6556 | 1226.6483 | 1226.6871 | -31.58 | 408 | - | 418 | 0 | 59 | K.VGDEVIVEVLR.G | | 1294.6636 | 1293.6563 | 1293.6942 | -29.29 | 163 | - | 173 | 0 | --- | K.GHVVTNYHVIR.G | | 1574.8022 | 1573.7949 | 1573.8464 | -32.72 | 192 | - | 205 | 1 | --- | K.IVGFDQDKDVAVLR.I | | 1574.8022 | 1573.7949 | 1573.8464 | -32.72 | 192 | - | 205 | 1 | 108 | K.IVGFDQDKDVAVLR.I | | 1664.8235 | 1663.8162 | 1663.8893 | -43.94 | 408 | - | 422 | 1 | --- | K.VGDEVIVEVLRGDHK.E | | 1664.8235 | 1663.8162 | 1663.8893 | -43.94 | 408 | - | 422 | 1 | 3 | K.VGDEVIVEVLRGDHK.E | | 1675.8113 | 1674.8040 | 1674.8651 | -36.45 | 1 | - | 15 | 0 | --- | -.MAYSLISSSTFLLSR.S | | 1776.0139 | 1775.0066 | 1775.0669 | -33.95 | 213 | - | 229 | 0 | --- | K.LRPIPIGVSADLLVGQK.V | | 1776.0139 | 1775.0066 | 1775.0669 | -33.95 | 213 | - | 229 | 0 | 110 | K.LRPIPIGVSADLLVGQK.V | | 1986.9907 | 1985.9834 | 1986.0534 | -35.24 | 121 | - | 138 | 0 | --- | R.LFQENTPSVVNITNLAAR.Q | | 1986.9907 | 1985.9834 | 1986.0534 | -35.24 | 121 | - | 138 | 0 | 139 | R.LFQENTPSVVNITNLAAR.Q | | 2401.2051 | 2400.1978 | 2400.2802 | -34.31 | 230 | - | 252 | 0 | --- | K.VYAIGNPFGLDHTLTTGVISGLR.R | | 2401.2051 | 2400.1978 | 2400.2802 | -34.31 | 230 | - | 252 | 0 | 95 | K.VYAIGNPFGLDHTLTTGVISGLR.R |  | **No match to:** 700.3634, 702.4082, 768.5113, 807.3590, 807.3590, 823.3680, 832.3029, 842.4819, 846.4556, 847.4457, 851.4266, 855.0226, 856.4766, 868.5295, 876.4941, 882.5372, 895.3973, 914.4102, 956.4505, 992.4467, 996.4433, 1008.4313, 1034.4965, 1045.5585, 1051.6746, 1114.6064, 1121.5433, 1128.5118, 1182.6167, 1183.6274, 1199.6075, 1200.6218, 1200.6218, 1214.6375, 1218.6244, 1223.5591, 1237.5851, 1241.6730, 1255.5541, 1256.5555, 1272.6260, 1272.6260, 1312.6757, 1315.6276, 1324.6101, 1326.6519, 1326.6519, 1334.7031, 1340.6533, 1342.6941, 1348.6581, 1356.6260, 1374.6677, 1383.6453, 1384.6467, 1386.6454, 1418.6737, 1431.6682, 1434.7250, 1439.7986, 1439.7986, 1453.8226, 1463.6656, 1463.6656, 1477.6904, 1480.7860, 1507.7745, 1573.7832, 1639.8054, 1678.7925, 1680.8276, 1703.7902, 1736.8348, 1800.8455, 1808.8174, 1808.8174, 1822.8485, 1835.8535, 1882.9052, 1922.9630, 1951.8699, 1954.8978, 1955.8772, 1967.8528, 2054.9561, 2076.9084, 2089.1023, 2107.9626, 2194.0205, 2198.0276, 2210.0171, 2246.1433, 2383.2039, 2673.1736, 2673.1736, 2676.0720, 2737.1660, 2737.1660 | | | --- | --- | | **S13.** | [Cs3g27720.1](http://localhost/mascot/cgi/protein_view.pl?file=../data/20140415/F003318.dat&hit=1)    **Mass:** 29900    **Score:** 366    **Expect:** 1.1e-032  **Matches:** 9 |  | **Observed** | **Mr(expt)** | **Mr(calc)** | **ppm** | **Start** |  | **End** | **Miss** | **Ions** | **Peptide** | | --- | --- | --- | --- | --- | --- | --- | --- | --- | --- | | 795.4247 | 794.4175 | 794.4763 | -74.00 | 186 | - | 193 | 0 | --- | R.RPPGVAAK.L | | 1409.6323 | 1408.6250 | 1408.6219 | 2.24 | 64 | - | 74 | 1 | --- | R.FRIEEQDDDSR.T | | 1492.6643 | 1491.6570 | 1491.7569 | -66.97 | 169 | - | 182 | 0 | --- | K.VEAFADTLVSGLDR.S | | 1492.6643 | 1491.6570 | 1491.7569 | -66.97 | 169 | - | 182 | 0 | 108 | K.VEAFADTLVSGLDR.S | | 1758.7332 | 1757.7259 | 1757.8360 | -62.59 | 235 | - | 249 | 0 | --- | R.LYTVTGQFVEEESEK.Y | | 1936.7838 | 1935.7765 | 1935.9003 | -63.92 | 202 | - | 217 | 0 | --- | K.GFYYIEYTLQNPGESR.K | | 1936.7838 | 1935.7765 | 1935.9003 | -63.92 | 202 | - | 217 | 0 | 101 | K.GFYYIEYTLQNPGESR.K | | 2916.2788 | 2915.2715 | 2915.4553 | -63.03 | 135 | - | 162 | 0 | --- | K.SITAFYPQEASSSSVSVVITGLGPDFTR.M | | 2916.2788 | 2915.2715 | 2915.4553 | -63.03 | 135 | - | 162 | 0 | 126 | K.SITAFYPQEASSSSVSVVITGLGPDFTR.M |  | **No match to:** 712.1978, 717.3118, 728.5100, 768.4822, 772.5266, 816.5491, 842.4513, 860.5738, 860.5738, 868.4955, 904.5971, 904.5971, 948.6177, 964.5159, 992.6466, 1013.4714, 1036.6653, 1080.6978, 1091.4388, 1098.5105, 1098.5105, 1127.5045, 1173.5443, 1173.5443, 1209.5635, 1226.5588, 1226.5588, 1240.5791, 1248.5342, 1252.5658, 1252.5658, 1273.4989, 1273.4989, 1277.5743, 1356.5852, 1378.6700, 1378.6700, 1382.7039, 1389.6685, 1390.6743, 1402.6976, 1411.6257, 1416.6281, 1506.6910, 1537.7346, 1566.6943, 1567.7155, 1568.7318, 1569.7236, 1616.7109, 1620.7437, 1634.7649, 1734.7531, 1734.7531, 1760.7677, 1760.7677, 1801.7921, 1808.7706, 1843.7445, 1900.8845, 1939.2229, 1950.7938, 1950.7938, 2018.9277, 2018.9277, 2022.8945, 2065.8843, 2424.9814 | | --- |  | **S2.** | [Cs6g11900.1](http://localhost/mascot/cgi/protein_view.pl?file=../data/20140808/F003834.dat&hit=1)    **Mass:** 30355    **Score:** 418    **Expect:** 7e-038  **Matches:** 13 | | --- | --- |  | **Observed** | **Mr(expt)** | **Mr(calc)** | **ppm** | **Start** |  | **End** | **Miss** | **Ions** | **Peptide** | | --- | --- | --- | --- | --- | --- | --- | --- | --- | --- | | 764.3447 | 763.3374 | 763.3501 | -16.58 | 177 | - | 182 | 0 | --- | R.EEFSPR.G | | 920.4544 | 919.4471 | 919.4512 | -4.38 | 176 | - | 182 | 1 | --- | R.REEFSPR.G | | 920.4544 | 919.4471 | 919.4876 | -43.97 | 167 | - | 175 | 0 | 68 | R.VNSGPPPPR.R | | 1001.5317 | 1000.5244 | 1000.5553 | -30.84 | 219 | - | 227 | 1 | --- | R.EQGKVLEAK.V | | 1044.4634 | 1043.4561 | 1043.4744 | -17.55 | 186 | - | 198 | 0 | --- | R.GGGAGAPSSGGNR.V | | 1044.4634 | 1043.4561 | 1043.4744 | -17.55 | 186 | - | 198 | 0 | 69 | R.GGGAGAPSSGGNR.V | | 1076.5724 | 1075.5651 | 1075.5887 | -21.89 | 167 | - | 176 | 1 | --- | R.VNSGPPPPRR.E | | 1076.5724 | 1075.5651 | 1075.5887 | -21.89 | 167 | - | 176 | 1 | 11 | R.VNSGPPPPRR.E | | 1112.5958 | 1111.5885 | 1111.6098 | -19.13 | 270 | - | 279 | 0 | --- | R.VSVAEARPQR.R | | 1112.5958 | 1111.5885 | 1111.6098 | -19.13 | 270 | - | 279 | 0 | 38 | R.VSVAEARPQR.R | | 2267.1077 | 2266.1004 | 2266.1634 | -27.79 | 199 | - | 218 | 0 | --- | R.VYVGNLSWGVDDLALETLFR.E | | 2903.2842 | 2902.2769 | 2902.3621 | -29.35 | 239 | - | 266 | 0 | --- | R.GFGFVTYSSAEEVDNAIDSLNGVDLAGR.A | | 2903.2842 | 2902.2769 | 2902.3621 | -29.35 | 239 | - | 266 | 0 | 195 | R.GFGFVTYSSAEEVDNAIDSLNGVDLAGR.A |  | **No match to:** 700.3907, 707.3644, 709.3474, 713.4066, 715.4048, 724.4979, 728.4326, 731.3942, 734.4576, 741.3987, 743.3839, 745.4121, 746.3453, 760.3557, 763.3324, 768.5176, 778.3563, 834.4822, 842.4882, 848.4489, 850.5083, 856.4849, 864.4634, 868.5325, 882.5452, 903.3976, 917.4464, 918.4442, 919.4346, 928.4657, 932.4946, 934.4592, 934.4592, 940.5352, 942.4473, 944.4484, 958.4499, 962.5403, 968.5490, 973.5132, 982.5465, 1004.4998, 1051.6808, 1055.5192, 1073.5796, 1129.6401, 1151.6984, 1216.5668, 1220.5662, 1232.5637, 1248.5652, 1252.7119, 1253.6270, 1265.6609, 1291.6561, 1322.6799, 1330.7085, 1330.7085, 1348.6802, 1348.6802, 1356.6250, 1421.6992, 1434.7863, 1529.7694, 1587.8693, 1587.8693, 2090.9417, 2131.9570, 2131.9570, 2145.9785, 2214.0112, 2214.0112, 2228.0142, 2246.1487, 2253.0906, 2269.1106, 2270.0942, 2271.0972, 2271.0972, 2281.1008, 2282.0947, 2283.0977, 2283.0977, 2287.1113, 2299.0938, 2299.0938, 2323.0977, 2725.1560, 2885.2878, 2885.2878, 2886.2900, 2900.3140, 2902.3179, 2917.2988, 3004.3926, 3005.2874, 3014.2646, 3018.3420, 3068.3218, 3069.3240, 3083.3513, 3435.5974 | | --- |  | **S32.** | [Cs7g01430.1](http://localhost/mascot/cgi/protein_view.pl?file=../data/20140415/F003341.dat&hit=1)    **Mass:** 27553    **Score:** 392    **Expect:** 2.8e-035  **Matches:** 19 | | --- | --- |  | **Observed** | **Mr(expt)** | **Mr(calc)** | **ppm** | **Start** |  | **End** | **Miss** | **Ions** | **Peptide** | | --- | --- | --- | --- | --- | --- | --- | --- | --- | --- | | 839.4228 | 838.4155 | 838.4046 | 13.1 | 136 | - | 142 | 0 | --- | K.QGNDHLR.Q | | 883.4394 | 882.4321 | 882.4195 | 14.3 | 123 | - | 130 | 0 | --- | K.AEPPQEGR.L | | 917.5276 | 916.5204 | 916.4623 | 63.4 | 31 | - | 38 | 0 | --- | K.NCAPLMLR.I | | 917.5276 | 916.5204 | 916.4623 | 63.4 | 31 | - | 38 | 0 | 11 | K.NCAPLMLR.I | | 923.4642 | 922.4570 | 922.4331 | 25.9 | 53 | - | 61 | 0 | --- | K.TGGPFGTMR.L | | 939.4473 | 938.4401 | 938.4280 | 12.8 | 53 | - | 61 | 0 | --- | K.TGGPFGTMR.L + Oxidation (M) | | 939.4473 | 938.4401 | 938.4280 | 12.8 | 53 | - | 61 | 0 | --- | K.TGGPFGTMR.L + Oxidation (M) | | 1036.4951 | 1035.4878 | 1035.4774 | 10.1 | 173 | - | 181 | 0 | --- | R.SGFEGPWTR.N | | 1036.4951 | 1035.4878 | 1035.4774 | 10.1 | 173 | - | 181 | 0 | 30 | R.SGFEGPWTR.N | | 1152.5018 | 1151.4945 | 1151.5757 | -70.53 | 51 | - | 61 | 1 | --- | K.TKTGGPFGTMR.L | | 1241.6022 | 1240.5949 | 1240.5684 | 21.4 | 120 | - | 130 | 1 | --- | R.DDKAEPPQEGR.L | | 1295.7257 | 1294.7184 | 1294.6994 | 14.7 | 155 | - | 167 | 0 | --- | K.DIVALSGGHTLGR.C | | 1611.9336 | 1610.9263 | 1610.9032 | 14.4 | 210 | - | 223 | 0 | --- | K.ALLDDPVFRPLVEK.Y | | 1611.9336 | 1610.9263 | 1610.9032 | 14.4 | 210 | - | 223 | 0 | 53 | K.ALLDDPVFRPLVEK.Y | | 1849.9890 | 1848.9817 | 1848.9442 | 20.3 | 62 | - | 79 | 0 | --- | R.LAAEQAHSANNGLDIAVR.L | | 1849.9890 | 1848.9817 | 1848.9442 | 20.3 | 62 | - | 79 | 0 | 114 | R.LAAEQAHSANNGLDIAVR.L | | 2046.9486 | 2045.9413 | 2045.9007 | 19.9 | 224 | - | 241 | 0 | --- | K.YAADEDAFFADYAEAHLK.L | | 2573.3945 | 2572.3872 | 2572.3068 | 31.3 | 143 | - | 167 | 1 | --- | R.QVFGAQMGLSDKDIVALSGGHTLGR.C + Oxidation (M) | | 2573.3945 | 2572.3872 | 2572.3068 | 31.3 | 143 | - | 167 | 1 | 100 | R.QVFGAQMGLSDKDIVALSGGHTLGR.C + Oxidation (M) |  | **No match to:** 728.5670, 772.5941, 816.6177, 822.3975, 860.6493, 861.4143, 875.4606, 904.6840, 926.5024, 932.7064, 948.6949, 951.5219, 974.4963, 979.5692, 990.5053, 990.5053, 992.7404, 1006.4941, 1034.4958, 1040.4901, 1045.5857, 1050.4901, 1051.4943, 1052.4941, 1052.4941, 1066.5062, 1068.4921, 1068.4921, 1084.6709, 1129.6078, 1160.6617, 1187.5386, 1206.5350, 1251.5481, 1255.7107, 1293.6799, 1361.7227, 1413.6489, 1414.7144, 1414.7144, 1569.7632, 1594.9121, 1619.8688, 1625.9460, 1625.9460, 1634.9319, 1832.9694, 1922.0723, 1922.0723, 1938.0497, 2057.0613, 2057.0613, 2073.0977, 2267.2341, 2267.2341, 2281.2461, 2492.1675, 2492.4092, 2493.3535, 2508.3708, 2509.3188, 2556.3779, 2556.3779 | | --- |  | **S17.** | [Cs1g23450.1](http://localhost/mascot/cgi/protein_view.pl?file=../data/20140415/F003328.dat&hit=1)    **Mass:** 35355    **Score:** 261    **Expect:** 3.5e-022  **Matches:** 7 | | --- | --- |  | **Observed** | **Mr(expt)** | **Mr(calc)** | **ppm** | **Start** |  | **End** | **Miss** | **Ions** | **Peptide** | | --- | --- | --- | --- | --- | --- | --- | --- | --- | --- | | 964.5743 | 963.5670 | 963.5793 | -12.81 | 210 | - | 217 | 0 | --- | R.VPFLFTIK.Q | | 1252.6566 | 1251.6493 | 1251.6459 | 2.74 | 92 | - | 101 | 1 | --- | K.RLTYDEIQSK.T | | 1252.6566 | 1251.6493 | 1251.6459 | 2.74 | 92 | - | 101 | 1 | 21 | K.RLTYDEIQSK.T | | 1760.8698 | 1759.8625 | 1759.8741 | -6.57 | 193 | - | 209 | 0 | --- | K.DGIDYAAVTVQLPGGER.V | | 1760.8698 | 1759.8625 | 1759.8741 | -6.57 | 193 | - | 209 | 0 | 116 | K.DGIDYAAVTVQLPGGER.V | | 2295.1660 | 2294.1587 | 2294.1695 | -4.71 | 218 | - | 238 | 0 | --- | K.QLVASGKPENFGGEFLVPSYR.G | | 2295.1660 | 2294.1587 | 2294.1695 | -4.71 | 218 | - | 238 | 0 | 99 | K.QLVASGKPENFGGEFLVPSYR.G |  | **No match to:** 926.4923, 926.4923, 1050.5992, 1071.5977, 1104.5767, 1104.5767, 1121.6108, 1121.6108, 1189.6846, 1189.6846, 1234.6830, 1234.6830, 1305.7223, 1305.7223, 1611.7697, 1625.7970, 1625.7970, 1734.8528, 1734.8528, 1806.9432, 1806.9432, 2027.9795, 2027.9795, 2278.1382, 2278.1382, 2309.1514, 2398.2261, 2398.2261, 2425.1523 | | --- |  | **S3.** | [Cs2g28060.4](http://localhost/mascot/cgi/protein_view.pl?file=../data/20140808/F003835.dat&hit=1)    **Mass:** 35306    **Score:** 171    **Expect:** 3.5e-013  **Matches:** 5 | | --- | --- |  | **Observed** | **Mr(expt)** | **Mr(calc)** | **ppm** | **Start** |  | **End** | **Miss** | **Ions** | **Peptide** | | --- | --- | --- | --- | --- | --- | --- | --- | --- | --- | | 823.3745 | 822.3672 | 822.3872 | -24.32 | 111 | - | 117 | 0 | --- | K.AFDSVER.I | | 1251.4818 | 1250.4745 | 1250.6983 | -178.89 | 35 | - | 46 | 0 | --- | R.LNSPASPPSLIR.N | | 1660.8617 | 1659.8544 | 1659.8984 | -26.51 | 189 | - | 204 | 0 | --- | K.YAGVGAAVEYAVLHLK.V | | 1921.9753 | 1920.9680 | 1921.0309 | -32.73 | 269 | - | 285 | 0 | --- | K.EAVNVSLSNLLTYPFVR.E | | 1921.9753 | 1920.9680 | 1921.0309 | -32.73 | 269 | - | 285 | 0 | 162 | K.EAVNVSLSNLLTYPFVR.E |  | **No match to:** 703.3970, 712.2419, 717.4117, 804.2778, 812.3989, 826.4159, 829.3539, 832.2902, 832.2902, 842.4894, 847.4448, 856.4832, 955.4412, 955.4412, 996.4960, 1034.5269, 1128.5066, 1132.5125, 1132.5125, 1136.5177, 1146.5371, 1148.5146, 1164.5167, 1182.6193, 1183.6281, 1183.6281, 1189.5397, 1195.6377, 1197.6512, 1197.6512, 1200.6421, 1205.6118, 1209.6487, 1211.6591, 1214.5935, 1223.5707, 1227.6364, 1235.4951, 1256.6459, 1272.6299, 1312.5850, 1320.6948, 1321.6616, 1321.6616, 1323.6438, 1336.6749, 1337.6672, 1352.6399, 1353.6400, 1368.6608, 1370.6381, 1370.6381, 1381.6505, 1384.6589, 1386.6294, 1386.6294, 1400.6539, 1402.6486, 1424.6954, 1425.7500, 1439.7819, 1482.7766, 1574.8021, 1610.8372, 1636.8547, 1636.8547, 1639.8293, 1650.8651, 1664.8341, 1667.8193, 1687.8011, 1703.7990, 1735.8877, 1736.8400, 1773.8113, 1776.0253, 1784.8492, 1800.8489, 1800.8489, 1826.8761, 1830.8655, 1834.8833, 1842.8700, 1846.8671, 1847.8867, 1861.9287, 1875.9462, 1903.9744, 1903.9744, 1976.0078, 1987.0100, 1990.0209, 1990.0209, 2056.9634, 2056.9634, 2070.9873, 2072.9834, 2146.0647, 2160.0933, 2194.0471, 2210.0830, 2224.0884, 2238.1121, 2283.9521, 2288.1519, 2336.1616, 2338.1707, 2352.1885, 2352.1885, 2401.2310 | | --- |  | **S11.** | [Cs8g16040.1](http://localhost/mascot/cgi/protein_view.pl?file=../data/20140808/F003842.dat&hit=1)    **Mass:** 61464    **Score:** 1250   **Expect:** 4.4e-121  **Matches:** 39 | | --- | --- |  | **Observed** | **Mr(expt)** | **Mr(calc)** | **ppm** | **Start** |  | **End** | **Miss** | **Ions** | **Peptide** | | --- | --- | --- | --- | --- | --- | --- | --- | --- | --- | | 731.3588 | 730.3516 | 730.3609 | -12.86 | 380 | - | 385 | 0 | --- | K.DEIQAR.I | | 733.3446 | 732.3373 | 732.3555 | -24.80 | 318 | - | 324 | 0 | --- | K.APGFGER.R | | 798.4404 | 797.4332 | 797.4508 | -22.08 | 175 | - | 181 | 0 | --- | R.ARPIEGR.D | | 802.4501 | 801.4428 | 801.4596 | -21.00 | 260 | - | 266 | 0 | --- | R.VLVTDQK.I | | 898.5496 | 897.5423 | 897.5647 | -25.01 | 309 | - | 317 | 0 | --- | R.GILNVAAIK.A | | 898.5496 | 897.5423 | 897.5647 | -25.01 | 309 | - | 317 | 0 | 54 | R.GILNVAAIK.A | | 940.5494 | 939.5421 | 939.5641 | -23.36 | 272 | - | 279 | 0 | --- | K.DIIPLLEK.T | | 1043.5598 | 1042.5525 | 1042.5771 | -23.59 | 89 | - | 98 | 0 | --- | K.VVNDGVTIAR.A | | 1043.5598 | 1042.5525 | 1042.5771 | -23.59 | 89 | - | 98 | 0 | 53 | K.VVNDGVTIAR.A | | 1064.5128 | 1063.5055 | 1063.5298 | -22.84 | 46 | - | 54 | 0 | --- | K.DIAFDQISR.A | | 1064.5128 | 1063.5055 | 1063.5298 | -22.84 | 46 | - | 54 | 0 | 62 | K.DIAFDQISR.A | | 1090.5494 | 1089.5421 | 1089.5818 | -36.44 | 251 | - | 259 | 0 | --- | K.LIVEFENAR.V | | 1090.5494 | 1089.5421 | 1089.5818 | -36.44 | 251 | - | 259 | 0 | 79 | K.LIVEFENAR.V | | 1196.6249 | 1195.6176 | 1195.6271 | -7.93 | 571 | - | 582 | 0 | --- | K.TPVAAPPQGLMV.- + Oxidation (M) | | 1198.6503 | 1197.6430 | 1197.6717 | -23.97 | 64 | - | 75 | 0 | --- | K.LSDAVGLTLGPR.G | | 1198.6503 | 1197.6430 | 1197.6717 | -23.97 | 64 | - | 75 | 0 | 99 | K.LSDAVGLTLGPR.G | | 1204.5947 | 1203.5874 | 1203.6136 | -21.71 | 78 | - | 88 | 0 | --- | R.NVVLDEFGSPK.V | | 1290.5903 | 1289.5830 | 1289.6099 | -20.85 | 421 | - | 432 | 0 | --- | K.VGAATETELEDR.K | | 1290.5903 | 1289.5830 | 1289.6099 | -20.85 | 421 | - | 432 | 0 | 69 | K.VGAATETELEDR.K | | 1409.7355 | 1408.7282 | 1408.7674 | -27.82 | 163 | - | 174 | 1 | --- | K.TVHGLVEELEKR.A | | 1409.7355 | 1408.7282 | 1408.7674 | -27.82 | 163 | - | 174 | 1 | 77 | K.TVHGLVEELEKR.A | | 1418.6914 | 1417.6841 | 1417.7049 | -14.63 | 421 | - | 433 | 1 | --- | K.VGAATETELEDRK.L | | 1479.7104 | 1478.7031 | 1478.7405 | -25.30 | 238 | - | 250 | 0 | --- | R.GYISPQFVTNPEK.L | | 1479.7104 | 1478.7031 | 1478.7405 | -25.30 | 238 | - | 250 | 0 | 86 | R.GYISPQFVTNPEK.L | | 1485.6376 | 1484.6303 | 1484.6518 | -14.48 | 392 | - | 404 | 0 | --- | K.ELAETDSVYDSEK.L | | 1555.8649 | 1554.8576 | 1554.8981 | -26.03 | 142 | - | 157 | 0 | --- | K.LGLLSVTSGANPVSLK.R | | 1564.7576 | 1563.7503 | 1563.7603 | -6.36 | 527 | - | 540 | 0 | --- | K.YENMLQAGVIDPAK.V + Oxidation (M) | | 1636.7406 | 1635.7333 | 1635.7700 | -22.43 | 121 | - | 137 | 0 | --- | K.TNDSAGDGTTTASVLAR.E | | 1636.7406 | 1635.7333 | 1635.7700 | -22.43 | 121 | - | 137 | 0 | 119 | K.TNDSAGDGTTTASVLAR.E | | 1754.8644 | 1753.8571 | 1753.9032 | -26.28 | 99 | - | 115 | 0 | --- | R.AIELADPMENAGAALIR.E | | 1770.8667 | 1769.8594 | 1769.8981 | -21.87 | 99 | - | 115 | 0 | --- | R.AIELADPMENAGAALIR.E + Oxidation (M) | | 1770.8667 | 1769.8594 | 1769.8981 | -21.87 | 99 | - | 115 | 0 | 87 | R.AIELADPMENAGAALIR.E + Oxidation (M) | | 1904.9053 | 1903.8980 | 1903.9487 | -26.60 | 368 | - | 385 | 1 | --- | K.DSTTIIADAASKDEIQAR.I | | 1904.9053 | 1903.8980 | 1903.9487 | -26.60 | 368 | - | 385 | 1 | 182 | K.DSTTIIADAASKDEIQAR.I | | 2273.2026 | 2272.1953 | 2272.2427 | -20.84 | 488 | - | 510 | 0 | --- | K.ALVAPASLIAHNAGVEGEVVVEK.V | | 2273.2026 | 2272.1953 | 2272.2427 | -20.84 | 488 | - | 510 | 0 | 139 | K.ALVAPASLIAHNAGVEGEVVVEK.V | | 2291.1177 | 2290.1104 | 2290.1362 | -11.27 | 186 | - | 208 | 0 | --- | K.AVATISAGNDDLIGTMIADAIDK.V + Oxidation (M) | | 2954.5068 | 2953.4995 | 2953.5661 | -22.56 | 441 | - | 470 | 0 | --- | K.NATFAAIEEGIVPGGGAALVHLSDHVPAIK.D | | 3114.3723 | 3113.3650 | 3113.4234 | -18.76 | 209 | - | 237 | 0 | --- | K.VGPDGVLSIESSSSFETTVEVEEGMEIDR.G + Oxidation (M) |  | **No match to:** 713.4145, 724.3608, 745.3773, 747.3649, 768.5173, 771.4540, 797.4171, 812.4584, 827.5137, 832.2955, 842.4877, 850.5094, 855.0293, 864.4032, 864.4032, 868.5317, 882.5371, 883.5235, 949.4781, 1019.5421, 1051.6737, 1055.5131, 1072.5654, 1073.5476, 1088.5488, 1089.5400, 1104.5732, 1104.5732, 1112.5442, 1118.5769, 1126.5562, 1128.5160, 1132.5989, 1158.5707, 1177.6395, 1218.6088, 1233.6046, 1252.6777, 1255.6555, 1267.6670, 1270.6692, 1280.7319, 1289.6418, 1299.6498, 1304.6082, 1349.6165, 1353.6178, 1356.6140, 1394.6106, 1398.6072, 1423.7535, 1437.7339, 1465.7135, 1467.6597, 1493.7297, 1499.7117, 1501.6925, 1517.6760, 1535.7456, 1550.7705, 1635.7858, 1700.8442, 1706.8594, 1720.8756, 1784.8784, 1789.8634, 1869.9226, 1882.9695, 1902.9192, 1993.9846, 2015.9794, 2033.0001, 2033.0001, 2227.0945, 2287.2119, 3050.3806 | | --- |  | **S9.** | [Cs7g31800.3](http://localhost/mascot/cgi/protein_view.pl?file=../data/20140415/F003312.dat&hit=1)    **Mass:** 46932    **Score:** 505    **Expect:** 1.4e-039  **Matches:** 21 | | --- | --- |  | **Observed** | **Mr(expt)** | **Mr(calc)** | **ppm** | **Start** |  | **End** | **Miss** | **Ions** | **Peptide** | | --- | --- | --- | --- | --- | --- | --- | --- | --- | --- | | 895.3723 | 894.3650 | 894.4083 | -48.45 | 343 | - | 349 | 0 | --- | R.VYDDEVR.K | | 895.3723 | 894.3650 | 894.4083 | -48.45 | 343 | - | 349 | 0 | 35 | R.VYDDEVR.K | | 940.4207 | 939.4134 | 939.4603 | -49.86 | 294 | - | 300 | 0 | --- | K.FYWAPTR.E | | 1145.5073 | 1144.5000 | 1144.5513 | -44.77 | 369 | - | 378 | 0 | --- | K.EAAPTFEQPR.M | | 1145.5073 | 1144.5000 | 1144.5513 | -44.77 | 369 | - | 378 | 0 | 48 | K.EAAPTFEQPR.M | | 1159.5098 | 1158.5025 | 1158.6397 | -118.42 | 351 | - | 362 | 0 | --- | K.WISGVGVGSIGK.S | | 1159.5098 | 1158.5025 | 1158.6397 | -118.42 | 351 | - | 362 | 0 | --- | K.WISGVGVGSIGK.S | | 1725.6945 | 1724.6872 | 1724.7489 | -35.78 | 64 | - | 78 | 0 | --- | K.GLAYDESDDQQDITR.G | | 1725.6945 | 1724.6872 | 1724.7489 | -35.78 | 64 | - | 78 | 0 | 107 | K.GLAYDESDDQQDITR.G | | 1882.8937 | 1881.8864 | 1881.9625 | -40.44 | 324 | - | 340 | 0 | --- | K.LVDTFPGQSIDFFGALR.A | | 1882.8937 | 1881.8864 | 1881.9625 | -40.44 | 324 | - | 340 | 0 | 103 | K.LVDTFPGQSIDFFGALR.A | | 2078.9780 | 2077.9707 | 2078.0466 | -36.51 | 384 | - | 400 | 1 | --- | K.LLEYGNMIVQEQENVKR.V + Oxidation (M) | | 2089.0874 | 2088.0801 | 2088.1619 | -39.17 | 269 | - | 287 | 0 | --- | R.VPIIVTGNDFSTLYAPLIR.D | | 2089.0874 | 2088.0801 | 2088.1619 | -39.17 | 269 | - | 287 | 0 | 100 | R.VPIIVTGNDFSTLYAPLIR.D |  | **No match to:** 816.5691, 944.4177, 944.4177, 956.4172, 956.4172, 972.4091, 972.4091, 1110.4518, 1110.4518, 1127.4988, 1127.4988, 1156.6448, 1228.5554, 1288.5725, 1332.7202, 1332.7202, 1885.3058, 2014.9525, 2014.9525, 2769.1533, 2769.1533, 2832.1953, 2832.1953 | | --- |  | **S14.** | [Cs7g31800.3](http://localhost/mascot/cgi/protein_view.pl?file=../data/20140415/F003322.dat&hit=1)    **Mass:** 46932    **Score:** 505    **Expect:** 1.4e-046  **Matches:** 21 | | --- | --- |  | **Observed** | **Mr(expt)** | **Mr(calc)** | **ppm** | **Start** |  | **End** | **Miss** | **Ions** | **Peptide** | | --- | --- | --- | --- | --- | --- | --- | --- | --- | --- | | 895.3399 | 894.3326 | 894.4083 | -84.62 | 343 | - | 349 | 0 | --- | R.VYDDEVR.K | | 895.3399 | 894.3326 | 894.4083 | -84.62 | 343 | - | 349 | 0 | 35 | R.VYDDEVR.K | | 940.3896 | 939.3823 | 939.4603 | -82.99 | 294 | - | 300 | 0 | --- | K.FYWAPTR.E | | 940.3896 | 939.3823 | 939.4603 | -82.99 | 294 | - | 300 | 0 | 24 | K.FYWAPTR.E | | 992.6321 | 991.6249 | 991.5637 | 61.7 | 304 | - | 312 | 1 | --- | R.IGVCKGIFR.N | | 1023.4255 | 1022.4182 | 1022.5033 | -83.17 | 343 | - | 350 | 1 | --- | R.VYDDEVRK.W | | 1145.4642 | 1144.4569 | 1144.5513 | -82.43 | 369 | - | 378 | 0 | --- | K.EAAPTFEQPR.M | | 1145.4642 | 1144.4569 | 1144.5513 | -82.43 | 369 | - | 378 | 0 | 42 | K.EAAPTFEQPR.M | | 1159.4952 | 1158.4879 | 1158.6397 | -131.02 | 351 | - | 362 | 0 | --- | K.WISGVGVGSIGK.S | | 1159.4952 | 1158.4879 | 1158.6397 | -131.02 | 351 | - | 362 | 0 | 2 | K.WISGVGVGSIGK.S | | 1328.5616 | 1327.5543 | 1327.6383 | -63.26 | 291 | - | 300 | 1 | --- | R.MEKFYWAPTR.E | | 1340.5490 | 1339.5417 | 1339.6309 | -66.58 | 294 | - | 303 | 1 | --- | K.FYWAPTREDR.I | | 1725.6373 | 1724.6300 | 1724.7489 | -68.94 | 64 | - | 78 | 0 | --- | K.GLAYDESDDQQDITR.G | | 1725.6373 | 1724.6300 | 1724.7489 | -68.94 | 64 | - | 78 | 0 | 86 | K.GLAYDESDDQQDITR.G | | 1882.8215 | 1881.8142 | 1881.9625 | -78.80 | 324 | - | 340 | 0 | --- | K.LVDTFPGQSIDFFGALR.A | | 1882.8215 | 1881.8142 | 1881.9625 | -78.80 | 324 | - | 340 | 0 | 100 | K.LVDTFPGQSIDFFGALR.A | | 2062.9285 | 2061.9212 | 2062.0517 | -63.27 | 384 | - | 400 | 1 | --- | K.LLEYGNMIVQEQENVKR.V | | 2062.9285 | 2061.9212 | 2062.0517 | -63.27 | 384 | - | 400 | 1 | 64 | K.LLEYGNMIVQEQENVKR.V | | 2078.9255 | 2077.9182 | 2078.0466 | -61.78 | 384 | - | 400 | 1 | --- | K.LLEYGNMIVQEQENVKR.V + Oxidation (M) | | 2089.0137 | 2088.0064 | 2088.1619 | -74.47 | 269 | - | 287 | 0 | --- | R.VPIIVTGNDFSTLYAPLIR.D | | 2089.0137 | 2088.0064 | 2088.1619 | -74.47 | 269 | - | 287 | 0 | 107 | R.VPIIVTGNDFSTLYAPLIR.D |  | **No match to:** 710.3475, 712.1921, 728.4952, 772.5117, 797.3237, 816.5348, 832.2357, 860.4272, 860.5590, 904.5714, 944.3863, 948.6092, 956.3805, 956.3805, 968.4021, 972.3792, 972.3792, 1036.6417, 1110.4067, 1127.4613, 1127.4613, 1149.4751, 1218.5476, 1228.5068, 1288.5222, 1288.5222, 1324.5280, 1332.6710, 1446.6027, 1536.6063, 1553.6357, 1553.6357, 1556.6582, 1680.7260, 1864.8059, 1864.8059, 1866.8180, 1880.8700, 1887.2839, 1896.8203, 1898.8260, 1904.8138, 2014.9153, 2014.9153, 2045.9316, 2071.0066, 2075.0059, 2087.9817, 2092.4673, 2095.9626, 2102.9956, 2106.0259, 3226.5562 | | --- |  | **S4.** | [Cs7g31800.3](http://localhost/mascot/cgi/protein_view.pl?file=../data/20140808/F003836.dat&hit=1)    **Mass:** 46932    **Score:** 579    **Expect:** 5.6e-054  **Matches:** 19 | | --- | --- |  | **Observed** | **Mr(expt)** | **Mr(calc)** | **ppm** | **Start** |  | **End** | **Miss** | **Ions** | **Peptide** | | --- | --- | --- | --- | --- | --- | --- | --- | --- | --- | | 895.3875 | 894.3802 | 894.4083 | -31.39 | 343 | - | 349 | 0 | --- | R.VYDDEVR.K | | 895.3875 | 894.3802 | 894.4083 | -31.39 | 343 | - | 349 | 0 | 57 | R.VYDDEVR.K | | 940.4424 | 939.4352 | 939.4603 | -26.74 | 294 | - | 300 | 0 | --- | K.FYWAPTR.E | | 940.4424 | 939.4352 | 939.4603 | -26.74 | 294 | - | 300 | 0 | 28 | K.FYWAPTR.E | | 1079.5291 | 1078.5218 | 1078.5481 | -24.36 | 139 | - | 147 | 0 | --- | K.NFMSLPNIK.V + Oxidation (M) | | 1145.5225 | 1144.5152 | 1144.5513 | -31.49 | 369 | - | 378 | 0 | --- | K.EAAPTFEQPR.M | | 1145.5225 | 1144.5152 | 1144.5513 | -31.49 | 369 | - | 378 | 0 | 81 | K.EAAPTFEQPR.M | | 1159.5465 | 1158.5392 | 1158.6397 | -86.74 | 351 | - | 362 | 0 | --- | K.WISGVGVGSIGK.S | | 1287.6403 | 1286.6330 | 1286.7347 | -79.00 | 350 | - | 362 | 1 | --- | R.KWISGVGVGSIGK.S | | 1340.6053 | 1339.5980 | 1339.6309 | -24.56 | 294 | - | 303 | 1 | --- | K.FYWAPTREDR.I | | 1344.6125 | 1343.6052 | 1343.6332 | -20.84 | 291 | - | 300 | 1 | --- | R.MEKFYWAPTR.E + Oxidation (M) | | 1614.8398 | 1613.8325 | 1613.6670 | 103 | 213 | - | 227 | 0 | --- | K.MCCLMINDLDAGAGR.M + 2 Oxidation (M) | | 1725.7030 | 1724.6957 | 1724.7489 | -30.85 | 64 | - | 78 | 0 | --- | K.GLAYDESDDQQDITR.G | | 1725.7030 | 1724.6957 | 1724.7489 | -30.85 | 64 | - | 78 | 0 | 114 | K.GLAYDESDDQQDITR.G | | 1882.9023 | 1881.8950 | 1881.9625 | -35.87 | 324 | - | 340 | 0 | --- | K.LVDTFPGQSIDFFGALR.A | | 1882.9023 | 1881.8950 | 1881.9625 | -35.87 | 324 | - | 340 | 0 | 129 | K.LVDTFPGQSIDFFGALR.A | | 2078.9932 | 2077.9859 | 2078.0466 | -29.20 | 384 | - | 400 | 1 | --- | K.LLEYGNMIVQEQENVKR.V + Oxidation (M) | | 2089.0945 | 2088.0872 | 2088.1619 | -35.77 | 269 | - | 287 | 0 | --- | R.VPIIVTGNDFSTLYAPLIR.D | | 2089.0945 | 2088.0872 | 2088.1619 | -35.77 | 269 | - | 287 | 0 | 139 | R.VPIIVTGNDFSTLYAPLIR.D |  | **No match to:** 724.3839, 731.3279, 744.3842, 768.5116, 774.4236, 787.4122, 832.2917, 835.3560, 842.4836, 850.4730, 855.5176, 855.5176, 861.0460, 868.5280, 909.4049, 944.4379, 956.4338, 972.4402, 1015.5274, 1038.5304, 1060.5205, 1070.5558, 1090.5575, 1106.5743, 1119.5824, 1127.5190, 1127.5190, 1151.5089, 1156.6453, 1165.5272, 1167.5458, 1174.5659, 1191.5753, 1191.5753, 1218.5510, 1228.5675, 1233.5902, 1235.5875, 1236.5748, 1250.5709, 1255.5735, 1288.6061, 1320.6174, 1324.6183, 1356.6022, 1356.6022, 1372.6659, 1381.7020, 1387.6541, 1409.6705, 1444.6901, 1551.7072, 1566.7423, 1573.7336, 1592.7522, 1610.8396, 1620.8284, 1626.7904, 1630.7579, 1630.7579, 1642.7612, 1658.7808, 1664.7144, 1707.7128, 1728.6940, 1733.8835, 1736.8762, 1739.7249, 1750.9495, 1750.9495, 1772.8716, 1772.8716, 1786.8983, 1794.8601, 1796.8904, 1796.8904, 1801.8615, 1810.8412, 1826.9135, 1828.9192, 1828.9192, 1842.9237, 1864.8855, 1878.9078, 1890.9349, 1904.8954, 1953.8990, 1996.8698, 2014.9790, 2060.8672, 2071.0479, 2079.9900, 2141.9978, 2338.0737, 2358.0869, 3589.7363 | | --- |  | **S10.** | [Cs7g31800.3](http://localhost/mascot/cgi/protein_view.pl?file=../data/20140808/F003841.dat&hit=1)    **Mass:** 46932    **Score:** 641    **Expect:** 3.5e-060  **Matches:** 24 | | --- | --- |  | **Observed** | **Mr(expt)** | **Mr(calc)** | **ppm** | **Start** |  | **End** | **Miss** | **Ions** | **Peptide** | | --- | --- | --- | --- | --- | --- | --- | --- | --- | --- | | 809.3913 | 808.3840 | 808.5171 | -164.56 | 132 | - | 138 | 0 | --- | K.LVVHITK.N | | 809.4916 | 808.4844 | 808.5171 | -40.46 | 132 | - | 138 | 0 | --- | K.LVVHITK.N | | 895.3874 | 894.3801 | 894.4083 | -31.52 | 343 | - | 349 | 0 | --- | R.VYDDEVR.K | | 895.3874 | 894.3801 | 894.4083 | -31.52 | 343 | - | 349 | 0 | 57 | R.VYDDEVR.K | | 940.4414 | 939.4341 | 939.4603 | -27.83 | 294 | - | 300 | 0 | --- | K.FYWAPTR.E | | 940.4414 | 939.4341 | 939.4603 | -27.83 | 294 | - | 300 | 0 | 32 | K.FYWAPTR.E | | 970.4266 | 969.4194 | 969.5607 | -145.79 | 31 | - | 39 | 1 | --- | K.VSSRIPPSK.V | | 1079.5305 | 1078.5232 | 1078.5481 | -23.06 | 139 | - | 147 | 0 | --- | K.NFMSLPNIK.V + Oxidation (M) | | 1145.5237 | 1144.5164 | 1144.5513 | -30.45 | 369 | - | 378 | 0 | --- | K.EAAPTFEQPR.M | | 1145.5237 | 1144.5164 | 1144.5513 | -30.45 | 369 | - | 378 | 0 | 76 | K.EAAPTFEQPR.M | | 1152.5944 | 1151.5871 | 1151.7067 | -103.79 | 148 | - | 158 | 0 | --- | K.VPLILGIWGGK.G | | 1159.5417 | 1158.5344 | 1158.6397 | -90.88 | 351 | - | 362 | 0 | --- | K.WISGVGVGSIGK.S | | 1159.5417 | 1158.5344 | 1158.6397 | -90.88 | 351 | - | 362 | 0 | --- | K.WISGVGVGSIGK.S | | 1287.6476 | 1286.6403 | 1286.7347 | -73.33 | 350 | - | 362 | 1 | --- | R.KWISGVGVGSIGK.S | | 1340.6017 | 1339.5944 | 1339.6309 | -27.24 | 294 | - | 303 | 1 | --- | K.FYWAPTREDR.I | | 1344.6010 | 1343.5937 | 1343.6332 | -29.40 | 291 | - | 300 | 1 | --- | R.MEKFYWAPTR.E + Oxidation (M) | | 1725.7059 | 1724.6986 | 1724.7489 | -29.17 | 64 | - | 78 | 0 | --- | K.GLAYDESDDQQDITR.G | | 1725.7059 | 1724.6986 | 1724.7489 | -29.17 | 64 | - | 78 | 0 | 117 | K.GLAYDESDDQQDITR.G | | 1882.9072 | 1881.8999 | 1881.9625 | -33.26 | 324 | - | 340 | 0 | --- | K.LVDTFPGQSIDFFGALR.A | | 1882.9072 | 1881.8999 | 1881.9625 | -33.26 | 324 | - | 340 | 0 | 137 | K.LVDTFPGQSIDFFGALR.A | | 2062.9854 | 2061.9781 | 2062.0517 | -35.67 | 384 | - | 400 | 1 | --- | K.LLEYGNMIVQEQENVKR.V | | 2079.0027 | 2077.9954 | 2078.0466 | -24.63 | 384 | - | 400 | 1 | --- | K.LLEYGNMIVQEQENVKR.V + Oxidation (M) | | 2089.1006 | 2088.0933 | 2088.1619 | -32.85 | 269 | - | 287 | 0 | --- | R.VPIIVTGNDFSTLYAPLIR.D | | 2089.1006 | 2088.0933 | 2088.1619 | -32.85 | 269 | - | 287 | 0 | 175 | R.VPIIVTGNDFSTLYAPLIR.D |  | **No match to:** 710.3850, 724.3398, 769.4052, 786.3829, 787.4102, 832.2907, 842.4870, 868.5184, 909.4082, 909.4082, 938.4372, 944.4366, 954.4418, 955.4352, 956.4379, 956.4379, 968.4377, 972.4343, 995.4494, 996.4613, 997.4606, 1010.4664, 1015.5271, 1025.5007, 1048.4969, 1090.5482, 1092.5315, 1100.5011, 1101.5013, 1102.5095, 1106.5596, 1114.5417, 1118.5096, 1120.5247, 1127.5187, 1127.5187, 1131.5214, 1136.5228, 1141.5315, 1144.4995, 1153.5966, 1156.6758, 1167.5331, 1173.5430, 1178.6300, 1184.5671, 1191.5697, 1218.5488, 1228.5701, 1228.5701, 1242.5809, 1250.5585, 1288.5925, 1288.5925, 1303.6606, 1324.6281, 1332.7336, 1356.6127, 1381.7080, 1409.6959, 1444.7091, 1555.7192, 1566.7502, 1592.7683, 1664.6863, 1707.7251, 1708.7125, 1712.6813, 1728.6886, 1733.9209, 1739.7240, 1739.7240, 1828.9330, 1864.8976, 1864.8976, 1880.9094, 1881.8931, 1885.3137, 1897.8997, 1898.8959, 1904.8982, 1920.8674, 2015.0018, 2015.0018, 2064.0081, 2071.1035, 2088.1191, 2103.0928, 2111.0730, 2291.0432, 3291.4709 | | --- |  | **S21.** | [Cs3g08480.1](http://localhost/mascot/cgi/protein_view.pl?file=../data/20140808/F003847.dat&hit=1)    **Mass:** 45159    **Score:** 686    **Expect:** 1.1e-064  **Matches:** 31 | | --- | --- |  | **Observed** | **Mr(expt)** | **Mr(calc)** | **ppm** | **Start** |  | **End** | **Miss** | **Ions** | **Peptide** | | --- | --- | --- | --- | --- | --- | --- | --- | --- | --- | | 828.4352 | 827.4279 | 827.4501 | -26.85 | 128 | - | 135 | 0 | --- | K.GVTALDPR.A | | 870.4499 | 869.4427 | 869.4607 | -20.69 | 222 | - | 229 | 0 | --- | R.GHSLESIK.A | | 1085.5627 | 1084.5554 | 1084.5877 | -29.72 | 126 | - | 135 | 1 | --- | K.EKGVTALDPR.A | | 1273.6418 | 1272.6345 | 1272.6714 | -28.98 | 82 | - | 94 | 0 | --- | R.LTSVFGGAAEPPK.G | | 1343.6404 | 1342.6331 | 1342.6591 | -19.36 | 355 | - | 365 | 0 | --- | K.FYGEVTQQMLK.H | | 1359.6237 | 1358.6164 | 1358.6540 | -27.68 | 355 | - | 365 | 0 | --- | K.FYGEVTQQMLK.H + Oxidation (M) | | 1359.6237 | 1358.6164 | 1358.6540 | -27.68 | 355 | - | 365 | 0 | 38 | K.FYGEVTQQMLK.H + Oxidation (M) | | 1429.7332 | 1428.7259 | 1428.7725 | -32.60 | 81 | - | 94 | 1 | --- | R.RLTSVFGGAAEPPK.G | | 1429.7332 | 1428.7259 | 1428.7725 | -32.60 | 81 | - | 94 | 1 | 45 | R.RLTSVFGGAAEPPK.G | | 1436.6627 | 1435.6554 | 1435.6983 | -29.89 | 236 | - | 247 | 0 | --- | R.KPDFDAYIDPQK.Q | | 1478.7686 | 1477.7613 | 1477.8140 | -35.65 | 387 | - | 398 | 1 | --- | K.IRDLYEQIITSK.A | | 1478.7686 | 1477.7613 | 1477.8140 | -35.65 | 387 | - | 398 | 1 | 72 | K.IRDLYEQIITSK.A | | 1487.6472 | 1486.6399 | 1486.6762 | -24.41 | 136 | - | 147 | 0 | --- | R.ANNFDLMYEQVK.A + Oxidation (M) | | 1487.6472 | 1486.6399 | 1486.6762 | -24.41 | 136 | - | 147 | 0 | 14 | R.ANNFDLMYEQVK.A + Oxidation (M) | | 1626.8184 | 1625.8111 | 1625.8599 | -30.01 | 178 | - | 191 | 0 | --- | K.ILVIEGLHPMYDAR.V | | 1626.8184 | 1625.8111 | 1625.8599 | -30.01 | 178 | - | 191 | 0 | (47) | K.ILVIEGLHPMYDAR.V | | 1642.8063 | 1641.7990 | 1641.8548 | -34.00 | 178 | - | 191 | 0 | --- | K.ILVIEGLHPMYDAR.V + Oxidation (M) | | 1642.8063 | 1641.7990 | 1641.8548 | -34.00 | 178 | - | 191 | 0 | 63 | K.ILVIEGLHPMYDAR.V + Oxidation (M) | | 1897.9196 | 1896.9123 | 1896.9720 | -31.47 | 194 | - | 209 | 0 | --- | R.ELLDFSIYLDISNEVK.F | | 1960.9640 | 1959.9567 | 1960.0153 | -29.88 | 338 | - | 354 | 0 | --- | R.LDELIYVESHLSNLSTK.F | | 1960.9640 | 1959.9567 | 1960.0153 | -29.88 | 338 | - | 354 | 0 | 82 | R.LDELIYVESHLSNLSTK.F | | 2153.0852 | 2152.0779 | 2152.1416 | -29.57 | 192 | - | 209 | 1 | --- | R.VRELLDFSIYLDISNEVK.F | | 2153.0852 | 2152.0779 | 2152.1416 | -29.57 | 192 | - | 209 | 1 | 33 | R.VRELLDFSIYLDISNEVK.F | | 2173.0437 | 2172.0364 | 2172.0964 | -27.61 | 366 | - | 386 | 0 | --- | K.HADFPGSNNGTGLFQTIVGLK.I | | 2173.0437 | 2172.0364 | 2172.0964 | -27.61 | 366 | - | 386 | 0 | 137 | K.HADFPGSNNGTGLFQTIVGLK.I | | 2378.0488 | 2377.0415 | 2377.0573 | -6.62 | 314 | - | 334 | 0 | --- | K.FSYGPDAYFGHEVSILEMDGK.F + Oxidation (M) | | 2379.1340 | 2378.1267 | 2378.2118 | -35.76 | 335 | - | 354 | 1 | --- | K.FDRLDELIYVESHLSNLSTK.F | | 2379.1340 | 2378.1267 | 2378.2118 | -35.76 | 335 | - | 354 | 1 | 99 | K.FDRLDELIYVESHLSNLSTK.F | | 2780.2334 | 2779.2261 | 2779.2588 | -11.76 | 314 | - | 337 | 1 | --- | K.FSYGPDAYFGHEVSILEMDGKFDR.L | | 2796.2068 | 2795.1995 | 2795.2537 | -19.40 | 314 | - | 337 | 1 | --- | K.FSYGPDAYFGHEVSILEMDGKFDR.L + Oxidation (M) | | 2955.5686 | 2954.5613 | 2954.6117 | -17.05 | 151 | - | 177 | 0 | --- | K.DGVSVEKPIYNHVTGLLDPPELIKPPK.I |  | **No match to:** 712.2527, 731.3414, 747.3649, 768.5129, 774.3273, 832.2842, 832.2842, 834.2917, 842.4828, 868.5253, 936.4446, 1018.5994, 1029.5438, 1038.5026, 1083.4738, 1141.5912, 1142.5894, 1166.5930, 1218.6101, 1292.6405, 1295.6271, 1324.6138, 1328.6390, 1351.6958, 1352.6626, 1381.6052, 1416.6545, 1416.6545, 1422.7029, 1423.6504, 1434.7415, 1443.7512, 1445.7415, 1458.6587, 1465.7183, 1470.7778, 1485.7661, 1492.7897, 1500.7561, 1509.6377, 1553.7395, 1560.7397, 1561.7262, 1576.8102, 1578.8099, 1578.8099, 1582.7128, 1592.7925, 1594.8181, 1596.8049, 1599.7599, 1624.8125, 1635.8174, 1656.8236, 1658.8173, 1664.7905, 1672.7153, 1728.8707, 1784.7726, 1882.9092, 1886.9391, 1906.8735, 1949.9412, 1955.9495, 1978.9230, 2069.9338, 2195.0308, 2411.1648, 2448.2761, 2449.0945, 2510.1248, 2514.1069, 2526.1050, 2536.1055, 2540.0977, 2540.0977, 2552.0996, 2554.1187, 2566.1758, 2695.2117, 2732.2427, 2980.3015, 2993.3057, 2993.3057 | | --- |  | **S33.** | [Cs7g31640.4](http://localhost/mascot/cgi/protein_view.pl?file=../data/20140808/F003859.dat&hit=1)    **Mass:** 32350    **Score:** 576    **Expect:** 1.1e-053  **Matches:** 27 | | --- | --- |  | **Observed** | **Mr(expt)** | **Mr(calc)** | **ppm** | **Start** |  | **End** | **Miss** | **Ions** | **Peptide** | | --- | --- | --- | --- | --- | --- | --- | --- | --- | --- | | 703.3920 | 702.3847 | 702.4025 | -25.28 | 107 | - | 113 | 0 | --- | K.LTGVTGR.D | | 808.4487 | 807.4415 | 807.4603 | -23.30 | 189 | - | 194 | 1 | --- | K.QKYTLR.Y | | 908.3768 | 907.3695 | 907.5379 | -185.52 | 127 | - | 134 | 0 | --- | R.TTYVIAIK.D | | 908.3768 | 907.3695 | 907.5379 | -185.52 | 127 | - | 134 | 0 | --- | R.TTYVIAIK.D | | 912.4955 | 911.4883 | 911.5116 | -25.63 | 182 | - | 188 | 0 | --- | K.LINYYVK.Q | | 921.4420 | 920.4347 | 920.4538 | -20.78 | 164 | - | 171 | 0 | --- | K.MFSPGNLR.A | | 937.4265 | 936.4192 | 936.4487 | -31.56 | 164 | - | 171 | 0 | --- | K.MFSPGNLR.A + Oxidation (M) | | 937.4265 | 936.4192 | 936.4487 | -31.56 | 164 | - | 171 | 0 | 32 | K.MFSPGNLR.A + Oxidation (M) | | 942.4119 | 941.4046 | 941.3769 | 29.4 | 2 | - | 9 | 0 | --- | M.MCMGEALR.T + 2 Oxidation (M) | | 959.4521 | 958.4448 | 958.4720 | -28.33 | 257 | - | 264 | 0 | --- | K.VINDLDDR.T | | 963.4511 | 962.4438 | 962.4556 | -12.33 | 155 | - | 163 | 0 | --- | K.ETTEIGEGK.M | | 1185.4869 | 1184.4796 | 1184.4986 | -16.00 | 172 | - | 181 | 0 | --- | R.ATFDNPDYDK.L | | 1188.5208 | 1187.5135 | 1187.5459 | -27.22 | 278 | - | 287 | 0 | --- | R.FEETLYGSSR.L | | 1188.5208 | 1187.5135 | 1187.5459 | -27.22 | 278 | - | 287 | 0 | 74 | R.FEETLYGSSR.L | | 1237.6150 | 1236.6077 | 1236.6350 | -22.08 | 212 | - | 223 | 0 | --- | K.GIFTNVTSPSSK.A | | 1364.6284 | 1363.6211 | 1363.6554 | -25.15 | 114 | - | 126 | 0 | --- | R.DQVAAAMGIYGPR.T + Oxidation (M) | | 1364.6284 | 1363.6211 | 1363.6554 | -25.15 | 114 | - | 126 | 0 | 46 | R.DQVAAAMGIYGPR.T + Oxidation (M) | | 1649.8180 | 1648.8107 | 1648.8495 | -23.49 | 195 | - | 209 | 0 | --- | R.YTGGMVPDVNQIIVK.E + Oxidation (M) | | 1649.8180 | 1648.8107 | 1648.8495 | -23.49 | 195 | - | 209 | 0 | 75 | R.YTGGMVPDVNQIIVK.E + Oxidation (M) | | 1717.8008 | 1716.7935 | 1716.8359 | -24.69 | 135 | - | 149 | 0 | --- | K.DFPGTHEFLLLDEGK.W | | 1717.8008 | 1716.7935 | 1716.8359 | -24.69 | 135 | - | 149 | 0 | 101 | K.DFPGTHEFLLLDEGK.W | | 1813.8623 | 1812.8550 | 1812.9006 | -25.13 | 273 | - | 287 | 1 | --- | K.NEIIRFEETLYGSSR.L | | 1813.8623 | 1812.8550 | 1812.9006 | -25.13 | 273 | - | 287 | 1 | 59 | K.NEIIRFEETLYGSSR.L | | 1906.8853 | 1905.8780 | 1905.9870 | -57.18 | 195 | - | 211 | 1 | --- | R.YTGGMVPDVNQIIVKEK.G + Oxidation (M) | | 2078.9583 | 2077.9510 | 2077.9996 | -23.40 | 172 | - | 188 | 1 | --- | R.ATFDNPDYDKLINYYVK.Q | | 2078.9583 | 2077.9510 | 2077.9996 | -23.40 | 172 | - | 188 | 1 | 74 | R.ATFDNPDYDKLINYYVK.Q | | 2632.3826 | 2631.3753 | 2631.4272 | -19.70 | 226 | - | 250 | 1 | --- | K.LRLLFEVAPLGLLIENAGGYSSDGK.I |  | **No match to: 7**13.3870, 720.4045, 724.3976, 736.3961, 752.3952, 768.5154, 787.3937, 790.4056, 791.4172, 809.3829, 809.3829, 816.3950, 821.3882, 832.2953, 840.4401, 842.4856, 846.4120, 850.4960, 858.4375, 868.5319, 870.4736, 871.4940, 872.4394, 873.4455, 873.4455, 880.4253, 888.4459, 890.4051, 891.4010, 892.4157, 895.5077, 919.4380, 953.4448, 958.4734, 991.5691, 1045.5391, 1077.4666, 1144.6053, 1144.6053, 1161.5872, 1193.5671, 1215.5453, 1232.5544, 1240.6035, 1254.5773, 1259.4974, 1260.5200, 1288.6353, 1291.6172, 1292.6506, 1297.6626, 1298.6608, 1299.6511, 1300.6324, 1300.9596, 1307.6133, 1320.7041, 1320.7041, 1329.6542, 1329.6542, 1362.6437, 1380.6500, 1386.6307, 1418.6926, 1418.6926, 1434.6934, 1448.7994, 1452.5938, 1455.6476, 1465.7133, 1480.7037, 1585.8262, 1671.7982, 1688.7509, 1699.8190, 1739.7793, 1759.8151, 1774.8688, 1791.8145, 1792.8660, 1792.8660, 1846.8357, 1910.8329, 1958.9845, 2069.9861, 2083.9707, 2100.9302, 2226.0791 | | --- |  | **S44.** | [Cs7g25390.1](http://localhost/mascot/cgi/protein_view.pl?file=../data/20140808/F003910.dat&hit=1)    **Mass:** 35461    **Score:** 613    **Expect:** 2.2e-057  **Matches:** 24 | | --- | --- |  | **Observed** | **Mr(expt)** | **Mr(calc)** | **ppm** | **Start** |  | **End** | **Miss** | **Ions** | **Peptide** | | --- | --- | --- | --- | --- | --- | --- | --- | --- | --- | | 705.3530 | 704.3458 | 704.3639 | -25.81 | 109 | - | 114 | 0 | --- | R.KPGMTR.D + Oxidation (M) | | 782.3511 | 781.3438 | 781.3606 | -21.52 | 162 | - | 168 | 0 | --- | K.AGTYNEK.K | | 798.4562 | 797.4489 | 797.4759 | -33.88 | 50 | - | 56 | 0 | --- | K.LNPLVSR.L | | 890.4052 | 889.3979 | 889.4803 | -92.65 | 1 | - | 7 | 1 | --- | -.MRSSVLR.S + Acetyl (Protein N-term) | | 988.4768 | 987.4695 | 987.4873 | -17.95 | 220 | - | 228 | 0 | --- | K.ANLADEDIK.A | | 1198.5646 | 1197.5573 | 1197.7193 | -135.26 | 8 | - | 19 | 1 | --- | R.SVKTLAKPAGAR.G | | 1219.6566 | 1218.6493 | 1218.6972 | -39.33 | 170 | - | 180 | 0 | --- | K.LFGVTTLDVVR.A | | 1219.6566 | 1218.6493 | 1218.6972 | -39.33 | 170 | - | 180 | 0 | 99 | K.LFGVTTLDVVR.A | | 1233.6274 | 1232.6201 | 1232.5885 | 25.7 | 234 | - | 245 | 0 | --- | R.TQDGGTEVVEAK.A | | 1318.6608 | 1317.6535 | 1317.6929 | -29.85 | 115 | - | 126 | 0 | --- | R.DDLFNINAGIVK.D | | 1318.6608 | 1317.6535 | 1317.6929 | -29.85 | 115 | - | 126 | 0 | 84 | R.DDLFNINAGIVK.D | | 1347.7552 | 1346.7479 | 1346.7922 | -32.88 | 169 | - | 180 | 1 | --- | K.KLFGVTTLDVVR.A | | 1347.7552 | 1346.7479 | 1346.7922 | -32.88 | 169 | - | 180 | 1 | 85 | K.KLFGVTTLDVVR.A | | 1389.6619 | 1388.6546 | 1388.6896 | -25.17 | 233 | - | 245 | 1 | --- | K.RTQDGGTEVVEAK.A | | 1795.0015 | 1793.9942 | 1794.0437 | -27.59 | 31 | - | 49 | 0 | --- | K.VAVLGAAGGIGQPLALLMK.L + Oxidation (M) | | 1795.0015 | 1793.9942 | 1794.0437 | -27.59 | 31 | - | 49 | 0 | 58 | K.VAVLGAAGGIGQPLALLMK.L + Oxidation (M) | | 2281.1128 | 2280.1055 | 2280.1862 | -35.40 | 57 | - | 78 | 0 | --- | R.LALYDIANTPGVAADVGHINTR.S | | 2281.1128 | 2280.1055 | 2280.1862 | -35.40 | 57 | - | 78 | 0 | 144 | R.LALYDIANTPGVAADVGHINTR.S | | 2359.0928 | 2358.0855 | 2358.1591 | -31.19 | 301 | - | 322 | 0 | --- | K.NGVEEVLGLGPLSDFEQEGLEK.L | | 2359.0928 | 2358.0855 | 2358.1591 | -31.19 | 301 | - | 322 | 0 | 39 | K.NGVEEVLGLGPLSDFEQEGLEK.L | | 3067.4702 | 3066.4629 | 3066.6125 | -48.76 | 301 | - | 328 | 1 | --- | K.NGVEEVLGLGPLSDFEQEGLEKLKPELK.A | | 3100.4253 | 3099.4180 | 3099.5183 | -32.35 | 79 | - | 108 | 0 | --- | R.SEVAGYMGNDQLGQALEDSDVVIIPAGVPR.K | | 3112.6404 | 3111.6331 | 3111.7081 | -24.09 | 189 | - | 219 | 0 | --- | K.ANVNVAEVNVPVVGGHAGITILPLFSQATPK.A | | 3116.5093 | 3115.5020 | 3115.5132 | -3.59 | 79 | - | 108 | 0 | --- | R.SEVAGYMGNDQLGQALEDSDVVIIPAGVPR.K + Oxidation (M) |  | **No match to:** 768.5077, 796.4338, 797.4305, 816.4700, 816.4700, 832.2837, 842.4831, 951.5691, 1007.6291, 1018.4903, 1020.6256, 1135.6445, 1150.5836, 1201.6515, 1214.5864, 1217.6526, 1218.6355, 1241.6444, 1251.5948, 1254.5746, 1324.6293, 1339.6471, 1340.6445, 1356.6216, 1367.6729, 1428.8234, 1444.7697, 1446.7316, 1462.6799, 1478.7535, 1478.7535, 1556.8650, 1682.7814, 1686.7771, 1686.7771, 1698.7803, 1765.9371, 1805.8512, 1816.9740, 1829.9529, 1829.9529, 1842.8563, 1846.7957, 1846.7957, 1857.8005, 1860.8286, 2138.9917, 2157.0034, 2157.0034, 2176.0027, 2237.1030, 2240.1321, 2241.0918, 2263.1052, 2264.1038, 2268.1033, 2268.1033, 2269.9978, 2277.0635, 2280.1340, 2282.6567, 2288.0984, 2291.0479, 2295.1323, 2297.1301, 2298.0969, 2302.0386, 2303.1028, 2310.1250, 2314.0923, 2316.0876, 2316.0876, 2330.1228, 2331.1216, 2332.0974, 2332.0974, 2339.1113, 2342.0613, 2343.0500, 2346.0798, 2348.0947, 2360.0947, 2364.1150, 2373.0862, 2381.0400, 2927.4004, 3052.4683, 3056.3723, 3099.5007, 3130.5071, 3134.5439 | | --- |  | **S45.** | [Cs7g25390.3](http://localhost/mascot/cgi/protein_view.pl?file=../data/20140808/F003911.dat&hit=1)    **Mass:** 31626    **Score:** 287    **Expect:** 8.8e-025  **Matches:** 9 | | --- | --- |  | **Observed** | **Mr(expt)** | **Mr(calc)** | **ppm** | **Start** |  | **End** | **Miss** | **Ions** | **Peptide** | | --- | --- | --- | --- | --- | --- | --- | --- | --- | --- | | 705.3500 | 704.3428 | 704.3639 | -30.06 | 109 | - | 114 | 0 | --- | R.KPGMTR.D + Oxidation (M) | | 798.4624 | 797.4551 | 797.4759 | -26.08 | 50 | - | 56 | 0 | --- | K.LNPLVSR.L | | 1219.6621 | 1218.6548 | 1218.6972 | -34.81 | 170 | - | 180 | 0 | --- | K.LFGVTTLDVVR.A | | 1219.6621 | 1218.6548 | 1218.6972 | -34.81 | 170 | - | 180 | 0 | 90 | K.LFGVTTLDVVR.A | | 1318.6583 | 1317.6510 | 1317.6929 | -31.75 | 115 | - | 126 | 0 | --- | R.DDLFNINAGIVK.D | | 1347.7518 | 1346.7445 | 1346.7922 | -35.40 | 169 | - | 180 | 1 | --- | K.KLFGVTTLDVVR.A | | 2281.1116 | 2280.1043 | 2280.1862 | -35.92 | 57 | - | 78 | 0 | --- | R.LALYDIANTPGVAADVGHINTR.S | | 2281.1116 | 2280.1043 | 2280.1862 | -35.92 | 57 | - | 78 | 0 | 171 | R.LALYDIANTPGVAADVGHINTR.S | | 3116.4978 | 3115.4905 | 3115.5132 | -7.28 | 79 | - | 108 | 0 | --- | R.SEVAGYMGNDQLGQALEDSDVVIIPAGVPR.K + Oxidation (M) |  | **No match to:** 703.3825, 734.4754, 756.4470, 767.4472, 768.4816, 784.4673, 832.2874, 833.4307, 834.4445, 842.4781, 914.5103, 938.4963, 950.4723, 964.4901, 966.4683, 1020.6103, 1041.4895, 1051.6580, 1139.4232, 1170.6833, 1175.6240, 1180.5011, 1201.6409, 1207.5864, 1218.6228, 1247.5735, 1251.6003, 1254.5767, 1254.5767, 1263.5801, 1268.5890, 1324.5983, 1329.7231, 1341.6743, 1367.6780, 1376.7350, 1380.7030, 1381.7249, 1381.7249, 1382.6952, 1383.6930, 1398.7030, 1400.7123, 1402.6996, 1426.7654, 1428.7543, 1430.7053, 1430.7053, 1431.7024, 1444.7463, 1446.6925, 1446.6925, 1458.7271, 1462.6908, 1462.6908, 1465.7124, 1467.7322, 1558.8085, 1566.7874, 1569.7616, 1571.7430, 1605.8193, 1656.8467, 1658.8368, 1658.8368, 1662.8945, 1680.8430, 1685.8223, 1699.8269, 1711.7822, 1711.7822, 1725.8035, 1786.8112, 1795.9478, 1800.8518, 1802.8663, 1805.8448, 1805.8448, 1818.7545, 1819.8048, 1825.9204, 1865.8538, 1955.8503, 1964.0675, 1964.0675, 2013.9690, 2077.9797, 2109.0095, 2266.1069, 2268.1199, 2268.1199, 2297.0886, 2332.1089, 2332.1089, 2347.0969, 2351.0659, 2351.0659, 2363.1006, 2365.0820, 2365.0820, 2379.1033, 2410.1851, 2461.0833, 2709.3477, 3054.3540, 3113.6580 | | --- |  | **S30.** | [Cs6g15540.1](http://localhost/mascot/cgi/protein_view.pl?file=../data/20140808/F003858.dat&hit=1)    **Mass:** 47758    **Score:** 928    **Expect:** 7e-089  **Matches:** 30 | | --- | --- |  | **Observed** | **Mr(expt)** | **Mr(calc)** | **ppm** | **Start** |  | **End** | **Miss** | **Ions** | **Peptide** | | --- | --- | --- | --- | --- | --- | --- | --- | --- | --- | | 765.3603 | 764.3530 | 764.3817 | -37.53 | 12 | - | 17 | 0 | --- | R.QIFDSR.G | | 806.4181 | 805.4108 | 805.4446 | -41.96 | 417 | - | 422 | 0 | --- | K.YNQLLR.I | | 806.4181 | 805.4108 | 805.4446 | -41.96 | 417 | - | 422 | 0 | 36 | K.YNQLLR.I | | 978.4613 | 977.4540 | 977.4971 | -44.02 | 438 | - | 445 | 1 | --- | K.FRAPVEPY.- | | 978.4613 | 977.4540 | 977.4971 | -44.02 | 438 | - | 445 | 1 | 42 | K.FRAPVEPY.- | | 992.4866 | 991.4793 | 991.5199 | -40.97 | 10 | - | 17 | 1 | --- | K.ARQIFDSR.G | | 992.4866 | 991.4793 | 991.5199 | -40.97 | 10 | - | 17 | 1 | --- | K.ARQIFDSR.G | | 1212.5392 | 1211.5319 | 1211.6121 | -66.18 | 190 | - | 199 | 0 | --- | K.MGVEVYHHLK.A | | 1215.5170 | 1214.5097 | 1214.5615 | -42.61 | 372 | - | 382 | 0 | --- | K.QAGWGVMASHR.S + Oxidation (M) | | 1222.6185 | 1221.6112 | 1221.6605 | -40.32 | 279 | - | 289 | 1 | --- | K.ISGDALKDLYK.S | | 1228.5717 | 1227.5644 | 1227.6070 | -34.70 | 190 | - | 199 | 0 | --- | K.MGVEVYHHLK.A + Oxidation (M) | | 1510.7988 | 1509.7915 | 1509.8403 | -32.29 | 323 | - | 336 | 0 | --- | K.VQIVGDDLLVTNPK.R | | 1549.7229 | 1548.7156 | 1548.7671 | -33.24 | 423 | - | 437 | 0 | --- | R.IEEELGAEAVYAGAK.F | | 1601.7856 | 1600.7783 | 1600.8420 | -39.80 | 354 | - | 368 | 0 | --- | K.VNQIGSVTESIEAVR.M | | 1601.7856 | 1600.7783 | 1600.8420 | -39.80 | 354 | - | 368 | 0 | 144 | K.VNQIGSVTESIEAVR.M | | 1623.7838 | 1622.7765 | 1622.8967 | -74.02 | 190 | - | 203 | 1 | --- | K.MGVEVYHHLKAVIK.K | | 1666.8967 | 1665.8894 | 1665.9414 | -31.18 | 323 | - | 337 | 1 | --- | K.VQIVGDDLLVTNPKR.V | | 1803.8285 | 1802.8212 | 1802.8397 | -10.23 | 246 | - | 262 | 0 | --- | K.VVIGMDVAASEFYGSDK.T + Oxidation (M) | | 1804.8717 | 1803.8644 | 1803.9366 | -40.04 | 36 | - | 53 | 0 | --- | R.AAVPSGASTGIYEALELR.D | | 1804.8717 | 1803.8644 | 1803.9366 | -40.04 | 36 | - | 53 | 0 | 152 | R.AAVPSGASTGIYEALELR.D | | 1853.8307 | 1852.8234 | 1852.8916 | -36.77 | 18 | - | 35 | 0 | --- | R.GNPTVEVDVTTSDGHVAR.A | | 1853.8307 | 1852.8234 | 1852.8916 | -36.77 | 18 | - | 35 | 0 | 154 | R.GNPTVEVDVTTSDGHVAR.A | | 1900.8984 | 1899.8911 | 1899.9474 | -29.64 | 169 | - | 185 | 0 | --- | K.LAMQEFMILPVGASSFK.E + 2 Oxidation (M) | | 1901.8641 | 1900.8568 | 1900.8439 | 6.81 | 263 | - | 278 | 1 | --- | K.TYDLNFKEENNDGSQK.I | | 2118.0728 | 2117.0655 | 2117.1382 | -34.31 | 148 | - | 168 | 0 | --- | K.NLVLPVPAFNVINGGSHAGNK.L | | 2118.0728 | 2117.0655 | 2117.1382 | -34.31 | 148 | - | 168 | 0 | 131 | K.NLVLPVPAFNVINGGSHAGNK.L | | 2252.0574 | 2251.0501 | 2251.1220 | -31.92 | 383 | - | 404 | 0 | --- | R.SGETEDTFIADLSVGLATGQIK.T | | 2354.2119 | 2353.2046 | 2353.2741 | -29.51 | 315 | - | 336 | 1 | --- | K.LTSEVGEKVQIVGDDLLVTNPK.R | | 2697.2356 | 2696.2283 | 2696.3293 | -37.45 | 36 | - | 62 | 1 | --- | R.AAVPSGASTGIYEALELRDGGSDYLGK.G | | 2697.2356 | 2696.2283 | 2696.3293 | -37.45 | 36 | - | 62 | 1 | 147 | R.AAVPSGASTGIYEALELRDGGSDYLGK.G |  | **No match to:** 712.2340, 748.3326, 814.4092, 828.4152, 832.2838, 841.3976, 842.4766, 870.4650, 910.4209, 932.4856, 959.5370, 977.4617, 985.5453, 1000.4577, 1003.5451, 1015.5948, 1021.4943, 1045.5679, 1116.5732, 1120.5817, 1131.5881, 1133.6136, 1134.5190, 1150.5132, 1164.5895, 1167.5287, 1173.6326, 1182.5170, 1187.6353, 1196.5033, 1198.5070, 1198.5070, 1201.6653, 1202.5116, 1214.5070, 1214.5070, 1218.6062, 1219.5959, 1230.5140, 1231.5190, 1247.5247, 1258.5190, 1274.5201, 1290.5193, 1312.7184, 1327.6024, 1328.6227, 1328.6227, 1371.6582, 1431.6450, 1433.7205, 1445.6736, 1465.7089, 1465.7089, 1472.6479, 1487.7131, 1492.7367, 1499.8185, 1513.7472, 1553.7501, 1563.7441, 1583.7886, 1615.8070, 1615.8070, 1625.8079, 1625.8079, 1786.8689, 1800.7731, 1802.8446, 1817.8556, 1818.8923, 1818.8923, 1835.8540, 1836.8740, 1867.8590, 1909.9001, 1919.9211, 1949.9205, 1954.9670, 1973.9137, 2076.9883, 2140.0464, 2141.0491, 2185.9968, 2398.1663 | | --- |  | **S36.** | [Cs6g15540.1](http://localhost/mascot/cgi/protein_view.pl?file=../data/20140415/F003343.dat&hit=1)    **Mass:** 47758    **Score:** 531    **Expect:** 3.5e-049  **Matches:** 15 | | --- | --- |  | **Observed** | **Mr(expt)** | **Mr(calc)** | **ppm** | **Start** |  | **End** | **Miss** | **Ions** | **Peptide** | | --- | --- | --- | --- | --- | --- | --- | --- | --- | --- | | 765.3636 | 764.3563 | 764.3817 | -33.21 | 12 | - | 17 | 0 | --- | R.QIFDSR.G | | 806.4239 | 805.4167 | 805.4446 | -34.69 | 417 | - | 422 | 0 | --- | K.YNQLLR.I | | 816.5915 | 815.5842 | 815.5116 | 89.0 | 2 | - | 9 | 0 | --- | M.AITITAVK.A | | 978.4809 | 977.4736 | 977.4971 | -23.97 | 438 | - | 445 | 1 | --- | K.FRAPVEPY.- | | 978.4809 | 977.4736 | 977.4971 | -23.97 | 438 | - | 445 | 1 | 25 | K.FRAPVEPY.- | | 1215.5475 | 1214.5402 | 1214.5615 | -17.50 | 372 | - | 382 | 0 | --- | K.QAGWGVMASHR.S + Oxidation (M) | | 1215.5475 | 1214.5402 | 1214.5615 | -17.50 | 372 | - | 382 | 0 | 21 | K.QAGWGVMASHR.S + Oxidation (M) | | 1601.8186 | 1600.8113 | 1600.8420 | -19.19 | 354 | - | 368 | 0 | --- | K.VNQIGSVTESIEAVR.M | | 1601.8186 | 1600.8113 | 1600.8420 | -19.19 | 354 | - | 368 | 0 | 136 | K.VNQIGSVTESIEAVR.M | | 1804.9131 | 1803.9058 | 1803.9366 | -17.09 | 36 | - | 53 | 0 | --- | R.AAVPSGASTGIYEALELR.D | | 1804.9131 | 1803.9058 | 1803.9366 | -17.09 | 36 | - | 53 | 0 | 118 | R.AAVPSGASTGIYEALELR.D | | 1853.8885 | 1852.8812 | 1852.8916 | -5.58 | 18 | - | 35 | 0 | --- | R.GNPTVEVDVTTSDGHVAR.A | | 1853.8885 | 1852.8812 | 1852.8916 | -5.58 | 18 | - | 35 | 0 | 84 | R.GNPTVEVDVTTSDGHVAR.A | | 2118.1296 | 2117.1223 | 2117.1382 | -7.48 | 148 | - | 168 | 0 | --- | K.NLVLPVPAFNVINGGSHAGNK.L | | 2118.1296 | 2117.1223 | 2117.1382 | -7.48 | 148 | - | 168 | 0 | 69 | K.NLVLPVPAFNVINGGSHAGNK.L |  | **No match to:** 728.5381, 748.3422, 860.6140, 1198.5225, 1198.5225, 1219.5320, 1230.5155, 1230.5155, 1247.5406, 1247.5406, 1509.6324, 1509.6324, 1625.8503, 1818.9336, 1818.9336, 1819.9175, 2119.1155 | | --- |  | **S43.** | [Cs2g21190.3](http://localhost/mascot/cgi/protein_view.pl?file=../data/20140415/F003350.dat&hit=1)    **Mass:** 43636    **Score:** 268    **Expect:** 7e-023  **Matches:** 8 | | --- | --- |  | **Observed** | **Mr(expt)** | **Mr(calc)** | **ppm** | **Start** |  | **End** | **Miss** | **Ions** | **Peptide** | | --- | --- | --- | --- | --- | --- | --- | --- | --- | --- | | 852.4905 | 851.4832 | 851.5593 | -89.34 | 274 | - | 281 | 0 | --- | K.GLVVPVIR.N | | 852.4905 | 851.4832 | 851.5593 | -89.34 | 274 | - | 281 | 0 | 51 | K.GLVVPVIR.N | | 881.4274 | 880.4201 | 880.4807 | -68.81 | 379 | - | 385 | 0 | --- | R.EAVFFLR.R | | 881.4274 | 880.4201 | 880.4807 | -68.81 | 379 | - | 385 | 0 | 48 | R.EAVFFLR.R | | 1825.7832 | 1824.7759 | 1824.9945 | -119.76 | 93 | - | 111 | 1 | --- | K.IAVISKSGEGVAQAAPAEK.A | | 1825.7832 | 1824.7759 | 1824.9945 | -119.76 | 93 | - | 111 | 1 | --- | K.IAVISKSGEGVAQAAPAEK.A | | 2564.2097 | 2563.2024 | 2563.3394 | -53.45 | 237 | - | 260 | 0 | --- | K.AAVSALQHQPVVNAVIDGDDIIYR.D | | 2564.2097 | 2563.2024 | 2563.3394 | -53.45 | 237 | - | 260 | 0 | 144 | K.AAVSALQHQPVVNAVIDGDDIIYR.D |  | **No match to:** 728.5129, 772.5304, 816.5549, 860.5763, 863.4139, 863.4139, 904.5962, 904.5962, 948.6343, 1008.4190, 1008.4190, 1044.3939, 1044.3939, 1324.5564, 1324.5564, 1465.6660, 1465.6660, 1537.6508, 1537.6508, 1891.8732, 1891.8732, 1903.8826, 2308.9888, 2308.9888 | | --- |   **Antioxidation and detoxification**   | **S1.** | [Cs6g13880.1](http://localhost/mascot/cgi/protein_view.pl?file=../data/20140808/F003832.dat&hit=1)    **Mass:** 29475    **Score:** 465    **Expect:** 1.4e-042  **Matches:** 17 | | --- | --- |  | **Observed** | **Mr(expt)** | **Mr(calc)** | **ppm** | **Start** |  | **End** | **Miss** | **Ions** | **Peptide** | | --- | --- | --- | --- | --- | --- | --- | --- | --- | --- | | **746.3940** | 745.3867 | 745.3970 | -13.84 | 166 | - | 173 | 0 | --- | K.SGGLGDLK.Y | | **805.4510** | 804.4437 | 804.4745 | -38.31 | 201 | - | 207 | 0 | --- | R.GLFIIDK.E | | **805.4510** | 804.4437 | 804.4745 | -38.31 | 201 | - | 207 | 0 | 27 | R.GLFIIDK.E | | **818.3683** | 817.3610 | 817.4004 | -48.15 | 256 | - | 262 | 0 | --- | K.SMKPDPK.L + Oxidation (M) | | **819.3881** | 818.3808 | 818.4134 | -39.77 | 224 | - | 230 | 0 | --- | R.SVDETLR.T | | **819.3881** | 818.3808 | 818.4134 | -39.77 | 224 | - | 230 | 0 | 50 | R.SVDETLR.T | | **1033.5559** | 1032.5486 | 1032.5855 | -35.74 | 174 | - | 182 | 0 | --- | K.YPLIADITK.S | | **1501.7738** | 1500.7665 | 1500.8300 | -42.31 | 187 | - | 200 | 0 | --- | K.SYGVLIPDQGIALR.G | | **1501.7738** | 1500.7665 | 1500.8300 | -42.31 | 187 | - | 200 | 0 | 114 | K.SYGVLIPDQGIALR.G | | **1721.8551** | 1720.8478 | 1720.9220 | -43.11 | 208 | - | 223 | 0 | --- | K.EGVIQHSTINNLAIGR.S | | **1721.8551** | 1720.8478 | 1720.9220 | -43.11 | 208 | - | 223 | 0 | 161 | K.EGVIQHSTINNLAIGR.S | | **1760.9124** | 1759.9051 | 1759.9720 | -37.99 | 166 | - | 182 | 1 | --- | K.SGGLGDLKYPLIADITK.S | | **1760.9124** | 1759.9051 | 1759.9720 | -37.99 | 166 | - | 182 | 1 | 46 | K.SGGLGDLKYPLIADITK.S | | **2508.3147** | 2507.3074 | 2507.3860 | -31.33 | 201 | - | 223 | 1 | --- | R.GLFIIDKEGVIQHSTINNLAIGR.S | | **2522.2698** | 2521.2625 | 2521.3248 | -24.72 | 208 | - | 230 | 1 | --- | K.EGVIQHSTINNLAIGRSVDETLR.T | | **2688.2505** | 2687.2432 | 2687.3555 | -41.78 | 141 | - | 164 | 0 | --- | K.LNTEILGVSTDSVFSHLAWVQTDR.K | | **3132.5513** | 3131.5440 | 3131.5718 | -8.88 | 109 | - | 134 | 1 | --- | K.KYVILFFYPLDFTFVCPTEITAFSDR.Y |  | **No match to: 712.2403, 768.4570, 786.3353, 804.2565, 809.4197, 827.4433, 832.2798, 832.2798, 833.4011, 841.3774, 842.4786, 861.0380, 868.4971, 981.5079, 1051.6545, 1136.5293, 1136.5293, 1150.5387, 1155.5627, 1364.1992, 1483.7794, 1485.7584, 1497.7743, 1498.7889, 1500.7844, 1513.7948, 1514.7811, 1515.7850, 1515.7850, 1523.7628, 1527.7911, 1529.7758, 1530.7859, 1537.7715, 1543.7878, 1553.7334, 1559.7802, 1575.7183, 1611.7834, 1638.8005, 1654.8923, 1654.8923, 1668.9120, 1668.9120, 1702.8201, 1703.8499, 1703.8499, 1707.7942, 1710.9673, 1719.8591, 1720.8325, 1735.8651, 1743.8478, 1874.9403, 1931.0300, 1933.9089, 2674.2490, 2679.1790, 2687.2632, 2689.2607, 2690.2620, 2692.2512, 2692.2512, 2699.2209, 2702.2554, 2704.2539, 2706.2571, 2711.2256, 2716.2864, 2719.2537, 2720.2520, 2720.2520, 2725.2373, 2735.2698, 2744.2559, 2744.2559, 2749.2534, 2752.2266, 2843.2791, 2844.3127, 2846.3120, 2857.3459, 2858.3047, 2858.3047, 2859.3120, 2862.2964, 2864.3069, 2871.3445, 2875.3030, 2885.3208, 2900.2881, 2902.3640, 3053.5298, 3061.4128, 3117.5371, 3118.5269, 3139.4285, 3155.4094** | | --- |  | **S20.** | [orange1.1t02144.1](http://localhost/mascot/cgi/protein_view.pl?file=../data/20140808/F003846.dat&hit=1)    **Mass:** 41327    **Score:** 880    **Expect:** 4.4e-084  **Matches:** 28 | | --- | --- |  | **Observed** | **Mr(expt)** | **Mr(calc)** | **ppm** | **Start** |  | **End** | **Miss** | **Ions** | **Peptide** | | --- | --- | --- | --- | --- | --- | --- | --- | --- | --- | | 719.3373 | 718.3300 | 718.4225 | -128.73 | 22 | - | 28 | 0 | --- | K.SSLATLK.L | | 771.3798 | 770.3725 | 770.4035 | -40.22 | 352 | - | 358 | 0 | --- | K.RPENAGK.L | | 842.4788 | 841.4715 | 841.4657 | 6.91 | 264 | - | 270 | 1 | --- | K.EKNPNIK.V | | 847.4412 | 846.4339 | 846.5174 | -98.71 | 21 | - | 28 | 1 | --- | K.KSSLATLK.L | | 1114.5925 | 1113.5852 | 1113.6295 | -39.75 | 292 | - | 302 | 0 | --- | K.IQGIGAGFVPR.N | | 1114.5925 | 1113.5852 | 1113.6295 | -39.75 | 292 | - | 302 | 0 | 109 | K.IQGIGAGFVPR.N | | 1267.6927 | 1266.6854 | 1266.5658 | 94.5 | 105 | - | 115 | 0 | --- | K.LEIMEPCCSVK.D + Oxidation (M) | | 1294.6714 | 1293.6641 | 1293.7003 | -27.93 | 85 | - | 95 | 0 | --- | K.TPMVYLNTIVK.G + Oxidation (M) | | 1312.6791 | 1311.6718 | 1311.7187 | -35.72 | 371 | - | 381 | 0 | --- | R.YLSSVLFQSIR.E | | 1312.6791 | 1311.6718 | 1311.7187 | -35.72 | 371 | - | 381 | 0 | 74 | R.YLSSVLFQSIR.E | | 1317.6327 | 1316.6254 | 1316.7564 | -99.50 | 29 | - | 40 | 1 | --- | K.LGYISPITAARR.L | | 1321.6542 | 1320.6469 | 1320.6925 | -34.52 | 180 | - | 192 | 0 | --- | K.AFGAELVLTDSAK.G | | 1325.6150 | 1324.6077 | 1324.6333 | -19.30 | 118 | - | 129 | 0 | --- | R.IGFSMIADAEQK.G + Oxidation (M) | | 1334.6949 | 1333.6876 | 1333.7394 | -38.84 | 359 | - | 370 | 0 | --- | K.LIAVVFPSFGER.Y | | 1334.6949 | 1333.6876 | 1333.7394 | -38.84 | 359 | - | 370 | 0 | 88 | K.LIAVVFPSFGER.Y | | 1461.7177 | 1460.7104 | 1460.7731 | -42.89 | 162 | - | 174 | 0 | --- | K.LILTMPASMSLER.R | | 1477.7242 | 1476.7169 | 1476.7680 | -34.58 | 162 | - | 174 | 0 | --- | K.LILTMPASMSLER.R + Oxidation (M) | | 1493.7203 | 1492.7130 | 1492.7629 | -33.42 | 162 | - | 174 | 0 | --- | K.LILTMPASMSLER.R + 2 Oxidation (M) | | 1493.7203 | 1492.7130 | 1492.7629 | -33.42 | 162 | - | 174 | 0 | 18 | K.LILTMPASMSLER.R + 2 Oxidation (M) | | 1649.7759 | 1648.7686 | 1648.8640 | -57.85 | 162 | - | 175 | 1 | --- | K.LILTMPASMSLERR.V + 2 Oxidation (M) | | 1747.8721 | 1746.8648 | 1746.9265 | -35.30 | 242 | - | 260 | 0 | --- | K.VDIFIGGIGTGGTISGAGR.Y | | 1747.8721 | 1746.8648 | 1746.9265 | -35.30 | 242 | - | 260 | 0 | 197 | K.VDIFIGGIGTGGTISGAGR.Y | | 1846.7961 | 1845.7888 | 1845.8533 | -34.95 | 225 | - | 239 | 0 | --- | K.IHYETTGPEIWEDTR.G | | 1846.7961 | 1845.7888 | 1845.8533 | -34.95 | 225 | - | 239 | 0 | 108 | K.IHYETTGPEIWEDTR.G | | 1932.9813 | 1931.9740 | 1932.0429 | -35.65 | 240 | - | 260 | 1 | --- | R.GKVDIFIGGIGTGGTISGAGR.Y | | 1932.9813 | 1931.9740 | 1932.0429 | -35.65 | 240 | - | 260 | 1 | 176 | R.GKVDIFIGGIGTGGTISGAGR.Y | | 2116.0723 | 2115.0650 | 2115.1324 | -31.85 | 271 | - | 291 | 0 | --- | K.VIGIEPSESNILSGGKPGPHK.I | | **2146.1113** | 2145.1040 | 2145.1681 | -29.88 | 137 | - | 158 | 0 | --- | K.SILVEPTSGNTGIGLAFIAASK.G |  | **No match to:** 733.4110, 737.3397, 832.2813, 851.4111, 851.4111, 856.4742, 865.4329, 905.4043, 995.5177, 1002.4769, 1018.4639, 1094.5364, 1136.5894, 1182.6057, 1200.6193, 1223.5593, 1256.6361, 1261.6162, 1272.6296, 1299.6514, 1300.7112, 1316.6785, 1324.6371, 1327.6472, 1348.7190, 1356.6506, 1357.6558, 1362.7141, 1376.7476, 1380.6332, 1381.5989, 1413.7188, 1427.7412, 1428.7941, 1429.7278, 1434.7117, 1434.7117, 1448.7288, 1465.7195, 1475.7178, 1491.7476, 1507.7379, 1524.6637, 1588.7314, 1626.8212, 1636.7811, 1636.7811, 1640.7942, 1645.7800, 1652.7167, 1652.7167, 1661.7786, 1687.8033, 1703.7982, 1729.8685, 1736.8365, 1784.8545, 1800.8400, 1800.8400, 1850.7986, 1860.8063, 1862.8019, 1864.8071, 1868.9551, 1874.8313, 1876.8207, 1878.8158, 1881.8625, 1914.9789, 1942.9557, 1959.9536, 1960.9680, 1960.9680, 1993.9194, 2049.9915, 2478.1392, 2479.0674, 2542.1206, 2622.3545, 2635.2849, 2650.3027, 2653.3059, 2653.3059, 2878.2874, 2878.2874, 2892.2896, 3034.4539 | | --- |  | **S39.** | [Cs9g06970.1](http://localhost/mascot/cgi/protein_view.pl?file=../data/20140415/F003346.dat&hit=1)    **Mass:** 34256    **Score:** 114    **Expect:** 1.8e-007  **Matches:** 6 | | --- | --- |  | **Observed** | **Mr(expt)** | **Mr(calc)** | **ppm** | **Start** |  | **End** | **Miss** | **Ions** | **Peptide** | | --- | --- | --- | --- | --- | --- | --- | --- | --- | --- | | 1299.6622 | 1298.6549 | 1298.6870 | -24.73 | 305 | - | 315 | 0 | --- | R.YLSSVLFESVR.K | | 1299.6622 | 1298.6549 | 1298.6870 | -24.73 | 305 | - | 315 | 0 | 49 | R.YLSSVLFESVR.K | | 1376.7594 | 1375.7521 | 1375.7864 | -24.90 | 293 | - | 304 | 0 | --- | K.LIVVIFPSFGER.Y | | 1376.7594 | 1375.7521 | 1375.7864 | -24.90 | 293 | - | 304 | 0 | 49 | K.LIVVIFPSFGER.Y | | 1647.9286 | 1646.9213 | 1646.8702 | 31.1 | 114 | - | 129 | 1 | --- | R.AFGAELVLTDPAKGMK.G | | 1647.9286 | 1646.9213 | 1646.8702 | 31.1 | 114 | - | 129 | 1 | --- | R.AFGAELVLTDPAKGMK.G |  | **No match to:** 706.3644, 772.5644, 807.4151, 816.5798, 830.3970, 860.6146, 860.6146, 904.6284, 904.6284, 948.6633, 950.4244, 950.4244, 1121.5437, 1121.5437, 1128.5148, 1128.5148, 1324.6021, 1324.6021, 1407.5763, 1407.5763, 1619.7738, 1619.7738, 1896.9540, 1896.9540, 1960.9708, 1960.9708, 2644.3464, 2645.3159, 2645.3159, 3062.5242 | | --- |  | **S34.** | [Cs8g17370.1](http://localhost/mascot/cgi/protein_view.pl?file=../data/20140415/F003341.dat&hit=1)    **Mass:** 27553    **Score:** 392    **Expect:** 2.8e-035  **Matches:** 19 | | --- | --- |  | **Observed** | **Mr(expt)** | **Mr(calc)** | **ppm** | **Start** |  | **End** | **Miss** | **Ions** | **Peptide** | | --- | --- | --- | --- | --- | --- | --- | --- | --- | --- | | 839.4228 | 838.4155 | 838.4046 | 13.1 | 136 | - | 142 | 0 | --- | K.QGNDHLR.Q | | 883.4394 | 882.4321 | 882.4195 | 14.3 | 123 | - | 130 | 0 | --- | K.AEPPQEGR.L | | 917.5276 | 916.5204 | 916.4623 | 63.4 | 31 | - | 38 | 0 | --- | K.NCAPLMLR.I | | 917.5276 | 916.5204 | 916.4623 | 63.4 | 31 | - | 38 | 0 | 11 | K.NCAPLMLR.I | | 923.4642 | 922.4570 | 922.4331 | 25.9 | 53 | - | 61 | 0 | --- | K.TGGPFGTMR.L | | 939.4473 | 938.4401 | 938.4280 | 12.8 | 53 | - | 61 | 0 | --- | K.TGGPFGTMR.L + Oxidation (M) | | 939.4473 | 938.4401 | 938.4280 | 12.8 | 53 | - | 61 | 0 | --- | K.TGGPFGTMR.L + Oxidation (M) | | 1036.4951 | 1035.4878 | 1035.4774 | 10.1 | 173 | - | 181 | 0 | --- | R.SGFEGPWTR.N | | 1036.4951 | 1035.4878 | 1035.4774 | 10.1 | 173 | - | 181 | 0 | 30 | R.SGFEGPWTR.N | | 1152.5018 | 1151.4945 | 1151.5757 | -70.53 | 51 | - | 61 | 1 | --- | K.TKTGGPFGTMR.L | | 1241.6022 | 1240.5949 | 1240.5684 | 21.4 | 120 | - | 130 | 1 | --- | R.DDKAEPPQEGR.L | | 1295.7257 | 1294.7184 | 1294.6994 | 14.7 | 155 | - | 167 | 0 | --- | K.DIVALSGGHTLGR.C | | 1611.9336 | 1610.9263 | 1610.9032 | 14.4 | 210 | - | 223 | 0 | --- | K.ALLDDPVFRPLVEK.Y | | 1611.9336 | 1610.9263 | 1610.9032 | 14.4 | 210 | - | 223 | 0 | 53 | K.ALLDDPVFRPLVEK.Y | | 1849.9890 | 1848.9817 | 1848.9442 | 20.3 | 62 | - | 79 | 0 | --- | R.LAAEQAHSANNGLDIAVR.L | | 1849.9890 | 1848.9817 | 1848.9442 | 20.3 | 62 | - | 79 | 0 | 114 | R.LAAEQAHSANNGLDIAVR.L | | 2046.9486 | 2045.9413 | 2045.9007 | 19.9 | 224 | - | 241 | 0 | --- | K.YAADEDAFFADYAEAHLK.L | | 2573.3945 | 2572.3872 | 2572.3068 | 31.3 | 143 | - | 167 | 1 | --- | R.QVFGAQMGLSDKDIVALSGGHTLGR.C + Oxidation (M) | | 2573.3945 | 2572.3872 | 2572.3068 | 31.3 | 143 | - | 167 | 1 | 100 | R.QVFGAQMGLSDKDIVALSGGHTLGR.C + Oxidation (M) |  | **No match to**: 728.5670, 772.5941, 816.6177, 822.3975, 860.6493, 861.4143, 875.4606, 904.6840, 926.5024, 932.7064, 948.6949, 951.5219, 974.4963, 979.5692, 990.5053, 990.5053, 992.7404, 1006.4941, 1034.4958, 1040.4901, 1045.5857, 1050.4901, 1051.4943, 1052.4941, 1052.4941, 1066.5062, 1068.4921, 1068.4921, 1084.6709, 1129.6078, 1160.6617, 1187.5386, 1206.5350, 1251.5481, 1255.7107, 1293.6799, 1361.7227, 1413.6489, 1414.7144, 1414.7144, 1569.7632, 1594.9121, 1619.8688, 1625.9460, 1625.9460, 1634.9319, 1832.9694, 1922.0723, 1922.0723, 1938.0497, 2057.0613, 2057.0613, 2073.0977, 2267.2341, 2267.2341, 2281.2461, 2492.1675, 2492.4092, 2493.3535, 2508.3708, 2509.3188, 2556.3779, 2556.3779 | | --- |  | **S24.** | [Cs5g03830.1](http://localhost/mascot/cgi/protein_view.pl?file=../data/20140808/F003850.dat&hit=1)    **Mass:** 25360    **Score:** 646    **Expect:** 1.1e-060  **Matches:** 21 | | --- | --- |  | **Observed** | **Mr(expt)** | **Mr(calc)** | **ppm** | **Start** |  | **End** | **Miss** | **Ions** | **Peptide** | | --- | --- | --- | --- | --- | --- | --- | --- | --- | --- | | 802.3816 | 801.3743 | 801.3981 | -29.64 | 200 | - | 206 | 0 | --- | K.EGNVVER.Y | | 802.3816 | 801.3743 | 801.3981 | -29.64 | 200 | - | 206 | 0 | 42 | K.EGNVVER.Y | | 850.5132 | 849.5059 | 849.3949 | 131 | 2 | - | 8 | 1 | --- | M.LRCCASR.Y + Acetyl (Protein N-term) | | 893.4437 | 892.4365 | 892.4654 | -32.48 | 181 | - | 189 | 0 | --- | K.GGLFGDSIK.W | | 893.4437 | 892.4365 | 892.4654 | -32.48 | 181 | - | 189 | 0 | 82 | K.GGLFGDSIK.W | | 966.4613 | 965.4540 | 965.4858 | -32.94 | 154 | - | 161 | 0 | --- | K.AEFPIFDK.V | | 966.4613 | 965.4540 | 965.4858 | -32.94 | 154 | - | 161 | 0 | 26 | K.AEFPIFDK.V | | 997.5150 | 996.5077 | 996.4303 | 77.7 | 1 | - | 8 | 1 | --- | -.MLRCCASR.Y + Acetyl (Protein N-term); Oxidation (M) | | 1019.4889 | 1018.4816 | 1018.5084 | -26.27 | 67 | - | 75 | 0 | --- | K.TSVHDFSVK.D | | 1019.4889 | 1018.4816 | 1018.5084 | -26.27 | 67 | - | 75 | 0 | 28 | K.TSVHDFSVK.D | | 1137.5452 | 1136.5379 | 1136.5713 | -29.41 | 79 | - | 88 | 0 | --- | K.GQDVDLSIYK.G | | 1137.5452 | 1136.5379 | 1136.5713 | -29.41 | 79 | - | 88 | 0 | 48 | K.GQDVDLSIYK.G | | 1241.6169 | 1240.6096 | 1240.6492 | -31.90 | 152 | - | 161 | 1 | --- | R.FKAEFPIFDK.V | | 1241.6169 | 1240.6096 | 1240.6492 | -31.90 | 152 | - | 161 | 1 | 50 | R.FKAEFPIFDK.V | | 1306.6473 | 1305.6400 | 1305.6816 | -31.85 | 207 | - | 218 | 0 | --- | R.YAPTTSPLSIEK.D | | 1306.6473 | 1305.6400 | 1305.6816 | -31.85 | 207 | - | 218 | 0 | 54 | R.YAPTTSPLSIEK.D | | 1375.6494 | 1374.6421 | 1374.6779 | -26.05 | 162 | - | 174 | 0 | --- | K.VDVNGDNAAPLYK.H | | 1404.6952 | 1403.6879 | 1403.7409 | -37.72 | 195 | - | 206 | 1 | --- | K.FLVDKEGNVVER.Y | | 1404.6952 | 1403.6879 | 1403.7409 | -37.72 | 195 | - | 206 | 1 | 100 | K.FLVDKEGNVVER.Y | | 2323.0938 | 2322.0865 | 2322.1532 | -28.71 | 154 | - | 174 | 1 | --- | K.AEFPIFDKVDVNGDNAAPLYK.H | | 2323.0938 | 2322.0865 | 2322.1532 | -28.71 | 154 | - | 174 | 1 | 135 | K.AEFPIFDKVDVNGDNAAPLYK.H |  | **No match to:** 700.3804, 705.3198, 713.3984, 716.3349, 720.3130, 731.3079, 732.2952, 734.4708, 736.3295, 742.3474, 746.3737, 768.5091, 784.3730, 798.3884, 813.3755, 816.3912, 827.3756, 832.2836, 832.2836, 834.4414, 842.4781, 855.0237, 857.3641, 859.3939, 861.0433, 864.4484, 868.5302, 882.5316, 890.4687, 915.4315, 968.4974, 980.4708, 982.5235, 996.5598, 1006.5193, 1033.4980, 1033.4980, 1045.5164, 1051.6783, 1066.0463, 1119.6127, 1151.6914, 1264.6084, 1320.6482, 1328.6277, 1356.6017, 1386.6991, 1386.6991, 1387.6896, 1395.6013, 1396.5997, 1402.6963, 1403.6761, 1416.6887, 1417.6857, 1418.7150, 1418.7150, 1426.6853, 1455.7083, 1459.6730, 1459.6730, 1510.6388, 1557.8002, 1571.7368, 2246.1667, 2337.1011, 2345.0557, 2598.2500, 2598.2500, 2696.1882, 3591.5815, 3593.4373, 3682.5308 | | --- |   **Chaperones and folding catalysts**   | **S16.** | [Cs5g01840.2](http://localhost/mascot/cgi/protein_view.pl?file=../data/20140808/F003844.dat&hit=1)    **Mass:** 55560    **Score:** 578    **Expect:** 7e-054  **Matches:** 27 | | --- | --- |  | **Observed** | **Mr(expt)** | **Mr(calc)** | **ppm** | **Start** |  | **End** | **Miss** | **Ions** | **Peptide** | | --- | --- | --- | --- | --- | --- | --- | --- | --- | --- | | 707.3869 | 706.3796 | 706.3221 | 81.5 | 1 | - | 6 | 0 | --- | -.MAGSWR.A | | 722.4022 | 721.3949 | 721.4010 | -8.46 | 98 | - | 103 | 0 | --- | R.TIFDVK.R | | 728.4547 | 727.4474 | 727.4592 | -16.19 | 378 | - | 383 | 0 | --- | K.VQQLLK.D | | 776.4452 | 775.4380 | 775.4262 | 15.2 | 345 | - | 351 | 1 | --- | K.TMGPVKK.A + Oxidation (M) | | 784.5247 | 783.5174 | 783.4239 | 119 | 281 | - | 287 | 1 | --- | K.HGKDISK.D | | 945.4904 | 944.4831 | 944.4677 | 16.3 | 269 | - | 275 | 0 | --- | R.VMEYFIK.L + Oxidation (M) | | 1025.5375 | 1024.5302 | 1024.5414 | -10.89 | 305 | - | 313 | 0 | --- | R.ALSSQHQVR.V | | 1107.5408 | 1106.5335 | 1106.5277 | 5.23 | 351 | - | 360 | 1 | --- | K.KAMEDAGLEK.N + Oxidation (M) | | 1169.6193 | 1168.6120 | 1168.6241 | -10.29 | 491 | - | 501 | 0 | --- | K.FDLSGIPPAPR.F | | 1169.6193 | 1168.6120 | 1168.6241 | -10.29 | 491 | - | 501 | 0 | 84 | K.FDLSGIPPAPR.F | | 1296.6096 | 1295.6023 | 1295.6146 | -9.47 | 334 | - | 343 | 0 | --- | R.FEELNNDLFR.K | | 1296.6096 | 1295.6023 | 1295.6146 | -9.47 | 334 | - | 343 | 0 | 35 | R.FEELNNDLFR.K | | 1316.7122 | 1315.7049 | 1315.7248 | -15.12 | 130 | - | 140 | 0 | --- | K.DGKPYIQVQIR.D | | 1316.7122 | 1315.7049 | 1315.7248 | -15.12 | 130 | - | 140 | 0 | 56 | K.DGKPYIQVQIR.D | | 1500.7734 | 1499.7661 | 1499.7944 | -18.84 | 361 | - | 374 | 0 | --- | K.NQIDEIVLVGGSTR.I | | 1500.7734 | 1499.7661 | 1499.7944 | -18.84 | 361 | - | 374 | 0 | 84 | K.NQIDEIVLVGGSTR.I | | 1523.7338 | 1522.7265 | 1522.7528 | -17.26 | 332 | - | 343 | 1 | --- | R.ARFEELNNDLFR.K | | 1523.7338 | 1522.7265 | 1522.7528 | -17.26 | 332 | - | 343 | 1 | 44 | R.ARFEELNNDLFR.K | | 1536.7383 | 1535.7310 | 1535.7440 | -8.48 | 55 | - | 68 | 0 | --- | K.NGHVEIIANDQGNR.I | | 1665.7941 | 1664.7868 | 1664.8158 | -17.43 | 174 | - | 188 | 0 | --- | K.DAVVTVPAYFNDAQR.Q | | 1665.7941 | 1664.7868 | 1664.8158 | -17.43 | 174 | - | 188 | 0 | 30 | K.DAVVTVPAYFNDAQR.Q | | 1787.9564 | 1786.9491 | 1786.9828 | -18.87 | 205 | - | 221 | 1 | --- | R.IINEPTAAAIAYGLDKK.G | | 1906.9675 | 1905.9602 | 1905.9949 | -18.17 | 172 | - | 188 | 1 | --- | K.IKDAVVTVPAYFNDAQR.Q | | 1906.9675 | 1905.9602 | 1905.9949 | -18.17 | 172 | - | 188 | 1 | 73 | K.IKDAVVTVPAYFNDAQR.Q | | 2066.9907 | 2065.9834 | 2066.0208 | -18.09 | 314 | - | 331 | 0 | --- | R.VEIESLFDGIDFSEPLTR.A | | 2066.9907 | 2065.9834 | 2066.0208 | -18.09 | 314 | - | 331 | 0 | 112 | R.VEIESLFDGIDFSEPLTR.A | | 2691.2371 | 2690.2298 | 2690.2824 | -19.55 | 457 | - | 479 | 0 | --- | K.SQVFTTYQDQQTTVSIQVFEGER.S |  | **No match to:** 700.3955, 711.3785, 713.4099, 716.3980, 719.3725, 721.3998, 724.4973, 727.4039, 733.3843, 734.4809, 750.4926, 752.4197, 758.3791, 768.5199, 786.4155, 810.4629, 811.4599, 824.4622, 832.3068, 832.3068, 834.4963, 842.4897, 850.5221, 856.4968, 864.4794, 868.5408, 881.2515, 881.2515, 901.4830, 912.4796, 914.4279, 967.4650, 968.5428, 974.5356, 982.5507, 995.6141, 1017.5894, 1019.5829, 1021.5275, 1045.5551, 1051.6831, 1081.5310, 1133.6654, 1151.7126, 1164.5980, 1187.6458, 1197.6427, 1197.6427, 1211.6471, 1228.6117, 1231.6230, 1237.6163, 1252.7047, 1261.6644, 1294.6200, 1310.6160, 1320.6383, 1329.6084, 1374.6047, 1416.7633, 1426.7388, 1436.7314, 1450.7131, 1457.6938, 1465.7303, 1473.6633, 1473.6633, 1487.6960, 1512.7185, 1527.7676, 1535.7458, 1535.7458, 1553.7180, 1556.7300, 1556.7300, 1581.8058, 1620.7994, 1624.8877, 1651.8206, 1675.7014, 1680.7999, 1680.7999, 1684.8173, 1821.8752, 2081.0117, 2246.1636, 2658.2327, 2692.2397 | | --- |  | **S8.** | [Cs1g06710.1](http://localhost/mascot/cgi/protein_view.pl?file=../data/20140415/F003310.dat&hit=1)    **Mass:** 50360    **Score:** 109    **Expect:** 5.6e-007  **Matches:** 5 | | --- | --- |  | **Observed** | **Mr(expt)** | **Mr(calc)** | **ppm** | **Start** |  | **End** | **Miss** | **Ions** | **Peptide** | | --- | --- | --- | --- | --- | --- | --- | --- | --- | --- | | 1324.6036 | 1323.5963 | 1323.6646 | -51.54 | 71 | - | 81 | 0 | --- | K.LFISTFQHGMK.T + Oxidation (M) | | 1324.6036 | 1323.5963 | 1323.6646 | -51.54 | 71 | - | 81 | 0 | --- | K.LFISTFQHGMK.T + Oxidation (M) | | 1575.8121 | 1574.8048 | 1574.8668 | -39.34 | 144 | - | 157 | 0 | --- | K.AIQASLEDISFLLR.I | | 1575.8121 | 1574.8048 | 1574.8668 | -39.34 | 144 | - | 157 | 0 | 92 | K.AIQASLEDISFLLR.I | | 2479.0950 | 2478.0877 | 2478.1816 | -37.88 | 407 | - | 430 | 0 | --- | R.NAGLGGLSFDEGQFSVFGYTTVGR.D |  | **No match to:** 772.5360, 816.5731, 860.5997, 904.6336, 1787.8101, 1787.8101, 2019.8898, 2019.8898, 2152.0325, 2152.0325, 2200.0393, 2200.0393, 2216.0215, 2216.0215, 2288.0203, 2686.2402, 2691.2468, 2691.2468, 2749.3650, 2749.3650 | | --- |  | **S26.** | [orange1.1t01459.2](http://localhost/mascot/cgi/protein_view.pl?file=../data/20140808/F003854.dat&hit=1)    **Mass:** 58188    **Score:** 727    **Expect:** 8.8e-069  **Matches:** 39 | | --- | --- |  | **Observed** | **Mr(expt)** | **Mr(calc)** | **ppm** | **Start** |  | **End** | **Miss** | **Ions** | **Peptide** | | --- | --- | --- | --- | --- | --- | --- | --- | --- | --- | | 801.4143 | 800.4070 | 800.4756 | -85.64 | 99 | - | 106 | 0 | --- | K.NIGASLVK.Q | | 842.4783 | 841.4710 | 841.4909 | -23.66 | 491 | - | 498 | 0 | --- | K.SGIIDPLK.V | | 847.3765 | 846.3692 | 846.3984 | -34.53 | 309 | - | 316 | 0 | --- | K.APGFGENR.K | | 847.3765 | 846.3692 | 846.3984 | -34.53 | 309 | - | 316 | 0 | 58 | K.APGFGENR.K | | 861.3919 | 860.3846 | 860.4538 | -80.41 | 60 | - | 67 | 1 | --- | K.VTMGPKGR.N + Oxidation (M) | | 861.3919 | 860.3846 | 860.4538 | -80.41 | 60 | - | 67 | 1 | --- | K.VTMGPKGR.N + Oxidation (M) | | 868.5195 | 867.5123 | 867.4160 | 111 | 129 | - | 136 | 0 | --- | R.AIFTEGCK.S | | 941.5823 | 940.5750 | 940.6069 | -33.93 | 267 | - | 274 | 1 | --- | R.VLELALKR.Q | | 956.4148 | 955.4075 | 955.4321 | -25.72 | 483 | - | 490 | 0 | --- | K.GEYVDMVK.S + Oxidation (M) | | 972.5513 | 971.5440 | 971.5764 | -33.32 | 258 | - | 266 | 0 | --- | K.ISNLTAVVR.V | | 972.5513 | 971.5440 | 971.5764 | -33.32 | 258 | - | 266 | 0 | 61 | K.ISNLTAVVR.V | | 975.4794 | 974.4721 | 974.4934 | -21.82 | 309 | - | 317 | 1 | --- | K.APGFGENRK.A | | 1023.4813 | 1022.4740 | 1022.5033 | -28.60 | 428 | - | 436 | 0 | --- | K.LSTANFDQK.I | | 1030.5027 | 1029.4954 | 1029.5342 | -37.69 | 50 | - | 59 | 0 | --- | K.GVEELADAVK.V | | 1100.6405 | 1099.6332 | 1099.6713 | -34.65 | 257 | - | 266 | 1 | --- | K.KISNLTAVVR.V | | 1196.6932 | 1195.6859 | 1195.7288 | -35.89 | 437 | - | 447 | 0 | --- | K.IGVQIIQNALK.T | | 1196.6932 | 1195.6859 | 1195.7288 | -35.89 | 437 | - | 447 | 0 | 48 | K.IGVQIIQNALK.T | | 1203.5596 | 1202.5523 | 1202.6255 | -60.85 | 393 | - | 403 | 1 | --- | K.DRVTDALNATK.A | | 1218.6238 | 1217.6165 | 1217.5776 | 32.0 | 359 | - | 370 | 0 | --- | K.DDTVILDGAGDK.K | | 1267.5370 | 1266.5297 | 1266.5696 | -31.52 | 137 | - | 148 | 0 | --- | K.SVAAGMNAMDLR.R + 2 Oxidation (M) | | 1267.5370 | 1266.5297 | 1266.5696 | -31.52 | 137 | - | 148 | 0 | 11 | K.SVAAGMNAMDLR.R + 2 Oxidation (M) | | 1327.6489 | 1326.6416 | 1326.6932 | -38.87 | 68 | - | 79 | 0 | --- | R.NVVIEQSWGAPK.V | | 1331.6490 | 1330.6417 | 1330.7166 | -56.25 | 188 | - | 199 | 1 | --- | R.EIGELIAKAMEK.V | | 1430.6849 | 1429.6776 | 1429.7242 | -32.54 | 229 | - | 240 | 0 | --- | R.GYISPYFITNQK.N | | 1430.6849 | 1429.6776 | 1429.7242 | -32.54 | 229 | - | 240 | 0 | 58 | R.GYISPYFITNQK.N | | 1447.7297 | 1446.7224 | 1446.7752 | -36.49 | 150 | - | 163 | 0 | --- | R.GITMAVDAVVTNLK.S + Oxidation (M) | | 1540.7120 | 1539.7047 | 1539.7490 | -28.78 | 213 | - | 225 | 0 | --- | K.TLYNELEVVEGMK.L + Oxidation (M) | | 1540.7120 | 1539.7047 | 1539.7490 | -28.78 | 213 | - | 225 | 0 | 31 | K.TLYNELEVVEGMK.L + Oxidation (M) | | 1586.6752 | 1585.6679 | 1585.8749 | -130.51 | 45 | - | 59 | 1 | --- | R.ALMLKGVEELADAVK.V | | 1603.8201 | 1602.8128 | 1602.8763 | -39.62 | 149 | - | 163 | 1 | --- | R.RGITMAVDAVVTNLK.S + Oxidation (M) | | 1665.7477 | 1664.7404 | 1664.7893 | -29.37 | 468 | - | 482 | 0 | --- | K.LLEQDNTDLGYDAAK.G | | 1884.9824 | 1883.9751 | 1884.0356 | -32.12 | 404 | - | 423 | 0 | --- | K.AAVEEGIVPGGGVALLYAAK.E | | 1889.9852 | 1888.9779 | 1889.0371 | -31.31 | 448 | - | 467 | 0 | --- | K.TPVHTIAANAGVEGAVVVGK.L | | 1889.9852 | 1888.9779 | 1889.0371 | -31.31 | 448 | - | 467 | 0 | 139 | K.TPVHTIAANAGVEGAVVVGK.L | | 1924.9119 | 1923.9046 | 1923.9612 | -29.38 | 213 | - | 228 | 1 | --- | K.TLYNELEVVEGMKLDR.G + Oxidation (M) | | 1924.9119 | 1923.9046 | 1923.9612 | -29.38 | 213 | - | 228 | 1 | 20 | K.TLYNELEVVEGMKLDR.G + Oxidation (M) | | 2108.9497 | 2107.9424 | 2108.0055 | -29.94 | 168 | - | 187 | 0 | --- | R.MISTSEEIAQVGTISANGER.E + Oxidation (M) | | 2108.9497 | 2107.9424 | 2108.0055 | -29.94 | 168 | - | 187 | 0 | 152 | R.MISTSEEIAQVGTISANGER.E + Oxidation (M) | | 2535.3389 | 2534.3316 | 2534.4319 | -39.57 | 275 | - | 297 | 0 | --- | R.QRPLLIVAEDVESEALATLILNK.L |  | **No match to:** 700.3068, 702.3151, 704.2819, 705.3039, 713.3640, 719.3284, 721.3345, 727.3564, 733.3351, 735.3483, 744.3569, 747.3509, 750.3611, 758.3648, 763.3760, 768.5076, 779.3705, 829.3755, 830.3752, 832.3596, 834.4051, 854.4143, 864.4183, 883.4559, 885.4078, 892.4159, 910.4171, 914.4008, 925.4205, 945.4883, 947.4374, 962.4909, 1013.5077, 1021.4856, 1047.5038, 1116.5690, 1175.5302, 1187.6183, 1202.5400, 1207.5947, 1233.5913, 1261.6604, 1324.5991, 1343.6528, 1452.6671, 1465.7053, 1479.7142, 1577.7356, 1591.7455, 1593.7614, 1594.7637, 1608.7694, 1626.8199, 1626.8199, 1640.8334, 1714.8977, 1733.8167, 1781.7643, 1795.8323, 1860.9003, 1906.9541, 1911.9541, 1938.8772, 2044.0120, 2044.9508, 2044.9508, 2058.9558, 2091.9453, 2122.9651, 2217.9685, 2217.9685, 2234.9985, 2234.9985, 2246.1343, 2518.3142, 2518.3142 | | --- |   **Signal transduction**   | **S35.** | [Cs9g03630.1](http://localhost/mascot/cgi/protein_view.pl?file=../data/20140808/F003860.dat&hit=1)    **Mass:** 17632    **Score:** 230    **Expect:** 4.4e-019  **Matches:** 15 | | --- | --- |  | **Observed** | **Mr(expt)** | **Mr(calc)** | **ppm** | **Start** |  | **End** | **Miss** | **Ions** | **Peptide** | | --- | --- | --- | --- | --- | --- | --- | --- | --- | --- | | 750.4710 | 749.4637 | 749.3973 | 88.7 | 67 | - | 71 | 1 | --- | K.YFKHR.V | | 782.4954 | 781.4881 | 781.5062 | -23.08 | 34 | - | 40 | 0 | --- | K.VLPQVVK.N | | 916.4655 | 915.4582 | 915.4662 | -8.68 | 107 | - | 116 | 0 | --- | K.VVGTPDGGSK.S | | 953.4998 | 952.4925 | 952.5090 | -17.30 | 70 | - | 77 | 1 | --- | K.HRVDALDK.E | | 1149.6027 | 1148.5954 | 1148.6230 | -23.98 | 123 | - | 132 | 0 | --- | K.FYPKPGAEIK.E | | 1149.6027 | 1148.5954 | 1148.6230 | -23.98 | 123 | - | 132 | 0 | 26 | K.FYPKPGAEIK.E | | 1212.5430 | 1211.5357 | 1211.5611 | -20.97 | 57 | - | 66 | 0 | --- | K.FNFVEGADWK.Y | | 1442.7856 | 1441.7783 | 1441.7413 | 25.7 | 41 | - | 55 | 0 | --- | K.NVELISGDGGPGSIK.K | | 1442.7856 | 1441.7783 | 1441.7413 | 25.7 | 41 | - | 55 | 0 | 23 | K.NVELISGDGGPGSIK.K | | 1570.8098 | 1569.8025 | 1569.8362 | -21.47 | 41 | - | 56 | 1 | --- | K.NVELISGDGGPGSIKK.F | | 1761.8817 | 1760.8744 | 1760.9461 | -40.69 | 145 | - | 161 | 1 | --- | K.GIFKALEAYALANPNAV.- | | 1762.8907 | 1761.8834 | 1761.9301 | -26.49 | 123 | - | 137 | 1 | --- | K.FYPKPGAEIKEEQVK.G | | 1762.8907 | 1761.8834 | 1761.9301 | -26.49 | 123 | - | 137 | 1 | 94 | K.FYPKPGAEIKEEQVK.G | | 1776.9386 | 1775.9313 | 1776.0185 | -49.11 | 117 | - | 132 | 1 | --- | K.STVVIKFYPKPGAEIK.E | | 1812.9183 | 1811.9110 | 1811.9517 | -22.43 | 2 | - | 18 | 0 | --- | M.GVLTLNVEDTSTLPPEK.L |  | **No match to:** 700.3887, 702.3738, 704.3697, 708.3658, 709.3605, 713.4095, 724.4935, 726.4191, 727.3941, 728.4406, 729.3942, 732.3767, 734.4747, 736.4730, 745.4130, 768.5178, 784.5141, 799.4058, 824.4692, 829.2192, 834.4809, 842.4834, 850.5200, 856.5070, 864.4755, 868.5321, 881.2473, 881.2473, 882.5442, 899.2537, 917.2684, 968.5454, 973.5141, 982.5478, 995.6061, 996.5884, 1012.5485, 1033.5565, 1045.5421, 1051.6842, 1140.5558, 1148.5896, 1163.6173, 1165.6603, 1171.5975, 1179.5725, 1210.5344, 1216.5411, 1216.5411, 1227.5852, 1228.5475, 1228.5475, 1277.6796, 1292.6389, 1320.6165, 1323.6382, 1324.6616, 1324.6616, 1338.6338, 1338.6338, 1344.6389, 1354.6221, 1356.6285, 1392.6038, 1424.7368, 1441.7524, 1456.7393, 1464.7694, 1477.7461, 1480.7369, 1520.6949, 1546.7800, 1546.7800, 1563.7554, 1564.7806, 1564.7806, 1584.7985, 1606.7615, 1606.7615, 1699.7927, 1784.8765, 1798.9077, 1798.9077, 1820.8785, 1901.9341, 1919.9357, 1919.9357, 1933.9525, 1938.8541, 1938.8541, 1952.8749, 1993.9399, 2246.1758, 2380.0762, 2696.2205, 2752.2505, 2895.3386, 2913.3401, 2913.3401, 2927.3511 | | --- |  | **S7.** | [orange1.1t01991.1](http://localhost/mascot/cgi/protein_view.pl?file=../data/20140808/F003838.dat&hit=1)    **Mass:** 29424    **Score:** 439    **Expect:** 5.6e-040  **Matches:** 18 | | --- | --- |  | **Observed** | **Mr(expt)** | **Mr(calc)** | **ppm** | **Start** |  | **End** | **Miss** | **Ions** | **Peptide** | | --- | --- | --- | --- | --- | --- | --- | --- | --- | --- | | 705.3352 | 704.3279 | 704.3453 | -24.66 | 143 | - | 148 | 1 | --- | K.TGDERK.V | | 770.4009 | 769.3936 | 769.4010 | -9.63 | 137 | - | 142 | 0 | --- | R.YLAEFK.T | | 816.4093 | 815.4020 | 815.4137 | -14.31 | 16 | - | 22 | 0 | --- | K.LAEQAER.Y | | 816.4093 | 815.4020 | 815.4137 | -14.31 | 16 | - | 22 | 0 | 23 | K.LAEQAER.Y | | 922.5898 | 921.5825 | 921.4127 | 184 | 130 | - | 136 | 1 | --- | K.MKGDYHR.Y + Oxidation (M) | | 1096.5574 | 1095.5501 | 1095.5673 | -15.66 | 80 | - | 89 | 0 | --- | R.GNQDHVSVIK.E | | 1189.6366 | 1188.6293 | 1188.6536 | -20.44 | 222 | - | 231 | 0 | --- | K.DSTLIMQLLR.D | | 1205.6361 | 1204.6288 | 1204.6485 | -16.37 | 222 | - | 231 | 0 | --- | K.DSTLIMQLLR.D + Oxidation (M) | | 1205.6361 | 1204.6288 | 1204.6485 | -16.37 | 222 | - | 231 | 0 | 30 | K.DSTLIMQLLR.D + Oxidation (M) | | 1328.6580 | 1327.6507 | 1327.6408 | 7.47 | 137 | - | 147 | 1 | --- | R.YLAEFKTGDER.K | | 1404.7098 | 1403.7025 | 1403.7256 | -16.43 | 68 | - | 79 | 1 | --- | R.IVSSIEQKEESR.G | | 1676.7969 | 1675.7896 | 1675.8264 | -21.97 | 33 | - | 48 | 0 | --- | K.VVASASTGEELTVEER.N | | 1676.7969 | 1675.7896 | 1675.8264 | -21.97 | 33 | - | 48 | 0 | 110 | K.VVASASTGEELTVEER.N | | 1703.6851 | 1702.6778 | 1702.9175 | -140.73 | 93 | - | 108 | 1 | --- | R.SKIEAELTEICGGILK.L | | 1803.8918 | 1802.8845 | 1802.9275 | -23.82 | 160 | - | 176 | 0 | --- | K.SAQDIANAELAPTHPIR.L | | 1803.8918 | 1802.8845 | 1802.9275 | -23.82 | 160 | - | 176 | 0 | 137 | K.SAQDIANAELAPTHPIR.L | | 2331.1541 | 2330.1468 | 2330.1947 | -20.53 | 177 | - | 196 | 0 | --- | R.LGLALNFSVFYYEILNSPDR.A | | 2331.1541 | 2330.1468 | 2330.1947 | -20.53 | 177 | - | 196 | 0 | 77 | R.LGLALNFSVFYYEILNSPDR.A |  | **No match to:** 700.3677, 711.3789, 713.4171, 725.4287, 734.4887, 736.4627, 768.5207, 782.4695, 795.5298, 830.4186, 832.2977, 834.4793, 838.5365, 842.4933, 850.5241, 868.5364, 882.5476, 982.5790, 1025.5834, 1026.5281, 1051.6824, 1058.5850, 1076.5970, 1080.5928, 1083.5367, 1085.6136, 1092.5912, 1112.5979, 1112.5979, 1138.6158, 1140.5863, 1140.5863, 1151.7090, 1154.6003, 1160.5903, 1256.6525, 1311.6072, 1324.6162, 1343.6124, 1359.6438, 1375.5997, 1394.6221, 1444.7213, 1494.8186, 1542.8613, 1554.7202, 1554.7202, 1566.7601, 1572.7256, 1572.7256, 1594.7131, 1616.7947, 1635.8497, 1690.8163, 1760.8954, 1802.9268, 1817.9122, 1855.8867, 1855.8867, 1868.8757, 1870.8926, 1872.9076, 1873.9050, 1874.8882, 1874.8882, 1884.9088, 1886.8917, 1886.8917, 1902.8947, 1910.9797, 1957.9991, 2013.0212, 2051.1641, 2103.0469, 2103.0469, 2103.8240, 2118.9990, 2120.0527, 2121.0554, 2121.0554, 2135.0732, 2143.0916, 2178.0737, 2393.1013, 2450.0505, 2464.1116, 2612.2380, 2703.3918, 2706.2974, 2735.3201, 2739.3328, 2750.2825, 2794.4099, 2794.4099, 2808.4197, 2922.4822, 3104.5264 | | --- |   **Cellular transport**   | **S5.** | [Cs6g09150.2](http://localhost/mascot/cgi/protein_view.pl?file=../data/20140415/F003306.dat&hit=1)    **Mass:** 28953    **Score:** 406    **Expect:** 1.1e-036  **Matches:** 13 | | --- | --- |  | **Observed** | **Mr(expt)** | **Mr(calc)** | **ppm** | **Start** |  | **End** | **Miss** | **Ions** | **Peptide** | | --- | --- | --- | --- | --- | --- | --- | --- | --- | --- | | 701.3674 | 700.3601 | 700.3868 | -38.08 | 123 | - | 128 | 0 | --- | R.DNIALR.G | | 765.3994 | 764.3921 | 764.4181 | -33.94 | 234 | - | 239 | 0 | --- | K.YVSQLR.M | | 1100.5048 | 1099.4975 | 1099.5120 | -13.20 | 148 | - | 155 | 0 | --- | K.FMEYQNLR.G | | 1116.4899 | 1115.4826 | 1115.5070 | -21.81 | 148 | - | 155 | 0 | --- | K.FMEYQNLR.G + Oxidation (M) | | 1116.4899 | 1115.4826 | 1115.5070 | -21.81 | 148 | - | 155 | 0 | 30 | K.FMEYQNLR.G + Oxidation (M) | | 1459.6050 | 1458.5977 | 1458.6222 | -16.80 | 136 | - | 147 | 1 | --- | K.ESSEEEREHAEK.F | | 1459.6050 | 1458.5977 | 1458.6222 | -16.80 | 136 | - | 147 | 1 | 72 | K.ESSEEEREHAEK.F | | 1607.9041 | 1606.8968 | 1606.9294 | -20.27 | 74 | - | 88 | 0 | --- | K.EVLDVPVSPLLSLAR.Q | | 1607.9041 | 1606.8968 | 1606.9294 | -20.27 | 74 | - | 88 | 0 | 129 | K.EVLDVPVSPLLSLAR.Q | | 1735.9946 | 1734.9873 | 1735.0244 | -21.34 | 73 | - | 88 | 1 | --- | K.KEVLDVPVSPLLSLAR.Q | | 1735.9946 | 1734.9873 | 1735.0244 | -21.34 | 73 | - | 88 | 1 | 81 | K.KEVLDVPVSPLLSLAR.Q | | 1881.8427 | 1880.8354 | 1880.8727 | -19.81 | 161 | - | 176 | 0 | --- | K.LHSIMQPPSEFDHAEK.G + Oxidation (M) | | 1881.8427 | 1880.8354 | 1880.8727 | -19.81 | 161 | - | 176 | 0 | 49 | K.LHSIMQPPSEFDHAEK.G + Oxidation (M) |  | **No match to:** 728.5421, 772.5666, 816.5870, 834.3486, 842.4631, 860.6061, 904.6379, 948.6697, 1018.4739, 1018.4739, 1051.1909, 1051.3446, 1051.6771, 1052.4879, 1052.4879, 1110.6011, 1110.6011, 1130.5038, 1132.4939, 1155.5293, 1155.5293, 1417.6711, 1516.7202, 1516.7202, 1589.8898, 1589.8898, 1621.8971, 1700.8304, 1700.8304, 1817.8436, 1990.8796, 2039.8926, 2039.8926, 2053.8840, 2053.8840, 2422.1841, 2422.1841 | | --- |   **Nucleic acid metabolism**   | **S42.** | [Cs6g16920.1](http://localhost/mascot/cgi/protein_view.pl?file=../data/20140415/F003349.dat&hit=1)    **Mass:** 50194    **Score:** 268    **Expect:** 7e-023  **Matches:** 14 | | --- | --- |  | **Observed** | **Mr(expt)** | **Mr(calc)** | **ppm** | **Start** |  | **End** | **Miss** | **Ions** | **Peptide** | | --- | --- | --- | --- | --- | --- | --- | --- | --- | --- | | 1168.5161 | 1167.5088 | 1167.5594 | -43.31 | 112 | - | 120 | 0 | --- | K.TEILMENFR.R + Oxidation (M) | | 1261.6526 | 1260.6453 | 1260.6965 | -40.62 | 175 | - | 185 | 0 | --- | K.LDPTIYDALIK.E | | 1261.6526 | 1260.6453 | 1260.6965 | -40.62 | 175 | - | 185 | 0 | --- | K.LDPTIYDALIK.E | | 1296.6095 | 1295.6022 | 1295.6543 | -40.23 | 111 | - | 120 | 1 | --- | K.KTEILMENFR.R + Oxidation (M) | | 1324.5774 | 1323.5701 | 1323.6605 | -68.27 | 112 | - | 121 | 1 | --- | K.TEILMENFRR.A + Oxidation (M) | | 1717.8815 | 1716.8742 | 1716.9410 | -38.90 | 321 | - | 336 | 0 | --- | R.ALESSLSPIVIFATNR.G | | 1717.8815 | 1716.8742 | 1716.9410 | -38.90 | 321 | - | 336 | 0 | 100 | R.ALESSLSPIVIFATNR.G | | 1832.9315 | 1831.9242 | 1831.9866 | -34.03 | 365 | - | 380 | 0 | --- | R.TQIYGPAEMIQILAIR.A + Oxidation (M) | | 1832.9315 | 1831.9242 | 1831.9866 | -34.03 | 365 | - | 380 | 0 | 29 | R.TQIYGPAEMIQILAIR.A + Oxidation (M) | | 1866.8381 | 1865.8308 | 1865.8942 | -33.95 | 343 | - | 359 | 0 | --- | R.GTDMNSPHGIPLDLLDR.L + Oxidation (M) | | 1866.8381 | 1865.8308 | 1865.8942 | -33.95 | 343 | - | 359 | 0 | 26 | R.GTDMNSPHGIPLDLLDR.L + Oxidation (M) | | 2310.1211 | 2309.1138 | 2309.2128 | -42.85 | 26 | - | 49 | 0 | --- | K.GLGLEANGNAVPLAAGFVGQVEAR.E | | 2310.1211 | 2309.1138 | 2309.2128 | -42.85 | 26 | - | 49 | 0 | 74 | K.GLGLEANGNAVPLAAGFVGQVEAR.E | | 2321.1277 | 2320.1204 | 2320.1910 | -30.42 | 381 | - | 401 | 0 | --- | R.AQVEEIVLDEESLAHLGEIAR.D |  | **No match to:** 728.5264, 772.5492, 816.5693, 823.4315, 842.4655, 860.6013, 904.6193, 910.3984, 914.3620, 948.6549, 978.4632, 992.6786, 1013.4919, 1021.4789, 1021.4789, 1036.7004, 1080.7238, 1116.5420, 1128.4827, 1170.5690, 1170.5690, 1187.6127, 1187.6127, 1277.6316, 1300.6249, 1303.6406, 1363.6187, 1447.7029, 1465.6931, 1465.6931, 1479.7126, 1481.6931, 1562.7400, 1562.7400, 1601.7897, 1619.7959, 1690.8278, 1690.8278, 1768.7623, 1803.8684, 1804.8734, 1804.8734, 1880.8853, 1941.9747, 1941.9747, 2069.9514, 2069.9514, 2121.9573, 2185.9314, 2185.9314, 2219.0928 | | --- |   **Others**   | **S27.** | [orange1.1t05091.1](http://localhost/mascot/cgi/protein_view.pl?file=../data/20140808/F003855.dat&hit=1)    **Mass:** 17526    **Score:** 246    **Expect:** 1.1e-020  **Matches:** 19 | | --- | --- |  | **Observed** | **Mr(expt)** | **Mr(calc)** | **ppm** | **Start** |  | **End** | **Miss** | **Ions** | **Peptide** | | --- | --- | --- | --- | --- | --- | --- | --- | --- | --- | | **861.3484** | 860.3412 | 860.3777 | -42.40 | 55 | - | 61 | 0 | --- | R.ANEDAWR.G | | **862.4047** | 861.3974 | 861.4596 | -72.23 | 20 | - | 27 | 0 | --- | R.YADPGVLK.N | | **893.3354** | 892.3281 | 892.3895 | -68.77 | 1 | - | 7 | 1 | --- | -.MACDQRK.L + Acetyl (Protein N-term) | | **893.3354** | 892.3281 | 892.3895 | -68.77 | 1 | - | 7 | 1 | --- | -.MACDQRK.L + Acetyl (Protein N-term) | | **942.4092** | 941.4020 | 941.4429 | -43.52 | 32 | - | 39 | 0 | --- | R.MDFGLFGR.S | | **942.4092** | 941.4020 | 941.4429 | -43.52 | 32 | - | 39 | 0 | --- | R.MDFGLFGR.S | | **947.4113** | 946.4040 | 946.4549 | -53.77 | 13 | - | 19 | 0 | --- | K.SDYFFLR.Y | | **947.4113** | 946.4040 | 946.4549 | -53.77 | 13 | - | 19 | 0 | 49 | K.SDYFFLR.Y | | **958.3996** | 957.3923 | 957.4379 | -47.55 | 32 | - | 39 | 0 | --- | R.MDFGLFGR.S + Oxidation (M) | | **962.5168** | 961.5095 | 961.5570 | -49.35 | 95 | - | 102 | 0 | --- | K.NRPIHGLR.T | | **962.5168** | 961.5095 | 961.5570 | -49.35 | 95 | - | 102 | 0 | 43 | K.NRPIHGLR.T | | **1100.5277** | 1099.5204 | 1099.5451 | -22.40 | 147 | - | 154 | 1 | --- | K.VYWRLYSN.- | | **1239.6095** | 1238.6022 | 1238.6217 | -15.69 | 103 | - | 112 | 0 | --- | R.TMVQLDELYK.D | | **1255.5815** | 1254.5742 | 1254.6166 | -33.76 | 103 | - | 112 | 0 | --- | R.TMVQLDELYK.D + Oxidation (M) | | **1505.7454** | 1504.7381 | 1504.8078 | -46.32 | 8 | - | 19 | 1 | --- | K.LIGFKSDYFFLR.Y | | **1510.6943** | 1509.6870 | 1509.7497 | -41.52 | 103 | - | 114 | 1 | --- | R.TMVQLDELYKDR.H | | **1526.6913** | 1525.6840 | 1525.7446 | -39.72 | 103 | - | 114 | 1 | --- | R.TMVQLDELYKDR.H + Oxidation (M) | | **1526.6913** | 1525.6840 | 1525.7446 | -39.72 | 103 | - | 114 | 1 | 73 | R.TMVQLDELYKDR.H + Oxidation (M) | | **3467.6802** | 3466.6729 | 3466.7481 | -21.69 | 115 | - | 146 | 0 | --- | R.HVQEGGSLVLQINLIEVPHGNVLDSDHTFDGK.V |  | **No match to:** 707.2522, 726.4236, 745.3483, 801.4645, 842.4749, 855.0199, 859.3357, 861.0366, 865.3433, 865.3433, 875.3475, 877.3417, 877.3417, 889.3475, 891.3491, 892.3621, 894.3810, 907.3557, 910.3752, 912.3743, 915.3660, 921.3610, 929.4089, 940.3983, 944.4056, 945.4099, 957.3632, 969.4064, 974.4028, 980.3931, 984.5041, 985.3853, 1000.4702, 1021.4231, 1023.4100, 1031.3806, 1041.4031, 1043.4047, 1081.4686, 1085.4640, 1097.4623, 1111.5148, 1113.4646, 1115.6370, 1118.5278, 1129.4781, 1134.4905, 1156.5851, 1167.5734, 1169.5499, 1171.6920, 1171.6920, 1183.5568, 1225.6066, 1225.6066, 1233.5302, 1261.5175, 1281.6600, 1462.2582, 1462.2582, 1462.6907, 1633.8470, 1731.8846, 1733.8767, 1735.8756, 1735.8756, 1743.8793, 1745.8719, 1747.8784, 1747.8784, 1751.8787, 1759.8715, 1761.8708, 1763.8700, 1763.8700, 1771.8763, 1775.8722, 1779.8711, 1785.8781, 1789.8794, 1791.8693, 1791.8693, 1807.8590, 1893.9260, 1901.9224, 2040.0706, 2094.0286, 2096.1223, 2096.1223, 2108.0977, 2124.1487, 2152.1824, 2253.1177, 3406.4766, 3409.3750, 3470.5837 | | --- |  | **S28.** | [orange1.1t05091.1](http://localhost/mascot/cgi/protein_view.pl?file=../data/20140808/F003856.dat&hit=1)    **Mass:** 17526    **Score:** 212    **Expect:** 2.8e-017  **Matches:** 16 | | --- | --- |  | **Observed** | **Mr(expt)** | **Mr(calc)** | **ppm** | **Start** |  | **End** | **Miss** | **Ions** | **Peptide** | | --- | --- | --- | --- | --- | --- | --- | --- | --- | --- | | **861.3673** | 860.3600 | 860.3777 | -20.48 | 55 | - | 61 | 0 | --- | R.ANEDAWR.G | | **862.4260** | 861.4188 | 861.4596 | -47.43 | 20 | - | 27 | 0 | --- | R.YADPGVLK.N | | **893.3653** | 892.3580 | 892.3895 | -35.25 | 1 | - | 7 | 1 | --- | -.MACDQRK.L + Acetyl (Protein N-term) | | **942.4232** | 941.4159 | 941.4429 | -28.67 | 32 | - | 39 | 0 | --- | R.MDFGLFGR.S | | **947.4211** | 946.4139 | 946.4549 | -43.32 | 13 | - | 19 | 0 | --- | K.SDYFFLR.Y | | **947.4211** | 946.4139 | 946.4549 | -43.32 | 13 | - | 19 | 0 | 51 | K.SDYFFLR.Y | | **958.4095** | 957.4022 | 957.4379 | -37.23 | 32 | - | 39 | 0 | --- | R.MDFGLFGR.S + Oxidation (M) | | **958.4095** | 957.4022 | 957.4379 | -37.23 | 32 | - | 39 | 0 | 17 | R.MDFGLFGR.S + Oxidation (M) | | **962.5284** | 961.5211 | 961.5570 | -37.29 | 95 | - | 102 | 0 | --- | K.NRPIHGLR.T | | **1100.5704** | 1099.5631 | 1099.5451 | 16.4 | 147 | - | 154 | 1 | --- | K.VYWRLYSN.- | | **1510.7089** | 1509.7016 | 1509.7497 | -31.85 | 103 | - | 114 | 1 | --- | R.TMVQLDELYKDR.H | | **1510.7089** | 1509.7016 | 1509.7497 | -31.85 | 103 |  | 114 | 1 | (66) | R.TMVQLDELYKDR.H | | **1526.7036** | 1525.6963 | 1525.7446 | -31.66 | 103 | - | 114 | 1 | --- | R.TMVQLDELYKDR.H + Oxidation (M) | | **1526.7036** | 1525.6963 | 1525.7446 | -31.66 | 103 | - | 114 | 1 | 70 | R.TMVQLDELYKDR.H + Oxidation (M) | | **3442.5403** | 3441.5330 | 3441.5783 | -13.15 | 62 | - | 94 | 0 | --- | R.GDLPPAAANNMDAEASFDVVASGYTNGYRPIDK.N + Oxidation (M) | | **3467.6548** | 3466.6475 | 3466.7481 | -29.02 | 115 | - | 146 | 0 | --- | R.HVQEGGSLVLQINLIEVPHGNVLDSDHTFDGK.V |  | **No match to:** 713.3981, 719.3286, 724.4892, 725.4562, 731.3249, 733.3425, 745.3757, 768.5106, 783.3608, 785.3749, 791.3227, 793.3492, 803.3683, 811.3824, 832.2914, 832.2914, 842.4811, 848.4523, 859.3559, 864.4462, 865.3608, 865.3608, 868.5239, 875.3710, 876.3662, 877.3550, 877.3550, 889.3639, 891.3745, 892.3827, 894.4097, 894.4097, 910.4119, 912.3951, 914.4094, 917.3962, 929.4285, 930.4262, 932.4298, 940.4130, 944.4358, 945.4490, 969.4144, 974.4158, 974.4158, 980.3997, 984.5123, 985.5153, 987.4941, 1000.4761, 1009.4824, 1019.5333, 1023.4265, 1031.4146, 1041.4279, 1043.4286, 1045.4884, 1051.4425, 1051.6703, 1102.5372, 1111.5316, 1115.6512, 1118.5616, 1121.4425, 1134.5443, 1139.4419, 1139.4419, 1153.4661, 1156.6038, 1161.4651, 1167.5895, 1171.7079, 1175.4940, 1179.4697, 1179.4697, 1193.4950, 1226.5983, 1252.6162, 1335.6545, 1356.6122, 1456.6770, 1462.7080, 1462.7080, 1495.7076, 1499.7112, 1508.7097, 1511.7134, 1540.7234, 1542.7156, 1735.8936, 1735.8936, 1747.8998, 1747.8998, 1763.8937, 1837.7305, 2043.9866, 2043.9866, 3378.5583, 3620.7007 | | --- |  | **S31.** | [Cs7g01430.1](http://localhost/mascot/cgi/protein_view.pl?file=../data/20140415/F003340.dat&hit=1)    **Mass:** 28511    **Score:** 170    **Expect:** 4.4e-013  **Matches:** 15 | | --- | --- |  | **Observed** | **Mr(expt)** | **Mr(calc)** | **ppm** | **Start** |  | **End** | **Miss** | **Ions** | **Peptide** | | --- | --- | --- | --- | --- | --- | --- | --- | --- | --- | | 837.4497 | 836.4424 | 836.4280 | 17.3 | 111 | - | 117 | 0 | --- | K.AEVIYDK.Y | | 863.5005 | 862.4932 | 862.4912 | 2.29 | 184 | - | 191 | 0 | --- | K.VYVGNLAK.T | | 905.4488 | 904.4416 | 904.4187 | 25.3 | 125 | - | 131 | 0 | --- | R.FAFVMMK.T + 2 Oxidation (M) | | 916.4966 | 915.4894 | 915.4774 | 13.1 | 143 | - | 151 | 0 | --- | K.LNGTEIGGR.E | | 916.4966 | 915.4894 | 915.4774 | 13.1 | 143 | - | 151 | 0 | 42 | K.LNGTEIGGR.E | | 931.5438 | 930.5365 | 930.5287 | 8.44 | 84 | - | 91 | 0 | --- | R.VYIGNIPR.N | | 931.5438 | 930.5365 | 930.5287 | 8.44 | 84 | - | 91 | 0 | 45 | R.VYIGNIPR.N | | 1036.7485 | 1035.7412 | 1035.5634 | 172 | 192 | - | 200 | 1 | --- | K.TVTSEMLKK.C | | 1061.5387 | 1060.5314 | 1060.5198 | 11.0 | 124 | - | 131 | 1 | --- | R.RFAFVMMK.T + 2 Oxidation (M) | | 1074.5516 | 1073.5443 | 1073.5869 | -39.67 | 40 | - | 48 | 0 | --- | K.LSYSLHNLK.T | | 1087.6576 | 1086.6503 | 1086.6298 | 18.9 | 83 | - | 91 | 1 | --- | R.RVYIGNIPR.N | | 1087.6576 | 1086.6503 | 1086.6298 | 18.9 | 83 | - | 91 | 1 | 18 | R.RVYIGNIPR.N | | 1109.6011 | 1108.5938 | 1108.5877 | 5.56 | 101 | - | 110 | 0 | --- | K.IVQEHGAVEK.A | | 1314.6967 | 1313.6894 | 1313.6615 | 21.2 | 111 | - | 121 | 1 | --- | K.AEVIYDKYTGR.S | | 3249.6323 | 3248.6250 | 3248.5109 | 35.1 | 222 | - | 252 | 0 | --- | K.SSGFGFVTFSSEEDAEAAISSLNNSLLEGQR.I |  | **No match to:** 713.4226, 728.5684, 734.4871, 768.5219, 772.5919, 795.4400, 807.4171, 816.6149, 842.5145, 850.5170, 859.4440, 859.4440, 860.6423, 868.5516, 899.4663, 899.4663, 904.6667, 906.4659, 930.4790, 945.5294, 948.7047, 949.5102, 963.5297, 992.7281, 1013.5659, 1015.5495, 1039.5162, 1051.7106, 1112.6417, 1117.5707, 1126.6487, 1148.5950, 1161.6389, 1170.5931, 1183.6520, 1190.6149, 1198.6530, 1209.6600, 1225.6805, 1233.6586, 1236.6948, 1237.6858, 1238.7201, 1277.7007, 1277.7007, 1280.6733, 1356.6846, 1363.7319, 1368.7401, 1379.7156, 1409.7357, 1409.7357, 1411.7588, 1413.7399, 1422.7551, 1423.7500, 1428.7847, 1485.8438, 1491.7972, 1492.7747, 1506.7793, 1516.8284, 1516.8284, 1528.8024, 1528.8024, 1538.8138, 1542.8411, 1553.7906, 1566.8345, 1568.8462, 1569.8734, 1573.8352, 1574.8398, 1578.8624, 1600.7615, 1600.7615, 1606.8497, 1614.8589, 1633.8939, 1634.9127, 1699.8832, 1708.9050, 1801.9651, 1801.9651, 1807.9509, 1809.9612, 2023.0677, 2078.1252, 2144.0503, 2185.1821, 2215.0596, 2215.0596, 2229.0898, 2229.0898, 2235.0537, 2320.1584, 2320.1584, 2334.1658, 2334.1658, 3238.8096 | | --- |  | **S23.** | [Cs5g24940.1](http://localhost/mascot/cgi/protein_view.pl?file=../data/20140808/F003848.dat&hit=1)    **Mass:** 39452    **Score:** 300    **Expect:** 4.4e-026  **Matches:** 17 | | --- | --- |  | **Observed** | **Mr(expt)** | **Mr(calc)** | **ppm** | **Start** |  | **End** | **Miss** | **Ions** | **Peptide** | | --- | --- | --- | --- | --- | --- | --- | --- | --- | --- | | 1039.4907 | 1038.4834 | 1038.4957 | -11.82 | 128 | - | 135 | 0 | --- | K.VLMDGWFR.L + Oxidation (M) | | 1039.6630 | 1038.6557 | 1038.4957 | 154 | 128 | - | 135 | 0 | --- | K.VLMDGWFR.L + Oxidation (M) | | 1294.6699 | 1293.6626 | 1293.6969 | -26.47 | 220 | - | 230 | 0 | --- | K.GINYDLPYVIK.N | | 1396.5911 | 1395.5838 | 1395.8337 | -179.01 | 51 | - | 63 | 0 | --- | K.LSVSEIVAQIPLK.G | | 1396.7969 | 1395.7896 | 1395.8337 | -31.57 | 51 | - | 63 | 0 | --- | K.LSVSEIVAQIPLK.G | | 1403.6492 | 1402.6419 | 1402.6510 | -6.50 | 64 | - | 76 | 0 | --- | K.GNNPETAAMTLER.V | | 1419.6682 | 1418.6609 | 1418.6460 | 10.5 | 64 | - | 76 | 0 | --- | K.GNNPETAAMTLER.V + Oxidation (M) | | 1435.7109 | 1434.7036 | 1434.7507 | -32.81 | 184 | - | 195 | 1 | --- | K.VLESYKGFEHVK.K | | 1485.7605 | 1484.7532 | 1484.8027 | -33.34 | 97 | - | 109 | 0 | --- | R.LYSLAPVSAYFVR.N | | 1485.7605 | 1484.7532 | 1484.8027 | -33.34 | 97 | - | 109 | 0 | 101 | R.LYSLAPVSAYFVR.N | | 1593.8042 | 1592.7969 | 1592.8232 | -16.49 | 136 | - | 149 | 1 | --- | R.LKEQILEGGMAFNK.V + Oxidation (M) | | 1773.8865 | 1772.8792 | 1772.9706 | -51.55 | 197 | - | 214 | 0 | --- | K.LVDVGGGLGATLNMIISK.Y + Oxidation (M) | | 1823.6825 | 1822.6752 | 1822.6961 | -11.44 | 150 | - | 164 | 0 | --- | K.VHGMDMYDYMGVDSR.F + 3 Oxidation (M) | | 1885.8707 | 1884.8634 | 1885.0707 | -109.94 | 196 | - | 214 | 1 | --- | K.KLVDVGGGLGATLNMIISK.Y | | 2028.9678 | 2027.9605 | 2028.0276 | -33.07 | 286 | - | 304 | 0 | --- | K.LLNVNAAFPEVPENSATSR.E | | 2028.9678 | 2027.9605 | 2028.0276 | -33.07 | 286 | - | 304 | 0 | 146 | K.LLNVNAAFPEVPENSATSR.E | | 2174.9268 | 2173.9195 | 2173.9409 | -9.82 | 165 | - | 183 | 0 | --- | R.FNDVFNNGMSSHTSVVMEK.V + 2 Oxidation (M) |  | **No match to:** 713.4066, 758.4226, 768.5118, 842.4855, 868.5323, 871.4031, 919.4822, 938.4552, 1043.4836, 1055.4728, 1061.6476, 1070.5465, 1115.4913, 1161.5692, 1179.5844, 1218.6141, 1233.5732, 1234.5681, 1255.6145, 1256.6637, 1256.6637, 1260.6589, 1270.6724, 1272.6613, 1272.6613, 1286.6753, 1288.6593, 1316.6509, 1319.6141, 1321.6289, 1321.6289, 1328.6462, 1343.6375, 1351.6411, 1351.6411, 1364.6553, 1410.6698, 1410.6698, 1418.7238, 1426.7051, 1430.7247, 1432.6785, 1433.6843, 1448.6890, 1461.7255, 1465.7089, 1467.7345, 1475.7246, 1478.7786, 1484.7261, 1642.8250, 1680.8074, 1693.7933, 1739.8153, 1743.8347, 1755.8185, 1759.7306, 1772.8680, 1796.8973, 1811.9332, 1826.8833, 1827.8003, 1827.8003, 1841.8182, 1873.8701, 1875.8058, 1875.8058, 1882.9172, 1887.8953, 1889.8682, 1890.8749, 1891.7992, 1891.7992, 1905.8159, 1905.8159, 1913.8015, 1919.8696, 1945.9708, 1963.9968, 1963.9968, 1968.0057, 1976.8807, 1978.9135, 1978.9135, 1993.9070, 2039.0693, 2040.8861, 2042.9365, 2212.0127, 2228.9250, 2246.1533, 2319.9119, 2319.9119, 2333.9250, 2333.9250, 2652.1714, 3220.4834, 3221.4941 | | --- |  | **S37.** | [Orange1.1t01892.1](http://localhost/mascot/cgi/protein_view.pl?file=../data/20140808/F003862.dat&hit=1)    **Mass:** 53078    **Score:** 776    **Expect:** 1.1e-073  **Matches:** 24 | | --- | --- |  | **Observed** | **Mr(expt)** | **Mr(calc)** | **ppm** | **Start** |  | **End** | **Miss** | **Ions** | **Peptide** | | --- | --- | --- | --- | --- | --- | --- | --- | --- | --- | | 881.4013 | 880.3940 | 880.4178 | -27.03 | 153 | - | 159 | 0 | --- | K.AEEIYEK.T | | 1008.4401 | 1007.4328 | 1007.4672 | -34.15 | 18 | - | 26 | 0 | --- | K.DLSQADFGR.L | | 1008.4401 | 1007.4328 | 1007.4672 | -34.15 | 18 | - | 26 | 0 | 72 | K.DLSQADFGR.L | | 1025.4860 | 1024.4787 | 1024.5124 | -32.86 | 246 | - | 254 | 0 | --- | R.HSLPDGLMR.A | | 1041.4800 | 1040.4727 | 1040.5073 | -33.24 | 246 | - | 254 | 0 | --- | R.HSLPDGLMR.A + Oxidation (M) | | 1071.5790 | 1070.5717 | 1070.6084 | -34.26 | 369 | - | 377 | 0 | --- | R.ITIKPQTDR.W | | 1071.5790 | 1070.5717 | 1070.6084 | -34.26 | 369 | - | 377 | 0 | 53 | R.ITIKPQTDR.W | | 1107.5101 | 1106.5028 | 1106.5397 | -33.29 | 44 | - | 53 | 0 | --- | R.AEFGPSQPFK.G | | 1107.5101 | 1106.5028 | 1106.5397 | -33.29 | 44 | - | 53 | 0 | 54 | R.AEFGPSQPFK.G | | 1116.5493 | 1115.5420 | 1115.5869 | -40.25 | 276 | - | 287 | 1 | --- | K.GCAAALKQAGAR.V | | 1235.5948 | 1234.5875 | 1234.6306 | -34.89 | 16 | - | 26 | 1 | --- | K.VKDLSQADFGR.L | | 1235.5948 | 1234.5875 | 1234.6306 | -34.89 | 16 | - | 26 | 1 | 72 | K.VKDLSQADFGR.L | | 1319.6464 | 1318.6391 | 1318.6980 | -44.67 | 199 | - | 211 | 0 | --- | R.LVGVSEETTTGVK.R | | 1363.6370 | 1362.6297 | 1362.6932 | -46.58 | 44 | - | 56 | 1 | --- | R.AEFGPSQPFKGAK.I | | 1475.7471 | 1474.7398 | 1474.7991 | -40.21 | 199 | - | 212 | 1 | --- | R.LVGVSEETTTGVKR.L | | 1887.9347 | 1886.9274 | 1886.9890 | -32.66 | 378 | - | 394 | 0 | --- | R.WVFPETNSGIIVLAEGR.L | | 1887.9347 | 1886.9274 | 1886.9890 | -32.66 | 378 | - | 394 | 0 | 132 | R.WVFPETNSGIIVLAEGR.L | | 1903.9248 | 1902.9175 | 1902.9253 | -4.07 | 27 | - | 43 | 0 | --- | R.LEIELAEVEMPGLMACR.A | | 1903.9248 | 1902.9175 | 1902.9253 | -4.07 | 27 | - | 43 | 0 | 6 | R.LEIELAEVEMPGLMACR.A | | 1919.9136 | 1918.9063 | 1918.9202 | -7.23 | 27 | - | 43 | 0 | --- | R.LEIELAEVEMPGLMACR.A + Oxidation (M) | | 2213.0994 | 2212.0921 | 2212.1739 | -36.98 | 163 | - | 182 | 0 | --- | K.LPDPASTDNAEFQIVLTIIR.D | | 2213.0994 | 2212.0921 | 2212.1739 | -36.98 | 163 | - | 182 | 0 | 172 | K.LPDPASTDNAEFQIVLTIIR.D | | 2309.0366 | 2308.0293 | 2308.1124 | -35.98 | 465 | - | 484 | 0 | --- | K.EQADYISVSADGPYKPLHYR.Y | | 2309.0366 | 2308.0293 | 2308.1124 | -35.98 | 465 | - | 484 | 0 | 164 | K.EQADYISVSADGPYKPLHYR.Y |  | **No match to:** 713.3956, 730.4021, 735.3881, 750.3872, 768.5074, 830.4142, 842.4782, 850.5122, 857.4249, 868.5213, 882.5326, 892.4570, 910.4150, 914.3793, 972.4574, 977.4918, 992.4746, 1003.5277, 1014.4621, 1021.4946, 1021.4946, 1026.4689, 1044.4229, 1044.4229, 1053.5497, 1060.4216, 1121.5331, 1128.4984, 1129.4901, 1130.5515, 1145.5051, 1168.5430, 1170.5573, 1187.6241, 1190.5299, 1210.6320, 1223.5725, 1233.5765, 1241.5432, 1259.5374, 1259.5374, 1275.5483, 1281.5304, 1306.6172, 1312.6993, 1324.6039, 1356.5856, 1438.7806, 1447.6937, 1465.7037, 1465.7037, 1471.7185, 1472.6779, 1479.7214, 1537.6962, 1572.7866, 1636.8259, 1729.7230, 1783.8079, 1801.8372, 1817.8793, 1819.8323, 1825.8378, 1865.8993, 1874.9023, 1885.8972, 1891.9260, 1900.9204, 1901.9274, 1913.9125, 1917.9393, 1928.8615, 1966.8518, 1992.8794, 1992.8794, 2006.9036, 2008.9080, 2041.0067, 2087.9644, 2161.9009, 2178.9033, 2178.9033, 2183.9033, 2185.9216, 2194.8977, 2227.1079, 2266.0759, 2291.0227, 2291.0227, 2308.0723, 2331.0171 | | --- |  | **S38.** | [Cs3g01140.1](http://localhost/mascot/cgi/protein_view.pl?file=../data/20140808/F003909.dat&hit=1)    **Mass:** 35212    **Score:** 396    **Expect:** 1.1e-035  **Matches:** 14 | | --- | --- |  | **Observed** | **Mr(expt)** | **Mr(calc)** | **ppm** | **Start** |  | **End** | **Miss** | **Ions** | **Peptide** | | --- | --- | --- | --- | --- | --- | --- | --- | --- | --- | | 717.3152 | 716.3079 | 716.3163 | -11.71 | 1 | - | 6 | 0 | --- | -.MNGPEK.E + Acetyl (Protein N-term) | | 733.3460 | 732.3387 | 732.3112 | 37.5 | 1 | - | 6 | 0 | --- | -.MNGPEK.E + Acetyl (Protein N-term); Oxidation (M) | | 951.4401 | 950.4328 | 950.4498 | -17.85 | 172 | - | 179 | 0 | --- | K.AAWEFAEK.N | | 1606.7578 | 1605.7505 | 1605.7998 | -30.69 | 60 | - | 73 | 0 | --- | R.VYEANILDNEAISR.A | | 1606.7578 | 1605.7505 | 1605.7998 | -30.69 | 60 | - | 73 | 0 | 122 | R.VYEANILDNEAISR.A | | 1682.7782 | 1681.7709 | 1681.8213 | -29.93 | 217 | - | 230 | 0 | --- | K.DTQEHYWLGAVHVK.D | | 1682.7782 | 1681.7709 | 1681.8213 | -29.93 | 217 | - | 230 | 0 | 84 | K.DTQEHYWLGAVHVK.D | | 1802.9299 | 1801.9226 | 1801.9826 | -33.26 | 295 | - | 310 | 0 | --- | R.LISLGLDFTPVEETIR.E | | 1802.9299 | 1801.9226 | 1801.9826 | -33.26 | 295 | - | 310 | 0 | 152 | R.LISLGLDFTPVEETIR.E | | 1879.0226 | 1878.0153 | 1878.0826 | -35.81 | 99 | - | 116 | 0 | --- | K.ELLIPAVQGTLNVLEAAK.K | | 1896.9199 | 1895.9126 | 1895.8764 | 19.1 | 249 | - | 264 | 0 | --- | R.YLCTNGIYQFAEFAEK.V | | 1896.9199 | 1895.9126 | 1895.8764 | 19.1 | 249 | - | 264 | 0 | --- | R.YLCTNGIYQFAEFAEK.V | | 2686.2813 | 2685.2740 | 2685.3551 | -30.19 | 30 | - | 55 | 0 | --- | K.GYTNIHAAIFPGTDASHLFSLPGATK.L | | 3176.5444 | 3175.5371 | 3175.6415 | -32.86 | 180 | - | 210 | 0 | --- | K.NGTDVVAIHPATSLGPFPQPYVNASGAVLQR.L |  | **No match to:** 706.3686, 713.4008, 744.3628, 747.3680, 763.3891, 768.5172, 790.3476, 807.4136, 807.4136, 811.3809, 825.4011, 827.3884, 827.3884, 830.4025, 832.2872, 832.2872, 842.4851, 868.5284, 882.5452, 946.4548, 967.4318, 972.4705, 990.4775, 1005.5283, 1014.5433, 1021.5072, 1034.4844, 1041.5179, 1085.5396, 1096.5845, 1128.5251, 1131.4980, 1154.5773, 1171.5842, 1171.5842, 1185.6082, 1185.6082, 1193.5858, 1225.5193, 1228.6158, 1281.5880, 1302.6108, 1314.6296, 1316.6017, 1324.6106, 1342.6271, 1343.5917, 1346.6599, 1356.6134, 1376.6873, 1378.6863, 1385.6414, 1387.6608, 1391.5826, 1407.5654, 1407.5654, 1412.5730, 1423.5686, 1429.6044, 1444.6571, 1449.7222, 1449.7222, 1463.7461, 1465.7307, 1471.7045, 1534.7781, 1534.7781, 1535.7505, 1545.7085, 1588.7611, 1611.7783, 1620.7781, 1620.7781, 1630.7518, 1686.7821, 1698.7767, 1714.7838, 1728.7611, 1732.7378, 1743.8336, 1784.8827, 1801.8816, 1816.9448, 1819.8597, 1819.8597, 1844.8665, 1845.9183, 1893.8678, 1895.8860, 1949.9235, 1953.8594, 1960.9635, 1960.9635, 1964.9550, 1967.9392, 1975.8744, 1979.9401, 1995.9373, 2079.9797, 3159.4976, 3177.5369 | | --- | |  |   **Unidentified protein spots**   | **S6.** | [Cs7g27290.1](http://localhost/mascot/cgi/protein_view.pl?file=../data/20140415/F003308.dat&hit=1)    **Mass:** 26545    **Score:** 105    **Expect:** 1.4e-006  **Matches:** 4 | | --- | --- |  | **Observed** | **Mr(expt)** | **Mr(calc)** | **ppm** | **Start** |  | **End** | **Miss** | **Ions** | **Peptide** | | --- | --- | --- | --- | --- | --- | --- | --- | --- | --- | | 1155.5563 | 1154.5490 | 1154.5931 | -38.20 | 74 | - | 83 | 0 | --- | R.LGEVPENLER.Y | | 1155.5563 | 1154.5490 | 1154.5931 | -38.20 | 74 | - | 83 | 0 | 50 | R.LGEVPENLER.Y | | 1355.6189 | 1354.6116 | 1354.6558 | -32.58 | 168 | - | 180 | 0 | --- | K.YPGGAFDPLGFSK.D | | 1355.6189 | 1354.6116 | 1354.6558 | -32.58 | 168 | - | 180 | 0 | 36 | K.YPGGAFDPLGFSK.D |  | **No match to:** 948.6606, 1169.5842, 1169.5842, 1334.6079, 1334.6079, 1335.5980, 1833.8923, 1833.8923, 3068.3613 | | | --- | --- | |  |  | | **S12.**[**Cs3g20630.1**](http://localhost/mascot/cgi/protein_view.pl?file=../data/20140415/F003314.dat&hit=1)**Mass: 19894    Score: 60     Expect: 0.047  Matches: 4** | |  | **Observed** | **Mr(expt)** | **Mr(calc)** | **ppm** | **Start** |  | **End** | **Miss** | **Ions** | **Peptide** | | --- | --- | --- | --- | --- | --- | --- | --- | --- | --- | | 1017.6243 | 1016.6170 | 1016.5800 | 36.4 | 64 | - | 72 | 1 | --- | R.NSLILCKAR.E | | 1051.5712 | 1050.5639 | 1050.6114 | -45.16 | 148 | - | 156 | 0 | --- | R.SIPTVLFFK.N | | 1201.5988 | 1200.5915 | 1200.6424 | -42.34 | 111 | - | 121 | 0 | --- | R.MIAPAIEELAK.E + Oxidation (M) | | 1201.5988 | 1200.5915 | 1200.6424 | -42.34 | 111 | - | 121 | 0 | 43 | R.MIAPAIEELAK.E + Oxidation (M) |  | **No match to:** 713.3846, 728.5224, 734.4617, 736.4716, 750.4958, 758.5186, 768.4946, 772.5397, 792.5453, 802.5430, 816.5657, 834.4648, 836.4785, 842.4672, 848.4887, 850.4913, 850.4913, 851.4810, 855.0015, 860.5884, 860.5884, 868.5099, 877.0143, 877.0143, 882.5269, 904.6147, 904.6147, 934.6248, 948.6434, 948.6434, 964.5743, 992.6670, 992.6670, 1036.6876, 1036.6876, 1073.5504, 1080.7151, 1080.7151, 1089.5259, 1124.7426, 1137.6161, 1137.6161, 1151.6691, 1168.7611, 1227.6033, 1277.6162, 1304.6097, 1304.6097, 2022.9026 | | --- | | **S15.**[orange1.1t04780.1](http://localhost/mascot/cgi/protein_view.pl?file=../data/20140415/F003326.dat&hit=1)    **Mass:** 38295    **Score:** 50     **Expect:** 0.49  **Matches:** 26 |  | **Observed** | **Mr(expt)** | **Mr(calc)** | **ppm** | **Start** |  | **End** | **Miss** | **Ions** | **Peptide** | | --- | --- | --- | --- | --- | --- | --- | --- | --- | --- | | 1030.5463 | 1029.5390 | 1029.5091 | 29.1 | 56 | - | 65 | 0 | --- | K.LALAEDAGDR.G | | 1030.5463 | 1029.5390 | 1029.5091 | 29.1 | 56 | - | 65 | 0 | 8 | K.LALAEDAGDR.G | | 1416.7902 | 1415.7829 | 1415.7555 | 19.4 | 336 | - | 347 | 1 | --- | K.IDMELALQVGRR.T + Oxidation (M) | | 1416.7902 | 1415.7829 | 1415.7555 | 19.4 | 336 | - | 347 | 1 | 131 | K.IDMELALQVGRR.T + Oxidation (M) | | 1553.7456 | 1552.7383 | 1552.8613 | -79.21 | 42 | - | 55 | 1 | --- | K.LPSHPTYDLKGVVK.L | | 1553.7456 | 1552.7383 | 1552.8613 | -79.21 | 42 | - | 55 | 1 | --- | K.LPSHPTYDLKGVVK.L | | 2225.2471 | 2224.2398 | 2224.1892 | 22.8 | 32 | - | 51 | 1 | --- | R.KPGFESPAIKLPSHPTYDLK.G | | 2225.2471 | 2224.2398 | 2224.1497 | 40.5 | 1 | - | 20 | 0 | --- | -.MPGILSISTLVWPHLCTNSR.Q |  | **No match to:** 719.3457, 736.3745, 776.4602, 822.4089, 859.4746, 860.6365, 876.4966, 901.4884, 904.5651, 904.5651, 1108.5393, 1112.6396, 1252.7272, 1252.7272, 1266.7371, 1324.6394, 1344.6637, 1344.6637, 1408.6604, 1408.6604, 1430.7949, 1430.7949, 1535.7761, 1535.7761, 1567.7213, 1620.8613, 1624.9469, 1624.9469, 1722.8776, 1722.8776, 1788.8956, 1852.9016, 1852.9016, 2289.2463, 2289.2463, 2305.2661, 2305.2661 | | --- | | **S18.**[Cs3g08210.1](http://localhost/mascot/cgi/protein_view.pl?file=../data/20140415/F003330.dat&hit=1)    **Mass:** 180121   **Score:** 50     **Expect:** 0.47  **Matches:** 10 |  | **Observed** | **Mr(expt)** | **Mr(calc)** | **ppm** | **Start** |  | **End** | **Miss** | **Ions** | **Peptide** | | --- | --- | --- | --- | --- | --- | --- | --- | --- | --- | | 734.4790 | 733.4717 | 733.3615 | 150 | 1524 | - | 1529 | 1 | --- | R.QCPKMK.I | | 824.4507 | 823.4434 | 823.3932 | 61.0 | 1462 | - | 1468 | 1 | --- | R.MKIADCK.M + Oxidation (M) | | 1036.7153 | 1035.7080 | 1035.5713 | 132 | 115 | - | 123 | 1 | --- | R.YQLSKQAAK.A | | 1053.4391 | 1052.4318 | 1052.6230 | -181.61 | 1153 | - | 1161 | 0 | --- | K.VLILDDLPR.L | | 1066.4922 | 1065.4849 | 1065.5165 | -29.60 | 1576 | - | 1584 | 0 | --- | K.LFNEMVSIN.- | | 1153.5343 | 1152.5270 | 1152.5631 | -31.31 | 953 | - | 962 | 1 | --- | K.VIVSSCDRMK.Y + Oxidation (M) | | 1153.5343 | 1152.5270 | 1152.5631 | -31.31 | 953 | - | 962 | 1 | 18 | K.VIVSSCDRMK.Y + Oxidation (M) | | 1549.6971 | 1548.6898 | 1548.6977 | -5.10 | 469 | - | 483 | 0 | --- | K.ASCLLLDGDAEDEAK.M | | 1553.7032 | 1552.6959 | 1552.6828 | 8.46 | 986 | - | 999 | 0 | --- | K.SMEGVVDTTGWSER.D | | 1553.7032 | 1552.6959 | 1552.8864 | -122.68 | 761 | - | 772 | 1 | --- | K.LLLERTEYLYLK.E | | 1565.6935 | 1564.6862 | 1564.7708 | -54.04 | 500 | - | 512 | 0 | --- | K.LMFNIPNVADFER.E | | 1565.6935 | 1564.6862 | 1564.7708 | -54.04 | 500 | - | 512 | 0 | 3 | K.LMFNIPNVADFER.E | | 1581.7103 | 1580.7030 | 1580.7657 | -39.65 | 500 | - | 512 | 0 | --- | K.LMFNIPNVADFER.E + Oxidation (M) | | 1582.6948 | 1581.6875 | 1581.7899 | -64.73 | 1392 | - | 1404 | 1 | --- | K.VQHLWKENAESNK.V | | 1583.6866 | 1582.6793 | 1582.7661 | -54.81 | 392 | - | 405 | 1 | --- | R.EIRGMDADVYSSIK.L | | 1583.6866 | 1582.6793 | 1582.7661 | -54.81 | 392 | - | 405 | 1 | --- | R.EIRGMDADVYSSIK.L | | 1600.6964 | 1599.6891 | 1599.9018 | -132.91 | 1110 | - | 1123 | 1 | --- | K.LKNLEINCSLVNLK.V | | 1600.6964 | 1599.6891 | 1599.9018 | -132.91 | 1110 | - | 1123 | 1 | --- | K.LKNLEINCSLVNLK.V | | 1842.8492 | 1841.8419 | 1841.8835 | -22.60 | 663 | - | 677 | 0 | --- | R.LEELYIGNSFSQWEK.V |  | **No match to:** 713.4019, 728.5373, 768.5121, 772.5625, 816.5855, 850.5140, 860.6165, 868.5349, 882.5362, 887.4219, 904.6407, 925.4892, 925.4892, 937.4835, 948.6659, 992.6999, 1000.5039, 1039.4100, 1039.4100, 1080.5081, 1080.5081, 1101.5582, 1101.5582, 1224.5240, 1224.5240, 1310.6726, 1310.6726, 1614.7096, 1614.7096, 1672.7679, 1672.7679, 1688.7603, 1858.8296, 1874.8618, 1948.9010, 1965.9265, 1965.9265, 1981.9304, 1981.9304, 2736.2500 | | | | --- | --- | --- | | **S22.** | [orange1.1t00308.3](http://localhost/mascot/cgi/protein_view.pl?file=../data/20140415/F003334.dat&hit=1)    **Mass:** 55228    **Score:** 57     **Expect:** 0.081  **Matches:** 24 |  |  | **Observed** | **Mr(expt)** | **Mr(calc)** | **ppm** | **Start** |  | **End** | **Miss** | **Ions** | **Peptide** | | --- | --- | --- | --- | --- | --- | --- | --- | --- | --- | | 772.5502 | 771.5429 | 771.4603 | 107 | 398 | - | 404 | 1 | --- | R.LTKAPSR.A | | 860.5889 | 859.5817 | 859.4624 | 139 | 97 | - | 103 | 1 | --- | R.RNSQLSR.E | | 1218.6013 | 1217.5940 | 1217.6074 | -10.99 | 51 | - | 61 | 0 | --- | R.QALLMSTSDPR.Q | | 1218.6013 | 1217.5940 | 1217.6768 | -67.99 | 192 | - | 202 | 0 | 117 | K.QYIPITGGTLR.T | | 1601.7549 | 1600.7476 | 1600.8362 | -55.31 | 384 | - | 397 | 1 | --- | K.GEYAVGTIPKWPQR.L | | 2076.9976 | 2075.9903 | 2076.1884 | -95.41 | 291 | - | 309 | 0 | --- | R.LLRPGGYLVISGPPVQWPK.Q | | 2076.9976 | 2075.9903 | 2076.1884 | -95.41 | 291 | - | 309 | 0 | --- | R.LLRPGGYLVISGPPVQWPK.Q |  | **No match to:** 816.5766, 904.6272, 1045.5557, 1045.5557, 1328.6207, 1328.6207, 1423.6985, 1431.6442, 1433.7190, 1433.7190, 1471.7211, 1471.7211, 1487.7031, 1487.7031, 1553.7483, 1553.7483, 1617.7572, 1617.7572, 1949.9269, 1954.9579, 1954.9579, 1968.9602, 2012.9480, 2141.0466, 2141.0466, 2171.0256, 2269.1401, 2269.1401, 2413.0994, 2413.0994, 2627.2361, 2627.2361, 2989.4792, 2989.4792, 3844.8579 | | | | --- | --- | --- | | **S25.** | [Cs9g09840.1](http://localhost/mascot/cgi/protein_view.pl?file=../data/20140808/F003853.dat&hit=1)    **Mass:** 100571   **Score:** 67     **Expect:** 0.0095  **Matches:** 9 |  |  | **Observed** | **Mr(expt)** | **Mr(calc)** | **ppm** | **Start** |  | **End** | **Miss** | **Ions** | **Peptide** | | --- | --- | --- | --- | --- | --- | --- | --- | --- | --- | | 975.5250 | 974.5177 | 974.4669 | 52.1 | 323 | - | 332 | 0 | --- | K.GVGENVEGSK.I | | 1045.5621 | 1044.5548 | 1044.5200 | 33.4 | 197 | - | 205 | 0 | --- | K.VDVLNSENR.L | | 1045.5621 | 1044.5548 | 1044.5200 | 33.4 | 197 | - | 205 | 0 | 41 | K.VDVLNSENR.L | | 1314.5883 | 1313.5810 | 1313.7343 | -116.68 | 256 | - | 266 | 1 | --- | K.LHEKLTFLEGK.V | | 1433.7098 | 1432.7025 | 1432.7773 | -52.20 | 238 | - | 251 | 1 | --- | K.SLSEEGLSTKVGVK.F | | 1433.7098 | 1432.7025 | 1432.7773 | -52.20 | 238 | - | 251 | 1 | 19 | K.SLSEEGLSTKVGVK.F | | 1459.6693 | 1458.6620 | 1458.7678 | -72.53 | 318 | - | 332 | 1 | --- | K.IVGSKGVGENVEGSK.I | | 1631.7687 | 1630.7614 | 1630.8162 | -33.59 | 209 | - | 223 | 1 | --- | K.IDRSVGLGLNESDEK.I | | 2476.1792 | 2475.1719 | 2475.1788 | -2.76 | 779 | - | 801 | 1 | --- | K.FGVNAQSVFSRGDSIFLGCCNVR.S |  | **No match to: 783.4100, 804.2474, 810.3010, 811.3148, 832.2723, 832.2723, 842.4766, 910.4076, 1007.5468, 1021.4998, 1173.6217, 1187.6284, 1192.5635, 1201.6611, 1208.5508, 1218.5977, 1218.5977, 1240.5828, 1261.6641, 1327.5986, 1328.6167, 1328.6167, 1342.6365, 1350.6017, 1390.6340, 1399.7159, 1422.6279, 1423.0699, 1423.7213, 1431.6322, 1445.6493, 1447.7073, 1455.6779, 1465.7012, 1469.6927, 1471.7051, 1471.7051, 1485.7217, 1487.7046, 1487.7046, 1501.7211, 1502.7096, 1553.1644, 1553.7451, 1560.7499, 1567.7625, 1601.7493, 1601.7493, 1615.7626, 1617.7500, 1617.7500, 1864.8763, 1868.9127, 1931.9180, 1932.9105, 1936.9435, 1949.9150, 1949.9150, 1952.3203, 1954.9442, 1963.9362, 1968.9585, 1976.9291, 1987.8635, 2012.9456, 2060.9551, 2076.9763, 2076.9763, 2125.0474, 2125.0474, 2141.0520, 2141.0520, 2152.9937, 2156.1252, 2164.1013, 2167.0249, 2169.9888, 2171.0176, 2178.1255, 2185.0403, 2207.0979, 2233.9819, 2249.9868, 2266.0532, 2269.1279, 2281.9841, 2298.0039, 2313.0425, 2313.9902, 2396.0652, 2413.0823, 2413.0823, 2427.1147, 2428.0967, 2462.1677, 2609.1882, 2626.2014, 2693.3560, 2711.3494, 2971.4421, 2985.4612, 2988.4609, 2988.4609, 3002.4995, 3010.4719, 3843.7979** | | --- |  | **S29.** | [Cs4g01370.1](http://localhost/mascot/cgi/protein_view.pl?file=../data/20140415/F003337.dat&hit=1)    **Mass:** 104363   **Score:** 68     **Expect:** 0.0067  **Matches:** 11 | | --- | --- |  | **Observed** | **Mr(expt)** | **Mr(calc)** | **ppm** | **Start** |  | **End** | **Miss** | **Ions** | **Peptide** | | --- | --- | --- | --- | --- | --- | --- | --- | --- | --- | | 1101.5740 | 1100.5667 | 1100.5614 | 4.81 | 851 | - | 859 | 0 | --- | K.AWDNLLQNK.T | | 1101.5740 | 1100.5667 | 1100.6302 | -57.66 | 306 | - | 315 | 1 | 22 | R.LSQQGAITKR.M | | 1300.6416 | 1299.6343 | 1299.6782 | -33.78 | 902 | - | 912 | 1 | --- | R.ELNELAEQAKR.R | | 1300.6416 | 1299.6343 | 1299.6782 | -33.78 | 902 | - | 912 | 1 | 10 | R.ELNELAEQAKR.R | | 1829.7925 | 1828.7852 | 1828.9175 | -72.33 | 426 | - | 442 | 1 | --- | K.GAPEQIIDLCGLKGEMR.R | | 1829.7925 | 1828.7852 | 1828.9175 | -72.33 | 426 | - | 442 | 1 | --- | K.GAPEQIIDLCGLKGEMR.R | | 1845.7885 | 1844.7812 | 1844.9124 | -71.12 | 426 | - | 442 | 1 | --- | K.GAPEQIIDLCGLKGEMR.R + Oxidation (M) | | 1845.7885 | 1844.7812 | 1844.9124 | -71.12 | 426 | - | 442 | 1 | --- | K.GAPEQIIDLCGLKGEMR.R + Oxidation (M) | | 2342.0669 | 2341.0596 | 2341.1583 | -42.17 | 370 | - | 391 | 1 | --- | R.VENQDAIDASIVGMLADPKEAR.A | | 2342.0669 | 2341.0596 | 2341.1583 | -42.17 | 370 | - | 391 | 1 | 98 | R.VENQDAIDASIVGMLADPKEAR.A |  | **No match to:** 728.5201, 772.5510, 816.5743, 860.6080, 904.6135, 948.6428, 992.6686, 1036.6896, 1080.7129, 1111.5597, 1111.5597, 1128.5858, 1128.5858, 1155.5251, 1209.6465, 1267.6403, 1284.6827, 1284.6827, 1440.7656, 1446.7780, 1446.7780, 1642.7988, 1765.6808, 1766.8447, 1810.8684, 1810.8684, 1825.8801, 1857.7844, 1893.7849, 1893.7849, 1905.8029, 1909.7872, 1909.7872, 1921.7827, 1921.7827, 2023.8899, 2312.2263, 2312.2263, 2327.2292, 2328.0381, 2328.0381, 3082.6323 | | | | --- | --- | --- | | **S40.** | [Cs5g28200.1](http://localhost/mascot/cgi/protein_view.pl?file=../data/20140415/F003347.dat&hit=1)    **Mass:** 52086    **Score:** 63     **Expect:** 0.024  **Matches:** 5 |  |  | **Observed** | **Mr(expt)** | **Mr(calc)** | **ppm** | **Start** |  | **End** | **Miss** | **Ions** | **Peptide** | | --- | --- | --- | --- | --- | --- | --- | --- | --- | --- | | 1020.4996 | 1019.4923 | 1019.5400 | -46.73 | 126 | - | 134 | 0 | --- | R.AVQIAYEAR.K | | 1020.4996 | 1019.4923 | 1019.5400 | -46.73 | 126 | - | 134 | 0 | 49 | R.AVQIAYEAR.K | | 1450.6986 | 1449.6913 | 1449.7252 | -23.37 | 391 | - | 402 | 1 | --- | R.GIPSYWIDSEKR.I | | 1589.6725 | 1588.6652 | 1588.7919 | -79.76 | 203 | - | 216 | 0 | --- | K.NVQIVDTTCPWVSK.V | | 1589.6725 | 1588.6652 | 1588.7919 | -79.76 | 203 | - | 216 | 0 | --- | K.NVQIVDTTCPWVSK.V |  | **No match to:** 816.5731, 904.6260, 904.6260, 948.6578, 1265.6436, 1265.6436, 1324.5918, 1324.5918, 1377.6981, 1377.6981, 1437.6831, 1437.6831, 1454.6777, 1454.6777, 1466.7114, 1479.6272, 1479.6272, 1493.6710, 1493.6710, 1577.6621, 1577.6621, 1620.7866, 1972.9344, 2727.1892, 2727.1892 | | --- |   **Proteins identified in *Citrus grandis***  Photosynthesis, carbohydrate and energy metabolism   | **G1.** | [Cs6g11900.1](http://localhost/mascot/cgi/protein_view.pl?file=../data/20140815/F003960.dat&hit=1)    **Mass:** 30355    **Score:** 358    **Expect:** 7e-032  **Matches:** 9 | | --- | --- |   **Observed Mr(expt) Mr(calc) ppm Start End Miss Ions Peptide**  764.3362 763.3289 763.3501 -27.70 177 - 182 0 --- R.EEFSPR.G  920.4428 919.4355 919.4512 -17.06 176 - 182 1 --- R.REEFSPR.G  920.4428 919.4355 919.4876 -56.65 167 – 175 0 68 R.VNSGPPPPR.R  1044.4530 1043.4457 1043.4744 -27.52 186 - 198 0 --- R.GGGAGAPSSGGNR.V  1044.4530 1043.4457 1043.4744 -27.52 186 - 198 0 87 R.GGGAGAPSSGGNR.V  1076.5759 1075.5686 1075.5887 -18.64 167 - 176 1 --- R.VNSGPPPPRR.E  2903.2766 2902.2693 2902.3621 -31.97 239 - 266 0 --- R.GFGFVTYSSAEEVDNAIDSLNGVDLAGR.A  2903.2766 2902.2693 2902.3621 -31.97 239 - 266 0 186 R.GFGFVTYSSAEEVDNAIDSLNGVDLAGR.A  **No match to:** 707.3712, 716.3805, 731.3906, 742.4378, 746.3457, 768.5192, 773.4350, 778.3654, 812.3545, 816.4377, 842.4816, 844.4642, 844.4642, 850.4827, 851.4410, 868.5293, 870.4929, 882.5463, 891.4215, 902.4201, 903.4049, 928.4760, 934.4573, 942.4492, 945.5507, 945.5507, 958.4313, 964.4764, 967.5359, 983.4966, 1004.4753, 1004.4753, 1010.4987, 1055.5110, 1086.4771, 1090.4722, 1110.5554, 1153.5737, 1161.5774, 1173.5334, 1195.5182, 1205.6298, 1212.5427, 1244.5623, 1248.5328, 1260.5670, 1322.6749, 1336.5642, 1346.5831, 1348.6743, 1376.6685, 1394.6012, 1398.5997, 1410.6052, 1411.6211, 1424.6549, 1428.6489, 1434.7180, 1440.6571, 1447.6296, 1456.6547, 1475.7114, 1529.7662, 1542.7295, 1560.7406, 1560.7406, 1582.7318, 1625.7671, 1670.8406, 1771.9071, 1777.8494, 1972.8112, 1976.8070, 1976.8070, 1988.8163, 1993.8961, 1997.9071, 2044.9253, 2113.9404, 2114.9458, 2116.9314, 2116.9314, 2121.0186, 2131.9407, 2131.9407, 2145.9617, 2203.9780, 2225.9592, 2225.9592, 2253.0874, 2271.0879, 2271.0879, 2281.0999, 2295.0278, 2299.0789, 2312.9890, 2312.9890, 2351.9207, 2480.0002, 2495.1218, 2725.1321, 2725.1321, 2820.2905, 2820.2905, 3004.2803, 3050.2644, 3068.3394   | **G30.** | [Cs7g13970.1](http://localhost/mascot/cgi/protein_view.pl?file=../data/20140815/F004004.dat&hit=1)    **Mass:** 45037    **Score:** 902    **Expect:** 2.8e-086  **Matches:** 22 | | --- | --- |  | **Observed** | **Mr(expt)** | **Mr(calc)** | **ppm** | **Start** |  | **End** | **Miss** | **Ions** | **Peptide** | | --- | --- | --- | --- | --- | --- | --- | --- | --- | --- | | 842.4438 | 841.4365 | 841.4334 | 3.71 | 159 | - | 165 | 0 | --- | R.FNSISFK.G | | 920.3959 | 919.3887 | 919.4334 | -48.67 | 303 | - | 309 | 0 | --- | R.IQNMGWR.A + Oxidation (M) | | 920.3959 | 919.3887 | 919.4334 | -48.67 | 303 | - | 309 | 0 | 21 | R.IQNMGWR.A + Oxidation (M) | | 1076.4905 | 1075.4832 | 1075.5345 | -47.69 | 302 | - | 309 | 1 | --- | R.RIQNMGWR.A + Oxidation (M) | | 1096.5186 | 1095.5113 | 1095.5713 | -54.75 | 344 | - | 353 | 0 | --- | R.GFGILDVGYR.S | | 1096.5186 | 1095.5113 | 1095.5713 | -54.75 | 344 | - | 353 | 0 | 23 | R.GFGILDVGYR.S | | 1099.5597 | 1098.5524 | 1098.6186 | -60.21 | 310 | - | 319 | 0 | --- | R.ADGGLWLLVR.G | | 1224.5360 | 1223.5287 | 1223.5782 | -40.46 | 267 | - | 278 | 0 | --- | R.SPDGSYVAVSSR.G | | 1224.5360 | 1223.5287 | 1223.5782 | -40.46 | 267 | - | 278 | 0 | --- | R.SPDGSYVAVSSR.G | | 1349.6515 | 1348.6442 | 1348.6986 | -40.35 | 382 | - | 394 | 0 | --- | K.AADNIAANLYSVK.F | | 1359.6561 | 1358.6488 | 1358.7154 | -48.98 | 233 | - | 245 | 0 | --- | R.AAVQETVSATLNR.T | | 1359.6561 | 1358.6488 | 1358.7154 | -48.98 | 233 | - | 245 | 0 | --- | R.AAVQETVSATLNR.T | | 1556.6047 | 1555.5974 | 1555.6790 | -52.46 | 146 | - | 158 | 0 | --- | R.SIPSAEEEDFNYR.F | | 1556.6047 | 1555.5974 | 1555.6790 | -52.46 | 146 | - | 158 | 0 | --- | R.SIPSAEEEDFNYR.F | | 1588.7275 | 1587.7202 | 1587.7893 | -43.49 | 354 | - | 369 | 0 | --- | R.SQDEAWAAGGSGVLLK.T | | 1676.8191 | 1675.8118 | 1675.8855 | -43.95 | 191 | - | 205 | 0 | --- | R.IPLSSQLPGDMVYIK.A + Oxidation (M) | | 1676.8191 | 1675.8118 | 1675.8855 | -43.95 | 191 | - | 205 | 0 | --- | R.IPLSSQLPGDMVYIK.A + Oxidation (M) | | 1692.8676 | 1691.8603 | 1691.9247 | -38.03 | 400 | - | 415 | 1 | --- | K.KGFVLGNDGVLLQYLG.- | | 1777.7676 | 1776.7603 | 1776.8530 | -52.16 | 328 | - | 343 | 0 | --- | K.GTGITEEFEEVPVQSR.G | | 1777.7676 | 1776.7603 | 1776.8530 | -52.16 | 328 | - | 343 | 0 | --- | K.GTGITEEFEEVPVQSR.G | | 1872.7771 | 1871.7698 | 1871.8571 | -46.62 | 211 | - | 227 | 0 | --- | K.SAEMVTDEGAIYITSNR.G + Oxidation (M) | | 1872.7771 | 1871.7698 | 1871.8571 | -46.62 | 211 | - | 227 | 0 | 42 | K.SAEMVTDEGAIYITSNR.G + Oxidation (M) | | 1900.8024 | 1899.7951 | 1899.8592 | -33.73 | 44 | - | 62 | 1 | --- | R.ACSLPSSDSSSSSSSLSRR.Q | | 2181.9304 | 2180.9231 | 2181.0338 | -50.76 | 246 | - | 266 | 0 | --- | R.TVSSGISGASYYTGTFNTVNR.S | | 2181.9304 | 2180.9231 | 2181.0338 | -50.76 | 246 | - | 266 | 0 | --- | R.TVSSGISGASYYTGTFNTVNR.S |  | **No match to:** 700.2955, 709.2782, 711.2864, 726.3062, 727.2939, 749.3096, 755.3415, 791.3339, 792.3600, 832.2731, 856.3817, 857.3655, 907.4168, 918.3964, 924.3974, 934.3865, 936.3923, 936.3923, 952.3900, 968.4460, 974.4145, 1072.4667, 1078.5107, 1080.4945, 1082.4805, 1085.5641, 1088.4539, 1090.4641, 1090.4641, 1094.4679, 1103.5663, 1103.5663, 1106.4634, 1112.5551, 1113.5585, 1114.5535, 1115.5656, 1115.5656, 1118.5076, 1122.4644, 1131.5625, 1131.5625, 1144.4915, 1153.5803, 1153.5803, 1156.5564, 1159.5791, 1167.5896, 1171.5941, 1173.5913, 1195.5815, 1199.5613, 1206.6328, 1213.5604, 1223.6190, 1252.5442, 1254.5532, 1538.6089, 1570.6311, 1604.7194, 1612.8203, 1652.5966, 1714.8090, 1759.7843, 1775.7867, 1780.1510, 1781.7932, 1785.7966, 1791.7925, 1795.7958, 1796.7872, 1797.7920, 1800.7803, 1806.7941, 1807.7944, 1808.7802, 1813.7906, 1835.8129, 1836.7979, 1854.7903, 1898.8258, 1968.7721, 2137.9050, 2163.9329, 2179.9329, 2183.4521, 2203.9326, 2277.9272, 2351.0046, 2355.0005 | | --- |  | **G31.** | [Cs7g13970.1](http://localhost/mascot/cgi/protein_view.pl?file=../data/20140815/F004004.dat&hit=1)    **Mass:** 45037    **Score:** 684    **Expect:** 2.8e-086  **Matches:** 28 | | --- | --- |  | **Observed** | **Mr(expt)** | **Mr(calc)** | **ppm** | **Start** |  | **End** | **Miss** | **Ions** | **Peptide** | | --- | --- | --- | --- | --- | --- | --- | --- | --- | --- | | 842.4438 | 841.4365 | 841.4334 | 3.71 | 159 | - | 165 | 0 | --- | R.FNSISFK.G | | 920.3959 | 919.3887 | 919.4334 | -48.67 | 303 | - | 309 | 0 | --- | R.IQNMGWR.A + Oxidation (M) | | 920.3959 | 919.3887 | 919.4334 | -48.67 | 303 | - | 309 | 0 | 21 | R.IQNMGWR.A + Oxidation (M) | | 1076.4905 | 1075.4832 | 1075.5345 | -47.69 | 302 | - | 309 | 1 | --- | R.RIQNMGWR.A + Oxidation (M) | | 1096.5186 | 1095.5113 | 1095.5713 | -54.75 | 344 | - | 353 | 0 | --- | R.GFGILDVGYR.S | | 1096.5186 | 1095.5113 | 1095.5713 | -54.75 | 344 | - | 353 | 0 | 63 | R.GFGILDVGYR.S | | 1099.5597 | 1098.5524 | 1098.6186 | -60.21 | 310 | - | 319 | 0 | --- | R.ADGGLWLLVR.G | | 1224.5360 | 1223.5287 | 1223.5782 | -40.46 | 267 | - | 278 | 0 | --- | R.SPDGSYVAVSSR.G | | 1224.5360 | 1223.5287 | 1223.5782 | -40.46 | 267 | - | 278 | 0 | 80 | R.SPDGSYVAVSSR.G | | 1349.6515 | 1348.6442 | 1348.6986 | -40.35 | 382 | - | 394 | 0 | --- | K.AADNIAANLYSVK.F | | 1359.6561 | 1358.6488 | 1358.7154 | -48.98 | 233 | - | 245 | 0 | --- | R.AAVQETVSATLNR.T | | 1359.6561 | 1358.6488 | 1358.7154 | -48.98 | 233 | - | 245 | 0 | 84 | R.AAVQETVSATLNR.T | | 1556.6047 | 1555.5974 | 1555.6790 | -52.46 | 146 | - | 158 | 0 | --- | R.SIPSAEEEDFNYR.F | | 1556.6047 | 1555.5974 | 1555.6790 | -52.46 | 146 | - | 158 | 0 | 107 | R.SIPSAEEEDFNYR.F | | 1588.7275 | 1587.7202 | 1587.7893 | -43.49 | 354 | - | 369 | 0 | --- | R.SQDEAWAAGGSGVLLK.T | | 1676.8191 | 1675.8118 | 1675.8855 | -43.95 | 191 | - | 205 | 0 | --- | R.IPLSSQLPGDMVYIK.A + Oxidation (M) | | 1676.8191 | 1675.8118 | 1675.8855 | -43.95 | 191 | - | 205 | 0 | 69 | R.IPLSSQLPGDMVYIK.A + Oxidation (M) | | 1692.8676 | 1691.8603 | 1691.9247 | -38.03 | 400 | - | 415 | 1 | --- | K.KGFVLGNDGVLLQYLG.- | | 1777.7676 | 1776.7603 | 1776.8530 | -52.16 | 328 | - | 343 | 0 | --- | K.GTGITEEFEEVPVQSR.G | | 1777.7676 | 1776.7603 | 1776.8530 | -52.16 | 328 | - | 343 | 0 | 103 | K.GTGITEEFEEVPVQSR.G | | 1872.7771 | 1871.7698 | 1871.8571 | -46.62 | 211 | - | 227 | 0 | --- | K.SAEMVTDEGAIYITSNR.G + Oxidation (M) | | 1872.7771 | 1871.7698 | 1871.8571 | -46.62 | 211 | - | 227 | 0 | 107 | K.SAEMVTDEGAIYITSNR.G + Oxidation (M) | | 1900.8024 | 1899.7951 | 1899.8592 | -33.73 | 44 | - | 62 | 1 | --- | R.ACSLPSSDSSSSSSSLSRR.Q | | 2181.9304 | 2180.9231 | 2181.0338 | -50.76 | 246 | - | 266 | 0 | --- | R.TVSSGISGASYYTGTFNTVNR.S | | 2181.9304 | 2180.9231 | 2181.0338 | -50.76 | 246 | - | 266 | 0 | 172 | R.TVSSGISGASYYTGTFNTVNR.S |  | **No match to:** 700.2955, 709.2782, 711.2864, 726.3062, 727.2939, 749.3096, 755.3415, 791.3339, 792.3600, 832.2731, 856.3817, 857.3655, 907.4168, 918.3964, 924.3974, 934.3865, 936.3923, 936.3923, 952.3900, 968.4460, 974.4145, 1072.4667, 1078.5107, 1080.4945, 1082.4805, 1085.5641, 1088.4539, 1090.4641, 1090.4641, 1094.4679, 1103.5663, 1103.5663, 1106.4634, 1112.5551, 1113.5585, 1114.5535, 1115.5656, 1115.5656, 1118.5076, 1122.4644, 1131.5625, 1131.5625, 1144.4915, 1153.5803, 1153.5803, 1156.5564, 1159.5791, 1167.5896, 1171.5941, 1173.5913, 1195.5815, 1199.5613, 1206.6328, 1213.5604, 1223.6190, 1252.5442, 1254.5532, 1538.6089, 1570.6311, 1604.7194, 1612.8203, 1652.5966, 1714.8090, 1759.7843, 1775.7867, 1780.1510, 1781.7932, 1785.7966, 1791.7925, 1795.7958, 1796.7872, 1797.7920, 1800.7803, 1806.7941, 1807.7944, 1808.7802, 1813.7906, 1835.8129, 1836.7979, 1854.7903, 1898.8258, 1968.7721, 2137.9050, 2163.9329, 2179.9329, 2183.4521, 2203.9326, 2277.9272, 2351.0046, 2355.0005 | | --- |  | **G10.** | [Cs1g25510.4](http://localhost/mascot/cgi/protein_view.pl?file=../data/20140815/F004018.dat&hit=1)    **Mass:** 76766    **Score:** 74     **Expect:** 0.0016  **Matches:** 14 | | --- | --- |  | **Observed** | **Mr(expt)** | **Mr(calc)** | **ppm** | **Start** |  | **End** | **Miss** | **Ions** | **Peptide** | | --- | --- | --- | --- | --- | --- | --- | --- | --- | --- | | 790.4500 | 789.4427 | 789.3504 | 117 | 240 | - | 245 | 0 | --- | K.VEEEER.G | | 860.4622 | 859.4549 | 859.4222 | 38.1 | 627 | - | 634 | 0 | --- | K.EASMGPLR.E | | 860.4622 | 859.4549 | 859.4222 | 38.1 | 627 | - | 634 | 0 | (24) | K.EASMGPLR.E | | 876.4631 | 875.4559 | 875.4171 | 44.3 | 627 | - | 634 | 0 | --- | K.EASMGPLR.E + Oxidation (M) | | 876.4631 | 875.4559 | 875.4171 | 44.3 | 627 | - | 634 | 0 | 47 | K.EASMGPLR.E + Oxidation (M) | | 1250.6907 | 1249.6834 | 1249.5469 | 109 | 346 | - | 357 | 0 | --- | R.NNGSNVGNMTSR.C | | 1266.6989 | 1265.6916 | 1265.5419 | 118 | 346 | - | 357 | 0 | --- | R.NNGSNVGNMTSR.C + Oxidation (M) | | 1266.6989 | 1265.6916 | 1265.5419 | 118 | 346 | - | 357 | 0 | 12 | R.NNGSNVGNMTSR.C + Oxidation (M) | | 1314.6925 | 1313.6852 | 1313.7125 | -20.78 | 623 | - | 634 | 1 | --- | K.NLVKEASMGPLR.E | | 1552.7175 | 1551.7102 | 1551.6069 | 66.6 | 22 | - | 34 | 0 | --- | R.EMEEEEQCGEVPK.T + Oxidation (M) | | 1591.6313 | 1590.6240 | 1590.8076 | -115.38 | 126 | - | 141 | 1 | --- | R.APGCAFSLKGGIDVEK.I | | 1652.8615 | 1651.8542 | 1651.8140 | 24.3 | 119 | - | 134 | 1 | --- | R.AFEQAGRAPGCAFSLK.G | | 2090.0034 | 2088.9961 | 2089.0927 | -46.21 | 424 | - | 440 | 0 | --- | K.CVMEMVIWPLLRPDIFK.G | | 2271.1309 | 2270.1236 | 2269.9289 | 85.8 | 22 | - | 40 | 1 | --- | R.EMEEEEQCGEVPKTTSCWR.K | | 2287.1438 | 2286.1365 | 2285.9239 | 93.0 | 22 | - | 40 | 1 | --- | R.EMEEEEQCGEVPKTTSCWR.K + Oxidation (M) |  | **No match to:** 719.3207, 736.3484, 771.4788, 776.4291, 795.4376, 797.4268, 812.4670, 815.4285, 822.3762, 822.3762, 832.2763, 836.3900, 842.4779, 843.4440, 859.4389, 889.4578, 901.4475, 901.4475, 904.5205, 924.4466, 935.4119, 946.4402, 1030.5118, 1030.5118, 1089.5061, 1090.4957, 1107.5123, 1107.5123, 1112.5994, 1139.5985, 1233.5748, 1252.6694, 1252.6694, 1260.5902, 1271.6832, 1290.6379, 1298.6992, 1317.6560, 1344.6069, 1392.6147, 1395.7061, 1408.6118, 1416.7164, 1416.7164, 1430.7438, 1438.7109, 1454.6808, 1517.7277, 1518.6996, 1534.7225, 1535.7064, 1535.7064, 1549.7379, 1553.6639, 1553.6639, 1567.6984, 1569.7273, 1573.6858, 1575.6610, 1602.7861, 1606.8556, 1610.7006, 1620.7976, 1624.8624, 1624.8624, 1640.8562, 1642.7797, 1646.8510, 1658.7452, 1662.8350, 1688.8298, 1706.7742, 1722.8044, 1744.8928, 1788.8441, 1836.8278, 1852.8350, 1998.9374, 2111.9612, 2183.1003, 2225.1311, 2225.1311, 2239.1350, 2241.1028, 2243.1001, 2273.1316, 2273.1316, 2289.1379, 2289.1379, 2303.1423, 2375.1143, 2376.1072, 2389.1262, 2597.2737, 2804.4089, 3238.5049, 3479.7151, 3561.7214, 3609.6853, 3625.7280 | | --- |  | **G42.** | [Cs1g25510.4](http://localhost/mascot/cgi/protein_view.pl?file=../data/20140815/F004017.dat&hit=1)    **Mass:** 37888    **Score:** 458    **Expect:** 7e-042  **Matches:** 26 | | --- | --- |  | **Observed** | **Mr(expt)** | **Mr(calc)** | **ppm** | **Start** |  | **End** | **Miss** | **Ions** | **Peptide** | | --- | --- | --- | --- | --- | --- | --- | --- | --- | --- | | 706.3601 | 705.3528 | 705.3810 | -39.88 | 83 | - | 88 | 0 | --- | K.TPYIGR.C | | 717.3030 | 716.2957 | 716.3203 | -34.40 | 231 | - | 235 | 0 | --- | K.MFFEK.H + Oxidation (M) | | 733.3380 | 732.3307 | 732.3555 | -33.77 | 270 | - | 275 | 0 | --- | K.APENFR.L | | 792.3496 | 791.3423 | 791.3670 | -31.12 | 201 | - | 206 | 0 | --- | K.EMLMPR.D + Oxidation (M) | | 807.4044 | 806.3971 | 806.4286 | -39.07 | 276 | - | 282 | 0 | --- | R.LDFAVSR.E | | 807.4044 | 806.3971 | 806.4286 | -39.07 | 276 | - | 282 | 0 | 41 | R.LDFAVSR.E | | 808.3693 | 807.3620 | 807.3619 | 0.21 | 201 | - | 206 | 0 | --- | K.EMLMPR.D + 2 Oxidation (M) | | 811.3837 | 810.3764 | 810.4058 | -36.28 | 292 | - | 297 | 0 | --- | K.MYIQTR.M | | 827.3762 | 826.3689 | 826.4007 | -38.51 | 292 | - | 297 | 0 | --- | K.MYIQTR.M + Oxidation (M) | | 827.3762 | 826.3689 | 826.4007 | -38.51 | 292 | - | 297 | 0 | --- | K.MYIQTR.M + Oxidation (M) | | 990.4726 | 989.4653 | 989.4930 | -27.98 | 268 | - | 275 | 1 | --- | K.EKAPENFR.L | | 990.4726 | 989.4653 | 989.4930 | -27.98 | 268 | - | 275 | 1 | 41 | K.EKAPENFR.L | | 1141.6173 | 1140.6100 | 1140.5597 | 44.1 | 289 | - | 297 | 1 | --- | K.GEKMYIQTR.M + Oxidation (M) | | 1378.6733 | 1377.6660 | 1377.7140 | -34.80 | 165 | - | 176 | 0 | --- | R.LVYTNENGEIVK.G | | 1378.6733 | 1377.6660 | 1377.7140 | -34.80 | 165 | - | 176 | 0 | 17 | R.LVYTNENGEIVK.G | | 1387.6599 | 1386.6526 | 1386.6991 | -33.50 | 119 | - | 132 | 0 | --- | K.EGQSIGVIADGVDK.N | | 1389.6062 | 1388.5989 | 1388.6071 | -5.87 | 231 | - | 240 | 1 | --- | K.MFFEKHEDYK.F + Oxidation (M) | | 1534.7668 | 1533.7595 | 1533.8151 | -36.22 | 164 | - | 176 | 1 | --- | K.RLVYTNENGEIVK.G | | 1534.7668 | 1533.7595 | 1533.8151 | -36.22 | 164 | - | 176 | 1 | 62 | K.RLVYTNENGEIVK.G | | 1630.7438 | 1629.7365 | 1629.7886 | -31.96 | 141 | - | 156 | 0 | --- | R.LYSIASSALGDFGDSK.T | | 1630.7438 | 1629.7365 | 1629.7886 | -31.96 | 141 | - | 156 | 0 | 106 | R.LYSIASSALGDFGDSK.T | | 1943.9540 | 1942.9467 | 1943.0687 | -62.80 | 2 | - | 21 | 1 | --- | M.AAVSAAVSLPTSKSTSLPTR.T | | 1944.9580 | 1943.9507 | 1944.0139 | -32.49 | 207 | - | 225 | 0 | --- | R.DPNATVIMLATGTGIAPFR.G | | 1944.9580 | 1943.9507 | 1944.0139 | -32.49 | 207 | - | 225 | 0 | (98) | R.DPNATVIMLATGTGIAPFR.G | | 1960.9521 | 1959.9448 | 1960.0088 | -32.64 | 207 | - | 225 | 0 | --- | R.DPNATVIMLATGTGIAPFR.G + Oxidation (M) | | 1960.9521 | 1959.9448 | 1960.0088 | -32.64 | 207 | - | 225 | 0 | 122 | R.DPNATVIMLATGTGIAPFR.G + Oxidation (M) | | 2682.1719 | 2681.1646 | 2681.1956 | -11.54 | 95 | - | 118 | 0 | --- | K.ITGDDAPGETWHMVFSTEGEVPYK.E + Oxidation (M) |  | **No match to:** 713.3981, 728.3552, 744.3538, 747.3646, 758.4013, 763.3878, 768.5102, 781.3657, 790.3439, 806.4083, 823.3685, 830.3906, 842.4783, 848.3943, 868.5241, 882.5411, 895.4689, 914.3789, 946.4837, 966.4714, 972.4594, 1005.5245, 1085.5265, 1096.5768, 1103.5828, 1104.5818, 1128.5117, 1171.5990, 1179.5516, 1187.6121, 1193.5864, 1235.4872, 1251.4736, 1251.4736, 1261.6755, 1264.5956, 1281.5712, 1285.5764, 1297.5746, 1303.5780, 1314.5812, 1316.5594, 1324.6276, 1342.5928, 1343.1985, 1343.5593, 1343.5593, 1375.5702, 1391.5598, 1391.5598, 1396.5973, 1400.6539, 1407.5520, 1407.5520, 1412.5638, 1423.5565, 1429.6865, 1458.7156, 1471.6490, 1476.7195, 1480.7268, 1517.7135, 1519.6549, 1535.7352, 1548.7784, 1610.8142, 1629.7375, 1652.7268, 1660.8428, 1757.8331, 1802.8423, 1845.9222, 1893.8605, 1893.8605, 1895.8790, 1896.8984, 1903.9592, 1915.8625, 1921.9717, 1921.9717, 1931.8378, 1942.9296, 1976.9465, 1982.9470, 2056.9536, 2056.9536, 2072.9646, 2283.9321 | | --- |  | **G8.** | [Cs8g16040.3](http://localhost/mascot/cgi/protein_view.pl?file=../data/20140815/F003975.dat&hit=1)    **Mass:** 55390    **Score:** 1350   **Expect:** 4.4e-131  **Matches:** 47 | | --- | --- |  | **Observed** | **Mr(expt)** | **Mr(calc)** | **ppm** | **Start** |  | **End** | **Miss** | **Ions** | **Peptide** | | --- | --- | --- | --- | --- | --- | --- | --- | --- | --- | | 731.3663 | 730.3591 | 730.3609 | -2.59 | 324 | - | 329 | 0 | --- | K.DEIQAR.I | | 733.3497 | 732.3424 | 732.3555 | -17.87 | 262 | - | 268 | 0 | --- | K.APGFGER.R | | 798.4476 | 797.4403 | 797.4508 | -13.12 | 119 | - | 125 | 0 | --- | R.ARPIEGR.D | | 802.4522 | 801.4449 | 801.4596 | -18.33 | 204 | - | 210 | 0 | --- | R.VLVTDQK.I | | 898.5576 | 897.5503 | 897.5647 | -16.03 | 253 | - | 261 | 0 | --- | R.GILNVAAIK.A | | 940.5528 | 939.5455 | 939.5641 | -19.72 | 216 | - | 223 | 0 | --- | K.DIIPLLEK.T | | 1043.5676 | 1042.5603 | 1042.5771 | -16.11 | 33 | - | 42 | 0 | --- | K.VVNDGVTIAR.A | | 1090.5481 | 1089.5408 | 1089.5818 | -37.64 | 195 | - | 203 | 0 | --- | K.LIVEFENAR.V | | 1090.5481 | 1089.5408 | 1089.5818 | -37.64 | 195 | - | 203 | 0 | 85 | K.LIVEFENAR.V | | 1198.6569 | 1197.6496 | 1197.6717 | -18.46 | 8 | - | 19 | 0 | --- | K.LSDAVGLTLGPR.G | | 1198.6569 | 1197.6496 | 1197.6717 | -18.46 | 8 | - | 19 | 0 | 77 | K.LSDAVGLTLGPR.G | | 1204.6064 | 1203.5991 | 1203.6136 | -11.99 | 22 | - | 32 | 0 | --- | R.NVVLDEFGSPK.V | | 1269.6703 | 1268.6630 | 1268.6837 | -16.27 | 119 | - | 129 | 1 | --- | R.ARPIEGRDDIK.A | | 1290.5961 | 1289.5888 | 1289.6099 | -16.35 | 365 | - | 376 | 0 | --- | K.VGAATETELEDR.K | | 1290.5961 | 1289.5888 | 1289.6099 | -16.35 | 365 | - | 376 | 0 | 75 | K.VGAATETELEDR.K | | 1409.7394 | 1408.7321 | 1408.7674 | -25.05 | 107 | - | 118 | 1 | --- | K.TVHGLVEELEKR.A | | 1409.7394 | 1408.7321 | 1408.7674 | -25.05 | 107 | - | 118 | 1 | 79 | K.TVHGLVEELEKR.A | | 1418.6940 | 1417.6867 | 1417.7049 | -12.80 | 365 | - | 377 | 1 | --- | K.VGAATETELEDRK.L | | 1452.8790 | 1451.8717 | 1451.8963 | -16.91 | 211 | - | 223 | 1 | --- | K.ISAIKDIIPLLEK.T | | 1479.7135 | 1478.7062 | 1478.7405 | -23.21 | 182 | - | 194 | 0 | --- | R.GYISPQFVTNPEK.L | | 1479.7135 | 1478.7062 | 1478.7405 | -23.21 | 182 | - | 194 | 0 | 76 | R.GYISPQFVTNPEK.L | | 1485.6423 | 1484.6350 | 1484.6518 | -11.32 | 336 | - | 348 | 0 | --- | K.ELAETDSVYDSEK.L | | 1555.8698 | 1554.8625 | 1554.8981 | -22.88 | 86 | - | 101 | 0 | --- | K.LGLLSVTSGANPVSLK.R | | 1636.7476 | 1635.7403 | 1635.7700 | -18.15 | 65 | - | 81 | 0 | --- | K.TNDSAGDGTTTASVLAR.E | | 1636.7476 | 1635.7403 | 1635.7700 | -18.15 | 65 | - | 81 | 0 | 141 | K.TNDSAGDGTTTASVLAR.E | | 1711.9636 | 1710.9563 | 1710.9992 | -25.06 | 86 | - | 102 | 1 | --- | K.LGLLSVTSGANPVSLKR.G | | 1711.9636 | 1710.9563 | 1710.9992 | -25.06 | 86 | - | 102 | 1 | 90 | K.LGLLSVTSGANPVSLKR.G | | 1754.8767 | 1753.8694 | 1753.9032 | -19.27 | 43 | - | 59 | 0 | --- | R.AIELADPMENAGAALIR.E | | 1770.8710 | 1769.8637 | 1769.8981 | -19.44 | 43 | - | 59 | 0 | --- | R.AIELADPMENAGAALIR.E + Oxidation (M) | | 1770.8710 | 1769.8637 | 1769.8981 | -19.44 | 43 | - | 59 | 0 | 105 | R.AIELADPMENAGAALIR.E + Oxidation (M) | | 1904.9064 | 1903.8991 | 1903.9487 | -26.02 | 312 | - | 329 | 1 | --- | K.DSTTIIADAASKDEIQAR.I | | 1904.9064 | 1903.8991 | 1903.9487 | -26.02 | 312 | - | 329 | 1 | 166 | K.DSTTIIADAASKDEIQAR.I | | 1921.1335 | 1920.1262 | 1919.9775 | 77.5 | 471 | - | 487 | 1 | --- | K.YENMLQAGVIDPAKVTR.C + Oxidation (M) | | 2250.2561 | 2249.2488 | 2249.2882 | -17.52 | 229 | - | 250 | 0 | --- | R.APLLIIAEDVTGEALATLVVNK.L | | 2273.2097 | 2272.2024 | 2272.2427 | -17.71 | 432 | - | 454 | 0 | --- | K.ALVAPASLIAHNAGVEGEVVVEK.V | | 2273.2097 | 2272.2024 | 2272.2427 | -17.71 | 432 | - | 454 | 0 | 169 | K.ALVAPASLIAHNAGVEGEVVVEK.V | | 2291.1226 | 2290.1153 | 2290.1362 | -9.13 | 130 | - | 152 | 0 | --- | K.AVATISAGNDDLIGTMIADAIDK.V + Oxidation (M) | | 2954.5295 | 2953.5222 | 2953.5661 | -14.87 | 385 | - | 414 | 0 | --- | K.NATFAAIEEGIVPGGGAALVHLSDHVPAIK.D | | 2954.5295 | 2953.5222 | 2953.5661 | -14.87 | 385 | - | 414 | 0 | 77 | K.NATFAAIEEGIVPGGGAALVHLSDHVPAIK.D | | 3098.3882 | 3097.3809 | 3097.4285 | -15.37 | 153 | - | 181 | 0 | --- | K.VGPDGVLSIESSSSFETTVEVEEGMEIDR.G | | 3114.4050 | 3113.3977 | 3113.4234 | -8.26 | 153 | - | 181 | 0 | --- | K.VGPDGVLSIESSSSFETTVEVEEGMEIDR.G + Oxidation (M) | | 3197.6687 | 3196.6614 | 3196.6880 | -8.33 | 385 | - | 416 | 1 | --- | K.NATFAAIEEGIVPGGGAALVHLSDHVPAIKDK.L |  | **No match to:** 713.4177, 724.5003, 747.3754, 768.5258, 771.4615, 825.5732, 827.5190, 827.5190, 832.3016, 842.4936, 864.4087, 864.4087, 868.5402, 882.5534, 896.5829, 1051.6774, 1055.5201, 1072.5713, 1073.5602, 1088.5665, 1089.5491, 1104.5824, 1104.5824, 1112.5455, 1118.5732, 1132.6185, 1158.5828, 1243.6769, 1252.6848, 1255.6703, 1261.6783, 1299.7117, 1394.6179, 1398.6176, 1423.7474, 1465.7275, 1499.7603, 1501.6930, 1516.7360, 1517.6880, 1535.7551, 1618.8961, 1635.8241, 1700.8373, 1705.8843, 1706.8607, 1752.8652, 1778.8931, 1784.9008, 1789.8732, 1845.9174, 1886.9109, 1993.9667, 2033.0054, 2033.0054, 2036.0302, 2058.0291, 2100.0752, 2114.0833, 2156.9717, 2227.1099, 2272.2068, 2295.1501, 2663.2358, 2691.2339, 2695.2227, 2776.4602, 2823.4885, 2840.4927, 2902.4282, 3050.4458, 3052.3240, 3096.3914 | | --- |  | **G9.** | [**Cs7g31800.3**](http://localhost/mascot/cgi/protein_view.pl?file=../data/20140815/F004022.dat&hit=1)**Mass: 46932    Score: 617    Expect: 8.8e-058  Matches: 17** | | --- | --- |  | **Observed** | **Mr(expt)** | **Mr(calc)** | **ppm** | **Start** |  | **End** | **Miss** | **Ions** | **Peptide** | | --- | --- | --- | --- | --- | --- | --- | --- | --- | --- | | 809.4714 | 808.4641 | 808.5171 | -65.53 | 132 | - | 138 | 0 | --- | K.LVVHITK.N | | 895.3655 | 894.3583 | 894.4083 | -55.95 | 343 | - | 349 | 0 | --- | R.VYDDEVR.K | | 895.3655 | 894.3583 | 894.4083 | -55.95 | 343 | - | 349 | 0 | 38 | R.VYDDEVR.K | | 940.4225 | 939.4152 | 939.4603 | -47.97 | 294 | - | 300 | 0 | --- | K.FYWAPTR.E | | 940.4225 | 939.4152 | 939.4603 | -47.97 | 294 | - | 300 | 0 | 36 | K.FYWAPTR.E | | 1145.5117 | 1144.5044 | 1144.5513 | -40.93 | 369 | - | 378 | 0 | --- | K.EAAPTFEQPR.M | | 1145.5117 | 1144.5044 | 1144.5513 | -40.93 | 369 | - | 378 | 0 | 46 | K.EAAPTFEQPR.M | | 1725.7058 | 1724.6985 | 1724.7489 | -29.23 | 64 | - | 78 | 0 | --- | K.GLAYDESDDQQDITR.G | | 1725.7058 | 1724.6985 | 1724.7489 | -29.23 | 64 | - | 78 | 0 | 101 | K.GLAYDESDDQQDITR.G | | 1882.9106 | 1881.9033 | 1881.9625 | -31.46 | 324 | - | 340 | 0 | --- | K.LVDTFPGQSIDFFGALR.A | | 1882.9106 | 1881.9033 | 1881.9625 | -31.46 | 324 | - | 340 | 0 | 117 | K.LVDTFPGQSIDFFGALR.A | | 2063.0022 | 2061.9949 | 2062.0517 | -27.53 | 384 | - | 400 | 1 | --- | K.LLEYGNMIVQEQENVKR.V | | 2063.0022 | 2061.9949 | 2062.0517 | -27.53 | 384 | - | 400 | 1 | 98 | K.LLEYGNMIVQEQENVKR.V | | 2078.9985 | 2077.9912 | 2078.0466 | -26.65 | 384 | - | 400 | 1 | --- | K.LLEYGNMIVQEQENVKR.V + Oxidation (M) | | 2089.1052 | 2088.0979 | 2088.1619 | -30.65 | 269 | - | 287 | 0 | --- | R.VPIIVTGNDFSTLYAPLIR.D | | 2089.1052 | 2088.0979 | 2088.1619 | -30.65 | 269 | - | 287 | 0 | 119 | R.VPIIVTGNDFSTLYAPLIR.D |  | **No match to:** 787.3787, 831.4547, 847.4235, 863.3894, 944.4210, 956.4252, 956.4252, 972.4173, 972.4173, 1332.7255, 1332.7255, 1885.2839 | | --- |  | **G6.** | [Cs7g31640.4](http://localhost/mascot/cgi/protein_view.pl?file=../data/20140815/F003970.dat&hit=1)    **Mass:** 32350    **Score:** 489    **Expect:** 5.6e-045  **Matches:** 29 | | --- | --- |  | **Observed** | **Mr(expt)** | **Mr(calc)** | **ppm** | **Start** |  | **End** | **Miss** | **Ions** | **Peptide** | | --- | --- | --- | --- | --- | --- | --- | --- | --- | --- | | 703.3916 | 702.3843 | 702.4025 | -25.81 | 107 | - | 113 | 0 | --- | K.LTGVTGR.D | | 808.4521 | 807.4448 | 807.4603 | -19.14 | 189 | - | 194 | 1 | --- | K.QKYTLR.Y | | 908.3770 | 907.3697 | 907.5379 | -185.25 | 127 | - | 134 | 0 | --- | R.TTYVIAIK.D | | 908.3770 | 907.3697 | 907.5379 | -185.25 | 127 | - | 134 | 0 | 8 | R.TTYVIAIK.D | | 921.4470 | 920.4397 | 920.4538 | -15.35 | 164 | - | 171 | 0 | --- | K.MFSPGNLR.A | | 937.4311 | 936.4238 | 936.4487 | -26.61 | 164 | - | 171 | 0 | --- | K.MFSPGNLR.A + Oxidation (M) | | 937.4311 | 936.4238 | 936.4487 | -26.61 | 164 | - | 171 | 0 | 40 | K.MFSPGNLR.A + Oxidation (M) | | 959.4398 | 958.4325 | 958.4720 | -41.13 | 257 | - | 264 | 0 | --- | K.VINDLDDR.T | | 1115.5659 | 1114.5586 | 1114.4279 | 117 | 1 | - | 9 | 0 | --- | -.MMCMGEALR.T + Acetyl (Protein N-term); 2 Oxidation (M) | | 1188.5292 | 1187.5219 | 1187.5459 | -20.15 | 278 | - | 287 | 0 | --- | R.FEETLYGSSR.L | | 1188.5292 | 1187.5219 | 1187.5459 | -20.15 | 278 | - | 287 | 0 | 74 | R.FEETLYGSSR.L | | 1237.5999 | 1236.5926 | 1236.6350 | -34.29 | 212 | - | 223 | 0 | --- | K.GIFTNVTSPSSK.A | | 1348.6418 | 1347.6345 | 1347.6605 | -19.28 | 114 | - | 126 | 0 | --- | R.DQVAAAMGIYGPR.T | | 1364.6316 | 1363.6243 | 1363.6554 | -22.81 | 114 | - | 126 | 0 | --- | R.DQVAAAMGIYGPR.T + Oxidation (M) | | 1364.6316 | 1363.6243 | 1363.6554 | -22.81 | 114 | - | 126 | 0 | 43 | R.DQVAAAMGIYGPR.T + Oxidation (M) | | 1436.6003 | 1435.5930 | 1435.7671 | -121.24 | 212 | - | 225 | 1 | --- | K.GIFTNVTSPSSKAK.L | | 1478.7621 | 1477.7548 | 1477.7889 | -23.03 | 265 | - | 277 | 1 | --- | R.TQVAYGSKNEIIR.F | | 1649.8232 | 1648.8159 | 1648.8495 | -20.34 | 195 | - | 209 | 0 | --- | R.YTGGMVPDVNQIIVK.E + Oxidation (M) | | 1649.8232 | 1648.8159 | 1648.8495 | -20.34 | 195 | - | 209 | 0 | 68 | R.YTGGMVPDVNQIIVK.E + Oxidation (M) | | 1717.8096 | 1716.8023 | 1716.8359 | -19.56 | 135 | - | 149 | 0 | --- | K.DFPGTHEFLLLDEGK.W | | 1813.8646 | 1812.8573 | 1812.9006 | -23.87 | 273 | - | 287 | 1 | --- | K.NEIIRFEETLYGSSR.L | | 1813.8646 | 1812.8573 | 1812.9006 | -23.87 | 273 | - | 287 | 1 | 84 | K.NEIIRFEETLYGSSR.L | | 1906.8914 | 1905.8841 | 1905.9870 | -53.98 | 195 | - | 211 | 1 | --- | R.YTGGMVPDVNQIIVKEK.G + Oxidation (M) | | 2078.9651 | 2077.9578 | 2077.9996 | -20.13 | 172 | - | 188 | 1 | --- | R.ATFDNPDYDKLINYYVK.Q | | 2078.9651 | 2077.9578 | 2077.9996 | -20.13 | 172 | - | 188 | 1 | 66 | R.ATFDNPDYDKLINYYVK.Q | | 2632.3579 | 2631.3506 | 2631.4272 | -29.09 | 226 | - | 250 | 1 | --- | K.LRLLFEVAPLGLLIENAGGYSSDGK.I |  | **No match to:** 720.4036, 724.4052, 734.4100, 736.4061, 752.3973, 768.5203, 790.4085, 791.4221, 809.3938, 840.4567, 842.4854, 864.4375, 868.5361, 872.4564, 873.4495, 882.5377, 888.4676, 890.3899, 891.3968, 892.4094, 895.5118, 958.4751, 991.5695, 991.5695, 1045.5430, 1077.4678, 1087.5562, 1144.6086, 1144.6086, 1161.5857, 1215.5449, 1232.5536, 1240.5986, 1254.5443, 1259.5065, 1260.5260, 1291.6188, 1291.6188, 1295.6150, 1298.6536, 1299.6595, 1301.6301, 1307.6133, 1320.7053, 1320.7053, 1325.6387, 1329.6527, 1329.6527, 1371.6422, 1380.6515, 1385.7776, 1386.6349, 1387.6962, 1389.6157, 1391.6416, 1394.6146, 1418.6959, 1418.6959, 1434.5897, 1442.7374, 1448.7985, 1452.5917, 1452.5917, 1455.6328, 1518.7065, 1585.8257, 1613.8502, 1657.7827, 1671.7960, 1679.8127, 1688.7556, 1764.8622, 1775.8073, 1779.8036, 1791.8033, 1792.8700, 1792.8700, 1796.8555, 1803.9081, 1807.9174, 1846.8384, 1910.8365, 2007.9843, 2082.9729, 2100.9294, 2226.0803, 2400.1582, 2430.2029, 2448.2578 | | --- |  | **G38.** | [Cs3g27520.2](http://localhost/mascot/cgi/protein_view.pl?file=../data/20140815/F004020.dat&hit=1)    **Mass:** 47973    **Score:** 515    **Expect:** 1.4e-047  **Matches:** 30 | | --- | --- |  | **Observed** | **Mr(expt)** | **Mr(calc)** | **ppm** | **Start** |  | **End** | **Miss** | **Ions** | **Peptide** | | --- | --- | --- | --- | --- | --- | --- | --- | --- | --- | | 724.3912 | 723.3840 | 723.4028 | -25.98 | 196 | - | 202 | 0 | --- | K.HIQAGAK.K | | 756.4453 | 755.4380 | 755.4653 | -36.15 | 315 | - | 321 | 0 | --- | K.LNGIALR.V | | 782.4170 | 781.4097 | 781.4446 | -44.66 | 126 | - | 132 | 0 | --- | K.NASHLLK.Y | | 811.4135 | 810.4062 | 810.4348 | -35.30 | 275 | - | 281 | 0 | --- | R.LLDASHR.D | | 811.4135 | 810.4062 | 810.4348 | -35.30 | 275 | - | 281 | 0 | 35 | R.LLDASHR.D | | 833.4269 | 832.4196 | 832.4555 | -43.16 | 89 | - | 96 | 0 | --- | K.VAINGFGR.I | | 833.4269 | 832.4196 | 832.4555 | -43.16 | 89 | - | 96 | 0 | 58 | K.VAINGFGR.I | | 1043.5100 | 1042.5027 | 1042.5335 | -29.53 | 133 | - | 141 | 0 | --- | K.YDSLLGTFK.A | | 1043.5100 | 1042.5027 | 1042.5335 | -29.53 | 133 | - | 141 | 0 | 30 | K.YDSLLGTFK.A | | 1101.5382 | 1100.5309 | 1100.6264 | -86.71 | 303 | - | 312 | 0 | --- | K.AVSLVMPQLK.G + Oxidation (M) | | 1148.5527 | 1147.5454 | 1147.5795 | -29.67 | 251 | - | 260 | 0 | --- | K.VMDEELGIVK.G + Oxidation (M) | | 1165.6525 | 1164.6452 | 1164.6867 | -35.58 | 412 | - | 422 | 0 | --- | R.VVDLAHLVATK.W | | 1263.5851 | 1262.5778 | 1262.6255 | -37.76 | 340 | - | 351 | 0 | --- | K.GITAEDVNAAFR.K | | 1263.5851 | 1262.5778 | 1262.6255 | -37.76 | 340 | - | 351 | 0 | 104 | K.GITAEDVNAAFR.K | | 1289.6361 | 1288.6288 | 1288.6511 | -17.26 | 146 | - | 157 | 0 | --- | K.IVDNETISVDGK.L | | 1391.6750 | 1390.6677 | 1390.7205 | -37.92 | 340 | - | 352 | 1 | --- | K.GITAEDVNAAFRK.A | | 1391.6750 | 1390.6677 | 1390.7205 | -37.92 | 339 | - | 351 | 1 | 95 | K.KGITAEDVNAAFR.K | | 1461.7090 | 1460.7017 | 1460.7511 | -33.81 | 212 | - | 225 | 0 | --- | K.GADIPTYVVGVNEK.D | | 1599.7764 | 1598.7691 | 1598.8152 | -28.82 | 110 | - | 125 | 0 | --- | K.DSPLDVVVVNDSGGVK.N | | 1702.8389 | 1701.8316 | 1701.8785 | -27.54 | 142 | - | 157 | 1 | --- | K.ADVKIVDNETISVDGK.L | | 1727.8617 | 1726.8544 | 1726.9102 | -32.27 | 109 | - | 125 | 1 | --- | R.KDSPLDVVVVNDSGGVK.N | | 1772.7377 | 1771.7304 | 1771.7954 | -36.69 | 398 | - | 411 | 0 | --- | K.VVAWYDNEWGYSQR.V | | 1772.7377 | 1771.7304 | 1771.7954 | -36.69 | 398 | - | 411 | 0 | 55 | K.VVAWYDNEWGYSQR.V | | 1807.9529 | 1806.9456 | 1807.0091 | -35.15 | 322 | - | 338 | 0 | --- | R.VPTPNVSVVDLVVNVEK.K | | 1807.9529 | 1806.9456 | 1807.0091 | -35.15 | 322 | - | 338 | 0 | 36 | R.VPTPNVSVVDLVVNVEK.K | | 1820.7280 | 1819.7207 | 1819.9726 | -138.42 | 1 | - | 17 | 1 | --- | -.MASHSALAPSRIPAITR.I + Acetyl (Protein N-term) |  | **No match to:** 700.2744, 702.4044, 714.2577, 715.2836, 718.2629, 719.2832, 730.2618, 731.2852, 739.4280, 747.2866, 755.4190, 768.5036, 778.3951, 816.4138, 826.3456, 832.2821, 842.4798, 855.4248, 877.3699, 880.3965, 911.4370, 942.5409, 963.4958, 965.5659, 1019.5227, 1031.5483, 1035.5051, 1042.5634, 1067.6493, 1067.6493, 1084.5376, 1089.5972, 1187.6125, 1206.5905, 1212.5441, 1233.5823, 1238.6515, 1255.5801, 1277.6146, 1277.6146, 1285.5875, 1291.6361, 1302.6926, 1324.5928, 1324.5928, 1359.6359, 1379.6564, 1405.6846, 1422.6716, 1450.6364, 1450.6364, 1491.7651, 1506.7172, 1507.6426, 1519.7705, 1519.7705, 1553.7090, 1555.6484, 1571.6279, 1571.6279, 1576.8002, 1619.7937, 1620.7919, 1667.8104, 1668.8590, 1709.8296, 1741.8650, 1759.8685, 1759.8685, 1770.7649, 1775.8009, 1776.7488, 1780.7378, 1786.9063, 1788.7515, 1788.7515, 1792.7322, 1801.8322, 1802.8087, 1804.7440, 1808.7517, 1829.9022, 1885.8782, 1887.8921, 1930.8909, 1932.8325, 2114.8975, 2134.9617, 2246.0762 | | --- |  | **G29.** | [C](http://localhost/mascot/cgi/protein_view.pl?file=../data/20140415/F003321.dat&hit=1)s5g16495.1    **Mass:** 26950    **Score:** 326    **Expect:** 1.1e-028  **Matches:** 7 | | --- | --- |  | **Observed** | **Mr(expt)** | **Mr(calc)** | **ppm** | **Start** |  | **End** | **Miss** | **Ions** | **Peptide** | | --- | --- | --- | --- | --- | --- | --- | --- | --- | --- | | 980.4330 | 979.4257 | 979.4281 | -2.40 | 13 | - | 21 | 0 | --- | K.CNGTTEEVK.K | | 1049.5562 | 1048.5489 | 1048.5917 | -40.77 | 114 | - | 123 | 0 | --- | K.VAYALSQGLK.V | | 1503.7596 | 1502.7523 | 1502.7980 | -30.42 | 101 | - | 113 | 0 | --- | R.LILNELNEFVGDK.V | | 1503.7596 | 1502.7523 | 1502.7980 | -30.42 | 101 | - | 113 | 0 | 59 | R.LILNELNEFVGDK.V | | 1613.8207 | 1612.8134 | 1612.8573 | -27.18 | 192 | - | 206 | 0 | --- | K.WLLANTSPEIAAATR.I | | 1659.8551 | 1658.8478 | 1658.8991 | -30.93 | 100 | - | 113 | 1 | --- | R.RLILNELNEFVGDK.V | | 1659.8551 | 1658.8478 | 1658.8991 | -30.93 | 100 | - | 113 | 1 | 90 | R.RLILNELNEFVGDK.V | | 1695.8241 | 1694.8168 | 1694.8740 | -33.74 | 176 | - | 190 | 0 | --- | K.VATPAQAQEVHFELR.K | | 1695.8241 | 1694.8168 | 1694.8740 | -33.74 | 176 | - | 190 | 0 | 129 | K.VATPAQAQEVHFELR.K | | 1823.9198 | 1822.9125 | 1822.9690 | -30.96 | 176 | - | 191 | 1 | --- | K.VATPAQAQEVHFELRK.W | | 1823.9198 | 1822.9125 | 1822.9690 | -30.96 | 176 | - | 191 | 1 | 11 | K.VATPAQAQEVHFELRK.W | | 2842.4426 | 2841.4353 | 2841.5164 | -28.53 | 220 | - | 246 | 0 | --- | K.ELAAQPDVDGFLVGGASLKPEFIDIIK.S |  | **No match to:** 713.3944, 728.5339, 734.4683, 748.3983, 763.3815, 768.5083, 772.5571, 816.5818, 842.4860, 860.6075, 866.4262, 868.5031, 900.4402, 900.4402, 904.6356, 931.4521, 948.6601, 958.4443, 958.4443, 960.5029, 970.4448, 992.6841, 1036.7095, 1070.5511, 1080.7410, 1134.5040, 1134.5040, 1256.6040, 1269.6371, 1269.6371, 1283.6563, 1368.6476, 1374.6653, 1374.6653, 1383.6749, 1383.6749, 1388.6716, 1390.6598, 1482.7402, 1483.7510, 1525.7493, 1600.7803, 1611.8020, 1617.8127, 1617.8127, 1623.8243, 1626.7858, 1628.8062, 1629.8134, 1629.8134, 1633.8108, 1635.7980, 1639.8085, 1641.8146, 1642.8031, 1643.7946, 1645.8113, 1651.8009, 1653.7948, 1673.8705, 1677.8363, 1678.7955, 1681.8406, 1685.8110, 1687.7985, 1688.8003, 1690.7965, 1693.8195, 1694.8241, 1701.8245, 1702.7972, 1709.8402, 1709.8402, 1717.8145, 1727.8181, 1733.7821, 1745.8619, 1749.7631, 1752.8253, 1758.8328, 1763.8896, 1765.8303, 1766.8516, 1767.8442, 1769.8333, 1777.8250, 1783.8400, 1868.9318, 1868.9318, 1870.9153, 1918.8776, 1921.9722, 1921.9722, 1960.9181, 1972.9141, 1976.9396, 1983.9690, 2010.9962, 2056.9629, 2072.9724, 2462.1516 | | --- |  | **G35.** | [Cs7g32500.1](http://localhost/mascot/cgi/protein_view.pl?file=../data/20140815/F004011.dat&hit=1)    **Mass:** 26940    **Score:** 236    **Expect:** 1.1e-019  **Matches:** 14 | | --- | --- |  | **Observed** | **Mr(expt)** | **Mr(calc)** | **ppm** | **Start** |  | **End** | **Miss** | **Ions** | **Peptide** | | --- | --- | --- | --- | --- | --- | --- | --- | --- | --- | | 954.4690 | 953.4617 | 953.4760 | -14.93 | 5 | - | 12 | 0 | --- | K.FFVGGNWK.C | | 1049.5741 | 1048.5668 | 1048.5917 | -23.70 | 114 | - | 123 | 0 | --- | K.VAYALSQGLK.V | | 1695.8307 | 1694.8234 | 1694.8740 | -29.85 | 176 | - | 190 | 0 | --- | K.VATPAQAQEVHFELR.K | | 1695.8307 | 1694.8234 | 1694.8740 | -29.85 | 176 | - | 190 | 0 | 125 | K.VATPAQAQEVHFELR.K | | 1823.9180 | 1822.9107 | 1822.9690 | -31.95 | 176 | - | 191 | 1 | --- | K.VATPAQAQEVHFELRK.W | | 1823.9180 | 1822.9107 | 1822.9690 | -31.95 | 176 | - | 191 | 1 | 94 | K.VATPAQAQEVHFELRK.W | | 2842.4680 | 2841.4607 | 2841.5164 | -19.59 | 220 | - | 246 | 0 | --- | K.ELAAQPDVDGFLVGGASLKPEFIDIIK.S |  | **No match to:** 768.5170, 823.3860, 842.4895, 868.5336, 870.5283, 958.4592, 970.4606, 986.4663, 1038.4760, 1070.5549, 1099.5332, 1134.5159, 1152.5636, 1205.6774, 1251.4958, 1260.6814, 1282.6550, 1283.6713, 1309.6177, 1352.6378, 1374.6646, 1374.6646, 1382.7054, 1388.6833, 1390.6688, 1408.7131, 1481.7374, 1503.7693, 1503.7693, 1525.7607, 1531.7761, 1542.7329, 1543.7183, 1561.7716, 1598.7830, 1610.8353, 1613.8264, 1617.8187, 1617.8187, 1621.7366, 1625.8284, 1626.8209, 1628.8208, 1629.8157, 1629.8157, 1637.7721, 1641.8282, 1642.8315, 1645.8215, 1655.8472, 1658.8365, 1659.8564, 1659.8564, 1668.8281, 1673.8700, 1681.8387, 1702.8057, 1709.8541, 1745.9005, 1766.9290, 1767.9305, 1806.9147, 1821.9324, 1822.9739, 1829.9302, 1837.9385, 1839.9235, 1845.9064, 1866.9240, 1867.9512, 1868.9885, 1868.9885, 1869.9220, 1871.9120, 1880.9459, 1881.9380, 1883.9097, 1897.9247, 1903.9598, 1919.9384, 1920.9290, 1921.9830, 1921.9830, 1954.9456, 1955.9331, 1956.9329, 1960.9266, 1960.9266, 1969.9298, 1970.9252, 1971.9221, 1972.9250, 1972.9250, 1976.9346, 1988.9293, 1988.9293, 1992.9651, 1994.9569, 2004.9392, 2011.9746, 2056.9712, 2056.9712, 2118.9868, 2534.3203, 2534.3203, 2690.4053, 2690.4053, 2695.2917 | | --- |  | **G41.** | [Cs8g18560.2](http://localhost/mascot/cgi/protein_view.pl?file=../data/20140815/F004016.dat&hit=1)    **Mass:** 27223    **Score:** 428    **Expect:** 7e-039  **Matches:** 17 | | --- | --- |  | **Observed** | **Mr(expt)** | **Mr(calc)** | **ppm** | **Start** |  | **End** | **Miss** | **Ions** | **Peptide** | | --- | --- | --- | --- | --- | --- | --- | --- | --- | --- | | 954.4615 | 953.4543 | 953.4760 | -22.74 | 5 | - | 12 | 0 | --- | K.FFVGGNWK.C | | 1219.5608 | 1218.5535 | 1218.5840 | -25.03 | 195 | - | 206 | 0 | --- | K.DNVSAEVAASTR.I | | 1219.5608 | 1218.5535 | 1218.5840 | -25.03 | 195 | - | 206 | 0 | 102 | K.DNVSAEVAASTR.I | | 1317.6450 | 1316.6377 | 1316.6758 | -28.94 | 124 | - | 135 | 0 | --- | K.VIACVGETLEQR.E | | 1405.6865 | 1404.6792 | 1404.7096 | -21.65 | 136 | - | 149 | 0 | --- | R.ESGSTVAVVAEQTK.A | | 1577.8026 | 1576.7953 | 1576.7845 | 6.86 | 100 | - | 113 | 1 | --- | R.RALLNESNDFVGDK.V | | 1619.8009 | 1618.7936 | 1618.8427 | -30.31 | 176 | - | 190 | 0 | --- | K.VATPAQAQEVHAELR.K | | 1619.8009 | 1618.7936 | 1618.8427 | -30.31 | 176 | - | 190 | 0 | 101 | K.VATPAQAQEVHAELR.K | | 1646.8058 | 1645.7985 | 1645.8424 | -26.63 | 192 | - | 206 | 1 | --- | K.WLKDNVSAEVAASTR.I | | 1747.8879 | 1746.8806 | 1746.9377 | -32.65 | 176 | - | 191 | 1 | --- | K.VATPAQAQEVHAELRK.W | | 1747.8879 | 1746.8806 | 1746.9377 | -32.65 | 176 | - | 191 | 1 | 92 | K.VATPAQAQEVHAELRK.W | | 2842.4854 | 2841.4781 | 2841.5164 | -13.47 | 220 | - | 246 | 0 | --- | K.ELAAQPDVDGFLVGGASLKPEFIDIIK.S | | 2842.4854 | 2841.4781 | 2841.5164 | -13.47 | 220 | - | 246 | 0 | 90 | K.ELAAQPDVDGFLVGGASLKPEFIDIIK.S |  | **No match to:** 713.4046, 768.5160, 780.3715, 803.3876, 807.3863, 819.3760, 830.3589, 835.3756, 842.4872, 868.5285, 882.5464, 931.4603, 936.3975, 940.4786, 956.5023, 956.5023, 969.4601, 970.4570, 978.4863, 979.5150, 986.4485, 1049.5714, 1049.5714, 1071.5537, 1109.5631, 1134.5077, 1160.6085, 1174.5465, 1186.6425, 1205.6655, 1261.6741, 1274.6510, 1281.7140, 1283.6646, 1327.5210, 1341.5410, 1350.6615, 1352.6309, 1352.6309, 1356.6532, 1361.6727, 1372.6617, 1374.6577, 1374.6577, 1388.6792, 1388.6792, 1390.6724, 1396.6450, 1422.7212, 1423.7119, 1428.7017, 1448.7317, 1449.7262, 1462.7151, 1469.6874, 1469.6874, 1483.7358, 1491.6674, 1507.6420, 1582.7534, 1625.7832, 1633.8214, 1640.8560, 1641.8170, 1650.8047, 1662.8102, 1730.8760, 1746.8840, 1761.9025, 1769.8727, 1785.8271, 1809.8829, 1858.9313, 1921.9600, 1933.9745, 1935.9963, 1937.0255, 1937.0255, 1939.4182, 1965.9618, 2007.9501, 2010.9558, 2011.9435, 2022.9491, 2023.9443, 2024.9708, 2028.9692, 2028.9692, 2038.9604, 2039.9773, 2040.9646, 2040.9646, 2044.9779, 2045.9822, 2056.9656, 2056.9656, 2153.9978, 2153.9978, 2187.0408, 2285.0837, 2664.4211, 2959.4185 | | --- |  | **G4.** | [orange1.1t02542.1](http://localhost/mascot/cgi/protein_view.pl?file=../data/20140815/F003964.dat&hit=1)    **Mass:** 35354    **Score:** 1050   **Expect:** 4.4e-101  **Matches:** 25 | | --- | --- |  | **Observed** | **Mr(expt)** | **Mr(calc)** | **ppm** | **Start** |  | **End** | **Miss** | **Ions** | **Peptide** | | --- | --- | --- | --- | --- | --- | --- | --- | --- | --- | | 793.4387 | 792.4314 | 792.4534 | -27.80 | 243 | - | 248 | 0 | --- | K.WVTFIK.D | | 868.4687 | 867.4614 | 867.4338 | 31.9 | 92 | - | 99 | 0 | --- | K.YTADLSAK.F | | 907.4731 | 906.4658 | 906.4593 | 7.19 | 53 | - | 60 | 1 | --- | K.SSDCVRIK.A | | 1022.4930 | 1021.4857 | 1021.5192 | -32.81 | 249 | - | 257 | 0 | --- | K.DSPKPPPER.I | | 1022.4930 | 1021.4857 | 1021.5192 | -32.81 | 249 | - | 257 | 0 | 63 | K.DSPKPPPER.I | | 1273.5713 | 1272.5640 | 1272.6040 | -31.42 | 137 | - | 145 | 0 | --- | K.WHVFWVDER.V | | 1273.5713 | 1272.5640 | 1272.6040 | -31.42 | 137 | - | 145 | 0 | 68 | K.WHVFWVDER.V | | 1293.5647 | 1292.5574 | 1292.6765 | -92.09 | 239 | - | 248 | 1 | --- | K.ENEKWVTFIK.D | | 1377.6964 | 1376.6891 | 1376.7300 | -29.67 | 200 | - | 213 | 0 | --- | K.SNILATSAATGFPK.F | | 1377.6964 | 1376.6891 | 1376.7300 | -29.67 | 200 | - | 213 | 0 | 71 | K.SNILATSAATGFPK.F | | 1416.6768 | 1415.6695 | 1415.6681 | 1.00 | 146 | - | 157 | 1 | --- | R.VVPKDHDDSNYK.L | | 1645.7969 | 1644.7896 | 1644.8399 | -30.57 | 123 | - | 136 | 0 | --- | K.LVEPPYVDSIEWAK.W | | 1707.8649 | 1706.8576 | 1706.9203 | -36.73 | 105 | - | 121 | 0 | --- | K.GSFTVVLSGGSLIDSLR.K | | 1707.8649 | 1706.8576 | 1706.9203 | -36.73 | 105 | - | 121 | 0 | 169 | K.GSFTVVLSGGSLIDSLR.K | | 1773.8906 | 1772.8833 | 1772.9349 | -29.07 | 122 | - | 136 | 1 | --- | R.KLVEPPYVDSIEWAK.W | | 1796.9143 | 1795.9070 | 1795.9621 | -30.66 | 243 | - | 257 | 1 | --- | K.WVTFIKDSPKPPPER.I | | 1796.9143 | 1795.9070 | 1795.9621 | -30.66 | 243 | - | 257 | 1 | 67 | K.WVTFIKDSPKPPPER.I | | 1835.9567 | 1834.9494 | 1835.0153 | -35.89 | 105 | - | 122 | 1 | --- | K.GSFTVVLSGGSLIDSLRK.L | | 1835.9567 | 1834.9494 | 1835.0153 | -35.89 | 105 | - | 122 | 1 | 137 | K.GSFTVVLSGGSLIDSLRK.L | | 1863.8794 | 1862.8721 | 1862.9262 | -29.01 | 75 | - | 91 | 0 | --- | K.NVQVFDSEEDLAVSLAK.Y | | 1863.8794 | 1862.8721 | 1862.9262 | -29.01 | 75 | - | 91 | 0 | 91 | K.NVQVFDSEEDLAVSLAK.Y | | 1965.0001 | 1963.9928 | 1964.0579 | -33.11 | 103 | - | 121 | 1 | --- | K.EKGSFTVVLSGGSLIDSLR.K | | 1965.0001 | 1963.9928 | 1964.0579 | -33.11 | 103 | - | 121 | 1 | 179 | K.EKGSFTVVLSGGSLIDSLR.K | | 2708.3359 | 2707.3286 | 2707.3866 | -21.42 | 214 | - | 238 | 0 | --- | K.FDLMLLGMGPDGHIASLFPGHPLLK.E + 2 Oxidation (M) | | 2708.3359 | 2707.3286 | 2707.3866 | -21.42 | 214 | - | 238 | 0 | 104 | K.FDLMLLGMGPDGHIASLFPGHPLLK.E + 2 Oxidation (M) |  | **No match to:** 768.5145, 791.4233, 797.4413, 804.4453, 809.4338, 825.4285, 830.4379, 842.4847, 946.5476, 977.4801, 1002.6085, 1036.5122, 1044.4824, 1055.5087, 1142.5155, 1155.5693, 1176.6185, 1231.6526, 1256.5498, 1260.5588, 1263.6481, 1271.5629, 1277.5679, 1281.5673, 1285.5737, 1287.5652, 1288.5587, 1289.5679, 1289.5679, 1292.5679, 1297.5635, 1299.5608, 1301.5654, 1303.5562, 1305.5646, 1305.5646, 1309.5621, 1312.5957, 1315.5618, 1319.5657, 1320.5599, 1321.5577, 1321.5577, 1325.5925, 1328.5640, 1334.5938, 1337.5648, 1344.5789, 1394.5952, 1398.5970, 1399.6563, 1410.5979, 1415.6373, 1426.6733, 1429.6655, 1430.6764, 1430.6764, 1442.6803, 1443.6754, 1458.6801, 1482.7247, 1486.7427, 1486.7427, 1498.7163, 1514.7212, 1569.7789, 1649.7941, 1661.7986, 1677.8118, 1689.8531, 1705.8522, 1706.8528, 1723.8679, 1729.8511, 1777.8904, 1800.9048, 1812.8995, 1817.9504, 1825.8573, 1834.9119, 1845.9077, 1857.9369, 1885.8517, 1901.8378, 1946.9971, 2093.0925, 2093.0925, 2462.1814, 2644.3542, 2646.2583 | | --- |  | **G36.** | [Cs3g21280.1](http://localhost/mascot/cgi/protein_view.pl?file=../data/20140815/F004012.dat&hit=1)    **Mass:** 37623    **Score:** 408    **Expect:** 7e-037  **Matches:** 22 | | --- | --- |  | **Observed** | **Mr(expt)** | **Mr(calc)** | **ppm** | **Start** |  | **End** | **Miss** | **Ions** | **Peptide** | | --- | --- | --- | --- | --- | --- | --- | --- | --- | --- | | 703.3901 | 702.3829 | 702.4024 | -27.84 | 87 | - | 93 | 0 | --- | K.ALISSGR.T | | 964.4901 | 963.4828 | 963.5025 | -20.45 | 18 | - | 25 | 0 | --- | R.FVLNEQSK.H | | 966.4799 | 965.4726 | 965.4852 | -12.99 | 10 | - | 17 | 0 | --- | R.TDLMTITR.F + Oxidation (M) | | 1094.5205 | 1093.5132 | 1093.5404 | -24.81 | 211 | - | 220 | 0 | --- | K.IYSVNEGNAK.N | | 1247.5890 | 1246.5817 | 1246.6128 | -24.96 | 247 | - | 257 | 0 | --- | R.YIGSMVADVHR.T | | 1263.5771 | 1262.5698 | 1262.6078 | -30.04 | 247 | - | 257 | 0 | --- | R.YIGSMVADVHR.T + Oxidation (M) | | 1263.5771 | 1262.5698 | 1262.6078 | -30.04 | 247 | - | 257 | 0 | 40 | R.YIGSMVADVHR.T + Oxidation (M) | | 1376.7388 | 1375.7315 | 1375.7711 | -28.78 | 75 | - | 86 | 1 | --- | K.KLDVLSNDVFVK.A | | 1430.7052 | 1429.6979 | 1429.7428 | -31.38 | 258 | - | 269 | 0 | --- | R.TLLYGGIFMYPR.D | | 1430.7052 | 1429.6979 | 1429.7428 | -31.38 | 258 | - | 269 | 0 | (38) | R.TLLYGGIFMYPR.D | | 1446.6968 | 1445.6895 | 1445.7377 | -33.32 | 258 | - | 269 | 0 | --- | R.TLLYGGIFMYPR.D + Oxidation (M) | | 1446.6968 | 1445.6895 | 1445.7377 | -33.32 | 258 | - | 269 | 0 | 54 | R.TLLYGGIFMYPR.D + Oxidation (M) | | 1570.7452 | 1569.7379 | 1569.7899 | -33.13 | 18 | - | 30 | 1 | --- | R.FVLNEQSKHPESR.G | | 1570.7452 | 1569.7379 | 1569.7899 | -33.13 | 18 | - | 30 | 1 | 80 | R.FVLNEQSKHPESR.G | | 1685.8221 | 1684.8148 | 1684.8632 | -28.69 | 59 | - | 74 | 0 | --- | K.LIGLAGETNVQGEEQK.K | | 1685.8221 | 1684.8148 | 1684.8632 | -28.69 | 59 | - | 74 | 0 | 71 | K.LIGLAGETNVQGEEQK.K | | 1711.7986 | 1710.7913 | 1710.8352 | -25.66 | 317 | - | 331 | 0 | --- | R.SPIFLGSYDDVEEIK.A | | 1711.7986 | 1710.7913 | 1710.8352 | -25.66 | 317 | - | 331 | 0 | 77 | R.SPIFLGSYDDVEEIK.A | | 1964.0797 | 1963.0724 | 1962.9435 | 65.7 | 211 | - | 228 | 1 | --- | K.IYSVNEGNAKNWDGPTAK.Y | | 2086.8823 | 2085.8750 | 2085.9208 | -21.93 | 1 | - | 17 | 1 | --- | -.MDHEADAHRTDLMTITR.F + Acetyl (Protein N-term); 2 Oxidation (M) | | 2086.8823 | 2085.8750 | 2085.9208 | -21.93 | 1 | - | 17 | 1 | 27 | -.MDHEADAHRTDLMTITR.F + Acetyl (Protein N-term); 2 Oxidation (M) | | 2598.1963 | 2597.1890 | 2597.2182 | -11.24 | 280 | - | 302 | 0 | --- | R.VLYEVFPMSFLMEQAGGQSFTGK.Q + 2 Oxidation (M) |  | **No match to:** 700.3810, 702.3655, 709.3538, 713.4048, 715.3737, 718.3563, 724.4921, 728.3835, 734.4715, 736.4507, 740.3917, 750.5055, 756.4278, 768.5145, 811.4113, 832.2911, 833.4399, 834.4571, 840.4268, 842.4814, 850.5220, 856.4902, 864.4677, 868.5300, 875.4822, 882.5417, 893.4266, 899.4186, 912.5491, 914.4824, 924.4390, 982.5572, 1051.6750, 1151.7034, 1198.6746, 1198.6746, 1199.9464, 1200.5878, 1232.5815, 1254.5773, 1254.5773, 1285.5746, 1286.6406, 1298.6300, 1324.6003, 1380.7362, 1382.1771, 1382.1771, 1382.6971, 1398.7103, 1400.7158, 1402.6947, 1404.6973, 1414.6919, 1419.6796, 1419.6796, 1428.7090, 1431.7065, 1444.7561, 1458.7167, 1462.7029, 1462.7029, 1468.6849, 1484.6901, 1561.7355, 1566.8104, 1569.7476, 1584.7618, 1599.8120, 1638.8134, 1658.8458, 1658.8458, 1667.8159, 1680.8225, 1707.8018, 1761.9346, 1800.8903, 1805.8591, 1865.8529, 2008.9747, 2022.8896, 2109.0244, 2110.0178, 2260.0732, 2307.0637, 2333.0791, 2351.0684, 2351.0684, 2365.0847, 2365.0847, 2692.2341, 2695.1965, 2696.2017 | | --- |  | **G17.** | [Cs7g21820.2](http://localhost/mascot/cgi/protein_view.pl?file=../data/20140815/F003988.dat&hit=1)    **Mass:** 47943    **Score:** 654    **Expect:** 1.8e-061  **Matches:** 29 | | --- | --- |  | **Observed** | **Mr(expt)** | **Mr(calc)** | **ppm** | **Start** |  | **End** | **Miss** | **Ions** | **Peptide** | | --- | --- | --- | --- | --- | --- | --- | --- | --- | --- | | 740.4396 | 739.4323 | 739.4592 | -36.39 | 292 | - | 298 | 0 | --- | R.INGLPVK.E | | 756.4822 | 755.4749 | 755.5017 | -35.52 | 318 | - | 324 | 1 | --- | K.RGGLLIK.K | | 799.3707 | 798.3635 | 798.3912 | -34.76 | 263 | - | 269 | 0 | --- | K.AGVFYDK.V | | 801.4243 | 800.4170 | 800.4504 | -41.70 | 207 | - | 214 | 0 | --- | K.ALNAVASR.N | | 801.4243 | 800.4170 | 800.4504 | -41.70 | 207 | - | 214 | 0 | 59 | K.ALNAVASR.N | | 858.4241 | 857.4169 | 857.4508 | -39.56 | 243 | - | 249 | 0 | --- | K.NFHALTR.L | | 858.4241 | 857.4169 | 857.4508 | -39.56 | 243 | - | 249 | 0 | 51 | K.NFHALTR.L | | 868.5153 | 867.5080 | 867.4814 | 30.7 | 299 | - | 305 | 1 | --- | K.EVIKDHK.W | | 1060.5156 | 1059.5083 | 1059.5448 | -34.40 | 405 | - | 413 | 0 | --- | K.TEQELLAEK.K | | 1155.5135 | 1154.5062 | 1154.5608 | -47.26 | 391 | - | 399 | 0 | --- | K.DVIFDDYLR.K | | 1155.5135 | 1154.5062 | 1154.5608 | -47.26 | 391 | - | 399 | 0 | 82 | K.DVIFDDYLR.K | | 1188.5983 | 1187.5910 | 1187.6397 | -41.01 | 405 | - | 414 | 1 | --- | K.TEQELLAEKK.C | | 1210.5590 | 1209.5517 | 1209.5877 | -29.76 | 380 | - | 390 | 1 | --- | R.SKGDGDYELVK.D | | 1283.6191 | 1282.6118 | 1282.6557 | -34.24 | 391 | - | 400 | 1 | --- | K.DVIFDDYLRK.R | | 1453.6675 | 1452.6602 | 1452.7130 | -36.33 | 329 | - | 343 | 0 | --- | R.SSAASTSVSIVDAMK.S | | 1480.6776 | 1479.6703 | 1479.7245 | -36.64 | 306 | - | 317 | 0 | --- | K.WLEEGFTETIQK.R | | 1480.6776 | 1479.6703 | 1479.7245 | -36.64 | 306 | - | 317 | 0 | 56 | K.WLEEGFTETIQK.R | | 1591.8461 | 1590.8388 | 1590.8981 | -37.25 | 116 | - | 131 | 0 | --- | K.LAAGEVLGPDQPIALK.L | | 1591.8461 | 1590.8388 | 1590.8981 | -37.25 | 116 | - | 131 | 0 | 83 | K.LAAGEVLGPDQPIALK.L | | 1636.7732 | 1635.7659 | 1635.8257 | -36.51 | 306 | - | 318 | 1 | --- | K.WLEEGFTETIQKR.G | | 1636.7732 | 1635.7659 | 1635.8257 | -36.51 | 306 | - | 318 | 1 | 86 | K.WLEEGFTETIQKR.G | | 1673.8224 | 1672.8151 | 1672.8784 | -37.84 | 191 | - | 206 | 0 | --- | R.AGLLDINGQIFAEQGK.A | | 1673.8224 | 1672.8151 | 1672.8784 | -37.84 | 191 | - | 206 | 0 | 104 | R.AGLLDINGQIFAEQGK.A | | 1868.7559 | 1867.7486 | 1867.9098 | -86.29 | 326 | - | 343 | 1 | --- | K.WGRSSAASTSVSIVDAMK.S + Oxidation (M) | | 1868.7559 | 1867.7486 | 1867.9098 | -86.29 | 326 | - | 343 | 1 | --- | K.WGRSSAASTSVSIVDAMK.S + Oxidation (M) | | 2089.0293 | 2088.0220 | 2088.0860 | -30.63 | 96 | - | 115 | 0 | --- | K.MVNIAVSGAAGMIANHLLFK.L + 2 Oxidation (M) | | 2131.9424 | 2130.9351 | 2131.0110 | -35.59 | 382 | - | 399 | 1 | --- | K.GDGDYELVKDVIFDDYLR.K | | 2347.0852 | 2346.0779 | 2346.2141 | -58.03 | 138 | - | 158 | 0 | --- | R.SLQALEGVAMELEDSLFPLLR.E + Oxidation (M) | | 2347.0852 | 2346.0779 | 2346.2141 | -58.03 | 138 | - | 158 | 0 | 19 | R.SLQALEGVAMELEDSLFPLLR.E + Oxidation (M) | | 2488.1040 | 2487.0967 | 2487.1965 | -40.12 | 270 | - | 291 | 0 | --- | K.VSNMTIWGNHSTTQVPDFLNAR.I | | 2504.1177 | 2503.1104 | 2503.1914 | -32.36 | 270 | - | 291 | 0 | --- | K.VSNMTIWGNHSTTQVPDFLNAR.I + Oxidation (M) |  | **No match to:** 715.3577, 731.3481, 768.5037, 796.4111, 842.4753, 855.0228, 882.5026, 938.4689, 954.4127, 1040.5068, 1096.5256, 1115.3894, 1137.5281, 1140.5208, 1172.5927, 1177.5093, 1179.5533, 1193.5085, 1234.6216, 1277.6531, 1324.5967, 1348.6415, 1375.7188, 1395.6642, 1434.7197, 1475.6934, 1484.6757, 1493.6840, 1496.6796, 1502.6581, 1506.6903, 1510.6509, 1599.7329, 1613.8293, 1629.7920, 1634.7821, 1640.7668, 1651.7649, 1652.7627, 1655.7773, 1656.7572, 1656.7572, 1668.7523, 1695.8004, 1699.7761, 1707.7186, 1711.7808, 1723.8103, 1776.7860, 1793.9452, 1793.9452, 1823.8511, 1823.8511, 1838.8727, 1845.8344, 1884.9340, 1884.9340, 1890.7529, 1906.9166, 1912.9944, 1922.8983, 1993.8979, 2025.0066, 2108.8796, 2113.9346, 2283.1252, 2329.0601, 2395.1965, 2440.1680, 2441.0332, 2444.0938, 2445.0725, 2457.0962, 2502.1243, 2508.1128, 2508.1128, 2518.1108, 2520.1040, 2536.1030, 2564.0977, 2580.1228, 2619.2766, 2619.2766, 2631.2837 | | --- |  | **G37.** | [Cs9g10470.1](http://localhost/mascot/cgi/protein_view.pl?file=../data/20140815/F004013.dat&hit=1)    **Mass:** 35517    **Score:** 212    **Expect:** 2.8e-017  **Matches:** 15 | | --- | --- |  | **Observed** | **Mr(expt)** | **Mr(calc)** | **ppm** | **Start** |  | **End** | **Miss** | **Ions** | **Peptide** | | --- | --- | --- | --- | --- | --- | --- | --- | --- | --- | | 741.4157 | 740.4084 | 740.4181 | -13.08 | 2 | - | 7 | 1 | --- | M.AKEPVR.V + Acetyl (Protein N-term) | | 765.3687 | 764.3614 | 764.3487 | 16.6 | 95 | - | 100 | 1 | --- | K.EGMERK.D + Oxidation (M) | | 959.5072 | 958.4999 | 958.5124 | -12.97 | 143 | - | 151 | 0 | --- | K.EFAPSIPAK.N | | 1362.7036 | 1361.6963 | 1361.7265 | -22.13 | 56 | - | 67 | 0 | --- | K.MELVDAAFPLIK.G + Oxidation (M) | | 1362.7036 | 1361.6963 | 1361.7265 | -22.13 | 56 | - | 67 | 0 | 37 | K.MELVDAAFPLIK.G + Oxidation (M) | | 1649.9524 | 1648.9451 | 1648.9876 | -25.74 | 127 | - | 142 | 0 | --- | K.VLVVANPANTNALILK.E | | 1649.9524 | 1648.9451 | 1648.9876 | -25.74 | 127 | - | 142 | 0 | 63 | K.VLVVANPANTNALILK.E | | 1827.9644 | 1826.9571 | 1827.0102 | -29.03 | 164 | - | 180 | 1 | --- | R.ALGQISEKLNVQVSDVK.N | | 1892.8735 | 1891.8662 | 1891.9064 | -21.26 | 216 | - | 231 | 0 | --- | K.DDAWLNGEFITTVQQR.G | | 1950.8994 | 1949.8921 | 1949.9483 | -28.81 | 294 | - | 310 | 0 | --- | R.NGEWTIVQGLSIDEFSR.K | | 2016.0763 | 2015.0690 | 2015.1238 | -27.16 | 8 | - | 27 | 0 | --- | R.VLVTGAAGQIGYALVPMIAR.G + Oxidation (M) | | 2016.0763 | 2015.0690 | 2015.1238 | -27.16 | 8 | - | 27 | 0 | 55 | R.VLVTGAAGQIGYALVPMIAR.G + Oxidation (M) | | 2078.9795 | 2077.9722 | 2078.0433 | -34.18 | 294 | - | 311 | 1 | --- | R.NGEWTIVQGLSIDEFSRK.K | | 2362.1414 | 2361.1341 | 2361.1965 | -26.41 | 212 | - | 231 | 1 | --- | R.ELVKDDAWLNGEFITTVQQR.G | | 2590.4375 | 2589.4302 | 2589.4894 | -22.84 | 127 | - | 151 | 1 | --- | K.VLVVANPANTNALILKEFAPSIPAK.N |  | **No match to:** 713.4102, 758.4196, 768.5208, 842.4872, 850.5168, 863.4922, 868.5354, 877.4347, 877.4347, 882.5458, 893.4300, 905.4795, 911.4663, 1049.5616, 1051.6779, 1086.6127, 1180.4927, 1180.4927, 1254.5842, 1384.6653, 1405.7128, 1454.7529, 1458.7480, 1464.7406, 1509.6356, 1595.7256, 1595.7256, 1617.7230, 1632.8317, 1645.7546, 1666.8058, 1671.9215, 1739.6094, 1739.9008, 1761.9293, 1773.8490, 1781.8308, 1803.9335, 1803.9335, 1809.9342, 1879.8568, 1896.8762, 1908.8850, 1933.9382, 1936.8789, 1937.8790, 1937.8790, 1948.9044, 1949.8760, 1951.9021, 1954.8837, 1954.8837, 1961.9242, 1964.8962, 1965.8894, 1966.8927, 1966.8927, 1968.8917, 1970.8990, 1980.9221, 1982.8907, 1982.8907, 1998.9009, 2000.9110, 2004.9282, 2032.0563, 2065.9822, 2070.9302, 2077.9692, 2082.9817, 2082.9817, 2094.9866, 2098.9631, 2110.0071, 2111.0007, 2120.0073, 2121.0098, 2122.9932, 2124.0081, 2124.0081, 2136.0090, 2187.1682, 2198.0269, 2203.0056, 2237.0845, 2348.1099, 2349.1277, 2360.1042, 2366.1277, 2366.1277, 2378.1350, 2394.1143, 2498.2261, 2543.2534, 2607.2285, 2607.2285, 2695.2341, 2736.2961, 2752.2522, 3365.5740 | | --- |  | **G39.** | [orange1.1t04637.2](http://localhost/mascot/cgi/protein_view.pl?file=../data/20140815/F004014.dat&hit=1)    **Mass:** 28852    **Score:** 193    **Expect:** 2.2e-015  **Matches:** 11 | | --- | --- |  | **Observed** | **Mr(expt)** | **Mr(calc)** | **ppm** | **Start** |  | **End** | **Miss** | **Ions** | **Peptide** | | --- | --- | --- | --- | --- | --- | --- | --- | --- | --- | | 775.4045 | 774.3973 | 774.4487 | -66.42 | 143 | - | 149 | 0 | --- | K.SISELVK.G | | 864.4554 | 863.4481 | 863.4245 | 27.4 | 1 | - | 7 | 0 | --- | -.MTNLCLK.Y + Acetyl (Protein N-term) | | 1636.7793 | 1635.7720 | 1635.8290 | -34.85 | 8 | - | 22 | 0 | --- | K.YPIALNGLMLDGTSR.M + Oxidation (M) | | 1636.7793 | 1635.7720 | 1635.8290 | -34.85 | 8 | - | 22 | 0 | 38 | K.YPIALNGLMLDGTSR.M + Oxidation (M) | | 1855.9525 | 1854.9452 | 1855.0164 | -38.35 | 85 | - | 105 | 0 | --- | K.GSSVAVLGLGTVGLGAVDGAR.M | | 1855.9525 | 1854.9452 | 1855.0164 | -38.35 | 85 | - | 105 | 0 | 144 | K.GSSVAVLGLGTVGLGAVDGAR.M |  | **No match to:** 700.3700, 701.4039, 713.3976, 724.4854, 768.5083, 792.3550, 825.0717, 842.4766, 850.5120, 852.5353, 852.5353, 861.0446, 863.4471, 868.5234, 881.4524, 881.4524, 896.3603, 896.3603, 910.4141, 914.3795, 915.4534, 946.5147, 967.4898, 970.4948, 974.5099, 986.4982, 993.4322, 1021.5018, 1037.5542, 1047.5449, 1049.4883, 1049.4883, 1051.6547, 1094.5270, 1105.5367, 1150.5548, 1171.6185, 1186.5653, 1187.5953, 1194.5245, 1226.6487, 1229.5660, 1233.5747, 1261.6383, 1273.6429, 1324.5936, 1354.6399, 1356.6720, 1366.6843, 1366.6843, 1403.6412, 1405.6390, 1421.7461, 1424.7687, 1424.7687, 1437.6736, 1465.7004, 1465.7004, 1470.6641, 1482.6704, 1498.7235, 1498.7235, 1502.7821, 1532.7485, 1538.7682, 1542.6654, 1549.8350, 1549.8350, 1556.7926, 1571.8135, 1572.8048, 1578.8066, 1579.7728, 1580.7465, 1593.7638, 1606.8329, 1619.7588, 1634.7784, 1653.7593, 1668.7930, 1671.7415, 1671.7415, 1685.7673, 1693.8286, 1729.7434, 1767.7239, 1773.8851, 1789.8885, 1808.8822, 1868.7496, 1868.7496, 1882.8223, 1885.8236, 1885.8236, 1967.7899, 1992.9739, 1992.9739, 2057.0537, 2071.9087, 2075.9050, 2181.0313, 2491.9766, 2664.1753, 2695.1824, 2696.1772, 2708.1953, 2723.1882, 2749.2141, 2753.2004 | | --- |  | **G18.** | [Cs2g13550.1](http://localhost/mascot/cgi/protein_view.pl?file=../data/20140815/F003990.dat&hit=1)    **Mass:** 59814    **Score:** 1240   **Expect:** 4.4e-120  **Matches:** 21 | | --- | --- |  | **Observed** | **Mr(expt)** | **Mr(calc)** | **ppm** | **Start** |  | **End** | **Miss** | **Ions** | **Peptide** | | --- | --- | --- | --- | --- | --- | --- | --- | --- | --- | | 866.3740 | 865.3667 | 865.3930 | -30.38 | 269 | - | 275 | 0 | --- | R.EGNDLYR.E | | 975.5306 | 974.5233 | 974.5549 | -32.45 | 229 | - | 239 | 0 | --- | K.IGLFGGAGVGK.T | | 1123.5146 | 1122.5073 | 1122.5418 | -30.68 | 267 | - | 275 | 1 | --- | R.TREGNDLYR.E | | 1165.5736 | 1164.5663 | 1164.6040 | -32.33 | 187 | - | 195 | 0 | --- | K.TEHYLPIHR.E | | 1173.6172 | 1172.6099 | 1172.6554 | -38.74 | 216 | - | 225 | 0 | --- | K.VVDLLAPYQR.G | | 1173.6172 | 1172.6099 | 1172.6554 | -38.74 | 216 | - | 225 | 0 | 85 | K.VVDLLAPYQR.G | | 1262.5997 | 1261.5924 | 1261.6336 | -32.67 | 137 | - | 148 | 0 | --- | R.TIAMDGTEGLVR.G | | 1278.5946 | 1277.5873 | 1277.6286 | -32.27 | 137 | - | 148 | 0 | --- | R.TIAMDGTEGLVR.G + Oxidation (M) | | 1390.6277 | 1389.6204 | 1389.6790 | -42.13 | 253 | - | 266 | 0 | --- | K.AHGGFSVFAGVGER.T | | 1390.6277 | 1389.6204 | 1389.6790 | -42.13 | 253 | - | 266 | 0 | 97 | K.AHGGFSVFAGVGER.T | | 1399.7159 | 1398.7086 | 1398.7620 | -38.13 | 311 | - | 323 | 0 | --- | R.VGLTGLTVAEHFR.D | | 1399.7159 | 1398.7086 | 1398.7620 | -38.13 | 311 | - | 323 | 0 | 82 | R.VGLTGLTVAEHFR.D | | 1409.7649 | 1408.7576 | 1408.8038 | -32.80 | 152 | - | 165 | 0 | --- | R.VLNTGSPITVPVGR.V | | 1457.7859 | 1456.7786 | 1456.8323 | -36.85 | 240 | - | 252 | 0 | --- | K.TVLIMELINNVAK.A | | 1473.7836 | 1472.7763 | 1472.8272 | -34.56 | 240 | - | 252 | 0 | --- | K.TVLIMELINNVAK.A + Oxidation (M) | | 1492.7190 | 1491.7117 | 1491.7681 | -37.82 | 340 | - | 353 | 0 | --- | R.FTQANSEVSALLGR.I | | 1492.7190 | 1491.7117 | 1491.7681 | -37.82 | 340 | - | 353 | 0 | 141 | R.FTQANSEVSALLGR.I | | 1578.7791 | 1577.7718 | 1577.8314 | -37.76 | 183 | - | 195 | 1 | --- | K.GDLKTEHYLPIHR.E | | 1636.8271 | 1635.8198 | 1635.8767 | -34.75 | 122 | - | 136 | 0 | --- | R.LVLEVAQHMGEGVVR.T | | 1652.8235 | 1651.8162 | 1651.8716 | -33.51 | 122 | - | 136 | 0 | --- | R.LVLEVAQHMGEGVVR.T + Oxidation (M) | | 1652.8235 | 1651.8162 | 1651.8716 | -33.51 | 122 | - | 136 | 0 | 12 | R.LVLEVAQHMGEGVVR.T + Oxidation (M) | | 1852.8475 | 1851.8402 | 1851.9036 | -34.24 | 269 | - | 284 | 1 | --- | R.EGNDLYREMIESGVIK.L | | 1864.8729 | 1863.8656 | 1863.9367 | -38.12 | 324 | - | 339 | 0 | --- | R.DAEGQDVLLFIDNIFR.F | | 1864.8729 | 1863.8656 | 1863.9367 | -38.12 | 324 | - | 339 | 0 | 132 | R.DAEGQDVLLFIDNIFR.F | | 1868.8892 | 1867.8819 | 1867.8985 | -8.90 | 269 | - | 284 | 1 | --- | R.EGNDLYREMIESGVIK.L + Oxidation (M) | | 1883.8463 | 1882.8390 | 1882.8996 | -32.15 | 436 | - | 451 | 0 | --- | R.MLSPHILGEEHYNTAR.G + Oxidation (M) | | 1883.8463 | 1882.8390 | 1882.8996 | -32.15 | 436 | - | 451 | 0 | 70 | R.MLSPHILGEEHYNTAR.G + Oxidation (M) | | 2060.9802 | 2059.9729 | 2060.0426 | -33.82 | 417 | - | 435 | 0 | --- | R.QISELGIYPAVDPLDSTSR.M | | 2060.9802 | 2059.9729 | 2060.0426 | -33.82 | 417 | - | 435 | 0 | 123 | R.QISELGIYPAVDPLDSTSR.M | | 2151.0139 | 2150.0066 | 2149.9884 | 8.45 | 289 | - | 308 | 1 | --- | K.QADSKCALVYGQMNEPPGAR.A + Oxidation (M) | | 2172.0750 | 2171.0677 | 2171.1474 | -36.69 | 196 | - | 215 | 0 | --- | R.EAPAFVEQATEQQILVTGIK.V | | 2186.0610 | 2185.0537 | 2185.1379 | -38.52 | 354 | - | 374 | 0 | --- | R.IPSAVGYQPTLATDLGGLQER.I | | 2186.0610 | 2185.0537 | 2185.1379 | -38.52 | 354 | - | 374 | 0 | 203 | R.IPSAVGYQPTLATDLGGLQER.I | | 2207.0889 | 2206.0816 | 2206.1634 | -37.06 | 102 | - | 121 | 0 | --- | R.FDEGLPPILTALEVVDHSVR.L | | 2207.0889 | 2206.0816 | 2206.1634 | -37.06 | 102 | - | 121 | 0 | 144 | R.FDEGLPPILTALEVVDHSVR.L | | 2672.2869 | 2671.2796 | 2671.3738 | -35.26 | 462 | - | 485 | 1 | --- | K.NLQDIIAILGMDELSEDDKLTVAR.A | | 2688.3020 | 2687.2947 | 2687.3687 | -27.54 | 462 | - | 485 | 1 | --- | K.NLQDIIAILGMDELSEDDKLTVAR.A + Oxidation (M) | | 3245.6116 | 3244.6043 | 3244.6881 | -25.81 | 311 | - | 339 | 1 | --- | R.VGLTGLTVAEHFRDAEGQDVLLFIDNIFR.F | | 3714.7771 | 3713.7698 | 3713.8789 | -29.36 | 381 | - | 416 | 0 | --- | K.GSITSVQAIYVPADDLTDPAPATTFAHLDATTVLSR.Q | | 3842.8875 | 3841.8802 | 3841.9738 | -24.36 | 380 | - | 416 | 1 | --- | K.KGSITSVQAIYVPADDLTDPAPATTFAHLDATTVLSR.Q |  | **No match to:** 775.3836, 798.4359, 842.4807, 1195.6086, 1302.6077, 1372.6522, 1381.6855, 1382.7477, 1388.6296, 1389.6097, 1404.6564, 1412.6252, 1431.6938, 1447.6698, 1465.7184, 1491.6982, 1506.7379, 1587.8181, 1588.2666, 1588.7885, 1596.8540, 1597.6937, 1608.8729, 1613.7225, 1614.7191, 1645.6919, 1661.6892, 1661.6892, 1662.6963, 1676.7185, 1677.6938, 1678.7069, 1678.7069, 1740.8816, 1740.8816, 1749.8643, 1804.8535, 1818.9031, 1819.8433, 1835.8541, 1846.8832, 1847.8638, 1850.8696, 1878.8966, 1881.8728, 1897.8691, 2042.9724, 2043.9528, 2043.9528, 2059.9763, 2125.9990, 2142.0298, 2150.0366, 2168.0479, 2168.0479, 2184.0684, 2185.0566, 2187.5708, 2194.0405, 2198.0627, 2200.0579, 2202.0918, 2328.1682, 2592.2991, 2607.3237, 2614.2820, 2624.3064, 2649.3174, 2670.2256, 2671.3091, 2673.3015, 2695.2056, 2869.4124, 2917.4343, 2933.4478 | | --- |  | **G23.** | [Cs2g03080.1](http://localhost/mascot/cgi/protein_view.pl?file=../data/20140815/F003996.dat&hit=1)    **Mass:** 40593    **Score:** 594    **Expect:** 1.8e-055  **Matches:** 25 | | --- | --- |  | **Observed** | **Mr(expt)** | **Mr(calc)** | **ppm** | **Start** |  | **End** | **Miss** | **Ions** | **Peptide** | | --- | --- | --- | --- | --- | --- | --- | --- | --- | --- | | 726.4890 | 725.4817 | 725.4912 | -13.10 | 116 | - | 121 | 1 | --- | K.VRPVKK.V | | 840.4117 | 839.4045 | 839.4290 | -29.19 | 168 | - | 174 | 0 | --- | K.GNAYFLR.R | | 840.4117 | 839.4045 | 839.4290 | -29.19 | 168 | - | 174 | 0 | 44 | K.GNAYFLR.R | | 968.5024 | 967.4952 | 967.5239 | -29.73 | 167 | - | 174 | 1 | --- | K.KGNAYFLR.R | | 968.5024 | 967.4952 | 967.5239 | -29.73 | 167 | - | 174 | 1 | 61 | K.KGNAYFLR.R | | 978.5126 | 977.5054 | 977.5294 | -24.60 | 340 | - | 347 | 0 | --- | K.NLSIVYNR.Q | | 978.5126 | 977.5054 | 977.5294 | -24.60 | 340 | - | 347 | 0 | 57 | K.NLSIVYNR.Q | | 1015.5377 | 1014.5304 | 1014.5611 | -30.19 | 175 | - | 182 | 0 | --- | R.RPYIPVDR.F | | 1015.5377 | 1014.5304 | 1014.5611 | -30.19 | 175 | - | 182 | 0 | 50 | R.RPYIPVDR.F | | 1028.5841 | 1027.5768 | 1027.6026 | -25.10 | 122 | - | 131 | 0 | --- | K.VALVVVTGDR.G | | 1028.5841 | 1027.5768 | 1027.6026 | -25.10 | 122 | - | 131 | 0 | 62 | K.VALVVVTGDR.G | | 1086.6077 | 1085.6004 | 1085.6193 | -17.39 | 268 | - | 276 | 1 | --- | K.LTVERDVVR.T | | 1106.5887 | 1105.5814 | 1105.6244 | -38.83 | 339 | - | 347 | 1 | --- | K.KNLSIVYNR.Q | | 1119.5862 | 1118.5789 | 1118.5972 | -16.31 | 183 | - | 193 | 0 | --- | R.FLEGGSLPTAK.E | | 1156.6824 | 1155.6751 | 1155.6976 | -19.42 | 121 | - | 131 | 1 | --- | K.KVALVVVTGDR.G | | 1249.6423 | 1248.6350 | 1248.6285 | 5.24 | 132 | - | 143 | 0 | --- | R.GLCGGFNNNIIK.K | | 1374.6826 | 1373.6753 | 1373.7150 | -28.89 | 311 | - | 323 | 0 | --- | R.ALQESLASELASR.M | | 1374.6826 | 1373.6753 | 1373.7150 | -28.89 | 311 | - | 323 | 0 | 104 | R.ALQESLASELASR.M | | 1379.7505 | 1378.7432 | 1378.7708 | -19.99 | 154 | - | 166 | 0 | --- | K.TLGLDYTIISVGK.K | | 1507.8463 | 1506.8390 | 1506.8657 | -17.73 | 154 | - | 167 | 1 | --- | K.TLGLDYTIISVGKK.G | | 1544.8317 | 1543.8244 | 1543.8722 | -30.96 | 227 | - | 240 | 0 | --- | K.SDPVIHTLLPLSPR.G | | 1544.8317 | 1543.8244 | 1543.8722 | -30.96 | 227 | - | 240 | 0 | 115 | K.SDPVIHTLLPLSPR.G | | 1583.8510 | 1582.8437 | 1582.8818 | -24.04 | 353 | - | 368 | 0 | --- | K.ITGEILEIVAGADALV.- | | 2116.1042 | 2115.0969 | 2115.1477 | -23.99 | 175 | - | 193 | 1 | --- | R.RPYIPVDRFLEGGSLPTAK.E | | 3088.5249 | 3087.5176 | 3087.5540 | -11.77 | 194 | - | 220 | 1 | --- | K.EAQTIADDVFSLFVSEEVDKVELLYTK.F |  | **No match to:** 713.4103, 724.4999, 768.5188, 783.4902, 823.4121, 826.5167, 842.4647, 850.5228, 859.4600, 868.5179, 882.5521, 896.5369, 943.4838, 951.4910, 972.5440, 984.4435, 988.5262, 990.5070, 996.5787, 997.4912, 997.4912, 1006.5122, 1010.5624, 1012.5320, 1037.5343, 1049.6038, 1051.6733, 1105.5596, 1121.5652, 1121.5652, 1128.5208, 1128.5208, 1131.5930, 1141.5728, 1143.5759, 1147.5818, 1159.6063, 1174.6680, 1177.6832, 1187.6808, 1227.6665, 1306.6289, 1306.6289, 1322.6383, 1324.6320, 1326.6661, 1328.6255, 1332.6938, 1342.7906, 1342.7906, 1348.6948, 1356.7175, 1373.6671, 1387.6951, 1388.7056, 1396.6901, 1401.7319, 1411.7286, 1434.7252, 1434.7252, 1480.8164, 1480.8164, 1510.8201, 1511.7982, 1527.8158, 1542.8181, 1543.8274, 1558.8484, 1560.8406, 1566.8322, 1572.8322, 1574.8234, 1601.8564, 1605.8384, 1640.8293, 1674.8702, 1697.9617, 1754.0157, 1897.9575, 1956.9402, 1987.0168, 2210.9470, 2283.9485, 2284.9211, 2300.0303, 2301.9202, 2301.9202, 2315.9453, 2317.9436, 3003.4849 | | --- |  | **G26.** | [**Cs9g13060.1**](http://localhost/mascot/cgi/protein_view.pl?file=../data/20140815/F004031.dat&hit=1)**Mass:**20805 **Score:** 216    **Expect:**1.1e-017**Matches:**7 | | --- | --- |  | **Observed** | **Mr(expt)** | **Mr(calc)** | **ppm** | **Start** |  | **End** | **Miss** | **Ions** | **Peptide** | | --- | --- | --- | --- | --- | --- | --- | --- | --- | --- | | 1124.4900 | 1123.4827 | 1123.5146 | -28.35 | 81 | - | 90 | 0 | --- | R.FGDLTADETR.D | | 1124.4900 | 1123.4827 | 1123.5146 | -28.35 | 81 | - | 90 | 0 | 60 | R.FGDLTADETR.D | | 1634.5914 | 1633.5841 | 1633.6236 | -24.14 | 166 | - | 178 | 0 | --- | R.TMEEMAQEADEYR.S + 2 Oxidation (M) | | 1634.5914 | 1633.5841 | 1633.6236 | -24.14 | 166 | - | 178 | 0 | 32 | R.TMEEMAQEADEYR.S + 2 Oxidation (M) | | 2946.5029 | 2945.4956 | 2945.5624 | -22.68 | 109 | - | 136 | 0 | --- | K.ASSLAFAIQDGPQAGQTVPHVHIHIVPR.K | | 2946.5029 | 2945.4956 | 2945.5624 | -22.68 | 109 | - | 136 | 0 | 105 | K.ASSLAFAIQDGPQAGQTVPHVHIHIVPR.K |  | **No match to:** 728.5320, 772.5623, 816.5907, 854.0283, 860.6134, 871.0060, 886.9680, 904.6367, 948.6526, 992.6899, 1036.7263, 1299.7548, 1333.7102, 1357.7415, 1357.7415, 1373.7346, 1415.6454, 1415.6454, 1437.6296, 1453.6140, 1511.9092, 1853.9227, 1853.9227, 1870.9550, 1870.9550, 1943.0365, 1948.0425, 1948.0425, 2024.1465, 2024.1465, 2040.1479, 2040.1479, 2094.0059, 2094.0059, 2236.9734, 2236.9734, 2353.2214, 2353.2214, 2370.2212, 2370.2212, 2672.4302, 2672.4302, 2929.5313 | | --- |  | **G24.** | [Cs2g18800.1](http://localhost/mascot/cgi/protein_view.pl?file=../data/20140815/F003998.dat&hit=1)    **Mass:** 57081    **Score:** 1120   **Expect:** 4.4e-108  **Matches:** 45 | | --- | --- |  | **Observed** | **Mr(expt)** | **Mr(calc)** | **ppm** | **Start** |  | **End** | **Miss** | **Ions** | **Peptide** | | --- | --- | --- | --- | --- | --- | --- | --- | --- | --- | | 704.3760 | 703.3688 | 703.3905 | -30.84 | 386 | - | 391 | 0 | --- | R.YLPPSK.M | | 713.4006 | 712.3933 | 712.2850 | 152 | 219 | - | 223 | 0 | --- | R.MDYER.F | | 718.3201 | 717.3129 | 717.3293 | -22.95 | 255 | - | 260 | 0 | --- | K.IDEEGR.I | | 729.2741 | 728.2668 | 728.2799 | -17.97 | 219 | - | 223 | 0 | --- | R.MDYER.F + Oxidation (M) | | 734.4409 | 733.4336 | 733.4374 | -5.21 | 103 | - | 108 | 0 | --- | R.LYPLTK.K | | 754.4049 | 753.3976 | 753.4245 | -35.80 | 463 | - | 468 | 1 | --- | K.NSHIKR.A | | 771.3990 | 770.3917 | 770.4187 | -35.09 | 224 | - | 229 | 0 | --- | R.FIQAHR.E | | 854.4226 | 853.4153 | 853.4368 | -25.19 | 247 | - | 254 | 0 | --- | R.ATAFGLMK.I + Oxidation (M) | | 972.4490 | 971.4418 | 971.4600 | -18.78 | 495 | - | 502 | 0 | --- | R.ETDGYFIK.S | | 973.4789 | 972.4716 | 972.5426 | -73.02 | 307 | - | 314 | 0 | --- | K.DVMLNLLR.D | | 989.5176 | 988.5103 | 988.5375 | -27.55 | 307 | - | 314 | 0 | --- | K.DVMLNLLR.D + Oxidation (M) | | 989.5176 | 988.5103 | 988.5375 | -27.55 | 307 | - | 314 | 0 | 37 | K.DVMLNLLR.D + Oxidation (M) | | 999.5412 | 998.5339 | 998.5648 | -30.92 | 511 | - | 520 | 0 | --- | K.DALIPSGTII.- | | 1010.5219 | 1009.5146 | 1009.5379 | -23.03 | 246 | - | 254 | 1 | --- | K.RATAFGLMK.I + Oxidation (M) | | 1017.5601 | 1016.5528 | 1016.5880 | -34.55 | 413 | - | 421 | 0 | --- | K.IHHSVVGLR.S | | 1017.5601 | 1016.5528 | 1016.5880 | -34.55 | 413 | - | 421 | 0 | 69 | K.IHHSVVGLR.S | | 1032.5099 | 1031.5026 | 1031.5400 | -36.21 | 377 | - | 385 | 0 | --- | R.SAPIYTQPR.Y | | 1032.5099 | 1031.5026 | 1031.5400 | -36.21 | 377 | - | 385 | 0 | 71 | R.SAPIYTQPR.Y | | 1077.4473 | 1076.4400 | 1076.4597 | -18.25 | 157 | - | 166 | 0 | --- | R.AYASNMGGYK.N + Oxidation (M) | | 1090.5848 | 1089.5775 | 1089.6070 | -27.03 | 261 | - | 269 | 0 | --- | R.IIEFSEKPK.G | | 1090.5848 | 1089.5775 | 1089.6070 | -27.03 | 261 | - | 269 | 0 | 51 | R.IIEFSEKPK.G | | 1256.6683 | 1255.6610 | 1255.7037 | -33.97 | 111 | - | 122 | 0 | --- | R.AKPAVPLGANYR.L | | 1256.6683 | 1255.6610 | 1255.7037 | -33.97 | 111 | - | 122 | 0 | 81 | R.AKPAVPLGANYR.L | | 1270.7029 | 1269.6956 | 1269.7405 | -35.34 | 89 | - | 102 | 0 | --- | R.SVLGIILGGGAGTR.L | | 1270.7029 | 1269.6956 | 1269.7405 | -35.34 | 89 | - | 102 | 0 | 110 | R.SVLGIILGGGAGTR.L | | 1288.6144 | 1287.6071 | 1287.6419 | -26.99 | 483 | - | 494 | 0 | --- | K.IVNSDSVQEAAR.E | | 1288.6144 | 1287.6071 | 1287.6419 | -26.99 | 483 | - | 494 | 0 | 74 | K.IVNSDSVQEAAR.E | | 1346.6412 | 1345.6339 | 1345.6725 | -28.69 | 278 | - | 289 | 0 | --- | K.VDTTILGLDDER.A | | 1346.6412 | 1345.6339 | 1345.6725 | -28.69 | 278 | - | 289 | 0 | 74 | K.VDTTILGLDDER.A | | 1384.6356 | 1383.6283 | 1383.6823 | -39.01 | 366 | - | 376 | 0 | --- | K.KPIPDFSFYDR.S | | 1384.6356 | 1383.6283 | 1383.6823 | -39.01 | 366 | - | 376 | 0 | 70 | K.KPIPDFSFYDR.S | | 1406.6332 | 1405.6259 | 1405.8041 | -126.77 | 454 | - | 467 | 1 | --- | K.GSVPIGIGKNSHIK.R | | 1545.7731 | 1544.7658 | 1544.8046 | -25.10 | 278 | - | 291 | 1 | --- | K.VDTTILGLDDERAK.E | | 1625.8054 | 1624.7981 | 1624.8573 | -36.41 | 139 | - | 152 | 0 | --- | K.IYVLTQFNSASLNR.H | | 1625.8054 | 1624.7981 | 1624.8573 | -36.41 | 139 | - | 152 | 0 | 121 | K.IYVLTQFNSASLNR.H | | 1645.8611 | 1644.8538 | 1644.9086 | -33.32 | 261 | - | 274 | 1 | --- | R.IIEFSEKPKGEQLK.A | | 1733.8159 | 1732.8086 | 1732.8416 | -19.00 | 292 | - | 306 | 0 | --- | K.EMPYIASMGIYVISK.D + 2 Oxidation (M) | | 1874.8661 | 1873.8588 | 1873.9091 | -26.84 | 230 | - | 246 | 1 | --- | R.ETDADITVAALPMDEKR.A | | 1890.8655 | 1889.8582 | 1889.9040 | -24.24 | 230 | - | 246 | 1 | --- | R.ETDADITVAALPMDEKR.A + Oxidation (M) | | 1890.8655 | 1889.8582 | 1889.9040 | -24.24 | 230 | - | 246 | 1 | 63 | R.ETDADITVAALPMDEKR.A + Oxidation (M) | | 2138.9792 | 2137.9719 | 2138.0103 | -17.93 | 317 | - | 337 | 0 | --- | K.FPGANDFGSEVIPGATSIGMR.V + Oxidation (M) | | 2366.0779 | 2365.0706 | 2365.1373 | -28.17 | 315 | - | 337 | 1 | --- | R.DKFPGANDFGSEVIPGATSIGMR.V | | 2382.0857 | 2381.0784 | 2381.1322 | -22.57 | 315 | - | 337 | 1 | --- | R.DKFPGANDFGSEVIPGATSIGMR.V + Oxidation (M) | | 2382.0857 | 2381.0784 | 2381.1322 | -22.57 | 315 | - | 337 | 1 | 106 | R.DKFPGANDFGSEVIPGATSIGMR.V + Oxidation (M) | | 3074.3530 | 3073.3457 | 3073.4530 | -34.89 | 167 | - | 194 | 0 | --- | K.NEGFVEVLAAQQSPENPNWFQGTADAVR.Q |  | **No match to:** 744.3907, 768.5112, 770.3771, 790.4244, 793.3916, 806.4302, 834.3616, 842.4838, 868.5287, 882.5417, 924.4916, 925.5176, 944.4976, 978.4840, 1011.5168, 1013.4542, 1015.4973, 1016.5352, 1039.5521, 1045.5503, 1051.6807, 1054.5079, 1057.5526, 1074.5797, 1112.5728, 1128.5123, 1132.5621, 1185.6365, 1238.6611, 1252.6849, 1255.6536, 1278.6604, 1286.6597, 1292.6841, 1294.6582, 1328.6530, 1366.6575, 1367.6516, 1377.5726, 1377.5726, 1383.6748, 1398.6450, 1400.6469, 1422.6091, 1489.7264, 1601.8154, 1607.7906, 1608.7938, 1623.8037, 1639.8322, 1641.8193, 1647.8081, 1669.8010, 1786.8959, 1786.8959, 1808.8657, 1824.8448, 1825.8865, 1826.8528, 1872.8728, 1937.8755, 2074.9495, 2318.0820, 2318.0820, 2364.0608, 2365.0649, 2860.2456, 2924.2297, 3078.3577, 3090.3442 | | --- |  | **G25.** | [Cs2g18800.1](http://localhost/mascot/cgi/protein_view.pl?file=../data/20140815/F003998.dat&hit=1)    **Mass:** 57081    **Score:** 750   **Expect:** 4.4e-108  **Matches:** 37 | | --- | --- |  | **Observed** | **Mr(expt)** | **Mr(calc)** | **ppm** | **Start** |  | **End** | **Miss** | **Ions** | **Peptide** | | --- | --- | --- | --- | --- | --- | --- | --- | --- | --- | | 704.3760 | 703.3688 | 703.3905 | -30.84 | 386 | - | 391 | 0 | --- | R.YLPPSK.M | | 713.4006 | 712.3933 | 712.2850 | 152 | 219 | - | 223 | 0 | --- | R.MDYER.F | | 718.3201 | 717.3129 | 717.3293 | -22.95 | 255 | - | 260 | 0 | --- | K.IDEEGR.I | | 729.2741 | 728.2668 | 728.2799 | -17.97 | 219 | - | 223 | 0 | --- | R.MDYER.F + Oxidation (M) | | 734.4409 | 733.4336 | 733.4374 | -5.21 | 103 | - | 108 | 0 | --- | R.LYPLTK.K | | 754.4049 | 753.3976 | 753.4245 | -35.80 | 463 | - | 468 | 1 | --- | K.NSHIKR.A | | 771.3990 | 770.3917 | 770.4187 | -35.09 | 224 | - | 229 | 0 | --- | R.FIQAHR.E | | 854.4226 | 853.4153 | 853.4368 | -25.19 | 247 | - | 254 | 0 | --- | R.ATAFGLMK.I + Oxidation (M) | | 972.4490 | 971.4418 | 971.4600 | -18.78 | 495 | - | 502 | 0 | --- | R.ETDGYFIK.S | | 973.4789 | 972.4716 | 972.5426 | -73.02 | 307 | - | 314 | 0 | --- | K.DVMLNLLR.D | | 989.5176 | 988.5103 | 988.5375 | -27.55 | 307 | - | 314 | 0 | --- | K.DVMLNLLR.D + Oxidation (M) | | 989.5176 | 988.5103 | 988.5375 | -27.55 | 307 | - | 314 | 0 | 37 | K.DVMLNLLR.D + Oxidation (M) | | 999.5412 | 998.5339 | 998.5648 | -30.92 | 511 | - | 520 | 0 | --- | K.DALIPSGTII.- | | 1010.5219 | 1009.5146 | 1009.5379 | -23.03 | 246 | - | 254 | 1 | --- | K.RATAFGLMK.I + Oxidation (M) | | 1017.5601 | 1016.5528 | 1016.5880 | -34.55 | 413 | - | 421 | 0 | --- | K.IHHSVVGLR.S | | 1017.5601 | 1016.5528 | 1016.5880 | -34.55 | 413 | - | 421 | 0 | 69 | K.IHHSVVGLR.S | | 1032.5099 | 1031.5026 | 1031.5400 | -36.21 | 377 | - | 385 | 0 | --- | R.SAPIYTQPR.Y | | 1032.5099 | 1031.5026 | 1031.5400 | -36.21 | 377 | - | 385 | 0 | 71 | R.SAPIYTQPR.Y | | 1077.4473 | 1076.4400 | 1076.4597 | -18.25 | 157 | - | 166 | 0 | --- | R.AYASNMGGYK.N + Oxidation (M) | | 1090.5848 | 1089.5775 | 1089.6070 | -27.03 | 261 | - | 269 | 0 | --- | R.IIEFSEKPK.G | | 1090.5848 | 1089.5775 | 1089.6070 | -27.03 | 261 | - | 269 | 0 | 51 | R.IIEFSEKPK.G | | 1256.6683 | 1255.6610 | 1255.7037 | -33.97 | 111 | - | 122 | 0 | --- | R.AKPAVPLGANYR.L | | 1256.6683 | 1255.6610 | 1255.7037 | -33.97 | 111 | - | 122 | 0 | 81 | R.AKPAVPLGANYR.L | | 1270.7029 | 1269.6956 | 1269.7405 | -35.34 | 89 | - | 102 | 0 | --- | R.SVLGIILGGGAGTR.L | | 1270.7029 | 1269.6956 | 1269.7405 | -35.34 | 89 | - | 102 | 0 | 110 | R.SVLGIILGGGAGTR.L | | 1288.6144 | 1287.6071 | 1287.6419 | -26.99 | 483 | - | 494 | 0 | --- | K.IVNSDSVQEAAR.E | | 1288.6144 | 1287.6071 | 1287.6419 | -26.99 | 483 | - | 494 | 0 | 74 | K.IVNSDSVQEAAR.E | | 1346.6412 | 1345.6339 | 1345.6725 | -28.69 | 278 | - | 289 | 0 | --- | K.VDTTILGLDDER.A | | 1346.6412 | 1345.6339 | 1345.6725 | -28.69 | 278 | - | 289 | 0 | 74 | K.VDTTILGLDDER.A | | 1384.6356 | 1383.6283 | 1383.6823 | -39.01 | 366 | - | 376 | 0 | --- | K.KPIPDFSFYDR.S | | 1384.6356 | 1383.6283 | 1383.6823 | -39.01 | 366 | - | 376 | 0 | 70 | K.KPIPDFSFYDR.S | | 1406.6332 | 1405.6259 | 1405.8041 | -126.77 | 454 | - | 467 | 1 | --- | K.GSVPIGIGKNSHIK.R | | 1545.7731 | 1544.7658 | 1544.8046 | -25.10 | 278 | - | 291 | 1 | --- | K.VDTTILGLDDERAK.E | | 1625.8054 | 1624.7981 | 1624.8573 | -36.41 | 139 | - | 152 | 0 | --- | K.IYVLTQFNSASLNR.H | | 1625.8054 | 1624.7981 | 1624.8573 | -36.41 | 139 | - | 152 | 0 | 121 | K.IYVLTQFNSASLNR.H | | 1645.8611 | 1644.8538 | 1644.9086 | -33.32 | 261 | - | 274 | 1 | --- | R.IIEFSEKPKGEQLK.A | | 1733.8159 | 1732.8086 | 1732.8416 | -19.00 | 292 | - | 306 | 0 | --- | K.EMPYIASMGIYVISK.D + 2 Oxidation (M) | | 1874.8661 | 1873.8588 | 1873.9091 | -26.84 | 230 | - | 246 | 1 | --- | R.ETDADITVAALPMDEKR.A | | 1890.8655 | 1889.8582 | 1889.9040 | -24.24 | 230 | - | 246 | 1 | --- | R.ETDADITVAALPMDEKR.A + Oxidation (M) | | 1890.8655 | 1889.8582 | 1889.9040 | -24.24 | 230 | - | 246 | 1 | 63 | R.ETDADITVAALPMDEKR.A + Oxidation (M) | | 2138.9792 | 2137.9719 | 2138.0103 | -17.93 | 317 | - | 337 | 0 | --- | K.FPGANDFGSEVIPGATSIGMR.V + Oxidation (M) | | 2366.0779 | 2365.0706 | 2365.1373 | -28.17 | 315 | - | 337 | 1 | --- | R.DKFPGANDFGSEVIPGATSIGMR.V | | 2382.0857 | 2381.0784 | 2381.1322 | -22.57 | 315 | - | 337 | 1 | --- | R.DKFPGANDFGSEVIPGATSIGMR.V + Oxidation (M) | | 2382.0857 | 2381.0784 | 2381.1322 | -22.57 | 315 | - | 337 | 1 | 106 | R.DKFPGANDFGSEVIPGATSIGMR.V + Oxidation (M) | | 3074.3530 | 3073.3457 | 3073.4530 | -34.89 | 167 | - | 194 | 0 | --- | K.NEGFVEVLAAQQSPENPNWFQGTADAVR.Q |  | **No match to:** 744.3907, 768.5112, 770.3771, 790.4244, 793.3916, 806.4302, 834.3616, 842.4838, 868.5287, 882.5417, 924.4916, 925.5176, 944.4976, 978.4840, 1011.5168, 1013.4542, 1015.4973, 1016.5352, 1039.5521, 1045.5503, 1051.6807, 1054.5079, 1057.5526, 1074.5797, 1112.5728, 1128.5123, 1132.5621, 1185.6365, 1238.6611, 1252.6849, 1255.6536, 1278.6604, 1286.6597, 1292.6841, 1294.6582, 1328.6530, 1366.6575, 1367.6516, 1377.5726, 1377.5726, 1383.6748, 1398.6450, 1400.6469, 1422.6091, 1489.7264, 1601.8154, 1607.7906, 1608.7938, 1623.8037, 1639.8322, 1641.8193, 1647.8081, 1669.8010, 1786.8959, 1786.8959, 1808.8657, 1824.8448, 1825.8865, 1826.8528, 1872.8728, 1937.8755, 2074.9495, 2318.0820, 2318.0820, 2364.0608, 2365.0649, 2860.2456, 2924.2297, 3078.3577, 3090.3442 | | --- |   **Antioxidation and detoxification**   | **G40.** | [Cs5g32800.1](http://localhost/mascot/cgi/protein_view.pl?file=../data/20140815/F004015.dat&hit=1)    **Mass:** 23813    **Score:** 389    **Expect:** 5.6e-035  **Matches:** 20 | | --- | --- |  | **Observed** | **Mr(expt)** | **Mr(calc)** | **ppm** | **Start** |  | **End** | **Miss** | **Ions** | **Peptide** | | --- | --- | --- | --- | --- | --- | --- | --- | --- | --- | | 956.4811 | 955.4738 | 955.5015 | -28.95 | 18 | - | 25 | 0 | --- | R.VFASLYEK.E | | 956.4811 | 955.4738 | 955.5015 | -28.95 | 18 | - | 25 | 0 | 10 | R.VFASLYEK.E | | 978.4852 | 977.4780 | 977.5182 | -41.14 | 143 | - | 150 | 0 | --- | K.ILDVYEAR.L | | 1289.6106 | 1288.6033 | 1288.6524 | -38.10 | 6 | - | 17 | 0 | --- | K.VHGSVFSTATQR.V | | 1289.6106 | 1288.6033 | 1288.6524 | -38.10 | 6 | - | 17 | 0 | 100 | K.VHGSVFSTATQR.V | | 1446.6707 | 1445.6634 | 1445.7038 | -27.94 | 101 | - | 113 | 0 | --- | K.EVEALQFDPPSSK.L | | 1446.6707 | 1445.6634 | 1445.7038 | -27.94 | 101 | - | 113 | 0 | 45 | K.EVEALQFDPPSSK.L | | 1480.6860 | 1479.6787 | 1479.7167 | -25.65 | 26 | - | 37 | 0 | --- | K.ELEYELVPVDMK.A + Oxidation (M) | | 1480.6860 | 1479.6787 | 1479.7167 | -25.65 | 26 | - | 37 | 0 | 32 | K.ELEYELVPVDMK.A + Oxidation (M) | | 1523.7297 | 1522.7224 | 1522.7667 | -29.10 | 70 | - | 82 | 0 | --- | R.AITQYIAQEFPDK.G | | 1523.7297 | 1522.7224 | 1522.7667 | -29.10 | 70 | - | 82 | 0 | 60 | R.AITQYIAQEFPDK.G | | 1700.8260 | 1699.8187 | 1699.9006 | -48.15 | 2 | - | 17 | 1 | --- | M.AGIKVHGSVFSTATQR.V + Acetyl (Protein N-term) | | 2325.1206 | 2324.1133 | 2324.1801 | -28.73 | 44 | - | 64 | 0 | --- | K.EAFLSLNPFGQVPVLEHGDQK.L | | 2325.1206 | 2324.1133 | 2324.1801 | -28.73 | 44 | - | 64 | 0 | 93 | K.EAFLSLNPFGQVPVLEHGDQK.L | | 2566.2322 | 2565.2249 | 2565.3241 | -38.64 | 184 | - | 206 | 0 | --- | K.LFDARPHVSAWAADITSRPAWAK.V |  | **No match to:** 713.4012, 749.4100, 752.3590, 768.5146, 804.4368, 842.4807, 850.5109, 855.4217, 855.4217, 860.4995, 868.5288, 882.5406, 914.3762, 920.4872, 947.4646, 962.5081, 963.4836, 964.4726, 964.4726, 983.5139, 986.4738, 1021.5040, 1022.4921, 1034.4803, 1051.6722, 1064.5479, 1064.5479, 1068.5433, 1080.5458, 1080.5458, 1096.5378, 1111.5614, 1112.5581, 1118.5341, 1129.6365, 1179.5713, 1192.5928, 1216.5428, 1234.6361, 1244.5975, 1244.5975, 1254.6440, 1261.6687, 1269.5986, 1271.6141, 1272.6079, 1277.6637, 1279.6224, 1286.6112, 1287.6108, 1288.5936, 1302.6268, 1303.6212, 1311.6016, 1314.6213, 1320.5541, 1329.6506, 1410.6671, 1410.6671, 1414.6674, 1416.6803, 1426.6636, 1434.7244, 1442.6862, 1445.6846, 1468.6515, 1493.6884, 1502.6720, 1545.7083, 1773.8077, 1837.7369, 1895.7800, 1953.0360, 1959.7842, 1959.7842, 1963.9348, 1967.9448, 1971.9393, 1979.9475, 1983.9332, 1993.9130, 1995.9373, 1998.9491, 2012.9614, 2016.9606, 2016.9606, 2028.9650, 2297.0913, 2297.0913, 2307.1448, 2319.0564, 2347.0837, 2366.1150, 2570.2510, 2574.2415, 2582.2400, 2586.2339, 2695.1978, 3049.5527, 3177.6357 | | --- |  | **G34.** | [Cs7g28340.4](http://localhost/mascot/cgi/protein_view.pl?file=../data/20140815/F004010.dat&hit=1)    **Mass:** 18116    **Score:** 544    **Expect:** 1.8e-050  **Matches:** 21 | | --- | --- |  | **Observed** | **Mr(expt)** | **Mr(calc)** | **ppm** | **Start** |  | **End** | **Miss** | **Ions** | **Peptide** | | --- | --- | --- | --- | --- | --- | --- | --- | --- | --- | | 825.4356 | 824.4283 | 824.4392 | -13.17 | 78 | - | 84 | 0 | --- | K.ALDEHLK.T | | 1082.5658 | 1081.5585 | 1081.5808 | -20.58 | 136 | - | 144 | 0 | --- | K.LFALESFQK.T | | 1082.5658 | 1081.5585 | 1081.5808 | -20.58 | 136 | - | 144 | 0 | 53 | K.LFALESFQK.T | | 1113.5472 | 1112.5399 | 1112.5615 | -19.36 | 85 | - | 95 | 0 | --- | K.THGGPFIAGEK.V | | 1113.5472 | 1112.5399 | 1112.5615 | -19.36 | 85 | - | 95 | 0 | 83 | K.THGGPFIAGEK.V | | 1159.5873 | 1158.5800 | 1158.6033 | -20.12 | 18 | - | 27 | 0 | --- | K.WVADSDVIVR.I | | 1210.6516 | 1209.6443 | 1209.6757 | -25.97 | 135 | - | 144 | 1 | --- | K.KLFALESFQK.T | | 1211.6499 | 1210.6426 | 1210.6750 | -26.77 | 51 | - | 60 | 0 | --- | K.IFPSFVNFLK.S | | 1211.6499 | 1210.6426 | 1210.6750 | -26.77 | 51 | - | 60 | 0 | 40 | K.IFPSFVNFLK.S | | 1323.6538 | 1322.6465 | 1322.7446 | -74.11 | 2 | - | 13 | 1 | --- | M.EISPEGKVPVVK.F + Acetyl (Protein N-term) | | 1497.7786 | 1496.7713 | 1496.8140 | -28.49 | 107 | - | 118 | 0 | --- | K.LYHLQVALEHFK.Q | | 1497.7786 | 1496.7713 | 1496.8140 | -28.49 | 107 | - | 118 | 0 | 103 | K.LYHLQVALEHFK.Q | | 1664.7833 | 1663.7760 | 1663.8206 | -26.79 | 14 | - | 27 | 1 | --- | K.FDDKWVADSDVIVR.I | | 1664.7833 | 1663.7760 | 1663.8206 | -26.79 | 14 | - | 27 | 1 | 127 | K.FDDKWVADSDVIVR.I | | 1886.8956 | 1885.8883 | 1885.9269 | -20.44 | 61 | - | 77 | 1 | --- | K.SKDPNDGTEQALLEELK.A | | 1886.8956 | 1885.8883 | 1885.9269 | -20.44 | 61 | - | 77 | 1 | 51 | K.SKDPNDGTEQALLEELK.A | | 1919.9038 | 1918.8965 | 1918.9901 | -48.76 | 78 | - | 95 | 1 | --- | K.ALDEHLKTHGGPFIAGEK.V | | 2532.2449 | 2531.2376 | 2531.2795 | -16.54 | 28 | - | 50 | 1 | --- | R.IVEEKYPEPSLTNPPEFASLGSK.I | | 2532.2449 | 2531.2376 | 2531.2795 | -16.54 | 28 | - | 50 | 1 | --- | R.IVEEKYPEPSLTNPPEFASLGSK.I |  | **No match to:** 794.3637, 830.5081, 832.3038, 842.4896, 964.5052, 1018.5211, 1022.5048, 1129.6594, 1135.5433, 1143.5999, 1144.5830, 1147.5919, 1163.5994, 1171.5935, 1174.5768, 1175.5856, 1192.5905, 1215.6199, 1232.6351, 1233.6350, 1251.5062, 1251.6387, 1289.6492, 1312.6985, 1340.6852, 1368.7593, 1406.7671, 1475.7102, 1496.7645, 1507.8223, 1511.8025, 1519.7634, 1535.7401, 1556.7670, 1646.7996, 1650.8109, 1655.8672, 1660.8743, 1662.8186, 1663.7972, 1668.7836, 1677.8104, 1678.7876, 1679.7816, 1680.7789, 1680.7789, 1684.8000, 1696.7874, 1714.8146, 1716.8146, 1720.8075, 1728.8322, 1732.8079, 1755.8264, 1756.8273, 1771.8307, 1772.8057, 1773.8051, 1773.8051, 1788.8296, 1789.8186, 1789.8186, 1794.8369, 1795.8077, 1805.8329, 1811.7942, 1824.8318, 1826.8474, 1834.8540, 1835.8584, 1835.8584, 1839.8634, 1844.8572, 1850.8677, 1851.8613, 1851.8613, 1856.8885, 1867.8724, 1868.8765, 1873.8827, 1877.9285, 1880.9071, 1903.9435, 1921.9702, 1921.9702, 1941.8784, 2056.9827, 2056.9827, 2072.9976, 2351.2075, 2355.1919, 2366.1516, 2366.1516, 2531.2483, 2554.2053, 2691.2786 | | --- |  | **G21.** | [**Cs3g12000.1**](http://localhost/mascot/cgi/protein_view.pl?file=../data/20140815/F004029.dat&hit=1)**Mass:**15085   **Score:**83**Expect:**0.00013 **Matches:**7 | | --- | --- |  | **Observed** | **Mr(expt)** | **Mr(calc)** | **ppm** | **Start** |  | **End** | **Miss** | **Ions** | **Peptide** | | --- | --- | --- | --- | --- | --- | --- | --- | --- | --- | | 733.3467 | 732.3395 | 732.3515 | -16.40 | 135 | - | 142 | 0 | --- | K.TTGNAGGR.V | | 1179.4863 | 1178.4790 | 1178.4952 | -13.73 | 69 | - | 78 | 0 | --- | K.EHGPPEDENR.H | | 1179.4863 | 1178.4790 | 1178.4952 | -13.73 | 69 | - | 78 | 0 | 46 | K.EHGPPEDENR.H | | 1335.6776 | 1334.6703 | 1334.6830 | -9.53 | 115 | - | 127 | 0 | --- | R.AVVVHADPDDLGK.G | | 1458.8109 | 1457.8036 | 1457.8276 | -16.44 | 1 | - | 15 | 1 | --- | -.MVKAVAVLGGTEGVK.G | | 1458.8109 | 1457.8036 | 1457.8276 | -16.44 | 1 | - | 15 | 1 | --- | -.MVKAVAVLGGTEGVK.G | | 3620.8154 | 3619.8081 | 3619.7867 | 5.91 | 79 | - | 114 | 0 | --- | R.HAGDLGNVNVGDDGTATFTVVDNQIPLSGPNSIIGR.A |  | **No match to:** 712.2301, 713.4085, 724.5037, 728.5462, 734.4802, 736.5019, 750.5133, 758.5513, 768.5230, 772.5654, 792.5739, 802.5663, 816.5922, 842.4889, 850.0497, 850.0497, 850.5217, 860.6135, 860.6135, 861.0584, 866.0174, 866.0174, 868.5325, 877.0243, 877.0243, 881.9851, 881.9851, 882.5513, 893.0014, 904.6405, 904.6405, 947.4467, 947.4467, 948.6686, 958.4293, 992.6934, 992.6934, 1036.7205, 1036.7205, 1051.6860, 1080.7588, 1124.7698, 1151.7109, 1161.4741, 1161.4741, 1193.5037, 1193.5037, 1209.6458, 1259.5641, 1499.7314, 1499.7314, 1880.0034, 1880.0034 | | --- |  | **G33.** | [Cs7g29850.1](http://localhost/mascot/cgi/protein_view.pl?file=../data/20140815/F004009.dat&hit=1)    **Mass:** 25273    **Score:** 520    **Expect:** 4.4e-048  **Matches:** 19 | | --- | --- |  | **Observed** | **Mr(expt)** | **Mr(calc)** | **ppm** | **Start** |  | **End** | **Miss** | **Ions** | **Peptide** | | --- | --- | --- | --- | --- | --- | --- | --- | --- | --- | | 973.4235 | 972.4162 | 972.4552 | -40.11 | 218 | - | 225 | 0 | --- | K.YASDVYQK.E | | 976.5027 | 975.4955 | 975.5389 | -44.53 | 201 | - | 208 | 0 | --- | K.NVKPDYLK.N | | 976.5027 | 975.4955 | 975.5389 | -44.53 | 201 | - | 208 | 0 | 46 | K.NVKPDYLK.N | | 1220.5389 | 1219.5316 | 1219.5808 | -40.32 | 209 | - | 217 | 0 | --- | K.NIWNVMNWK.Y + Oxidation (M) | | 1240.5300 | 1239.5227 | 1239.7663 | -196.47 | 11 | - | 23 | 1 | --- | K.AIGLGKSVGLGLR.G | | 1240.5300 | 1239.5227 | 1239.7663 | -196.47 | 11 | - | 23 | 1 | --- | K.AIGLGKSVGLGLR.G | | 1260.6372 | 1259.6299 | 1259.6874 | -45.60 | 67 | - | 77 | 0 | --- | K.AVEQLFQALNK.V | | 1260.6372 | 1259.6299 | 1259.6874 | -45.60 | 67 | - | 77 | 0 | 71 | K.AVEQLFQALNK.V | | 1374.5935 | 1373.5862 | 1373.6476 | -44.70 | 56 | - | 66 | 0 | --- | K.HHQAYVTNYNK.A | | 1374.5935 | 1373.5862 | 1373.6476 | -44.70 | 56 | - | 66 | 0 | 65 | K.HHQAYVTNYNK.A | | 1458.7554 | 1457.7481 | 1457.8090 | -41.74 | 78 | - | 91 | 0 | --- | K.VDTSTVVQLQGAIK.F | | 1458.7554 | 1457.7481 | 1457.8090 | -41.74 | 78 | - | 91 | 0 | 107 | K.VDTSTVVQLQGAIK.F | | 1599.7134 | 1598.7061 | 1598.7743 | -42.62 | 92 | - | 105 | 0 | --- | K.FNGGGHVNHSIFWK.N | | 1627.8218 | 1626.8145 | 1626.8829 | -42.00 | 164 | - | 178 | 0 | --- | R.LVVETTANQDPLVTK.A | | 1627.8218 | 1626.8145 | 1626.8829 | -42.00 | 164 | - | 178 | 0 | 81 | R.LVVETTANQDPLVTK.A | | 1783.9175 | 1782.9102 | 1782.9840 | -41.36 | 163 | - | 178 | 1 | --- | K.RLVVETTANQDPLVTK.A | | 1783.9175 | 1782.9102 | 1782.9840 | -41.36 | 163 | - | 178 | 1 | 15 | K.RLVVETTANQDPLVTK.A | | 2700.3833 | 2699.3760 | 2699.4858 | -40.66 | 67 | - | 91 | 1 | --- | K.AVEQLFQALNKVDTSTVVQLQGAIK.F | | 2700.3833 | 2699.3760 | 2699.4858 | -40.66 | 67 | - | 91 | 1 | 72 | K.AVEQLFQALNKVDTSTVVQLQGAIK.F |  | **No match to:** 713.3929, 714.3669, 720.3808, 768.5014, 832.2745, 842.4703, 850.4977, 868.5126, 882.5325, 998.4861, 1019.4835, 1051.6636, 1080.5663, 1172.6212, 1185.6735, 1189.6047, 1222.5320, 1224.5358, 1224.5358, 1228.5334, 1236.5374, 1237.5431, 1243.5804, 1250.5299, 1252.5385, 1256.5337, 1259.5841, 1268.5315, 1282.6224, 1285.5602, 1285.5602, 1296.5651, 1298.5968, 1320.5724, 1342.5837, 1356.5801, 1357.5780, 1367.6704, 1372.5750, 1373.5852, 1386.6003, 1388.5846, 1390.5900, 1396.5796, 1400.5892, 1404.5767, 1410.6161, 1412.5667, 1416.6027, 1431.6317, 1480.7356, 1496.6942, 1502.6941, 1555.6841, 1581.7131, 1582.6987, 1585.7192, 1586.6931, 1586.6931, 1597.7177, 1598.7007, 1603.7086, 1603.7086, 1609.7025, 1613.7090, 1614.7146, 1615.7078, 1615.7078, 1620.7115, 1621.6942, 1625.6940, 1631.7139, 1637.6810, 1641.7378, 1642.8168, 1647.7081, 1649.7899, 1654.6986, 1665.7815, 1700.8905, 1779.7699, 2569.1045, 2581.1902, 2591.2593, 2593.2742, 2593.2742, 2605.2698, 2615.2058, 2627.2095, 2633.1687, 2633.1687, 2645.1829, 2661.1917, 2681.1440, 2695.1943, 3568.6804 | | --- |  | **G16.** | [Cs7g08640.2](http://localhost/mascot/cgi/protein_view.pl?file=../data/20140815/F003986.dat&hit=1)    **Mass:** 41851    **Score:** 728    **Expect:** 7e-069  **Matches:** 27 | | --- | --- |  | **Observed** | **Mr(expt)** | **Mr(calc)** | **ppm** | **Start** |  | **End** | **Miss** | **Ions** | **Peptide** | | --- | --- | --- | --- | --- | --- | --- | --- | --- | --- | | 763.4156 | 762.4083 | 762.4276 | -25.25 | 270 | - | 275 | 0 | --- | R.NLEFLK.S | | 764.3869 | 763.3796 | 763.3977 | -23.67 | 255 | - | 261 | 0 | --- | K.QVFGASR.V | | 770.4630 | 769.4557 | 769.4698 | -18.28 | 104 | - | 110 | 0 | --- | K.VTVPQVK.E | | 774.4681 | 773.4609 | 773.4800 | -24.69 | 386 | - | 392 | 0 | --- | K.VVIHPIP.- | | 792.4031 | 791.3958 | 791.4137 | -22.65 | 262 | - | 269 | 0 | --- | R.VAATSSTR.N | | 1020.5135 | 1019.5062 | 1019.5287 | -22.08 | 346 | - | 354 | 0 | --- | K.LNPYLESGK.V | | 1148.5983 | 1147.5910 | 1147.6237 | -28.46 | 345 | - | 354 | 1 | --- | K.KLNPYLESGK.V | | 1192.6240 | 1191.6167 | 1191.6499 | -27.88 | 334 | - | 344 | 0 | --- | R.FVVTSNGEVLK.K | | 1266.6241 | 1265.6168 | 1265.6503 | -26.46 | 276 | - | 287 | 0 | --- | K.SLGADLAIDYTK.D | | 1309.7242 | 1308.7169 | 1308.7514 | -26.32 | 118 | - | 130 | 1 | --- | K.VVAAALNPVDGKR.R | | 1320.7114 | 1319.7041 | 1319.7449 | -30.90 | 334 | - | 345 | 1 | --- | R.FVVTSNGEVLKK.L | | 1367.5648 | 1366.5575 | 1366.5888 | -22.91 | 166 | - | 177 | 0 | --- | K.EGDEVYGDINEK.A | | 1569.7587 | 1568.7514 | 1568.7875 | -22.99 | 86 | - | 99 | 0 | --- | K.AWLYGEYGGVDVLK.F | | 1595.7205 | 1594.7132 | 1594.9294 | -135.55 | 104 | - | 117 | 1 | --- | K.VTVPQVKEDQVLIK.V | | 1599.6982 | 1598.6909 | 1598.7576 | -41.72 | 184 | - | 197 | 0 | --- | K.QFGSLAEYTAVEER.L | | 1599.6982 | 1598.6909 | 1598.7576 | -41.72 | 184 | - | 197 | 0 | 128 | K.QFGSLAEYTAVEER.L | | 1728.8459 | 1727.8386 | 1727.9207 | -47.48 | 316 | - | 333 | 0 | --- | K.EGGTVVALTGAVTPPGFR.F | | 1728.8459 | 1727.8386 | 1727.9207 | -47.48 | 316 | - | 333 | 0 | 155 | K.EGGTVVALTGAVTPPGFR.F | | 1771.7501 | 1770.7428 | 1770.7948 | -29.35 | 163 | - | 177 | 1 | --- | K.EFKEGDEVYGDINEK.A | | 1925.0895 | 1924.0822 | 1924.1357 | -27.80 | 235 | - | 254 | 0 | --- | K.SILVLNGSGGVGSLVIQLAK.Q | | 1984.9988 | 1983.9915 | 1984.0517 | -30.35 | 137 | - | 156 | 0 | --- | K.ATDSPLPTVPGYDVAGVVVK.V | | 1984.9988 | 1983.9915 | 1984.0517 | -30.35 | 137 | - | 156 | 0 | 117 | K.ATDSPLPTVPGYDVAGVVVK.V | | 2159.9966 | 2158.9893 | 2159.0575 | -31.59 | 363 | - | 381 | 0 | --- | K.GPFPFSQVVEAFSYIETNK.A | | 2490.1743 | 2489.1670 | 2489.2802 | -45.45 | 205 | - | 227 | 0 | --- | K.NLDFVQAAGLPLAIETAYEGLER.T | | 2490.1743 | 2489.1670 | 2489.2802 | -45.45 | 205 | - | 227 | 0 | 189 | K.NLDFVQAAGLPLAIETAYEGLER.T | | 2902.4519 | 2901.4446 | 2901.5601 | -39.78 | 316 | - | 344 | 1 | --- | K.EGGTVVALTGAVTPPGFRFVVTSNGEVLK.K |  | **No match to:** 747.3571, 832.2888, 842.4874, 1115.5886, 1121.5276, 1190.5502, 1251.5955, 1286.6871, 1295.6002, 1322.6290, 1324.6089, 1328.6405, 1359.6340, 1410.5977, 1416.6605, 1429.7402, 1433.7228, 1436.6782, 1449.6775, 1465.7853, 1466.6558, 1478.7776, 1478.7776, 1485.7834, 1487.6915, 1534.7750, 1539.6693, 1542.7883, 1556.6884, 1557.6414, 1557.6414, 1564.8020, 1573.7380, 1573.7380, 1577.7688, 1579.7307, 1581.7008, 1582.6678, 1582.6678, 1596.7079, 1604.6945, 1609.8716, 1609.8716, 1613.7271, 1614.7451, 1621.6943, 1642.8074, 1642.8074, 1678.6892, 1684.8325, 1702.8568, 1709.8276, 1710.8599, 1710.8599, 1723.7800, 1726.8376, 1727.8566, 1749.8044, 1750.8375, 1824.8567, 1834.9932, 1875.9229, 1882.9199, 1947.0590, 1960.9685, 1965.9808, 1965.9808, 2006.9785, 2148.0535, 2148.0535, 2173.0400, 2181.9829, 2246.1147, 2263.0715, 2263.0715, 2379.1287, 2472.2178, 2473.1868, 2488.1694, 2504.1973, 2512.1887, 2540.0898, 2552.1602, 2553.1563, 2554.1370, 2627.1140, 2645.0798, 2645.0798, 2993.2932 | | --- |   **Chaperones and folding catalysts**   | **G15.** | [Cs5g33860.2](http://localhost/mascot/cgi/protein_view.pl?file=../data/20140815/F003984.dat&hit=1)    **Mass:** 35011    **Score:** 522    **Expect:** 2.8e-048  **Matches:** 28 | | --- | --- |  | **Observed** | **Mr(expt)** | **Mr(calc)** | **ppm** | **Start** |  | **End** | **Miss** | **Ions** | **Peptide** | | --- | --- | --- | --- | --- | --- | --- | --- | --- | --- | | 749.3804 | 748.3732 | 748.3868 | -18.19 | 73 | - | 78 | 1 | --- | K.KYEGPR.S | | 768.5194 | 767.5121 | 767.3813 | 170 | 281 | - | 287 | 1 | --- | K.GSDYAKK.E | | 821.4217 | 820.4145 | 820.4331 | -22.65 | 305 | - | 311 | 0 | --- | K.ADEFVLK.K | | 849.4240 | 848.4167 | 848.4279 | -13.22 | 19 | - | 25 | 0 | --- | K.LAPEYEK.L | | 935.4709 | 934.4636 | 934.4760 | -13.22 | 137 | - | 144 | 0 | --- | K.NLAPTYEK.V | | 939.3627 | 938.3555 | 938.3730 | -18.68 | 189 | - | 197 | 0 | --- | K.DGEEYGGGR.D | | 939.3627 | 938.3555 | 938.3730 | -18.68 | 189 | - | 197 | 0 | 59 | K.DGEEYGGGR.D | | 977.5156 | 976.5083 | 976.5229 | -14.97 | 18 | - | 25 | 1 | --- | K.KLAPEYEK.L | | 1052.4817 | 1051.4744 | 1051.4822 | -7.39 | 236 | - | 245 | 0 | --- | K.EFVAASGDEK.K | | 1068.5499 | 1067.5426 | 1067.5652 | -21.11 | 172 | - | 181 | 0 | --- | K.YGVSGFPTLK.F | | 1068.5499 | 1067.5426 | 1067.5652 | -21.11 | 172 | - | 181 | 0 | 60 | K.YGVSGFPTLK.F | | 1238.5129 | 1237.5056 | 1237.5323 | -21.58 | 186 | - | 197 | 1 | --- | K.GNKDGEEYGGGR.D | | 1238.5129 | 1237.5056 | 1237.5323 | -21.58 | 186 | - | 197 | 1 | 104 | K.GNKDGEEYGGGR.D | | 1439.8007 | 1438.7934 | 1438.7528 | 28.2 | 255 | - | 268 | 1 | --- | R.GVEVLEGSTARHGK.I | | 1439.8007 | 1438.7934 | 1438.7528 | 28.2 | 255 | - | 268 | 1 | --- | R.GVEVLEGSTARHGK.I | | 1455.6689 | 1454.6616 | 1454.6929 | -21.51 | 198 | - | 209 | 0 | --- | R.DLEDFVSFINEK.C | | 1683.8329 | 1682.8256 | 1682.8457 | -11.92 | 53 | - | 66 | 0 | --- | K.YGVQGYPTIQWFPK.G | | 1743.9507 | 1742.9434 | 1742.9778 | -19.73 | 218 | - | 235 | 0 | --- | K.GQLTSTAGIVASLDALVK.E | | 1743.9507 | 1742.9434 | 1742.9778 | -19.73 | 218 | - | 235 | 0 | 130 | K.GQLTSTAGIVASLDALVK.E | | 2376.0313 | 2375.0240 | 2375.0553 | -13.19 | 189 | - | 209 | 1 | --- | K.DGEEYGGGRDLEDFVSFINEK.C | | 2442.2839 | 2441.2766 | 2441.3054 | -11.77 | 97 | - | 119 | 0 | --- | K.IAAVPSNVVVLTADNFDEIVLDK.S | | 2442.2839 | 2441.2766 | 2441.3054 | -11.77 | 97 | - | 119 | 0 | 94 | K.IAAVPSNVVVLTADNFDEIVLDK.S |  | **No match to:** 702.4105, 703.3276, 713.4097, 734.4153, 752.4210, 789.3912, 832.2991, 842.4896, 846.4662, 856.4708, 868.5372, 882.5465, 889.4633, 894.3564, 902.4901, 912.4966, 953.3913, 958.5530, 966.4839, 996.6223, 1033.5640, 1051.6797, 1078.5691, 1090.5410, 1092.5067, 1121.5559, 1128.5265, 1128.5265, 1145.6138, 1148.5511, 1155.5475, 1162.5573, 1227.6693, 1263.6322, 1294.6754, 1324.6143, 1332.6226, 1345.6812, 1374.6980, 1387.6886, 1409.6499, 1410.7000, 1410.7000, 1433.7776, 1454.7416, 1457.7081, 1457.7081, 1469.7084, 1471.7380, 1471.7380, 1477.6589, 1479.6903, 1480.7827, 1483.7462, 1483.7462, 1493.6495, 1522.6945, 1522.6945, 1574.8209, 1610.8240, 1666.8494, 1687.8143, 1687.8143, 1698.8591, 1699.8287, 1705.8096, 1707.7911, 1709.7996, 1715.8315, 1765.9219, 1776.0269, 1779.8350, 1779.8350, 1794.7831, 1798.7875, 1801.8137, 1810.7827, 1816.7694, 1863.8782, 1864.8735, 1865.8571, 1865.8571, 1879.8696, 1880.8441, 1884.8229, 1887.8311, 1896.8435, 1987.0134, 2095.9475, 2207.0007, 2364.2727, 2401.2476, 2464.2253 | | --- |  | **G12.** | [Cs4g07030.2](http://localhost/mascot/cgi/protein_view.pl?file=../data/20140815/F003980.dat&hit=1)    **Mass:** 26576    **Score:** 874    **Expect:** 1.8e-083  **Matches:** 20 | | --- | --- |  | **Observed** | **Mr(expt)** | **Mr(calc)** | **ppm** | **Start** |  | **End** | **Miss** | **Ions** | **Peptide** | | --- | --- | --- | --- | --- | --- | --- | --- | --- | --- | | 814.3481 | 813.3409 | 813.3657 | -30.54 | 226 | - | 232 | 0 | --- | K.YAGNDFK.G | | 970.4966 | 969.4894 | 969.5243 | -36.06 | 158 | - | 165 | 0 | --- | K.DLKPLNDR.V | | 970.4966 | 969.4894 | 969.5243 | -36.06 | 158 | - | 165 | 0 | 61 | K.DLKPLNDR.V | | 1149.5820 | 1148.5747 | 1148.6190 | -38.51 | 58 | - | 67 | 0 | --- | K.YTSIKPLGDR.V | | 1149.5820 | 1148.5747 | 1148.6190 | -38.51 | 58 | - | 67 | 0 | 65 | K.YTSIKPLGDR.V | | 1166.5337 | 1165.5264 | 1165.5727 | -39.74 | 233 | - | 243 | 0 | --- | K.GSDGTNYIALR.A | | 1166.5337 | 1165.5264 | 1165.5727 | -39.74 | 233 | - | 243 | 0 | 84 | K.GSDGTNYIALR.A | | 1574.7313 | 1573.7240 | 1573.7723 | -30.67 | 144 | - | 157 | 0 | --- | R.EDDVVGILETDEIK.D | | 1574.7313 | 1573.7240 | 1573.7723 | -30.67 | 144 | - | 157 | 0 | 94 | R.EDDVVGILETDEIK.D | | 1621.8124 | 1620.8051 | 1620.8545 | -30.47 | 211 | - | 225 | 0 | --- | R.KPLSIAPGNTVMYSK.Y + Oxidation (M) | | 1621.8124 | 1620.8051 | 1620.8545 | -30.47 | 211 | - | 225 | 0 | 102 | R.KPLSIAPGNTVMYSK.Y + Oxidation (M) | | 1647.8754 | 1646.8681 | 1646.9243 | -34.12 | 112 | - | 126 | 0 | --- | K.LDISVKPGTQVIYSK.Y | | 1647.8754 | 1646.8681 | 1646.9243 | -34.12 | 112 | - | 126 | 0 | 98 | K.LDISVKPGTQVIYSK.Y | | 1889.9146 | 1888.9073 | 1888.9629 | -29.43 | 170 | - | 188 | 0 | --- | K.VAEAEETTAGGLLLTEASK.E | | 1916.9020 | 1915.8947 | 1916.0480 | -79.97 | 13 | - | 30 | 1 | --- | K.VPARSLTSFDGLRPSSVK.F | | 1917.9070 | 1916.8997 | 1916.9744 | -38.97 | 127 | - | 143 | 0 | --- | K.YAGTELEFNGANHLILR.E | | 1917.9070 | 1916.8997 | 1916.9744 | -38.97 | 127 | - | 143 | 0 | 157 | K.YAGTELEFNGANHLILR.E | | 1961.8962 | 1960.8889 | 1960.9279 | -19.87 | 226 | - | 243 | 1 | --- | K.YAGNDFKGSDGTNYIALR.A | | 2526.2117 | 2525.2044 | 2525.2861 | -32.33 | 144 | - | 165 | 1 | --- | R.EDDVVGILETDEIKDLKPLNDR.V | | 2526.2117 | 2525.2044 | 2525.2861 | -32.33 | 144 | - | 165 | 1 | 143 | R.EDDVVGILETDEIKDLKPLNDR.V |  | **No match to:** 700.3563, 713.3975, 768.5113, 799.4805, 832.2888, 842.4816, 850.4881, 855.4825, 868.5238, 882.5402, 895.6104, 907.4718, 925.4818, 952.4876, 953.4837, 992.4998, 1037.5444, 1042.5813, 1051.6704, 1078.5602, 1123.6061, 1130.5039, 1131.5714, 1148.5387, 1164.5533, 1165.5302, 1179.6614, 1188.5254, 1204.5323, 1239.5670, 1419.7791, 1432.7599, 1556.7332, 1557.7985, 1573.7195, 1596.7177, 1612.8026, 1629.8430, 1643.7900, 1646.8525, 1669.8469, 1685.8165, 1795.8604, 1809.8715, 1847.0081, 1847.0081, 1868.9851, 1881.9080, 1885.9030, 1886.8866, 1886.8866, 1899.9242, 1900.8984, 1902.8953, 1903.8904, 1903.8904, 1910.9004, 1911.9014, 1915.8977, 1924.9484, 1925.8821, 1931.9139, 1934.9121, 1939.8828, 1941.8639, 1959.8687, 1960.9135, 1962.9044, 1974.9020, 1977.9371, 2056.9744, 2057.9905, 2217.0791, 2217.0791, 2231.0859, 2233.0850, 2234.0615, 2235.0774, 2235.0774, 2237.6230, 2248.1050, 2249.0994, 2249.0994, 2257.0813, 2508.2439, 2525.2078, 2573.3059, 2609.3059, 2627.3022, 2627.3022, 2649.2556, 2755.3743, 3774.0186, 3776.8022, 3837.9519 | | --- |  | **G11.** | [Cs7g29010.1](http://localhost/mascot/cgi/protein_view.pl?file=../data/20140815/F003979.dat&hit=1)    **Mass:** 70947    **Score:** 794    **Expect:** 1.8e-075  **Matches:** 5 | | --- | --- |  | **Observed** | **Mr(expt)** | **Mr(calc)** | **ppm** | **Start** |  | **End** | **Miss** | **Ions** | **Peptide** | | --- | --- | --- | --- | --- | --- | --- | --- | --- | --- | | 758.3577 | 757.3505 | 757.3719 | -28.26 | 258 | - | 264 | 0 | --- | K.DISGNPR.A | | 1183.6073 | 1182.6000 | 1182.6397 | -33.54 | 465 | - | 475 | 0 | --- | K.FELSGIPPAPR.G | | 1228.5863 | 1227.5790 | 1227.6207 | -33.98 | 29 | - | 39 | 0 | --- | R.VEIIANDQGNR.T | | 1228.5863 | 1227.5790 | 1227.6207 | -33.98 | 29 | - | 39 | 0 | 54 | R.VEIIANDQGNR.T | | 1231.6038 | 1230.5965 | 1230.6391 | -34.56 | 164 | - | 175 | 0 | --- | K.DAGVIAGLNVMR.I + Oxidation (M) | | 1278.6437 | 1277.6364 | 1277.6227 | 10.8 | 243 | - | 252 | 0 | --- | R.MVNHFVQEFK.R | | 1294.5864 | 1293.5791 | 1293.6176 | -29.74 | 243 | - | 252 | 0 | --- | R.MVNHFVQEFK.R + Oxidation (M) | | 1294.5864 | 1293.5791 | 1293.6176 | -29.74 | 243 | - | 252 | 0 | 32 | R.MVNHFVQEFK.R + Oxidation (M) | | 1313.5757 | 1312.5684 | 1312.6122 | -33.31 | 308 | - | 317 | 0 | --- | R.FEELNMDLFR.K | | 1329.5680 | 1328.5607 | 1328.6071 | -34.88 | 308 | - | 317 | 0 | --- | R.FEELNMDLFR.K + Oxidation (M) | | 1329.5680 | 1328.5607 | 1328.6071 | -34.88 | 308 | - | 317 | 0 | 56 | R.FEELNMDLFR.K + Oxidation (M) | | 1356.5645 | 1355.5572 | 1355.6140 | -41.86 | 80 | - | 91 | 1 | --- | R.RFSDASVQGDMK.L + Oxidation (M) | | 1358.5768 | 1357.5695 | 1357.6084 | -28.66 | 546 | - | 556 | 0 | --- | K.NALENYAYNMR.N | | 1374.5638 | 1373.5565 | 1373.6033 | -34.09 | 546 | - | 556 | 0 | --- | K.NALENYAYNMR.N + Oxidation (M) | | 1374.5638 | 1373.5565 | 1373.6033 | -34.09 | 546 | - | 556 | 0 | 38 | K.NALENYAYNMR.N + Oxidation (M) | | 1426.7150 | 1425.7077 | 1425.7576 | -35.00 | 335 | - | 348 | 0 | --- | K.STVHDVVLVGGSTR.I | | 1426.7150 | 1425.7077 | 1425.7576 | -35.00 | 335 | - | 348 | 0 | 66 | K.STVHDVVLVGGSTR.I | | 1436.7054 | 1435.6981 | 1435.7460 | -33.33 | 352 | - | 363 | 0 | --- | K.VQQLLQDFFNGK.E | | 1436.7054 | 1435.6981 | 1435.7460 | -33.33 | 352 | - | 363 | 0 | 44 | K.VQQLLQDFFNGK.E | | 1450.6781 | 1449.6708 | 1449.7187 | -33.03 | 243 | - | 253 | 1 | --- | R.MVNHFVQEFKR.K + Oxidation (M) | | 1473.6298 | 1472.6225 | 1472.6784 | -37.91 | 40 | - | 52 | 0 | --- | R.TTPSYVGFTDTER.L | | 1473.6298 | 1472.6225 | 1472.6784 | -37.91 | 40 | - | 52 | 0 | 113 | R.TTPSYVGFTDTER.L | | 1540.7001 | 1539.6928 | 1539.7504 | -37.37 | 306 | - | 317 | 1 | --- | R.ARFEELNMDLFR.K | | 1556.6967 | 1555.6894 | 1555.7453 | -35.91 | 306 | - | 317 | 1 | --- | R.ARFEELNMDLFR.K + Oxidation (M) | | 1556.6967 | 1555.6894 | 1555.7453 | -35.91 | 306 | - | 317 | 1 | 40 | R.ARFEELNMDLFR.K + Oxidation (M) | | 1581.7681 | 1580.7608 | 1580.8120 | -32.35 | 117 | - | 130 | 0 | --- | K.QFAAEEISSMVLIK.M + Oxidation (M) | | 1659.8342 | 1658.8269 | 1658.8774 | -30.42 | 160 | - | 175 | 1 | --- | R.QATKDAGVIAGLNVMR.I + Oxidation (M) | | 1665.7532 | 1664.7459 | 1664.7828 | -22.16 | 60 | - | 74 | 0 | --- | K.NQVAMNPTNTVFDAK.R + Oxidation (M) | | 1665.7532 | 1664.7459 | 1664.7828 | -22.16 | 60 | - | 74 | 0 | --- | K.NQVAMNPTNTVFDAK.R + Oxidation (M) | | 1675.6641 | 1674.6568 | 1674.7234 | -39.76 | 227 | - | 242 | 0 | --- | K.ATAGDTHLGGEDFDNR.M | | 1680.7670 | 1679.7597 | 1679.8267 | -39.89 | 145 | - | 159 | 0 | --- | K.NAVVTVPAYFNDSQR.Q | | 1680.7670 | 1679.7597 | 1679.8267 | -39.89 | 145 | - | 159 | 0 | 105 | K.NAVVTVPAYFNDSQR.Q | | 1688.8420 | 1687.8347 | 1687.8967 | -36.73 | 97 | - | 112 | 0 | --- | K.VIAGPADKPMIGVNYK.G + Oxidation (M) | | 1787.9312 | 1786.9239 | 1786.9828 | -32.97 | 176 | - | 192 | 1 | --- | R.IINEPTAAAIAYGLDKK.A | | 2132.0396 | 2131.0323 | 2131.0983 | -30.96 | 97 | - | 116 | 1 | --- | K.VIAGPADKPMIGVNYKGEEK.Q + Oxidation (M) | | 2601.2131 | 2600.2058 | 2600.2718 | -25.36 | 368 | - | 393 | 0 | --- | K.NINPDEAVAYGAAVQAAILSGEGNEK.V | | 2658.1653 | 2657.1580 | 2657.2609 | -38.72 | 431 | - | 453 | 0 | --- | K.EQVFSTYSDNQPGVLIQVYEGER.T | | 2658.1653 | 2657.1580 | 2657.2609 | -38.72 | 431 | - | 453 | 0 | 142 | K.EQVFSTYSDNQPGVLIQVYEGER.T | | 3025.3813 | 3024.3740 | 3024.4815 | -35.55 | 279 | - | 305 | 0 | --- | R.TLSSTAQTTIEIDSLYEGIDFYSTITR.A |  | **No match to:** 713.3842, 768.5101, 796.4509, 842.4806, 868.5252, 870.4389, 1025.5154, 1165.5811, 1166.5541, 1167.6019, 1169.5959, 1169.5959, 1179.5728, 1186.5901, 1196.5936, 1197.6190, 1197.6190, 1211.6102, 1213.6005, 1219.5999, 1260.5719, 1263.6409, 1264.6687, 1266.5768, 1277.6487, 1296.5809, 1299.5989, 1300.6073, 1309.6295, 1310.5797, 1316.6785, 1316.6785, 1323.6167, 1328.5503, 1343.5870, 1386.6686, 1412.7010, 1416.7343, 1458.6766, 1472.6289, 1479.7065, 1487.6526, 1491.7799, 1493.6802, 1495.6943, 1500.7484, 1500.7484, 1508.6895, 1512.6819, 1523.7094, 1535.7307, 1536.7087, 1538.7014, 1553.6844, 1555.6803, 1564.7401, 1601.7478, 1624.8513, 1657.6910, 1662.7701, 1663.7516, 1674.6514, 1687.7280, 1696.7756, 1697.6937, 1702.7522, 1803.8258, 1861.8779, 1906.9371, 2066.9539, 2401.0808, 2512.1162, 2592.1179, 2640.1721, 2657.1455, 2691.1863 | | --- |  | **G19.** | [orange1.1t01459.2](http://localhost/mascot/cgi/protein_view.pl?file=../data/20140815/F003992.dat&hit=1)    **Mass:** 58188    **Score:** 632    **Expect:** 2.8e-059  **Matches:** 46 | | --- | --- |  | **Observed** | **Mr(expt)** | **Mr(calc)** | **ppm** | **Start** |  | **End** | **Miss** | **Ions** | **Peptide** | | --- | --- | --- | --- | --- | --- | --- | --- | --- | --- | | 842.4731 | 841.4659 | 841.4909 | -29.76 | 491 | - | 498 | 0 | --- | K.SGIIDPLK.V | | 847.3707 | 846.3635 | 846.3984 | -41.30 | 309 | - | 316 | 0 | --- | K.APGFGENR.K | | 847.3707 | 846.3635 | 846.3984 | -41.30 | 309 | - | 316 | 0 | 53 | K.APGFGENR.K | | 868.5156 | 867.5084 | 867.4160 | 106 | 129 | - | 136 | 0 | --- | R.AIFTEGCK.S | | 941.5748 | 940.5676 | 940.6069 | -41.85 | 267 | - | 274 | 1 | --- | R.VLELALKR.Q | | 941.5748 | 940.5676 | 940.6069 | -41.85 | 267 | - | 274 | 1 | 55 | R.VLELALKR.Q | | 972.5467 | 971.5395 | 971.5764 | -37.98 | 258 | - | 266 | 0 | --- | K.ISNLTAVVR.V | | 975.4642 | 974.4570 | 974.4934 | -37.36 | 309 | - | 317 | 1 | --- | K.APGFGENRK.A | | 975.4642 | 974.4570 | 974.4934 | -37.36 | 309 | - | 317 | 1 | 42 | K.APGFGENRK.A | | 1023.4755 | 1022.4682 | 1022.5033 | -34.27 | 428 | - | 436 | 0 | --- | K.LSTANFDQK.I | | 1068.5336 | 1067.5263 | 1067.5611 | -32.60 | 203 | - | 212 | 0 | --- | K.EGVITIHDGK.T | | 1093.5830 | 1092.5757 | 1092.5307 | 41.2 | 343 | - | 352 | 0 | --- | K.VNLDMLGTCK.K | | 1100.6343 | 1099.6270 | 1099.6713 | -40.29 | 257 | - | 266 | 1 | --- | K.KISNLTAVVR.V | | 1100.6343 | 1099.6270 | 1099.6713 | -40.29 | 257 | - | 266 | 1 | 60 | K.KISNLTAVVR.V | | 1196.6901 | 1195.6828 | 1195.7288 | -38.48 | 437 | - | 447 | 0 | --- | K.IGVQIIQNALK.T | | 1203.5773 | 1202.5700 | 1202.6255 | -46.13 | 393 | - | 403 | 1 | --- | K.DRVTDALNATK.A | | 1210.7091 | 1209.7018 | 1209.7445 | -35.27 | 491 | - | 501 | 1 | --- | K.SGIIDPLKVIR.T | | 1210.7091 | 1209.7018 | 1209.7445 | -35.27 | 491 | - | 501 | 1 | 52 | K.SGIIDPLKVIR.T | | 1218.6719 | 1217.6646 | 1217.5776 | 71.5 | 359 | - | 370 | 0 | --- | K.DDTVILDGAGDK.K | | 1267.5314 | 1266.5241 | 1266.5696 | -35.94 | 137 | - | 148 | 0 | --- | K.SVAAGMNAMDLR.R + 2 Oxidation (M) | | 1302.5741 | 1301.5668 | 1301.6438 | -59.11 | 1 | - | 11 | 1 | --- | -.MYRFASSLASK.A + Acetyl (Protein N-term) | | 1327.6445 | 1326.6372 | 1326.6932 | -42.19 | 68 | - | 79 | 0 | --- | R.NVVIEQSWGAPK.V | | 1331.6497 | 1330.6424 | 1330.7166 | -55.73 | 188 | - | 199 | 1 | --- | R.EIGELIAKAMEK.V | | 1352.6997 | 1351.6924 | 1351.7460 | -39.61 | 200 | - | 212 | 1 | --- | K.VGKEGVITIHDGK.T | | 1391.6492 | 1390.6419 | 1390.6809 | -28.03 | 137 | - | 149 | 1 | --- | K.SVAAGMNAMDLRR.G | | 1407.6346 | 1406.6273 | 1406.6758 | -34.48 | 137 | - | 149 | 1 | --- | K.SVAAGMNAMDLRR.G + Oxidation (M) | | 1423.6279 | 1422.6206 | 1422.6707 | -35.23 | 137 | - | 149 | 1 | --- | K.SVAAGMNAMDLRR.G + 2 Oxidation (M) | | 1430.6748 | 1429.6675 | 1429.7242 | -39.61 | 229 | - | 240 | 0 | --- | R.GYISPYFITNQK.N | | 1430.6748 | 1429.6675 | 1429.7242 | -39.61 | 229 | - | 240 | 0 | 51 | R.GYISPYFITNQK.N | | 1447.7216 | 1446.7143 | 1446.7752 | -42.09 | 150 | - | 163 | 0 | --- | R.GITMAVDAVVTNLK.S + Oxidation (M) | | 1540.7258 | 1539.7185 | 1539.7490 | -19.82 | 213 | - | 225 | 0 | --- | K.TLYNELEVVEGMK.L + Oxidation (M) | | 1586.6669 | 1585.6596 | 1585.8749 | -135.74 | 45 | - | 59 | 1 | --- | R.ALMLKGVEELADAVK.V | | 1603.8184 | 1602.8111 | 1602.8763 | -40.68 | 149 | - | 163 | 1 | --- | R.RGITMAVDAVVTNLK.S + Oxidation (M) | | 1638.7922 | 1637.7849 | 1637.5466 | 146 | 530 | - | 546 | 0 | --- | K.EAPGGMGGMGGMGGMDY.- + 4 Oxidation (M) | | 1814.8716 | 1813.8643 | 1813.9363 | -39.66 | 226 | - | 240 | 1 | --- | K.LDRGYISPYFITNQK.N | | 1884.9733 | 1883.9660 | 1884.0356 | -36.95 | 404 | - | 423 | 0 | --- | K.AAVEEGIVPGGGVALLYAAK.E | | 1889.9747 | 1888.9674 | 1889.0371 | -36.87 | 448 | - | 467 | 0 | --- | K.TPVHTIAANAGVEGAVVVGK.L | | 1908.9150 | 1907.9077 | 1907.9662 | -30.67 | 213 | - | 228 | 1 | --- | K.TLYNELEVVEGMKLDR.G | | 1924.8964 | 1923.8891 | 1923.9612 | -37.44 | 213 | - | 228 | 1 | --- | K.TLYNELEVVEGMKLDR.G + Oxidation (M) | | 1924.8964 | 1923.8891 | 1923.9612 | -37.44 | 213 | - | 228 | 1 | 41 | K.TLYNELEVVEGMKLDR.G + Oxidation (M) | | 2092.9551 | 2091.9478 | 2092.0106 | -30.02 | 168 | - | 187 | 0 | --- | R.MISTSEEIAQVGTISANGER.E | | 2108.9392 | 2107.9319 | 2108.0055 | -34.92 | 168 | - | 187 | 0 | --- | R.MISTSEEIAQVGTISANGER.E + Oxidation (M) | | 2535.3301 | 2534.3228 | 2534.4319 | -43.04 | 275 | - | 297 | 0 | --- | R.QRPLLIVAEDVESEALATLILNK.L | | 2603.1353 | 2602.1280 | 2602.2108 | -31.83 | 468 | - | 490 | 1 | --- | K.LLEQDNTDLGYDAAKGEYVDMVK.S + Oxidation (M) | | 2962.4089 | 2961.4016 | 2961.4964 | -32.01 | 168 | - | 195 | 1 | --- | R.MISTSEEIAQVGTISANGEREIGELIAK.A + Oxidation (M) |  | **No match to:** 704.2783, 705.2993, 713.3967, 768.5066, 850.5079, 855.0237, 861.0388, 882.5314, 885.3789, 925.4120, 948.5561, 963.5543, 968.5204, 1051.6698, 1151.7041, 1179.5601, 1315.6484, 1319.5884, 1319.5884, 1325.6245, 1332.7189, 1332.7189, 1341.6056, 1343.6385, 1359.6354, 1394.6517, 1405.6371, 1418.6658, 1445.6453, 1452.6576, 1465.6968, 1468.6331, 1475.6937, 1479.7042, 1544.7511, 1545.7291, 1545.7291, 1577.7306, 1594.7506, 1616.7400, 1705.8174, 1722.8423, 1856.9188, 1859.8979, 1860.8865, 1860.8865, 1874.9255, 1906.9465, 1923.9232, 1936.8352, 1938.8387, 1961.8973, 1964.9320, 2003.0313, 2044.9817, 2094.9551, 2112.9187, 2112.9187, 2126.9421, 2169.0920, 2217.9517, 2217.9517, 2234.9858, 2234.9858, 2252.1133, 2518.3064, 2518.3064, 2787.4771, 2898.4109, 2975.4124 | | --- |   **Signal transduction**   | **G3.** | [Cs3g15060.3](http://localhost/mascot/cgi/protein_view.pl?file=../data/20140815/F004021.dat&hit=1)    **Mass:** 41139    **Score:** 122    **Expect:** 2.8e-008  **Matches:** 11 | | --- | --- |  | **Observed** | **Mr(expt)** | **Mr(calc)** | **ppm** | **Start** |  | **End** | **Miss** | **Ions** | **Peptide** | | --- | --- | --- | --- | --- | --- | --- | --- | --- | --- | | 1068.4869 | 1067.4796 | 1067.4883 | -8.17 | 69 | - | 77 | 0 | --- | K.GIQTSEDYR.F | | 1068.4869 | 1067.4796 | 1067.4883 | -8.17 | 69 | - | 77 | 0 | 30 | K.GIQTSEDYR.F | | 1068.5795 | 1067.5722 | 1067.6015 | -27.47 | 94 | - | 102 | 0 | --- | K.TLVFQFSVK.H | | 1311.6835 | 1310.6762 | 1310.7235 | -36.03 | 92 | - | 102 | 1 | --- | K.DKTLVFQFSVK.H | | 1465.7273 | 1464.7200 | 1464.6490 | 48.5 | 103 | - | 115 | 1 | --- | K.HEQKLDCGGGYMK.L | | 1465.7273 | 1464.7200 | 1464.6490 | 48.5 | 103 | - | 115 | 1 | --- | K.HEQKLDCGGGYMK.L | | 1590.8428 | 1589.8355 | 1589.8566 | -13.25 | 94 | - | 106 | 1 | --- | K.TLVFQFSVKHEQK.L | | 1649.7557 | 1648.7484 | 1648.7773 | -17.51 | 78 | - | 91 | 0 | --- | R.FYAISAEFPEFSNK.D | | 1649.7557 | 1648.7484 | 1648.7773 | -17.51 | 78 | - | 91 | 0 | 99 | R.FYAISAEFPEFSNK.D | | 1892.8730 | 1891.8657 | 1891.8992 | -17.69 | 78 | - | 93 | 1 | --- | R.FYAISAEFPEFSNKDK.T |  | **No match to:** 713.4006, 716.3989, 721.3210, 724.4883, 728.5417, 734.4730, 750.5033, 758.5023, 768.5092, 772.5541, 792.5551, 802.5527, 816.5747, 825.0639, 834.4774, 841.0364, 842.4830, 848.4930, 850.5173, 854.0284, 855.0219, 857.0117, 860.6100, 860.6100, 861.0355, 868.5276, 870.0050, 871.0020, 877.0168, 877.0168, 882.5446, 886.9687, 886.9687, 892.9759, 902.9384, 904.6229, 904.6229, 914.3827, 948.6627, 963.4435, 963.4435, 977.4529, 977.4529, 982.5441, 989.4625, 992.6758, 1017.3854, 1021.4905, 1036.6909, 1051.6827, 1066.0342, 1082.0082, 1097.9838, 1151.6981, 1165.7123, 1170.6084, 1171.5383, 1171.5383, 1187.6432, 1187.6432, 1209.6254, 1238.6467, 1238.6467, 1261.6798, 1261.6798, 1398.6002, 1410.6008, 1414.7097, 1414.7097, 1932.0181, 1932.0181 | | --- |  | **G27.** | [Cs9g03630.1](http://localhost/mascot/cgi/protein_view.pl?file=../data/20140815/F004019.dat&hit=1)    **Mass:** 17632    **Score:** 253    **Expect:** 2.2e-021  **Matches:** 19 | | --- | --- |  | **Observed** | **Mr(expt)** | **Mr(calc)** | **ppm** | **Start** |  | **End** | **Miss** | **Ions** | **Peptide** | | --- | --- | --- | --- | --- | --- | --- | --- | --- | --- | | 750.4648 | 749.4575 | 749.3973 | 80.4 | 67 | - | 71 | 1 | --- | K.YFKHR.V | | 782.4901 | 781.4828 | 781.5062 | -29.87 | 34 | - | 40 | 0 | --- | K.VLPQVVK.N | | 916.4550 | 915.4477 | 915.4662 | -20.21 | 107 | - | 116 | 0 | --- | K.VVGTPDGGSK.S | | 953.4946 | 952.4874 | 952.5090 | -22.74 | 70 | - | 77 | 1 | --- | K.HRVDALDK.E | | 1031.5145 | 1030.5072 | 1030.5295 | -21.58 | 72 | - | 80 | 1 | --- | R.VDALDKENK.I | | 1149.5902 | 1148.5829 | 1148.6230 | -34.87 | 123 | - | 132 | 0 | --- | K.FYPKPGAEIK.E | | 1149.5902 | 1148.5829 | 1148.6230 | -34.87 | 123 | - | 132 | 0 | 32 | K.FYPKPGAEIK.E | | 1212.5295 | 1211.5222 | 1211.5611 | -32.11 | 57 | - | 66 | 0 | --- | K.FNFVEGADWK.Y | | 1340.6244 | 1339.6171 | 1339.6561 | -29.08 | 56 | - | 66 | 1 | --- | K.KFNFVEGADWK.Y | | 1442.7803 | 1441.7730 | 1441.7413 | 22.0 | 41 | - | 55 | 0 | --- | K.NVELISGDGGPGSIK.K | | 1442.7803 | 1441.7730 | 1441.7413 | 22.0 | 41 | - | 55 | 0 | 22 | K.NVELISGDGGPGSIK.K | | 1570.7974 | 1569.7901 | 1569.8362 | -29.37 | 41 | - | 56 | 1 | --- | K.NVELISGDGGPGSIKK.F | | 1761.8777 | 1760.8704 | 1760.9461 | -42.96 | 145 | - | 161 | 1 | --- | K.GIFKALEAYALANPNAV.- | | 1762.8755 | 1761.8682 | 1761.9301 | -35.12 | 123 | - | 137 | 1 | --- | K.FYPKPGAEIKEEQVK.G | | 1762.8755 | 1761.8682 | 1761.9301 | -35.12 | 123 | - | 137 | 1 | 104 | K.FYPKPGAEIKEEQVK.G | | 1812.9152 | 1811.9079 | 1811.9517 | -24.14 | 2 | - | 18 | 0 | --- | M.GVLTLNVEDTSTLPPEK.L |  | **No match to:** 712.2415, 713.4059, 724.4879, 768.5145, 822.4586, 832.2825, 832.2825, 842.4818, 850.5183, 868.5194, 882.5364, 1021.5126, 1051.6753, 1147.5261, 1151.5621, 1155.5184, 1163.5609, 1167.5129, 1171.5729, 1179.5437, 1183.5189, 1195.5249, 1210.5236, 1216.5259, 1216.5259, 1227.5942, 1228.5354, 1228.5354, 1232.5404, 1234.5247, 1238.5118, 1244.5253, 1250.4984, 1260.5210, 1266.5042, 1277.6570, 1289.6165, 1292.6278, 1307.6118, 1320.6022, 1323.6309, 1324.6556, 1324.6556, 1329.6832, 1338.6245, 1344.6193, 1344.6193, 1352.6392, 1354.6050, 1356.6173, 1360.6235, 1362.6176, 1372.6190, 1388.6246, 1392.5878, 1392.5878, 1406.6302, 1407.6719, 1424.7423, 1441.7734, 1456.7477, 1464.7604, 1475.7218, 1477.7637, 1480.7369, 1520.6885, 1533.8302, 1535.7494, 1546.7686, 1546.7686, 1562.8011, 1563.7719, 1564.7672, 1564.7672, 1586.7632, 1592.7816, 1692.8705, 1744.8793, 1784.8584, 1798.8951, 1798.8951, 1820.8658, 1901.9266, 1916.9910, 1919.9205, 1919.9205, 1933.9424, 1938.9030, 2246.1594, 2559.2056, 2895.3223, 2911.3914, 2912.3643, 2913.3147, 2913.3147, 2927.3259, 2935.2603, 2935.2603, 2951.2476 | | --- |  | **G5 .** | [Cs3g18200.2](http://localhost/mascot/cgi/protein_view.pl?file=../data/20140815/F003968.dat&hit=1)    **Mass:** 28810    **Score:** 670    **Expect:** 4.4e-063  **Matches:** 29 | | --- | --- |  | **Observed** | **Mr(expt)** | **Mr(calc)** | **ppm** | **Start** |  | **End** | **Miss** | **Ions** | **Peptide** | | --- | --- | --- | --- | --- | --- | --- | --- | --- | --- | | 708.3721 | 707.3649 | 707.3966 | -44.86 | 83 | - | 87 | 1 | --- | R.IKEYR.Q | | 750.3221 | 749.3149 | 749.3378 | -30.55 | 1 | - | 5 | 1 | --- | -.MEKER.E + Acetyl (Protein N-term); Oxidation (M) | | 770.3757 | 769.3685 | 769.4010 | -42.32 | 132 | - | 137 | 0 | --- | R.YLAEFK.G | | 816.3887 | 815.3815 | 815.4137 | -39.54 | 14 | - | 20 | 0 | --- | R.LAEQAER.Y | | 870.4055 | 869.3982 | 869.4243 | -29.94 | 144 | - | 151 | 0 | --- | K.EAADHSLK.A | | 879.4555 | 878.4482 | 878.4862 | -43.17 | 44 | - | 51 | 0 | --- | R.NLVSVGYK.N | | 932.3991 | 931.3918 | 931.4222 | -32.56 | 125 | - | 131 | 1 | --- | K.MKGDYYR.Y | | 944.4595 | 943.4522 | 943.4835 | -33.20 | 75 | - | 82 | 1 | --- | K.GNEQNVKR.I | | 948.3879 | 947.3806 | 947.4171 | -38.50 | 125 | - | 131 | 1 | --- | K.MKGDYYR.Y + Oxidation (M) | | 948.3879 | 947.3806 | 947.4171 | -38.50 | 125 | - | 131 | 1 | 20 | K.MKGDYYR.Y + Oxidation (M) | | 1024.4893 | 1023.4820 | 1023.5250 | -41.98 | 6 | - | 13 | 0 | --- | R.EHHVYLAR.L | | 1024.4893 | 1023.4820 | 1023.5250 | -41.98 | 6 | - | 13 | 0 | 59 | R.EHHVYLAR.L | | 1087.5321 | 1086.5248 | 1086.5669 | -38.73 | 88 | - | 96 | 1 | --- | R.QRVEDELAK.I | | 1202.5804 | 1201.5731 | 1201.6190 | -38.19 | 34 | - | 43 | 0 | --- | K.LDVELTVEER.N | | 1202.5804 | 1201.5731 | 1201.6190 | -38.19 | 34 | - | 43 | 0 | 75 | K.LDVELTVEER.N | | 1205.6075 | 1204.6002 | 1204.6485 | -40.11 | 217 | - | 226 | 0 | --- | K.DSTLIMQLLR.D + Oxidation (M) | | 1284.5729 | 1283.5656 | 1283.6146 | -38.15 | 132 | - | 142 | 1 | --- | R.YLAEFKGADDR.K | | 1284.5729 | 1283.5656 | 1283.6146 | -38.15 | 132 | - | 142 | 1 | 51 | R.YLAEFKGADDR.K | | 1309.6238 | 1308.6165 | 1308.6687 | -39.86 | 4 | - | 13 | 1 | --- | K.EREHHVYLAR.L | | 1309.6238 | 1308.6165 | 1308.6687 | -39.86 | 4 | - | 13 | 1 | 52 | K.EREHHVYLAR.L | | 1374.6962 | 1373.6889 | 1373.7402 | -37.30 | 63 | - | 74 | 1 | --- | R.ILSSIEQKEEAK.G | | 1500.7665 | 1499.7592 | 1499.8195 | -40.19 | 31 | - | 43 | 1 | --- | K.VAKLDVELTVEER.N | | 1500.7665 | 1499.7592 | 1499.8195 | -40.19 | 31 | - | 43 | 1 | 121 | K.VAKLDVELTVEER.N | | 1928.8406 | 1927.8333 | 1927.8655 | -16.69 | 14 | - | 29 | 1 | --- | R.LAEQAERYDEMVEAMK.K + Oxidation (M) | | 1944.8044 | 1943.7971 | 1943.8604 | -32.57 | 14 | - | 29 | 1 | --- | R.LAEQAERYDEMVEAMK.K + 2 Oxidation (M) | | 2036.9531 | 2035.9458 | 2036.0327 | -42.65 | 152 | - | 171 | 0 | --- | K.AYEAATAAAASELPPTHPIR.L | | 2036.9531 | 2035.9458 | 2036.0327 | -42.65 | 152 | - | 171 | 0 | 174 | K.AYEAATAAAASELPPTHPIR.L | | 2345.1248 | 2344.1175 | 2344.2103 | -39.58 | 172 | - | 191 | 0 | --- | R.LGLALNFSVFYYEILNSPER.A | | 2888.3350 | 2887.3277 | 2887.4464 | -41.09 | 144 | - | 171 | 1 | --- | K.EAADHSLKAYEAATAAAASELPPTHPIR.L |  | **No match to:** 700.3325, 703.3206, 707.3344, 713.3686, 729.3558, 730.3583, 747.3627, 758.4007, 768.5068, 819.4313, 830.3973, 832.2811, 835.3851, 836.3972, 837.3996, 842.4726, 845.3803, 848.3989, 850.4810, 855.3945, 868.5172, 882.5200, 884.4218, 901.4278, 956.4113, 964.4998, 1006.4833, 1051.6492, 1070.5057, 1070.5057, 1092.4949, 1130.5427, 1140.5691, 1155.5604, 1177.5386, 1216.6018, 1248.6193, 1252.6107, 1279.6422, 1291.6199, 1291.6199, 1325.6302, 1331.6073, 1384.7662, 1394.5930, 1412.6608, 1412.6608, 1456.6941, 1512.6930, 1514.7705, 1531.7776, 1602.7924, 1628.8501, 1635.8501, 1673.8298, 1691.8063, 1734.7960, 1755.7974, 1755.7974, 1802.8651, 1942.9387, 1992.9030, 2007.9486, 2008.8739, 2008.8739, 2018.9259, 2035.9482, 2039.4152, 2050.9714, 2054.9607, 2056.9028, 2058.9175, 2072.8936, 2072.8936, 2094.9419, 2124.9802, 2144.0330, 2264.0496, 2278.0532, 2295.0925, 2300.1262, 2300.1262, 2361.2029, 2361.2029, 3187.4600, 3341.5417 | | --- |  | **G22.** | [Cs3g18360.1](http://localhost/mascot/cgi/protein_view.pl?file=../data/20140815/F003994.dat&hit=1)    **Mass:** 36075    **Score:** 784    **Expect:** 1.8e-074  **Matches:** 31 | | --- | --- |  | **Observed** | **Mr(expt)** | **Mr(calc)** | **ppm** | **Start** |  | **End** | **Miss** | **Ions** | **Peptide** | | --- | --- | --- | --- | --- | --- | --- | --- | --- | --- | | 745.3563 | 744.3490 | 744.3667 | -23.80 | 123 | - | 128 | 0 | --- | R.QAYHAR.F | | 800.4017 | 799.3945 | 799.4188 | -30.46 | 287 | - | 293 | 0 | --- | R.NSVPLDR.A | | 956.5063 | 955.4990 | 955.5199 | -21.87 | 286 | - | 293 | 1 | --- | R.RNSVPLDR.A | | 1023.4485 | 1022.4412 | 1022.4669 | -25.09 | 68 | - | 75 | 0 | --- | K.ELTNDFER.V | | 1036.4995 | 1035.4922 | 1035.5237 | -30.35 | 226 | - | 233 | 0 | --- | K.DEYLDLLR.A | | 1047.4785 | 1046.4712 | 1046.4993 | -26.78 | 257 | - | 266 | 0 | --- | K.QGTDEGALTR.V | | 1108.4865 | 1107.4792 | 1107.5196 | -36.48 | 183 | - | 191 | 0 | --- | K.AYNDEDLIR.I | | 1108.4865 | 1107.4792 | 1107.5196 | -36.48 | 183 | - | 191 | 0 | 87 | K.AYNDEDLIR.I | | 1158.6827 | 1157.6754 | 1157.7172 | -36.09 | 148 | - | 157 | 0 | --- | K.LLLPLVTAYR.Y | | 1158.6827 | 1157.6754 | 1157.7172 | -36.09 | 148 | - | 157 | 0 | 74 | K.LLLPLVTAYR.Y | | 1235.6007 | 1234.5934 | 1234.6306 | -30.09 | 88 | - | 98 | 1 | --- | R.DAFLANEATKR.W | | 1263.6313 | 1262.6240 | 1262.6619 | -29.97 | 199 | - | 209 | 0 | --- | K.AQINATLNQYK.N | | 1286.7776 | 1285.7703 | 1285.8122 | -32.54 | 147 | - | 157 | 1 | --- | R.KLLLPLVTAYR.Y | | 1445.7021 | 1444.6948 | 1444.7384 | -30.19 | 76 | - | 87 | 0 | --- | R.VVMLWTLDPAER.D + Oxidation (M) | | 1466.6600 | 1465.6527 | 1465.7049 | -35.57 | 64 | - | 75 | 1 | --- | K.SLDKELTNDFER.V | | 1466.6600 | 1465.6527 | 1465.7049 | -35.57 | 64 | - | 75 | 1 | 98 | K.SLDKELTNDFER.V | | 1478.7423 | 1477.7350 | 1477.7888 | -36.42 | 197 | - | 209 | 1 | --- | R.SKAQINATLNQYK.N | | 1516.6769 | 1515.6696 | 1515.7093 | -26.17 | 51 | - | 63 | 0 | --- | R.QTYADTYGEDLLK.S | | 1550.7319 | 1549.7246 | 1549.7736 | -31.60 | 179 | - | 191 | 1 | --- | K.ISNKAYNDEDLIR.I | | 1738.7059 | 1737.6986 | 1737.7594 | -35.00 | 132 | - | 146 | 0 | --- | K.SLEEDVGYHTNGDFR.K | | 1738.7059 | 1737.6986 | 1737.7594 | -35.00 | 132 | - | 146 | 0 | 87 | K.SLEEDVGYHTNGDFR.K | | 1819.8346 | 1818.8273 | 1818.9087 | -44.73 | 234 | - | 248 | 0 | --- | R.ATVQCLVRPEHYFEK.I | | 1866.7949 | 1865.7876 | 1865.8544 | -35.79 | 132 | - | 147 | 1 | --- | K.SLEEDVGYHTNGDFRK.L | | 1866.7949 | 1865.7876 | 1865.8544 | -35.79 | 132 | - | 147 | 1 | 35 | K.SLEEDVGYHTNGDFRK.L | | 1898.8507 | 1897.8434 | 1897.9785 | -71.18 | 48 | - | 63 | 1 | --- | K.LIRQTYADTYGEDLLK.S | | 2021.9219 | 2020.9146 | 2020.9412 | -13.13 | 298 | - | 316 | 1 | --- | K.DTSGDYEKMLLALLGHGDA.- + Oxidation (M) | | 2155.0508 | 2154.0435 | 2154.1222 | -36.51 | 23 | - | 41 | 0 | --- | K.AFEGWGTNEQLIISILAHR.N | | 2491.2869 | 2490.2796 | 2490.3733 | -37.63 | 148 | - | 169 | 1 | --- | K.LLLPLVTAYRYEGDEVNITLAK.S | | 2491.2869 | 2490.2796 | 2490.3733 | -37.63 | 148 | - | 169 | 1 | 108 | K.LLLPLVTAYRYEGDEVNITLAK.S | | 2823.2498 | 2822.2425 | 2822.3246 | -29.09 | 210 | - | 233 | 1 | --- | K.NVYGNDIDQDLEADPKDEYLDLLR.A | | 2823.2498 | 2822.2425 | 2822.3246 | -29.09 | 210 | - | 233 | 1 | 138 | K.NVYGNDIDQDLEADPKDEYLDLLR.A |  | **No match to:** 712.3746, 728.3244, 768.4772, 832.2813, 842.4813, 968.5102, 1030.4434, 1030.4434, 1064.5345, 1064.5345, 1121.5551, 1128.5134, 1220.6416, 1261.6366, 1261.6366, 1374.6833, 1449.6967, 1461.7004, 1465.7067, 1477.7079, 1480.7406, 1499.6456, 1521.6592, 1544.8267, 1562.6541, 1564.8271, 1688.8165, 1719.8615, 1721.7181, 1747.7899, 1749.7773, 1752.7631, 1763.7738, 1784.7902, 1794.7872, 1795.7742, 1811.7865, 1811.7865, 1815.7837, 1827.7830, 1843.7893, 1848.7882, 1849.7822, 1858.8506, 1859.8386, 1874.8774, 1875.8846, 1876.8625, 1876.8625, 1888.7981, 1890.8571, 1892.8842, 1908.8469, 1933.8900, 1994.8849, 2020.9384, 2141.0479, 2158.0532, 2159.0427, 2159.0427, 2169.0332, 2171.0469, 2181.0430, 2187.0386, 2272.0332, 2272.0332, 2287.1245, 2308.0737, 2308.5581, 2309.1194, 2312.1279, 2358.1306, 2368.1978, 2382.1458, 2398.1475, 2400.1233, 2400.1233, 2414.1318, 2473.2781, 2602.2615, 2619.3826, 2662.2969, 2666.2871, 2678.2849 | | --- |   **Cellular transport**   | **G28.** | [Cs7g30630.1](http://localhost/mascot/cgi/protein_view.pl?file=../data/20140815/F004002.dat&hit=1)    **Mass:** 29447    **Score:** 499    **Expect:** 5.6e-046  **Matches:** 27 | | --- | --- |  | **Observed** | **Mr(expt)** | **Mr(calc)** | **ppm** | **Start** |  | **End** | **Miss** | **Ions** | **Peptide** | | --- | --- | --- | --- | --- | --- | --- | --- | --- | --- | | 737.4570 | 736.4498 | 736.4595 | -13.28 | 197 | - | 202 | 0 | --- | K.LLNLHK.V | | 865.3365 | 864.3292 | 864.3461 | -19.50 | 136 | - | 142 | 0 | --- | K.ESSEEER.E | | 941.4217 | 940.4145 | 940.4324 | -19.05 | 148 | - | 154 | 0 | --- | K.LMEYQNK.R + Oxidation (M) | | 1078.5524 | 1077.5451 | 1077.5818 | -34.07 | 231 | - | 239 | 0 | --- | K.ISEYVAQLR.R | | 1078.5524 | 1077.5451 | 1077.5818 | -34.07 | 231 | - | 239 | 0 | 78 | K.ISEYVAQLR.R | | 1097.5148 | 1096.5075 | 1096.5335 | -23.68 | 148 | - | 155 | 1 | --- | K.LMEYQNKR.G + Oxidation (M) | | 1097.5148 | 1096.5075 | 1096.5335 | -23.68 | 148 | - | 155 | 1 | 16 | K.LMEYQNKR.G + Oxidation (M) | | 1206.6539 | 1205.6466 | 1205.6768 | -25.02 | 230 | - | 239 | 1 | --- | K.KISEYVAQLR.R | | 1206.6539 | 1205.6466 | 1205.6768 | -25.02 | 230 | - | 239 | 1 | 89 | K.KISEYVAQLR.R | | 1234.6581 | 1233.6508 | 1233.6829 | -26.03 | 231 | - | 240 | 1 | --- | K.ISEYVAQLRR.V | | 1234.6581 | 1233.6508 | 1233.6829 | -26.03 | 231 | - | 240 | 1 | 7 | K.ISEYVAQLRR.V | | 1287.7217 | 1286.7144 | 1286.5779 | 106 | 133 | - | 142 | 1 | --- | K.FFKESSEEER.E | | 1459.6798 | 1458.6725 | 1458.6222 | 34.5 | 136 | - | 147 | 1 | --- | K.ESSEEEREHAEK.L | | 1650.8405 | 1649.8332 | 1649.9352 | -61.81 | 74 | - | 88 | 0 | --- | K.ELDLVPTVPQLSLAR.H | | 1669.7998 | 1668.7925 | 1668.8280 | -21.26 | 177 | - | 191 | 0 | --- | K.GDALYAMELTLSLEK.L + Oxidation (M) | | 1873.8899 | 1872.8826 | 1872.9291 | -24.83 | 161 | - | 176 | 0 | --- | K.LQSILMPLSEFDHAEK.G + Oxidation (M) | | 1873.8899 | 1872.8826 | 1872.9291 | -24.83 | 161 | - | 176 | 0 | 83 | K.LQSILMPLSEFDHAEK.G + Oxidation (M) | | 2046.9272 | 2045.9199 | 2046.0422 | -59.76 | 54 | - | 72 | 0 | --- | K.NANNSPLTGVIFAPFEEVK.K | | 2175.0884 | 2174.0811 | 2174.1371 | -25.77 | 54 | - | 73 | 1 | --- | K.NANNSPLTGVIFAPFEEVKK.E | | 2175.0884 | 2174.0811 | 2174.1371 | -25.77 | 54 | - | 73 | 1 | 155 | K.NANNSPLTGVIFAPFEEVKK.E |  | **No match to:** 713.4042, 768.5182, 782.4457, 818.3613, 818.3613, 822.3750, 823.3781, 832.2933, 842.4888, 868.5286, 883.4177, 939.4167, 960.5292, 970.5005, 971.4765, 972.4445, 990.4665, 1006.5704, 1020.4390, 1021.4819, 1032.5311, 1033.5173, 1036.4305, 1036.4305, 1040.4569, 1052.4586, 1068.4612, 1074.5062, 1084.6150, 1092.5691, 1100.5355, 1104.5762, 1173.4945, 1195.6189, 1213.5710, 1229.6230, 1231.6515, 1241.5548, 1251.4983, 1264.6110, 1348.6267, 1350.6177, 1357.7109, 1357.7109, 1374.6687, 1379.6981, 1392.6813, 1410.6256, 1456.7445, 1465.7169, 1478.6808, 1500.6693, 1567.8221, 1605.7972, 1610.8306, 1611.8635, 1611.8635, 1616.8307, 1617.8247, 1629.8086, 1632.8346, 1634.8221, 1634.8221, 1652.8654, 1654.8146, 1659.8553, 1660.8597, 1680.8552, 1682.8629, 1683.8594, 1695.8342, 1697.8335, 1698.8560, 1698.8560, 1714.8533, 1720.8463, 1809.8823, 1823.9298, 1826.9474, 1839.9015, 1848.8655, 1849.8939, 1849.8939, 1903.9078, 1920.9370, 1921.9771, 1921.9771, 2056.9636, 2056.9636, 2197.0454, 2401.1033, 2437.0964, 2465.1191, 2477.1199, 2750.2693 | | --- |   **Cell wall and cytoskeleton**   | **G7.** | [Cs3g26180.1](http://localhost/mascot/cgi/protein_view.pl?file=../data/20140815/F003972.dat&hit=1)    **Mass:** 50343    **Score:** 775    **Expect:** 1.4e-073  **Matches:** 30 | | --- | --- |  | **Observed** | **Mr(expt)** | **Mr(calc)** | **ppm** | **Start** |  | **End** | **Miss** | **Ions** | **Peptide** | | --- | --- | --- | --- | --- | --- | --- | --- | --- | --- | | 1059.5001 | 1058.4928 | 1058.5219 | -27.45 | 312 | - | 320 | 0 | --- | R.YLTASAMFR.G | | 1075.4941 | 1074.4868 | 1074.5168 | -27.90 | 312 | - | 320 | 0 | --- | R.YLTASAMFR.G + Oxidation (M) | | 1075.4941 | 1074.4868 | 1074.5168 | -27.90 | 312 | - | 320 | 0 | 20 | R.YLTASAMFR.G + Oxidation (M) | | 1077.5013 | 1076.4940 | 1076.5250 | -28.81 | 157 | - | 164 | 1 | --- | K.IREEYPDR.M | | 1139.6526 | 1138.6453 | 1138.6862 | -35.94 | 255 | - | 264 | 0 | --- | K.LAVNLIPFPR.L | | 1139.6526 | 1138.6453 | 1138.6862 | -35.94 | 255 | - | 264 | 0 | 66 | K.LAVNLIPFPR.L | | 1146.5542 | 1145.5469 | 1145.5829 | -31.42 | 244 | - | 253 | 0 | --- | R.FPGQLNSDLR.K | | 1146.5542 | 1145.5469 | 1145.5829 | -31.42 | 244 | - | 253 | 0 | 76 | R.FPGQLNSDLR.K | | 1215.5537 | 1214.5464 | 1214.5754 | -23.85 | 383 | - | 392 | 0 | --- | R.VSEQFTAMFR.R | | 1230.5645 | 1229.5572 | 1229.5888 | -25.67 | 38 | - | 48 | 0 | --- | R.GDGVEDLQLER.I | | 1231.5405 | 1230.5332 | 1230.5703 | -30.14 | 383 | - | 392 | 0 | --- | R.VSEQFTAMFR.R + Oxidation (M) | | 1231.5405 | 1230.5332 | 1230.5703 | -30.14 | 383 | - | 392 | 0 | 27 | R.VSEQFTAMFR.R + Oxidation (M) | | 1267.7523 | 1266.7450 | 1266.7812 | -28.56 | 254 | - | 264 | 1 | --- | R.KLAVNLIPFPR.L | | 1274.6407 | 1273.6334 | 1273.6779 | -34.90 | 244 | - | 254 | 1 | --- | R.FPGQLNSDLRK.L | | 1274.6407 | 1273.6334 | 1273.6779 | -34.90 | 244 | - | 254 | 1 | 81 | R.FPGQLNSDLRK.L | | 1342.5957 | 1341.5884 | 1341.6313 | -31.96 | 49 | - | 60 | 0 | --- | R.INVYYNEASGGR.Y | | 1342.5957 | 1341.5884 | 1341.6313 | -31.96 | 49 | - | 60 | 0 | 94 | R.INVYYNEASGGR.Y | | 1387.6407 | 1386.6334 | 1386.6714 | -27.40 | 383 | - | 393 | 1 | --- | R.VSEQFTAMFRR.K + Oxidation (M) | | 1387.6407 | 1386.6334 | 1386.6714 | -27.40 | 382 | - | 392 | 1 | 19 | R.RVSEQFTAMFR.R + Oxidation (M) | | 1549.7150 | 1548.7077 | 1548.7532 | -29.38 | 36 | - | 48 | 1 | --- | K.YRGDGVEDLQLER.I | | 1549.7150 | 1548.7077 | 1548.7532 | -29.38 | 36 | - | 48 | 1 | 70 | K.YRGDGVEDLQLER.I | | 1638.7958 | 1637.7885 | 1637.8388 | -30.72 | 265 | - | 278 | 0 | --- | R.LHFFMVGFAPLTSR.G + Oxidation (M) | | 1638.7958 | 1637.7885 | 1637.8388 | -30.72 | 265 | - | 278 | 0 | 63 | R.LHFFMVGFAPLTSR.G + Oxidation (M) | | 1679.7548 | 1678.7475 | 1678.7906 | -25.66 | 65 | - | 79 | 0 | --- | R.AVLMDLEPGTMDSIR.S + 2 Oxidation (M) | | 1893.7654 | 1892.7581 | 1892.8972 | -73.46 | 323 | - | 338 | 1 | --- | K.MSTKEVDEQMINVQNK.N | | 1951.8419 | 1950.8346 | 1950.8815 | -24.05 | 365 | - | 381 | 0 | --- | K.MASTFIGNSTSIQEMFR.R + 2 Oxidation (M) | | 1956.9380 | 1955.9307 | 1955.9953 | -32.99 | 106 | - | 123 | 0 | --- | K.GHYTEGAELIDAVLDVVR.K | | 1956.9380 | 1955.9307 | 1955.9953 | -32.99 | 106 | - | 123 | 0 | 162 | K.GHYTEGAELIDAVLDVVR.K | | 2085.0298 | 2084.0225 | 2084.0255 | -1.43 | 1 | - | 19 | 1 | --- | -.MREILHIQGGQCGNQIGSK.F + Oxidation (M) | | 2107.9519 | 2106.9446 | 2106.9827 | -18.05 | 365 | - | 382 | 1 | --- | K.MASTFIGNSTSIQEMFRR.V + 2 Oxidation (M) |  | **No match to:** 738.3505, 768.5178, 842.4848, 948.5047, 1000.5948, 1011.5052, 1026.5831, 1030.4728, 1086.4989, 1097.4036, 1116.5363, 1138.6350, 1161.6362, 1166.5823, 1167.5461, 1168.5684, 1169.5319, 1169.5319, 1183.5623, 1207.5386, 1212.5695, 1235.5459, 1256.5924, 1257.5957, 1270.6803, 1276.6041, 1294.6321, 1296.6317, 1324.6254, 1336.5806, 1364.5973, 1370.5988, 1381.6934, 1398.6381, 1408.6232, 1414.6208, 1431.5951, 1443.6102, 1468.6323, 1469.5889, 1486.6119, 1486.6119, 1500.6420, 1531.6879, 1532.6899, 1574.3557, 1574.7928, 1592.7800, 1598.7417, 1615.7676, 1637.7789, 1661.7678, 1665.7526, 1733.7777, 1788.8058, 1876.8617, 1887.7983, 1904.8014, 1905.7577, 1972.9438, 1993.7634, 2009.8083, 2019.7538, 2030.8643, 2043.8987, 2077.9670, 2101.0347, 2142.0071, 2250.9553, 2250.9553, 2264.9756, 2266.9556, 2268.9373, 2268.9373, 2282.9441, 2282.9441, 2325.9529, 2634.0322, 2695.0771, 2753.2366, 2818.2488, 2830.2119, 2859.1721, 2920.2158, 3312.3984 | | --- |  | **G2.** | [Cs8g01850.1](http://localhost/mascot/cgi/protein_view.pl?file=../data/20140815/F003962.dat&hit=1)    **Mass:** 35362    **Score:** 75     **Expect:** 0.0013  **Matches:** 9 | | --- | --- |  | **Observed** | **Mr(expt)** | **Mr(calc)** | **ppm** | **Start** |  | **End** | **Miss** | **Ions** | **Peptide** | | --- | --- | --- | --- | --- | --- | --- | --- | --- | --- | | 743.3947 | 742.3874 | 742.3762 | 15.1 | 183 | - | 188 | 1 | --- | R.GKYYGR.G | | 1215.5404 | 1214.5331 | 1214.5641 | -25.53 | 87 | - | 95 | 0 | --- | K.MFEDLLEYR.N | | 1231.5277 | 1230.5204 | 1230.5591 | -31.39 | 87 | - | 95 | 0 | --- | K.MFEDLLEYR.N + Oxidation (M) | | 1231.5277 | 1230.5204 | 1230.5591 | -31.39 | 87 | - | 95 | 0 | 33 | K.MFEDLLEYR.N + Oxidation (M) | | 1343.6127 | 1342.6054 | 1342.6591 | -39.97 | 86 | - | 95 | 1 | --- | R.KMFEDLLEYR.N | | 1359.6161 | 1358.6088 | 1358.6540 | -33.26 | 86 | - | 95 | 1 | --- | R.KMFEDLLEYR.N + Oxidation (M) | | 1359.6161 | 1358.6088 | 1358.6540 | -33.26 | 86 | - | 95 | 1 | 33 | R.KMFEDLLEYR.N + Oxidation (M) | | 1626.7533 | 1625.7460 | 1625.7620 | -9.83 | 117 | - | 131 | 1 | --- | K.AFPAFGNSGNETMRK.R |  | **No match to:** 711.3567, 713.3825, 728.3644, 767.4167, 768.5094, 782.3738, 806.3401, 816.3290, 832.2852, 842.4782, 864.4342, 868.5283, 882.5351, 1013.4211, 1028.4617, 1032.4613, 1038.4459, 1040.4618, 1044.4573, 1060.4575, 1079.4380, 1083.4675, 1084.4823, 1115.4904, 1119.4890, 1131.4904, 1147.4873, 1166.5176, 1177.5332, 1181.5652, 1184.5209, 1237.6350, 1244.4561, 1245.5164, 1280.4811, 1280.4811, 1294.6276, 1294.6276, 1296.6261, 1344.6121, 1345.5978, 1373.6282, 1442.5680, 1447.6538, 1459.6542, 1481.5481, 1533.5223, 1533.5223, 1552.7190, 1581.6478, 1590.7449, 1594.7701, 1597.6788, 1598.6669, 1609.7571, 1619.7372, 1620.7385, 1623.7675, 1627.7615, 1627.7615, 1636.7677, 1637.7463, 1638.7542, 1639.7618, 1639.7618, 1643.7632, 1655.7584, 1667.7753, 1678.7561, 1679.7717, 1681.7729, 1685.7773, 1685.7773, 1707.7804, 1723.7859, 1769.7343, 1799.8190, 1812.6311, 1866.7878, 1867.8762, 1869.7402, 1869.7402, 1942.7085, 1959.6931, 1960.7091, 1960.7091, 1976.7336, 1982.7032, 1995.8312, 2029.8478, 2085.8508, 2086.8494, 2086.8494, 2296.9651, 2313.9680, 2313.9680, 2511.2341, 2512.8906, 2538.9700, 2585.1760, 2602.2063, 2602.2063, 2621.0903, 2621.0903, 2621.9670, 2685.0205, 2685.0205 | | --- |   **Stress response**   | **G14.** | [**Cs3g21500.1**](http://localhost/mascot/cgi/protein_view.pl?file=../data/20140815/F004024.dat&hit=1)**Mass:**20038 **Score:**323**Expect:**2.2e-028**Matches:** 8 | | --- | --- |  | **Observed** | **Mr(expt)** | **Mr(calc)** | **ppm** | **Start** |  | **End** | **Miss** | **Ions** | **Peptide** | | --- | --- | --- | --- | --- | --- | --- | --- | --- | --- | | 861.3134 | 860.3061 | 860.3889 | -96.19 | 128 | - | 134 | 0 | --- | K.DPEHAHR.H | | 861.3134 | 860.3061 | 860.3889 | -96.19 | 128 | - | 134 | 0 | 45 | K.DPEHAHR.H | | 1353.4897 | 1352.4824 | 1352.5957 | -83.72 | 75 | - | 86 | 0 | --- | R.RPSETESGDYGR.K | | 1353.4897 | 1352.4824 | 1352.5957 | -83.72 | 75 | - | 86 | 0 | 45 | R.RPSETESGDYGR.K | | 1509.5713 | 1508.5640 | 1508.6968 | -87.98 | 74 | - | 86 | 1 | --- | R.RRPSETESGDYGR.K | | 1509.5713 | 1508.5640 | 1508.6968 | -87.98 | 74 | - | 86 | 1 | 71 | R.RRPSETESGDYGR.K | | 2772.1365 | 2771.1292 | 2771.3415 | -76.61 | 135 | - | 160 | 1 | --- | R.HKIEEEIAAAAAVGSGGFAFHEHHEK.K | | 2772.1365 | 2771.1292 | 2771.3415 | -76.61 | 135 | - | 160 | 1 | 123 | R.HKIEEEIAAAAAVGSGGFAFHEHHEK.K |  | **No match to:** 728.4928, 768.4603, 772.5099, 825.0316, 840.9866, 842.4243, 860.5421, 876.9576, 876.9576, 892.9369, 892.9369, 904.5777, 948.5986, 1031.4272, 1293.5470, 1293.5470, 1379.5900, 1379.5900, 1579.6492, 1579.6492 | | --- |   **Others**   | **G20.** | [**orange1.1t05091.1**](http://localhost/mascot/cgi/protein_view.pl?file=../data/20140815/F004027.dat&hit=1)**Mass:**17526  **Score:**161**Expect:**3.5e-012**Matches:**31 | | --- | --- |  | **Observed** | **Mr(expt)** | **Mr(calc)** | **ppm** | **Start** |  | **End** | **Miss** | **Ions** | **Peptide** | | --- | --- | --- | --- | --- | --- | --- | --- | --- | --- | | 861.3870 | 860.3797 | 860.3777 | 2.43 | 55 | - | 61 | 0 | --- | R.ANEDAWR.G | | 893.3728 | 892.3655 | 892.3895 | -26.85 | 1 | - | 7 | 1 | --- | -.MACDQRK.L + Acetyl (Protein N-term) | | 893.3728 | 892.3655 | 892.3895 | -26.85 | 1 | - | 7 | 1 | --- | -.MACDQRK.L + Acetyl (Protein N-term) | | 942.4482 | 941.4409 | 941.4429 | -2.15 | 32 | - | 39 | 0 | --- | R.MDFGLFGR.S | | 942.4482 | 941.4409 | 941.4429 | -2.15 | 32 | - | 39 | 0 | 10 | R.MDFGLFGR.S | | 947.4519 | 946.4446 | 946.4549 | -10.82 | 13 | - | 19 | 0 | --- | K.SDYFFLR.Y | | 947.4519 | 946.4446 | 946.4549 | -10.82 | 13 | - | 19 | 0 | 4 | K.SDYFFLR.Y | | 958.4408 | 957.4335 | 957.4379 | -4.52 | 32 | - | 39 | 0 | --- | R.MDFGLFGR.S + Oxidation (M) | | 958.4408 | 957.4335 | 957.4379 | -4.52 | 32 | - | 39 | 0 | (10) | R.MDFGLFGR.S + Oxidation (M) | | 962.5582 | 961.5510 | 961.5570 | -6.25 | 95 | - | 102 | 0 | --- | K.NRPIHGLR.T | | 1505.8176 | 1504.8103 | 1504.8078 | 1.66 | 8 | - | 19 | 1 | --- | K.LIGFKSDYFFLR.Y | | 1510.7634 | 1509.7561 | 1509.7497 | 4.25 | 103 | - | 114 | 1 | --- | R.TMVQLDELYKDR.H | | 1510.7634 | 1509.7561 | 1509.7497 | 4.25 | 103 | - | 114 | 1 | 12 | R.TMVQLDELYKDR.H | | 1526.7612 | 1525.7539 | 1525.7446 | 6.09 | 103 | - | 114 | 1 | --- | R.TMVQLDELYKDR.H + Oxidation (M) | | 1526.7612 | 1525.7539 | 1525.7446 | 6.09 | 103 | - | 114 | 1 | (11) | R.TMVQLDELYKDR.H + Oxidation (M) | | 1727.9785 | 1726.9712 | 1726.9559 | 8.87 | 40 | - | 54 | 0 | --- | R.SWAPIVSWAVLFVPR.A | | 3467.9016 | 3466.8943 | 3466.7481 | 42.2 | 115 | - | 146 | 0 | --- | R.HVQEGGSLVLQINLIEVPHGNVLDSDHTFDGK.V |  | **No match to:** 728.5577, 772.5894, 801.4957, 816.6141, 848.5021, 865.3816, 865.3816, 877.3798, 891.3658, 892.2266, 892.3962, 904.6683, 910.4453, 912.4173, 929.4381, 940.4385, 945.4634, 949.7076, 969.4385, 974.4355, 980.4265, 984.5483, 991.5690, 992.7160, 1000.4999, 1018.5032, 1031.4268, 1041.4515, 1081.5133, 1081.5133, 1085.5094, 1103.5060, 1111.5641, 1113.5073, 1115.6838, 1129.5325, 1167.6418, 1171.7513, 1261.5757, 1261.5757, 1458.8542, 1461.8977, 1462.3075, 1462.7614, 1614.9230, 1633.9160, 1633.9160, 1660.9204, 1705.9604, 1717.9767, 1729.9734, 1731.9679, 1735.9565, 1735.9565, 1742.9734, 1743.9725, 1745.9653, 1746.9653, 1747.9611, 1747.9611, 1751.9617, 1755.9583, 1757.9524, 1759.9602, 1763.9517, 1763.9517, 1769.9467, 1771.9463, 1773.9679, 1775.9570, 1775.9570, 1779.9441, 1783.9573, 1785.9558, 1787.9590, 1789.9832, 1791.9578, 1791.9578, 1807.9677, 1839.9856, 1889.0256, 1894.0400, 1898.0040, 1901.9915, 1912.0117, 1914.0396, 1945.1182, 2093.0618, 2253.2263, 3018.4238, 3390.0166, 3390.7087, 3407.7593, 3410.6345, 3412.3616, 3453.6970, 3454.7654, 3470.8013 | | --- |  | **G32.** | [orange1.1t01892.1](http://localhost/mascot/cgi/protein_view.pl?file=../data/20140815/F004006.dat&hit=1)    **Mass:** 53078    **Score:** 577    **Expect:** 1.8e-064  **Matches:** 35 | | --- | --- |  | **Observed** | **Mr(expt)** | **Mr(calc)** | **ppm** | **Start** |  | **End** | **Miss** | **Ions** | **Peptide** | | --- | --- | --- | --- | --- | --- | --- | --- | --- | --- | | 808.4762 | 807.4689 | 807.4967 | -34.35 | 447 | - | 454 | 0 | --- | K.VAALHLGK.L | | 846.4982 | 845.4909 | 845.5375 | -55.07 | 435 | - | 441 | 1 | --- | K.KVYVLPK.H | | 881.3950 | 880.3877 | 880.4178 | -34.17 | 153 | - | 159 | 0 | --- | K.AEEIYEK.T | | 1008.4333 | 1007.4260 | 1007.4672 | -40.89 | 18 | - | 26 | 0 | --- | K.DLSQADFGR.L | | 1008.4333 | 1007.4260 | 1007.4672 | -40.89 | 18 | - | 26 | 0 | 76 | K.DLSQADFGR.L | | 1025.4833 | 1024.4760 | 1024.5124 | -35.50 | 246 | - | 254 | 0 | --- | R.HSLPDGLMR.A | | 1041.4750 | 1040.4677 | 1040.5073 | -38.04 | 246 | - | 254 | 0 | --- | R.HSLPDGLMR.A + Oxidation (M) | | 1071.5752 | 1070.5679 | 1070.6084 | -37.80 | 369 | - | 377 | 0 | --- | R.ITIKPQTDR.W | | 1071.5752 | 1070.5679 | 1070.6084 | -37.80 | 369 | - | 377 | 0 | 56 | R.ITIKPQTDR.W | | 1107.5042 | 1106.4969 | 1106.5397 | -38.62 | 44 | - | 53 | 0 | --- | R.AEFGPSQPFK.G | | 1107.5042 | 1106.4969 | 1106.5397 | -38.62 | 44 | - | 53 | 0 | 63 | R.AEFGPSQPFK.G | | 1133.5005 | 1132.4932 | 1132.5257 | -28.64 | 329 | - | 337 | 0 | --- | K.DIIMVDHMK.K + 2 Oxidation (M) | | 1202.5242 | 1201.5169 | 1201.5550 | -31.67 | 236 | - | 245 | 1 | --- | K.SKFDNLYGCR.H | | 1227.6704 | 1226.6631 | 1226.7095 | -37.81 | 368 | - | 377 | 1 | --- | K.RITIKPQTDR.W | | 1235.5934 | 1234.5861 | 1234.6306 | -36.02 | 16 | - | 26 | 1 | --- | K.VKDLSQADFGR.L | | 1235.5934 | 1234.5861 | 1234.6306 | -36.02 | 16 | - | 26 | 1 | 53 | K.VKDLSQADFGR.L | | 1245.5642 | 1244.5569 | 1244.6257 | -55.26 | 329 | - | 338 | 1 | --- | K.DIIMVDHMKK.M + Oxidation (M) | | 1319.6542 | 1318.6469 | 1318.6980 | -38.75 | 199 | - | 211 | 0 | --- | R.LVGVSEETTTGVK.R | | 1475.7511 | 1474.7438 | 1474.7991 | -37.50 | 199 | - | 212 | 1 | --- | R.LVGVSEETTTGVKR.L | | 1887.9293 | 1886.9220 | 1886.9890 | -35.52 | 378 | - | 394 | 0 | --- | R.WVFPETNSGIIVLAEGR.L | | 1903.9103 | 1902.9030 | 1902.9253 | -11.69 | 27 | - | 43 | 0 | --- | R.LEIELAEVEMPGLMACR.A | | 1903.9103 | 1902.9030 | 1902.9253 | -11.69 | 27 | - | 43 | 0 | 12 | R.LEIELAEVEMPGLMACR.A | | 1919.9082 | 1918.9009 | 1918.9202 | -10.04 | 27 | - | 43 | 0 | --- | R.LEIELAEVEMPGLMACR.A + Oxidation (M) | | 1927.9327 | 1926.9254 | 1926.9655 | -20.82 | 246 | - | 263 | 1 | --- | R.HSLPDGLMRATDVMIAGK.V + Oxidation (M) | | 2213.0984 | 2212.0911 | 2212.1739 | -37.43 | 163 | - | 182 | 0 | --- | K.LPDPASTDNAEFQIVLTIIR.D | | 2213.0984 | 2212.0911 | 2212.1739 | -37.43 | 163 | - | 182 | 0 | 97 | K.LPDPASTDNAEFQIVLTIIR.D | | 2309.0327 | 2308.0254 | 2308.1124 | -37.67 | 465 | - | 484 | 0 | --- | K.EQADYISVSADGPYKPLHYR.Y | | 2309.0327 | 2308.0254 | 2308.1124 | -37.67 | 465 | - | 484 | 0 | --- | K.EQADYISVSADGPYKPLHYR.Y |  | **No match to:** 713.3962, 730.4034, 750.3923, 768.5092, 780.3427, 832.2772, 842.4770, 850.5056, 857.4358, 868.5192, 882.5319, 975.4811, 976.4769, 977.4822, 999.5577, 1014.4574, 1021.4938, 1026.4636, 1030.4274, 1044.4152, 1044.4152, 1055.4718, 1060.4222, 1063.4658, 1066.4135, 1102.5920, 1129.4918, 1130.5292, 1145.4679, 1168.5364, 1168.5364, 1179.5599, 1187.6127, 1190.5131, 1217.5887, 1223.5656, 1246.5165, 1259.5320, 1259.5320, 1275.5431, 1281.5328, 1306.6196, 1447.6798, 1465.7010, 1471.7100, 1471.7100, 1636.8286, 1729.7018, 1745.6997, 1757.8507, 1778.9567, 1783.7983, 1832.8146, 1864.7954, 1870.9056, 1872.8828, 1873.8914, 1874.9020, 1885.8987, 1891.9194, 1891.9194, 1900.9349, 1901.8983, 1902.8894, 1913.8623, 1917.9104, 1928.8689, 1928.8689, 1933.9032, 1976.8738, 1992.8818, 1992.8818, 2008.8901, 2041.0209, 2087.9045, 2091.9082, 2161.8767, 2178.8992, 2178.8992, 2182.9014, 2194.8965, 2210.8962, 2291.0205, 2308.0557, 2331.0164, 2891.3555, 3049.3972 | | --- |   Unidentified protein spots   | **G13.** | [**Cs9g04750.2**](http://localhost/mascot/cgi/protein_view.pl?file=../data/20140815/F004023.dat&hit=1)**Mass:**42191  **Score:**45  **Expect:**1.5**Matches:**13 | | --- | --- |  | **Observed** | **Mr(expt)** | **Mr(calc)** | **ppm** | **Start** |  | **End** | **Miss** | **Ions** | **Peptide** | | --- | --- | --- | --- | --- | --- | --- | --- | --- | --- | | 815.3958 | 814.3885 | 814.4661 | -95.26 | 350 | - | 357 | 1 | --- | R.ERAVAAAK.V | | 860.5678 | 859.5605 | 859.4949 | 76.3 | 55 | - | 63 | 1 | --- | R.MKIAAGAAK.G | | 860.5678 | 859.5605 | 859.4949 | 76.3 | 55 | - | 63 | 1 | --- | R.MKIAAGAAK.G | | 992.6395 | 991.6322 | 991.4570 | 177 | 297 | - | 306 | 0 | --- | R.DGSTGAELSR.N | | 1030.5293 | 1029.5220 | 1029.4879 | 33.1 | 47 | - | 54 | 0 | --- | K.EPLDWNTR.M | | 1030.5293 | 1029.5220 | 1029.4879 | 33.1 | 47 | - | 54 | 0 | 109 | K.EPLDWNTR.M | | 1456.7108 | 1455.7035 | 1455.7358 | -22.16 | 83 | - | 95 | 0 | --- | K.SSNILLDEGFHPK.L | | 1457.7296 | 1456.7223 | 1456.7575 | -24.15 | 352 | - | 364 | 1 | --- | R.AVAAAKVWGENWR.E | | 1812.7953 | 1811.7880 | 1811.9417 | -84.84 | 80 | - | 95 | 1 | --- | R.DLKSSNILLDEGFHPK.L | | 1812.7953 | 1811.7880 | 1811.9417 | -84.84 | 80 | - | 95 | 1 | --- | R.DLKSSNILLDEGFHPK.L | | 1829.8168 | 1828.8095 | 1828.7758 | 18.4 | 260 | - | 276 | 1 | --- | R.SMADGQDSPDEHGRVGR.H + Oxidation (M) | | 1829.8168 | 1828.8095 | 1828.7758 | 18.4 | 260 | - | 276 | 1 | --- | R.SMADGQDSPDEHGRVGR.H + Oxidation (M) |  | **No match to:** 715.3916, 728.5037, 734.4288, 772.5234, 816.5444, 842.4316, 904.5896, 904.5896, 948.6187, 1046.5387, 1057.4077, 1057.4077, 1213.4948, 1213.4948, 1238.5795, 1414.6974, 1414.6974, 1440.7141, 1458.7255, 1458.7255, 1465.6577, 1472.7284, 1474.7230, 1480.6981, 1496.6758, 1512.6433, 1512.6433, 1693.8163, 1699.8909, 1699.8909, 1806.8756, 1811.8124, 1827.8226, 1828.8334, 1834.7698, 1835.7698, 1843.8187, 1843.8187, 1851.7766, 1867.7621, 2014.9835, 2014.9835, 2036.9507, 2048.0415, 2048.0415, 2052.9436, 2171.0667, 2171.0667 | | --- | |
| --- | --- | --- | --- | --- | --- | --- | --- | --- | --- | --- | --- | --- | --- | --- | --- | --- | --- | --- | --- | --- | --- | --- | --- | --- | --- | --- | --- | --- | --- | --- | --- | --- | --- | --- | --- | --- | --- | --- | --- | --- | --- | --- | --- | --- | --- | --- | --- | --- | --- | --- | --- | --- | --- | --- | --- | --- | --- | --- | --- | --- | --- | --- | --- | --- | --- | --- | --- | --- | --- | --- | --- | --- | --- | --- | --- | --- | --- | --- | --- | --- | --- | --- | --- | --- | --- | --- | --- | --- | --- | --- | --- | --- | --- | --- | --- | --- | --- | --- | --- | --- | --- | --- | --- | --- | --- | --- | --- | --- | --- | --- | --- | --- | --- | --- | --- | --- | --- | --- | --- | --- | --- | --- | --- | --- | --- | --- | --- | --- | --- | --- | --- | --- | --- | --- | --- | --- | --- | --- | --- | --- | --- | --- | --- | --- | --- | --- | --- | --- | --- | --- | --- | --- | --- | --- | --- | --- | --- | --- | --- | --- | --- | --- | --- | --- | --- | --- | --- | --- | --- | --- | --- | --- | --- | --- | --- | --- | --- | --- | --- | --- | --- | --- | --- | --- | --- | --- | --- | --- | --- | --- | --- | --- | --- | --- | --- | --- | --- | --- | --- | --- | --- | --- | --- | --- | --- | --- | --- | --- | --- | --- | --- | --- | --- | --- | --- | --- | --- | --- | --- | --- | --- | --- | --- | --- | --- | --- | --- | --- | --- | --- | --- | --- | --- | --- | --- | --- | --- | --- | --- | --- | --- | --- | --- | --- | --- | --- | --- | --- | --- | --- | --- | --- | --- | --- | --- | --- | --- | --- | --- | --- | --- | --- | --- | --- | --- | --- | --- | --- | --- | --- | --- | --- | --- | --- | --- | --- | --- | --- | --- | --- | --- | --- | --- | --- | --- | --- | --- | --- | --- | --- | --- | --- | --- | --- | --- | --- | --- | --- | --- | --- | --- | --- | --- | --- | --- | --- | --- | --- | --- | --- | --- | --- | --- | --- | --- | --- | --- | --- | --- | --- | --- | --- | --- | --- | --- | --- | --- | --- | --- | --- | --- | --- | --- | --- | --- | --- | --- | --- | --- | --- | --- | --- | --- | --- | --- | --- | --- | --- | --- | --- | --- | --- | --- | --- | --- | --- | --- | --- | --- | --- | --- | --- | --- | --- | --- | --- | --- | --- | --- | --- | --- | --- | --- | --- | --- | --- | --- | --- | --- | --- | --- | --- | --- | --- | --- | --- | --- | --- | --- | --- | --- | --- | --- | --- | --- | --- | --- | --- | --- | --- | --- | --- | --- | --- | --- | --- | --- | --- | --- | --- | --- | --- | --- | --- | --- | --- | --- | --- | --- | --- | --- | --- | --- | --- | --- | --- | --- | --- | --- | --- | --- | --- | --- | --- | --- | --- | --- | --- | --- | --- | --- | --- | --- | --- | --- | --- | --- | --- | --- | --- | --- | --- | --- | --- | --- | --- | --- | --- | --- | --- | --- | --- | --- | --- | --- | --- | --- | --- | --- | --- | --- | --- | --- | --- | --- | --- | --- | --- | --- | --- | --- | --- | --- | --- | --- | --- | --- | --- | --- | --- | --- | --- | --- | --- | --- | --- | --- | --- | --- | --- | --- | --- | --- | --- | --- | --- | --- | --- | --- | --- | --- | --- | --- | --- | --- | --- | --- | --- | --- | --- | --- | --- | --- | --- | --- | --- | --- | --- | --- | --- | --- | --- | --- | --- | --- | --- | --- | --- | --- | --- | --- | --- | --- | --- | --- | --- | --- | --- | --- | --- | --- | --- | --- | --- | --- | --- | --- | --- | --- | --- | --- | --- | --- | --- | --- | --- | --- | --- | --- | --- | --- | --- | --- | --- | --- | --- | --- | --- | --- | --- | --- | --- | --- | --- | --- | --- | --- | --- | --- | --- | --- | --- | --- | --- | --- | --- | --- | --- | --- | --- | --- | --- | --- | --- | --- | --- | --- | --- | --- | --- | --- | --- | --- | --- | --- | --- | --- | --- | --- | --- | --- | --- | --- | --- | --- | --- | --- | --- | --- | --- | --- | --- | --- | --- | --- | --- | --- | --- | --- | --- | --- | --- | --- | --- | --- | --- | --- | --- | --- | --- | --- | --- | --- | --- | --- | --- | --- | --- | --- | --- | --- | --- | --- | --- | --- | --- | --- | --- | --- | --- | --- | --- | --- | --- | --- | --- | --- | --- | --- | --- | --- | --- | --- | --- | --- | --- | --- | --- | --- | --- | --- | --- | --- | --- | --- | --- | --- | --- | --- | --- | --- | --- | --- | --- | --- | --- | --- | --- | --- | --- | --- | --- | --- | --- | --- | --- | --- | --- | --- | --- | --- | --- | --- | --- | --- | --- | --- | --- | --- | --- | --- | --- | --- | --- | --- | --- | --- | --- | --- | --- | --- | --- | --- | --- | --- | --- | --- | --- | --- | --- | --- | --- | --- | --- | --- | --- | --- | --- | --- | --- | --- | --- | --- | --- | --- | --- | --- | --- | --- | --- | --- | --- | --- | --- | --- | --- | --- | --- | --- | --- | --- | --- | --- | --- | --- | --- | --- | --- | --- | --- | --- | --- | --- | --- | --- | --- | --- | --- | --- | --- | --- | --- | --- | --- | --- | --- | --- | --- | --- | --- | --- | --- | --- | --- | --- | --- | --- | --- | --- | --- | --- | --- | --- | --- | --- | --- | --- | --- | --- | --- | --- | --- | --- | --- | --- | --- | --- | --- | --- | --- | --- | --- | --- | --- | --- | --- | --- | --- | --- | --- | --- | --- | --- | --- | --- | --- | --- | --- | --- | --- | --- | --- | --- | --- | --- | --- | --- | --- | --- | --- | --- | --- | --- | --- | --- | --- | --- | --- | --- | --- | --- | --- | --- | --- | --- | --- | --- | --- | --- | --- | --- | --- | --- | --- | --- | --- | --- | --- | --- | --- | --- | --- | --- | --- | --- | --- | --- | --- | --- | --- | --- | --- | --- | --- | --- | --- | --- | --- | --- | --- | --- | --- | --- | --- | --- | --- | --- | --- | --- | --- | --- | --- | --- | --- | --- | --- | --- | --- | --- | --- | --- | --- | --- | --- | --- | --- | --- | --- | --- | --- | --- | --- | --- | --- | --- | --- | --- | --- | --- | --- | --- | --- | --- | --- | --- | --- | --- | --- | --- | --- | --- | --- | --- | --- | --- | --- | --- | --- | --- | --- | --- | --- | --- | --- | --- | --- | --- | --- | --- | --- | --- | --- | --- | --- | --- | --- | --- | --- | --- | --- | --- | --- | --- | --- | --- | --- | --- | --- | --- | --- | --- | --- | --- | --- | --- | --- | --- | --- | --- | --- | --- | --- | --- | --- | --- | --- | --- | --- | --- | --- | --- | --- | --- | --- | --- | --- | --- | --- | --- | --- | --- | --- | --- | --- | --- | --- | --- | --- | --- | --- | --- | --- | --- | --- | --- | --- | --- | --- | --- | --- | --- | --- | --- | --- | --- | --- | --- | --- | --- | --- | --- | --- | --- | --- | --- | --- | --- | --- | --- | --- | --- | --- | --- | --- | --- | --- | --- | --- | --- | --- | --- | --- | --- | --- | --- | --- | --- | --- | --- | --- | --- | --- | --- | --- | --- | --- | --- | --- | --- | --- | --- | --- | --- | --- | --- | --- | --- | --- | --- | --- | --- | --- | --- | --- | --- | --- | --- | --- | --- | --- | --- | --- | --- | --- | --- | --- | --- | --- | --- | --- | --- | --- | --- | --- | --- | --- | --- | --- | --- | --- | --- | --- | --- | --- | --- | --- | --- | --- | --- | --- | --- | --- | --- | --- | --- | --- | --- | --- | --- | --- | --- | --- | --- | --- | --- | --- | --- | --- | --- | --- | --- | --- | --- | --- | --- | --- | --- | --- | --- | --- | --- | --- | --- | --- | --- | --- | --- | --- | --- | --- | --- | --- | --- | --- | --- | --- | --- | --- | --- | --- | --- | --- | --- | --- | --- | --- | --- | --- | --- | --- | --- | --- | --- | --- | --- | --- | --- | --- | --- | --- | --- | --- | --- | --- | --- | --- | --- | --- | --- | --- | --- | --- | --- | --- | --- | --- | --- | --- | --- | --- | --- | --- | --- | --- | --- | --- | --- | --- | --- | --- | --- | --- | --- | --- | --- | --- | --- | --- | --- | --- | --- | --- | --- | --- | --- | --- | --- | --- | --- | --- | --- | --- | --- | --- | --- | --- | --- | --- | --- | --- | --- | --- | --- | --- | --- | --- | --- | --- | --- | --- | --- | --- | --- | --- | --- | --- | --- | --- | --- | --- | --- | --- | --- | --- | --- | --- | --- | --- | --- | --- | --- | --- | --- | --- | --- | --- | --- | --- | --- | --- | --- | --- | --- | --- | --- | --- | --- | --- | --- | --- | --- | --- | --- | --- | --- | --- | --- | --- | --- | --- | --- | --- | --- | --- | --- | --- | --- | --- | --- | --- | --- | --- | --- | --- | --- | --- | --- | --- | --- | --- | --- | --- | --- | --- | --- | --- | --- | --- | --- | --- | --- | --- | --- | --- | --- | --- | --- | --- | --- | --- | --- | --- | --- | --- | --- | --- | --- | --- | --- | --- | --- | --- | --- | --- | --- | --- | --- | --- | --- | --- | --- | --- | --- | --- | --- | --- | --- | --- | --- | --- | --- | --- | --- | --- | --- | --- | --- | --- | --- | --- | --- | --- | --- | --- | --- | --- | --- | --- | --- | --- | --- | --- | --- | --- | --- | --- | --- | --- | --- | --- | --- | --- | --- | --- | --- | --- | --- | --- | --- | --- | --- | --- | --- | --- | --- | --- | --- | --- | --- | --- | --- | --- | --- | --- | --- | --- | --- | --- | --- | --- | --- | --- | --- | --- | --- | --- | --- | --- | --- | --- | --- | --- | --- | --- | --- | --- | --- | --- | --- | --- | --- | --- | --- | --- | --- | --- | --- | --- | --- | --- | --- | --- | --- | --- | --- | --- | --- | --- | --- | --- | --- | --- | --- | --- | --- | --- | --- | --- | --- | --- | --- | --- | --- | --- | --- | --- | --- | --- | --- | --- | --- | --- | --- | --- | --- | --- | --- | --- | --- | --- | --- | --- | --- | --- | --- | --- | --- | --- | --- | --- | --- | --- | --- | --- | --- | --- | --- | --- | --- | --- | --- | --- | --- | --- | --- | --- | --- | --- | --- | --- | --- | --- | --- | --- | --- | --- | --- | --- | --- | --- | --- | --- | --- | --- | --- | --- | --- | --- | --- | --- | --- | --- | --- | --- | --- | --- | --- | --- | --- | --- | --- | --- | --- | --- | --- | --- | --- | --- | --- | --- | --- | --- | --- | --- | --- | --- | --- | --- | --- | --- | --- | --- | --- | --- | --- | --- | --- | --- | --- | --- | --- | --- | --- | --- | --- | --- | --- | --- | --- | --- | --- | --- | --- | --- | --- | --- | --- | --- | --- | --- | --- | --- | --- | --- | --- | --- | --- | --- | --- | --- | --- | --- | --- | --- | --- | --- | --- | --- | --- | --- | --- | --- | --- | --- | --- | --- | --- | --- | --- | --- | --- | --- | --- | --- | --- | --- | --- | --- | --- | --- | --- | --- | --- | --- | --- | --- | --- | --- | --- | --- | --- | --- | --- | --- | --- | --- | --- | --- | --- | --- | --- | --- | --- | --- | --- | --- | --- | --- | --- | --- | --- | --- | --- | --- | --- | --- | --- | --- | --- | --- | --- | --- | --- | --- | --- | --- | --- | --- | --- | --- | --- | --- | --- | --- | --- | --- | --- | --- | --- | --- | --- | --- | --- | --- | --- | --- | --- | --- | --- | --- | --- | --- | --- | --- | --- | --- | --- | --- | --- | --- | --- | --- | --- | --- | --- | --- | --- | --- | --- | --- | --- | --- | --- | --- | --- | --- | --- | --- | --- | --- | --- | --- | --- | --- | --- | --- | --- | --- | --- | --- | --- | --- | --- | --- | --- | --- | --- | --- | --- | --- | --- | --- | --- | --- | --- | --- | --- | --- | --- | --- | --- | --- | --- | --- | --- | --- | --- | --- | --- | --- | --- | --- | --- | --- | --- | --- | --- | --- | --- | --- | --- | --- | --- | --- | --- | --- | --- | --- | --- | --- | --- | --- | --- | --- | --- | --- | --- | --- | --- | --- | --- | --- | --- | --- | --- | --- | --- | --- | --- | --- | --- | --- | --- | --- | --- | --- | --- | --- | --- | --- | --- | --- | --- | --- | --- | --- | --- | --- | --- | --- | --- | --- | --- | --- | --- | --- | --- | --- | --- | --- | --- | --- | --- | --- | --- | --- | --- | --- | --- | --- | --- | --- | --- | --- | --- | --- | --- | --- | --- | --- | --- | --- | --- | --- | --- | --- | --- | --- | --- | --- | --- | --- | --- | --- | --- | --- | --- | --- | --- | --- | --- | --- | --- | --- | --- | --- | --- | --- | --- | --- | --- | --- | --- | --- | --- | --- | --- | --- | --- | --- | --- | --- | --- | --- | --- | --- | --- | --- | --- | --- | --- | --- | --- | --- | --- | --- | --- | --- | --- | --- | --- | --- | --- | --- | --- | --- | --- | --- | --- | --- | --- | --- | --- | --- | --- | --- | --- | --- | --- | --- | --- | --- | --- | --- | --- | --- | --- | --- | --- | --- | --- | --- | --- | --- | --- | --- | --- | --- | --- | --- | --- | --- | --- | --- | --- | --- | --- | --- | --- | --- | --- | --- | --- | --- | --- | --- | --- | --- | --- | --- | --- | --- | --- | --- | --- | --- | --- | --- | --- | --- | --- | --- | --- | --- | --- | --- | --- | --- | --- | --- | --- | --- | --- | --- | --- | --- | --- | --- | --- | --- | --- | --- | --- | --- | --- | --- | --- | --- | --- | --- | --- | --- | --- | --- | --- | --- | --- | --- | --- | --- | --- | --- | --- | --- | --- | --- | --- | --- | --- | --- | --- | --- | --- | --- | --- | --- | --- | --- | --- | --- | --- | --- | --- | --- | --- | --- | --- | --- | --- | --- | --- | --- | --- | --- | --- | --- | --- | --- | --- | --- | --- | --- | --- | --- | --- | --- | --- | --- | --- | --- | --- | --- | --- | --- | --- | --- | --- | --- | --- | --- | --- | --- | --- | --- | --- | --- | --- | --- | --- | --- | --- | --- | --- | --- | --- | --- | --- | --- | --- | --- | --- | --- | --- | --- | --- | --- | --- | --- | --- | --- | --- | --- | --- | --- | --- | --- | --- | --- | --- | --- | --- | --- | --- | --- | --- | --- | --- | --- | --- | --- | --- | --- | --- | --- | --- | --- | --- | --- | --- | --- | --- | --- | --- | --- | --- | --- | --- | --- | --- | --- | --- | --- | --- | --- | --- | --- | --- | --- | --- | --- | --- | --- | --- | --- | --- | --- | --- | --- | --- | --- | --- | --- | --- | --- | --- | --- | --- | --- | --- | --- | --- | --- | --- | --- | --- | --- | --- | --- | --- | --- | --- | --- | --- | --- | --- | --- | --- | --- | --- | --- | --- | --- | --- | --- | --- | --- | --- | --- | --- | --- | --- | --- | --- | --- | --- | --- | --- | --- | --- | --- | --- | --- | --- | --- | --- | --- | --- | --- | --- | --- | --- | --- | --- | --- | --- | --- | --- | --- | --- | --- | --- | --- | --- | --- | --- | --- | --- | --- | --- | --- | --- | --- | --- | --- | --- | --- | --- | --- | --- | --- | --- | --- | --- | --- | --- | --- | --- | --- | --- | --- | --- | --- | --- | --- | --- | --- | --- | --- | --- | --- | --- | --- | --- | --- | --- | --- | --- | --- | --- | --- | --- | --- | --- | --- | --- | --- | --- | --- | --- | --- | --- | --- | --- | --- | --- | --- | --- | --- | --- | --- | --- | --- | --- | --- | --- | --- | --- | --- | --- | --- | --- | --- | --- | --- | --- | --- | --- | --- | --- | --- | --- | --- | --- | --- | --- | --- | --- | --- | --- | --- | --- | --- | --- | --- | --- | --- | --- | --- | --- | --- | --- | --- | --- | --- | --- | --- | --- | --- | --- | --- | --- | --- | --- | --- | --- | --- | --- | --- | --- | --- | --- | --- | --- | --- | --- | --- | --- | --- | --- | --- | --- | --- | --- | --- | --- | --- | --- | --- | --- | --- | --- | --- | --- | --- | --- | --- | --- | --- | --- | --- | --- | --- | --- | --- | --- | --- | --- | --- | --- | --- | --- | --- | --- | --- | --- | --- | --- | --- | --- | --- | --- | --- | --- | --- | --- | --- | --- | --- | --- | --- | --- | --- | --- | --- | --- | --- | --- | --- | --- | --- | --- | --- | --- | --- | --- | --- | --- | --- | --- | --- | --- | --- | --- | --- | --- | --- | --- | --- | --- | --- | --- | --- | --- | --- | --- | --- | --- | --- | --- | --- | --- | --- | --- | --- | --- | --- | --- | --- | --- | --- | --- | --- | --- | --- | --- | --- | --- | --- | --- | --- | --- | --- | --- | --- | --- | --- | --- | --- | --- | --- | --- | --- | --- | --- | --- | --- | --- | --- | --- | --- | --- | --- | --- | --- | --- | --- | --- | --- | --- | --- | --- | --- | --- | --- | --- | --- | --- | --- | --- | --- | --- | --- | --- | --- | --- | --- | --- | --- | --- | --- | --- | --- | --- | --- | --- | --- | --- | --- | --- | --- | --- | --- | --- | --- | --- | --- | --- | --- | --- | --- | --- | --- | --- | --- | --- | --- | --- | --- | --- | --- | --- | --- | --- | --- | --- | --- | --- | --- | --- | --- | --- | --- | --- | --- | --- | --- | --- | --- | --- | --- | --- | --- | --- | --- | --- | --- | --- | --- | --- | --- | --- | --- | --- | --- | --- | --- | --- | --- | --- | --- | --- | --- | --- | --- | --- | --- | --- | --- | --- | --- | --- | --- | --- | --- | --- | --- | --- | --- | --- | --- | --- | --- | --- | --- | --- | --- | --- | --- | --- | --- | --- | --- | --- | --- | --- | --- | --- | --- | --- | --- | --- | --- | --- | --- | --- | --- | --- | --- | --- | --- | --- | --- | --- | --- | --- | --- | --- | --- | --- | --- | --- | --- | --- | --- | --- | --- | --- | --- | --- | --- | --- | --- | --- | --- | --- | --- | --- | --- | --- | --- | --- | --- | --- | --- | --- | --- | --- | --- | --- | --- | --- | --- | --- | --- | --- | --- | --- | --- | --- | --- | --- | --- | --- | --- | --- | --- | --- | --- | --- | --- | --- | --- | --- | --- | --- | --- | --- | --- | --- | --- | --- | --- | --- | --- | --- | --- | --- | --- | --- | --- | --- | --- | --- | --- | --- | --- | --- | --- | --- | --- | --- | --- | --- | --- | --- | --- | --- | --- | --- | --- | --- | --- | --- | --- | --- | --- | --- | --- | --- | --- | --- | --- | --- | --- | --- | --- | --- | --- | --- | --- | --- | --- | --- | --- | --- | --- | --- | --- | --- | --- | --- | --- | --- | --- | --- | --- | --- | --- | --- | --- | --- | --- | --- | --- | --- | --- | --- | --- | --- | --- | --- | --- | --- | --- | --- | --- | --- | --- | --- | --- | --- | --- | --- | --- | --- | --- | --- | --- | --- | --- | --- | --- | --- | --- | --- | --- | --- | --- | --- | --- | --- | --- | --- | --- | --- | --- | --- | --- | --- | --- | --- | --- | --- | --- | --- | --- | --- | --- | --- | --- | --- | --- | --- | --- | --- | --- | --- | --- | --- | --- | --- | --- | --- | --- | --- | --- | --- | --- | --- | --- | --- | --- | --- | --- | --- | --- | --- | --- | --- | --- | --- | --- | --- | --- | --- | --- | --- | --- | --- | --- | --- | --- | --- | --- | --- | --- | --- | --- | --- | --- | --- | --- | --- | --- | --- | --- | --- | --- | --- | --- | --- | --- | --- | --- | --- | --- | --- | --- | --- | --- | --- | --- | --- | --- | --- | --- | --- | --- | --- | --- | --- | --- | --- | --- | --- | --- | --- | --- | --- | --- | --- | --- | --- | --- | --- | --- | --- | --- | --- | --- | --- | --- | --- | --- | --- | --- | --- | --- | --- | --- | --- | --- | --- | --- | --- | --- | --- | --- | --- | --- | --- | --- | --- | --- | --- | --- | --- | --- | --- | --- | --- | --- | --- | --- | --- | --- | --- | --- | --- | --- | --- | --- | --- | --- | --- | --- | --- | --- | --- | --- | --- | --- | --- | --- | --- | --- | --- | --- | --- | --- | --- | --- | --- | --- | --- | --- | --- | --- | --- | --- | --- | --- | --- | --- | --- | --- | --- | --- | --- | --- | --- | --- | --- | --- | --- | --- | --- | --- | --- | --- | --- | --- | --- | --- | --- | --- | --- | --- | --- | --- | --- | --- | --- | --- | --- | --- | --- | --- | --- | --- | --- | --- | --- | --- | --- | --- | --- | --- | --- | --- | --- | --- | --- | --- | --- | --- | --- | --- | --- | --- | --- | --- | --- | --- | --- | --- | --- | --- | --- | --- | --- | --- | --- | --- | --- | --- | --- | --- | --- | --- | --- | --- | --- | --- | --- | --- | --- | --- | --- | --- | --- | --- | --- | --- | --- | --- | --- | --- | --- | --- | --- | --- | --- | --- | --- | --- | --- | --- | --- | --- | --- | --- | --- | --- | --- | --- | --- | --- | --- | --- | --- | --- | --- | --- | --- | --- | --- | --- | --- | --- | --- | --- | --- | --- | --- | --- | --- | --- | --- | --- | --- | --- | --- | --- | --- | --- | --- | --- | --- | --- | --- | --- | --- | --- | --- | --- | --- | --- | --- | --- | --- | --- | --- | --- | --- | --- | --- | --- | --- | --- | --- | --- | --- | --- | --- | --- | --- | --- | --- | --- | --- | --- | --- | --- | --- | --- | --- | --- | --- | --- | --- | --- | --- | --- | --- | --- | --- | --- | --- | --- | --- | --- | --- | --- | --- | --- | --- | --- | --- | --- | --- | --- | --- | --- | --- | --- | --- | --- | --- | --- | --- | --- | --- | --- | --- | --- | --- | --- | --- | --- | --- | --- | --- | --- | --- | --- | --- | --- | --- | --- | --- | --- | --- | --- | --- | --- | --- | --- | --- | --- | --- | --- | --- | --- | --- | --- | --- | --- | --- | --- | --- | --- | --- | --- | --- | --- | --- | --- | --- | --- | --- | --- | --- | --- | --- | --- | --- | --- | --- | --- | --- | --- | --- | --- | --- | --- | --- | --- | --- | --- | --- | --- | --- | --- | --- | --- | --- | --- | --- | --- | --- | --- | --- | --- | --- | --- | --- | --- | --- | --- | --- | --- | --- | --- | --- | --- | --- | --- | --- | --- | --- | --- | --- | --- | --- | --- | --- | --- | --- | --- | --- | --- | --- | --- | --- | --- | --- | --- | --- | --- | --- | --- | --- | --- | --- | --- | --- | --- | --- | --- | --- | --- | --- | --- | --- | --- | --- | --- | --- | --- | --- | --- | --- | --- | --- | --- | --- | --- | --- | --- | --- | --- | --- | --- | --- | --- | --- | --- | --- | --- | --- | --- | --- | --- | --- | --- | --- | --- | --- | --- | --- | --- | --- | --- | --- | --- | --- | --- | --- | --- | --- | --- | --- | --- | --- | --- | --- | --- | --- | --- | --- | --- | --- | --- | --- | --- | --- | --- | --- | --- | --- | --- | --- | --- | --- | --- | --- | --- | --- | --- | --- | --- | --- | --- | --- | --- | --- | --- | --- | --- | --- | --- | --- | --- | --- | --- | --- | --- | --- | --- | --- | --- | --- | --- | --- | --- | --- | --- | --- | --- | --- | --- | --- | --- | --- | --- | --- | --- | --- | --- | --- | --- | --- | --- | --- | --- | --- | --- | --- | --- | --- | --- | --- | --- | --- | --- | --- | --- | --- | --- | --- | --- | --- | --- | --- | --- | --- | --- | --- | --- | --- | --- | --- | --- | --- | --- | --- | --- | --- | --- | --- | --- | --- | --- | --- | --- | --- | --- | --- | --- | --- | --- | --- | --- | --- | --- | --- | --- | --- | --- | --- | --- | --- | --- | --- | --- | --- | --- | --- | --- | --- | --- | --- | --- | --- | --- | --- | --- | --- | --- | --- | --- | --- | --- | --- | --- | --- | --- | --- | --- | --- | --- | --- | --- | --- | --- | --- | --- | --- | --- | --- | --- | --- | --- | --- | --- | --- | --- | --- | --- | --- | --- | --- | --- | --- | --- | --- | --- | --- | --- | --- | --- | --- | --- | --- | --- | --- | --- | --- | --- | --- | --- | --- | --- | --- | --- | --- | --- | --- | --- | --- | --- | --- | --- | --- | --- | --- | --- | --- | --- | --- | --- | --- | --- | --- | --- | --- | --- | --- | --- | --- | --- | --- | --- | --- | --- | --- | --- | --- | --- | --- | --- | --- | --- | --- | --- | --- | --- | --- | --- | --- | --- | --- | --- | --- | --- | --- | --- | --- | --- | --- | --- | --- | --- | --- | --- | --- | --- | --- | --- | --- | --- | --- | --- | --- | --- | --- | --- | --- | --- | --- | --- | --- | --- | --- | --- | --- | --- | --- | --- | --- | --- | --- | --- | --- | --- | --- | --- | --- | --- | --- | --- | --- | --- | --- | --- | --- | --- | --- | --- | --- | --- | --- | --- | --- | --- | --- | --- | --- | --- | --- | --- | --- | --- | --- | --- | --- | --- | --- | --- | --- | --- | --- | --- | --- | --- | --- | --- | --- | --- | --- | --- | --- | --- | --- | --- | --- | --- | --- | --- | --- | --- | --- | --- | --- | --- | --- | --- | --- | --- | --- | --- | --- | --- | --- | --- | --- | --- | --- | --- | --- | --- | --- | --- | --- | --- | --- | --- | --- | --- | --- | --- | --- | --- | --- | --- | --- | --- | --- | --- | --- | --- | --- | --- | --- | --- | --- | --- | --- | --- | --- | --- | --- | --- | --- | --- | --- | --- | --- | --- | --- | --- | --- | --- | --- | --- | --- | --- | --- | --- | --- | --- | --- | --- | --- | --- | --- | --- | --- | --- | --- | --- | --- | --- | --- | --- | --- | --- | --- | --- | --- | --- | --- | --- | --- | --- | --- | --- | --- | --- | --- | --- | --- | --- | --- | --- | --- | --- | --- | --- | --- | --- | --- | --- | --- | --- | --- | --- | --- | --- | --- | --- | --- | --- | --- | --- | --- | --- | --- | --- | --- | --- | --- | --- | --- | --- | --- | --- | --- | --- | --- | --- | --- | --- | --- | --- | --- | --- | --- | --- | --- | --- | --- | --- | --- | --- | --- | --- | --- | --- | --- | --- | --- | --- | --- | --- | --- | --- | --- | --- | --- | --- | --- | --- | --- | --- | --- | --- | --- | --- | --- | --- | --- | --- | --- | --- | --- | --- | --- | --- | --- | --- | --- | --- | --- | --- | --- | --- | --- | --- | --- | --- | --- | --- | --- | --- | --- | --- | --- | --- | --- | --- | --- | --- | --- | --- | --- | --- | --- | --- | --- | --- | --- | --- | --- | --- | --- | --- | --- | --- | --- | --- | --- | --- | --- | --- | --- | --- | --- | --- | --- | --- | --- | --- | --- | --- | --- | --- | --- | --- | --- | --- | --- | --- | --- | --- | --- | --- | --- | --- | --- | --- | --- | --- | --- | --- | --- | --- | --- | --- | --- | --- | --- | --- | --- | --- | --- | --- | --- | --- | --- | --- | --- | --- | --- | --- | --- | --- | --- | --- | --- | --- | --- | --- | --- | --- | --- | --- | --- | --- | --- | --- | --- | --- | --- | --- | --- | --- | --- | --- | --- | --- | --- | --- | --- | --- | --- | --- | --- | --- | --- | --- | --- | --- | --- | --- | --- | --- | --- | --- | --- | --- | --- | --- | --- | --- | --- | --- | --- | --- | --- | --- | --- | --- | --- | --- | --- | --- | --- | --- | --- | --- | --- | --- | --- | --- | --- | --- | --- | --- | --- | --- | --- | --- | --- | --- | --- | --- | --- | --- | --- | --- | --- | --- | --- | --- | --- | --- | --- | --- | --- | --- | --- | --- | --- | --- | --- | --- | --- | --- | --- | --- | --- | --- | --- | --- | --- | --- | --- | --- | --- | --- | --- | --- | --- | --- | --- | --- | --- | --- | --- | --- | --- | --- | --- | --- | --- | --- | --- | --- | --- | --- | --- | --- | --- | --- | --- | --- | --- | --- | --- | --- | --- | --- | --- | --- | --- | --- | --- | --- | --- | --- | --- | --- | --- | --- | --- | --- | --- | --- | --- | --- | --- | --- | --- | --- | --- | --- | --- | --- | --- | --- | --- | --- | --- | --- | --- | --- | --- | --- | --- | --- | --- | --- | --- | --- | --- | --- | --- | --- | --- | --- | --- | --- | --- | --- | --- | --- | --- | --- | --- | --- | --- | --- | --- | --- | --- | --- | --- | --- | --- | --- | --- | --- | --- | --- | --- | --- | --- | --- | --- | --- | --- | --- | --- | --- | --- | --- | --- | --- | --- | --- | --- | --- | --- | --- | --- | --- | --- | --- | --- | --- | --- | --- | --- | --- | --- | --- | --- | --- | --- | --- | --- | --- | --- | --- | --- | --- | --- | --- | --- | --- | --- | --- | --- | --- | --- | --- | --- | --- | --- | --- | --- | --- | --- | --- | --- | --- | --- | --- | --- | --- | --- | --- | --- | --- | --- | --- | --- | --- | --- | --- | --- | --- | --- | --- | --- | --- | --- | --- | --- | --- | --- | --- | --- | --- | --- | --- | --- | --- | --- | --- | --- | --- | --- | --- | --- | --- | --- | --- | --- | --- | --- | --- | --- | --- | --- | --- | --- | --- | --- | --- | --- | --- | --- | --- | --- | --- | --- | --- | --- | --- | --- | --- | --- | --- | --- | --- | --- | --- | --- | --- | --- | --- | --- | --- | --- | --- | --- | --- | --- | --- | --- | --- | --- | --- | --- | --- | --- | --- | --- | --- | --- | --- | --- | --- | --- | --- | --- | --- | --- | --- | --- | --- | --- | --- | --- | --- | --- | --- | --- | --- | --- | --- | --- | --- | --- | --- | --- | --- | --- | --- | --- | --- | --- | --- | --- | --- | --- | --- | --- | --- | --- | --- | --- | --- | --- | --- | --- | --- | --- | --- | --- | --- | --- | --- | --- | --- | --- | --- | --- | --- | --- | --- | --- | --- | --- | --- | --- | --- | --- | --- | --- | --- | --- | --- | --- | --- | --- | --- | --- | --- | --- | --- | --- | --- | --- | --- | --- | --- | --- | --- | --- | --- | --- | --- | --- | --- | --- | --- | --- | --- | --- | --- | --- | --- | --- | --- | --- | --- | --- | --- | --- | --- | --- | --- | --- | --- | --- | --- | --- | --- | --- | --- | --- | --- | --- | --- | --- | --- | --- | --- | --- | --- | --- | --- | --- | --- | --- | --- | --- | --- | --- | --- | --- | --- | --- | --- | --- | --- | --- | --- | --- | --- | --- | --- | --- | --- | --- | --- | --- | --- | --- | --- | --- | --- | --- | --- | --- | --- | --- | --- | --- | --- | --- | --- | --- | --- | --- | --- | --- | --- | --- | --- | --- | --- | --- | --- | --- | --- | --- | --- | --- | --- | --- | --- | --- | --- | --- | --- | --- | --- | --- | --- | --- | --- | --- | --- | --- | --- | --- | --- | --- | --- | --- | --- | --- | --- | --- | --- | --- | --- | --- | --- | --- | --- | --- | --- | --- | --- | --- | --- | --- | --- | --- | --- | --- | --- | --- | --- | --- | --- | --- | --- | --- | --- | --- | --- | --- | --- | --- | --- | --- | --- | --- | --- | --- | --- | --- | --- | --- | --- | --- | --- | --- | --- | --- | --- | --- | --- | --- | --- | --- | --- | --- | --- | --- | --- | --- | --- | --- | --- | --- | --- | --- | --- | --- | --- | --- | --- | --- | --- | --- | --- | --- | --- | --- | --- | --- | --- | --- | --- | --- | --- | --- | --- | --- | --- | --- | --- | --- | --- | --- | --- | --- | --- | --- | --- | --- | --- | --- | --- | --- | --- | --- | --- | --- | --- | --- | --- | --- | --- | --- | --- | --- | --- | --- | --- | --- | --- | --- | --- | --- | --- | --- | --- | --- | --- | --- | --- | --- | --- | --- | --- | --- | --- | --- | --- | --- | --- | --- | --- | --- | --- | --- | --- | --- | --- | --- | --- | --- | --- | --- | --- | --- | --- | --- | --- | --- | --- | --- | --- | --- | --- | --- | --- | --- | --- | --- | --- | --- | --- | --- | --- | --- | --- | --- | --- | --- | --- | --- | --- | --- | --- | --- | --- | --- | --- | --- | --- | --- | --- | --- | --- | --- | --- | --- | --- | --- | --- | --- | --- | --- | --- | --- | --- | --- | --- | --- | --- | --- | --- | --- | --- | --- | --- | --- | --- | --- | --- | --- | --- | --- | --- | --- | --- | --- | --- | --- | --- | --- | --- | --- | --- | --- | --- | --- | --- | --- | --- | --- | --- | --- | --- | --- | --- | --- | --- | --- | --- | --- | --- | --- | --- | --- | --- | --- | --- | --- | --- | --- | --- | --- | --- | --- | --- | --- | --- | --- | --- | --- | --- | --- | --- | --- | --- | --- | --- | --- | --- | --- | --- | --- | --- | --- | --- | --- | --- | --- | --- | --- | --- | --- | --- | --- | --- | --- | --- | --- | --- | --- | --- | --- | --- | --- | --- | --- | --- | --- | --- | --- | --- | --- | --- | --- | --- | --- | --- | --- | --- | --- | --- | --- | --- | --- | --- | --- | --- | --- | --- | --- | --- | --- | --- | --- | --- | --- | --- | --- | --- | --- | --- | --- | --- | --- | --- | --- | --- | --- | --- | --- | --- | --- | --- | --- | --- | --- | --- | --- | --- | --- | --- | --- | --- | --- | --- | --- | --- | --- | --- | --- | --- | --- | --- | --- | --- | --- | --- | --- | --- | --- | --- | --- | --- | --- | --- | --- | --- | --- | --- | --- | --- | --- | --- | --- | --- | --- | --- | --- | --- | --- | --- | --- | --- | --- | --- | --- | --- | --- | --- | --- | --- | --- | --- | --- | --- | --- | --- | --- | --- | --- | --- | --- | --- | --- | --- | --- | --- | --- | --- | --- | --- | --- | --- | --- | --- | --- | --- | --- | --- | --- | --- | --- | --- | --- | --- | --- | --- | --- | --- | --- | --- | --- | --- | --- | --- | --- | --- | --- | --- | --- | --- | --- | --- | --- | --- | --- | --- | --- | --- | --- | --- | --- | --- | --- | --- | --- | --- | --- | --- | --- | --- | --- | --- | --- | --- | --- | --- | --- | --- | --- | --- | --- | --- | --- | --- | --- | --- | --- | --- | --- | --- | --- | --- | --- | --- | --- | --- | --- | --- | --- | --- | --- | --- | --- | --- | --- | --- | --- | --- | --- | --- | --- | --- | --- | --- | --- | --- | --- | --- | --- | --- | --- | --- | --- | --- | --- | --- | --- | --- | --- | --- | --- | --- | --- | --- | --- | --- | --- | --- | --- | --- | --- | --- | --- | --- | --- | --- | --- | --- | --- | --- | --- | --- | --- | --- | --- | --- | --- | --- | --- | --- | --- | --- | --- | --- | --- | --- | --- | --- | --- | --- | --- | --- | --- | --- | --- | --- | --- | --- | --- | --- | --- | --- | --- | --- | --- | --- | --- | --- | --- | --- | --- | --- | --- | --- | --- | --- | --- | --- | --- | --- | --- | --- | --- | --- | --- | --- | --- | --- | --- | --- | --- | --- | --- | --- | --- | --- | --- | --- | --- | --- | --- | --- | --- | --- | --- | --- | --- | --- | --- | --- | --- | --- | --- | --- | --- | --- | --- | --- | --- | --- | --- | --- | --- | --- | --- | --- | --- | --- | --- | --- | --- | --- | --- | --- | --- | --- | --- | --- | --- | --- | --- | --- | --- | --- | --- | --- | --- | --- | --- | --- | --- | --- | --- | --- | --- | --- | --- | --- | --- | --- | --- | --- | --- | --- | --- | --- | --- | --- | --- | --- | --- | --- | --- | --- | --- | --- | --- | --- | --- | --- | --- | --- | --- | --- | --- | --- | --- | --- | --- | --- | --- | --- | --- | --- | --- | --- | --- | --- | --- | --- | --- | --- | --- | --- | --- | --- | --- | --- | --- | --- | --- | --- | --- | --- | --- | --- | --- | --- | --- | --- | --- | --- | --- | --- | --- | --- | --- | --- | --- | --- | --- | --- | --- | --- | --- | --- | --- | --- | --- | --- | --- | --- | --- | --- | --- | --- | --- | --- | --- | --- | --- | --- | --- | --- | --- | --- | --- | --- | --- | --- | --- | --- | --- | --- | --- | --- | --- | --- | --- | --- | --- | --- | --- | --- | --- | --- | --- | --- | --- | --- | --- | --- | --- | --- | --- | --- | --- | --- | --- | --- | --- | --- | --- | --- | --- | --- | --- | --- | --- | --- | --- | --- | --- | --- | --- | --- | --- | --- | --- | --- | --- | --- | --- | --- | --- | --- | --- | --- | --- | --- | --- | --- | --- | --- | --- | --- | --- | --- | --- | --- | --- | --- | --- | --- | --- | --- | --- | --- | --- | --- | --- | --- | --- | --- | --- | --- | --- | --- | --- | --- | --- | --- | --- | --- | --- | --- | --- | --- | --- | --- | --- | --- | --- | --- | --- | --- | --- | --- | --- | --- | --- | --- | --- | --- | --- | --- | --- | --- | --- | --- | --- | --- | --- | --- | --- | --- | --- | --- | --- | --- | --- | --- | --- | --- | --- | --- | --- | --- | --- | --- | --- | --- | --- | --- | --- | --- | --- | --- | --- | --- | --- | --- | --- | --- | --- | --- | --- | --- | --- | --- | --- | --- | --- | --- | --- | --- | --- | --- | --- | --- | --- | --- | --- | --- | --- | --- | --- | --- | --- | --- | --- | --- | --- | --- | --- | --- | --- | --- | --- | --- | --- | --- | --- | --- | --- | --- | --- | --- | --- | --- | --- | --- | --- | --- | --- | --- | --- | --- | --- | --- | --- | --- | --- | --- | --- | --- | --- | --- | --- | --- | --- | --- | --- | --- | --- | --- | --- | --- | --- | --- | --- | --- | --- | --- | --- | --- | --- | --- | --- | --- | --- | --- | --- | --- | --- | --- | --- | --- | --- | --- | --- | --- | --- | --- | --- | --- | --- | --- | --- | --- | --- | --- | --- | --- | --- | --- | --- | --- | --- | --- | --- | --- | --- | --- | --- | --- | --- | --- | --- | --- | --- | --- | --- | --- | --- | --- | --- | --- | --- | --- | --- | --- | --- | --- | --- | --- | --- | --- | --- | --- | --- | --- | --- | --- | --- | --- | --- | --- | --- | --- | --- | --- | --- | --- | --- | --- | --- | --- | --- | --- | --- | --- | --- | --- | --- | --- | --- | --- | --- | --- | --- | --- | --- | --- | --- | --- | --- | --- | --- | --- | --- | --- | --- | --- | --- | --- | --- | --- | --- | --- | --- | --- | --- | --- | --- | --- | --- | --- | --- | --- | --- | --- | --- | --- | --- | --- | --- | --- | --- | --- | --- | --- | --- | --- | --- | --- | --- | --- | --- | --- | --- | --- | --- | --- | --- | --- | --- | --- | --- | --- | --- | --- | --- | --- | --- | --- | --- | --- | --- | --- | --- | --- | --- | --- | --- | --- | --- | --- | --- | --- | --- | --- | --- | --- | --- | --- | --- | --- | --- | --- | --- | --- | --- | --- | --- | --- | --- | --- | --- | --- | --- | --- | --- | --- | --- | --- | --- | --- | --- | --- | --- | --- | --- | --- | --- | --- | --- | --- | --- | --- | --- | --- | --- | --- | --- | --- | --- | --- | --- | --- | --- | --- | --- | --- | --- | --- | --- | --- | --- | --- | --- | --- | --- | --- | --- | --- | --- | --- | --- | --- | --- | --- | --- | --- | --- | --- | --- | --- | --- | --- | --- | --- | --- | --- | --- | --- | --- | --- | --- | --- | --- | --- | --- | --- | --- | --- | --- | --- | --- | --- | --- | --- | --- | --- | --- | --- | --- | --- | --- | --- | --- | --- | --- | --- | --- | --- | --- | --- | --- | --- | --- | --- | --- | --- | --- | --- | --- | --- | --- | --- | --- | --- | --- | --- | --- | --- | --- | --- | --- | --- | --- | --- | --- | --- | --- | --- | --- | --- | --- | --- | --- | --- | --- | --- | --- | --- | --- | --- | --- | --- | --- | --- | --- | --- | --- | --- | --- | --- | --- | --- | --- | --- | --- | --- | --- | --- | --- | --- | --- | --- | --- | --- | --- | --- | --- | --- | --- | --- | --- | --- | --- | --- | --- | --- | --- | --- | --- | --- | --- | --- | --- | --- | --- | --- | --- | --- | --- | --- | --- | --- | --- | --- | --- | --- | --- | --- | --- | --- | --- | --- | --- | --- | --- | --- | --- | --- | --- | --- | --- | --- | --- | --- | --- | --- | --- | --- | --- | --- | --- | --- | --- | --- | --- | --- | --- | --- | --- | --- | --- | --- | --- | --- | --- | --- | --- | --- | --- | --- | --- | --- | --- | --- | --- | --- | --- | --- | --- | --- | --- | --- | --- | --- | --- | --- | --- | --- | --- | --- | --- | --- | --- | --- | --- | --- | --- | --- | --- | --- | --- | --- | --- | --- | --- | --- | --- | --- | --- | --- | --- | --- | --- | --- | --- | --- | --- | --- | --- | --- | --- | --- | --- | --- | --- | --- | --- | --- | --- | --- | --- | --- | --- | --- | --- | --- | --- | --- | --- | --- | --- | --- | --- | --- | --- | --- | --- | --- | --- | --- | --- | --- | --- | --- | --- | --- | --- | --- | --- | --- | --- | --- | --- | --- | --- | --- | --- | --- | --- | --- | --- | --- | --- | --- | --- | --- | --- | --- | --- | --- | --- | --- | --- | --- | --- | --- | --- | --- | --- | --- | --- | --- | --- | --- | --- | --- | --- | --- | --- | --- | --- | --- | --- | --- | --- | --- | --- | --- | --- | --- | --- | --- | --- | --- | --- | --- | --- | --- | --- | --- | --- | --- | --- | --- | --- | --- | --- | --- | --- | --- | --- | --- | --- | --- | --- | --- | --- | --- | --- | --- | --- | --- | --- | --- | --- | --- | --- | --- | --- | --- | --- | --- | --- | --- | --- | --- | --- | --- | --- | --- | --- | --- | --- | --- | --- | --- | --- | --- | --- | --- | --- | --- | --- | --- | --- | --- | --- | --- | --- | --- | --- | --- | --- | --- | --- | --- | --- | --- | --- | --- | --- | --- | --- | --- | --- | --- | --- | --- | --- | --- | --- | --- | --- | --- | --- | --- | --- | --- | --- | --- | --- | --- | --- | --- | --- | --- | --- | --- | --- | --- | --- | --- | --- | --- | --- | --- | --- | --- | --- | --- | --- | --- | --- | --- | --- | --- | --- | --- | --- | --- | --- | --- | --- | --- | --- | --- | --- | --- | --- | --- | --- | --- | --- | --- | --- | --- | --- | --- | --- | --- | --- | --- | --- | --- | --- | --- | --- | --- | --- | --- | --- | --- | --- | --- | --- | --- | --- | --- | --- | --- | --- | --- | --- | --- | --- | --- | --- | --- | --- | --- | --- | --- | --- | --- | --- | --- | --- | --- | --- | --- | --- | --- | --- | --- | --- | --- | --- | --- | --- | --- | --- | --- | --- | --- | --- | --- | --- | --- | --- | --- | --- | --- | --- | --- | --- | --- | --- | --- | --- | --- | --- | --- | --- | --- | --- | --- | --- | --- | --- | --- | --- | --- | --- | --- | --- | --- | --- | --- | --- | --- | --- | --- | --- | --- | --- | --- | --- | --- | --- | --- | --- | --- | --- | --- | --- | --- | --- | --- | --- | --- | --- | --- | --- | --- | --- | --- | --- | --- | --- | --- | --- | --- | --- | --- | --- | --- | --- | --- | --- | --- | --- | --- | --- | --- | --- | --- | --- | --- | --- | --- | --- | --- | --- | --- | --- | --- | --- | --- | --- | --- | --- | --- | --- | --- | --- | --- | --- | --- | --- | --- | --- | --- | --- | --- | --- | --- | --- | --- | --- | --- | --- | --- | --- | --- | --- | --- | --- | --- | --- | --- | --- | --- | --- | --- | --- | --- | --- | --- | --- | --- | --- | --- | --- | --- | --- | --- | --- | --- | --- | --- | --- | --- | --- | --- | --- | --- | --- | --- | --- | --- | --- | --- | --- | --- | --- | --- | --- | --- | --- | --- | --- | --- | --- | --- | --- | --- | --- | --- | --- | --- | --- | --- | --- | --- | --- | --- | --- | --- | --- | --- | --- | --- | --- | --- | --- | --- | --- | --- | --- | --- | --- | --- | --- | --- | --- | --- | --- | --- | --- | --- | --- | --- | --- | --- | --- | --- | --- | --- | --- | --- | --- | --- | --- | --- | --- | --- | --- | --- | --- | --- | --- | --- | --- | --- | --- | --- | --- | --- | --- | --- | --- | --- | --- | --- | --- | --- | --- | --- | --- | --- | --- | --- | --- | --- | --- | --- | --- | --- | --- | --- | --- | --- | --- | --- | --- | --- | --- | --- | --- | --- | --- | --- | --- | --- | --- | --- | --- | --- | --- | --- | --- | --- | --- | --- | --- | --- | --- | --- | --- | --- | --- | --- | --- | --- | --- | --- | --- | --- | --- | --- | --- | --- | --- | --- | --- | --- | --- | --- | --- | --- | --- | --- | --- | --- | --- | --- | --- | --- | --- | --- | --- | --- | --- | --- | --- | --- | --- | --- | --- | --- | --- | --- | --- | --- | --- | --- | --- | --- | --- | --- | --- | --- | --- | --- | --- | --- | --- | --- | --- | --- | --- | --- | --- | --- | --- | --- | --- | --- | --- | --- | --- | --- | --- | --- | --- | --- | --- | --- | --- | --- | --- | --- | --- | --- | --- | --- | --- | --- | --- | --- | --- | --- | --- | --- | --- | --- | --- | --- | --- | --- | --- | --- | --- | --- | --- | --- | --- | --- | --- | --- | --- | --- | --- | --- | --- | --- | --- | --- | --- | --- | --- | --- | --- | --- | --- | --- | --- | --- | --- | --- | --- | --- | --- | --- | --- | --- | --- | --- | --- | --- | --- | --- | --- | --- | --- | --- | --- | --- | --- | --- | --- | --- | --- | --- | --- | --- | --- | --- | --- | --- | --- | --- | --- | --- | --- | --- | --- | --- | --- | --- | --- | --- | --- | --- | --- | --- | --- | --- | --- | --- | --- | --- | --- | --- | --- | --- | --- | --- | --- | --- | --- | --- | --- | --- | --- | --- | --- | --- | --- | --- | --- | --- | --- | --- | --- | --- | --- | --- | --- | --- | --- | --- | --- | --- | --- | --- | --- | --- | --- | --- | --- | --- | --- | --- | --- | --- | --- | --- | --- | --- | --- | --- | --- | --- | --- | --- | --- | --- | --- | --- | --- | --- | --- | --- | --- | --- | --- | --- | --- | --- | --- | --- | --- | --- | --- | --- | --- | --- | --- | --- | --- | --- | --- | --- | --- | --- | --- | --- | --- | --- | --- | --- | --- | --- | --- | --- | --- | --- | --- | --- | --- | --- | --- | --- | --- | --- | --- | --- | --- | --- | --- | --- | --- | --- | --- | --- | --- | --- | --- | --- | --- | --- | --- | --- | --- | --- | --- | --- | --- | --- | --- | --- | --- | --- | --- | --- | --- | --- | --- | --- | --- | --- | --- | --- | --- | --- | --- | --- | --- | --- | --- | --- | --- | --- | --- | --- | --- | --- | --- | --- | --- | --- | --- | --- | --- | --- | --- | --- | --- | --- | --- | --- | --- | --- | --- | --- | --- | --- | --- | --- | --- | --- | --- | --- | --- | --- | --- | --- | --- | --- | --- | --- | --- | --- | --- | --- | --- | --- | --- | --- | --- | --- | --- | --- | --- | --- | --- | --- | --- | --- | --- | --- | --- | --- | --- | --- | --- | --- | --- | --- | --- | --- | --- | --- | --- | --- | --- | --- | --- | --- | --- | --- | --- | --- | --- | --- | --- | --- | --- | --- | --- | --- | --- | --- | --- | --- | --- | --- | --- | --- | --- | --- | --- | --- | --- | --- | --- | --- | --- | --- | --- | --- | --- | --- | --- | --- | --- | --- | --- | --- | --- | --- | --- | --- | --- | --- | --- | --- | --- | --- | --- | --- | --- | --- | --- | --- | --- | --- | --- | --- | --- | --- | --- | --- | --- | --- | --- | --- | --- | --- | --- | --- | --- | --- | --- | --- | --- | --- | --- | --- | --- | --- | --- | --- | --- | --- | --- | --- | --- | --- | --- | --- | --- | --- | --- | --- | --- | --- | --- | --- | --- | --- | --- | --- | --- | --- | --- | --- | --- | --- | --- | --- | --- | --- | --- | --- | --- | --- | --- | --- | --- | --- | --- | --- | --- | --- | --- | --- | --- | --- | --- | --- | --- | --- | --- | --- | --- | --- | --- | --- | --- | --- | --- | --- | --- | --- | --- | --- | --- | --- | --- | --- | --- | --- | --- | --- | --- | --- | --- | --- | --- | --- | --- | --- | --- | --- | --- | --- | --- | --- | --- | --- | --- | --- | --- | --- | --- | --- | --- | --- | --- | --- | --- | --- | --- | --- | --- | --- | --- | --- | --- | --- | --- | --- | --- | --- | --- | --- | --- | --- | --- | --- | --- | --- | --- | --- | --- | --- | --- | --- | --- | --- | --- | --- | --- | --- | --- | --- | --- | --- | --- | --- | --- | --- | --- | --- | --- | --- | --- | --- | --- | --- | --- | --- | --- | --- | --- | --- | --- | --- | --- | --- | --- | --- | --- | --- | --- | --- | --- | --- | --- | --- | --- | --- | --- | --- | --- | --- | --- | --- | --- | --- | --- | --- | --- | --- | --- | --- | --- | --- | --- | --- | --- | --- | --- | --- | --- | --- | --- | --- | --- | --- | --- | --- | --- | --- | --- | --- | --- | --- | --- | --- | --- | --- | --- | --- | --- | --- | --- | --- | --- | --- | --- | --- | --- | --- | --- | --- | --- | --- | --- | --- | --- | --- | --- | --- | --- | --- | --- | --- | --- | --- | --- | --- | --- | --- | --- | --- | --- | --- | --- | --- | --- | --- | --- | --- | --- | --- | --- | --- | --- | --- | --- | --- | --- | --- | --- | --- | --- | --- | --- | --- | --- | --- | --- | --- | --- | --- | --- | --- | --- | --- | --- | --- | --- | --- | --- | --- | --- | --- | --- | --- | --- | --- | --- | --- | --- | --- | --- | --- | --- | --- | --- | --- | --- | --- | --- | --- | --- | --- | --- | --- | --- | --- | --- | --- | --- | --- | --- | --- | --- | --- | --- | --- | --- | --- | --- | --- | --- | --- | --- | --- | --- | --- | --- | --- | --- | --- | --- | --- | --- | --- | --- | --- | --- | --- | --- | --- | --- | --- | --- | --- | --- | --- | --- | --- | --- | --- | --- | --- | --- | --- | --- | --- | --- | --- | --- | --- | --- | --- | --- | --- | --- | --- | --- | --- | --- | --- | --- | --- | --- | --- | --- | --- | --- | --- | --- | --- | --- | --- | --- | --- | --- | --- | --- | --- | --- | --- | --- | --- | --- | --- | --- | --- | --- | --- | --- | --- | --- | --- | --- | --- | --- | --- | --- | --- | --- | --- | --- | --- | --- | --- | --- | --- | --- | --- | --- | --- | --- | --- | --- | --- | --- | --- | --- | --- | --- | --- | --- | --- | --- | --- | --- | --- | --- | --- | --- | --- | --- | --- | --- | --- | --- | --- | --- | --- | --- | --- | --- | --- | --- | --- | --- | --- | --- | --- | --- | --- | --- | --- | --- | --- | --- | --- | --- | --- | --- | --- | --- | --- | --- | --- | --- | --- | --- | --- | --- | --- | --- | --- | --- | --- | --- | --- | --- | --- | --- | --- | --- | --- | --- | --- | --- | --- | --- | --- | --- | --- | --- | --- | --- | --- | --- | --- | --- | --- | --- | --- | --- | --- | --- | --- | --- | --- | --- | --- | --- | --- | --- | --- | --- | --- | --- | --- | --- | --- | --- | --- | --- | --- | --- | --- | --- | --- | --- | --- | --- | --- | --- | --- | --- | --- | --- | --- | --- | --- | --- | --- | --- | --- | --- | --- | --- | --- | --- | --- | --- | --- | --- | --- | --- | --- | --- | --- | --- | --- | --- | --- | --- | --- | --- | --- | --- | --- | --- | --- | --- | --- | --- | --- | --- | --- | --- | --- | --- | --- | --- | --- | --- | --- | --- | --- | --- | --- | --- | --- | --- | --- | --- | --- | --- | --- | --- | --- | --- | --- | --- | --- | --- | --- | --- | --- | --- | --- | --- | --- | --- | --- | --- | --- | --- | --- | --- | --- | --- | --- | --- | --- | --- | --- | --- | --- | --- | --- | --- | --- | --- | --- | --- | --- | --- | --- | --- | --- | --- | --- | --- | --- | --- | --- | --- | --- | --- | --- | --- | --- | --- | --- | --- | --- | --- | --- | --- | --- | --- | --- | --- | --- | --- | --- | --- | --- | --- | --- | --- | --- | --- | --- | --- | --- | --- | --- | --- | --- | --- | --- | --- | --- | --- | --- | --- | --- | --- | --- | --- | --- | --- | --- | --- | --- | --- | --- | --- | --- | --- | --- | --- | --- | --- | --- | --- | --- | --- | --- | --- | --- | --- | --- | --- | --- | --- | --- | --- | --- | --- | --- | --- | --- | --- | --- | --- | --- | --- | --- | --- | --- | --- | --- | --- | --- | --- | --- | --- | --- | --- | --- | --- | --- | --- | --- | --- | --- | --- | --- | --- | --- | --- | --- | --- | --- | --- | --- | --- | --- | --- | --- | --- | --- | --- | --- | --- | --- | --- | --- | --- | --- | --- | --- | --- | --- | --- | --- | --- | --- | --- | --- | --- | --- | --- | --- | --- | --- | --- | --- | --- | --- | --- | --- | --- | --- | --- | --- | --- | --- | --- | --- | --- | --- | --- | --- | --- | --- | --- | --- | --- | --- | --- | --- | --- | --- | --- | --- | --- | --- | --- | --- | --- | --- | --- | --- | --- | --- | --- | --- | --- | --- | --- | --- | --- | --- | --- | --- | --- | --- | --- | --- | --- | --- | --- | --- | --- | --- | --- | --- | --- | --- | --- | --- | --- | --- | --- | --- | --- | --- | --- | --- | --- | --- | --- | --- | --- | --- | --- | --- | --- | --- | --- | --- | --- | --- | --- | --- | --- | --- | --- | --- | --- | --- | --- | --- | --- | --- | --- | --- | --- | --- | --- | --- | --- | --- | --- | --- | --- | --- | --- | --- | --- | --- | --- | --- | --- | --- | --- | --- | --- | --- | --- | --- | --- | --- | --- | --- | --- | --- | --- | --- | --- | --- | --- | --- | --- | --- | --- | --- | --- | --- | --- | --- | --- | --- | --- | --- | --- | --- | --- | --- | --- | --- | --- | --- | --- | --- | --- | --- | --- | --- | --- | --- | --- | --- | --- | --- | --- | --- | --- | --- | --- | --- | --- | --- | --- | --- | --- | --- | --- | --- | --- | --- | --- | --- | --- | --- | --- | --- | --- | --- | --- | --- | --- | --- | --- | --- | --- | --- | --- | --- | --- | --- | --- | --- | --- | --- | --- | --- | --- | --- | --- | --- | --- | --- | --- | --- | --- | --- | --- | --- | --- | --- | --- | --- | --- | --- | --- | --- | --- | --- | --- | --- | --- | --- | --- | --- | --- | --- | --- | --- | --- | --- | --- | --- | --- | --- | --- | --- | --- | --- | --- | --- | --- | --- | --- | --- | --- | --- | --- | --- | --- | --- | --- | --- | --- | --- | --- | --- | --- | --- | --- | --- | --- | --- | --- | --- | --- | --- | --- | --- | --- | --- | --- | --- | --- | --- | --- | --- | --- | --- | --- | --- | --- | --- | --- | --- | --- | --- | --- | --- | --- | --- | --- | --- | --- | --- | --- | --- | --- | --- | --- | --- | --- | --- | --- | --- | --- | --- | --- | --- | --- | --- | --- | --- | --- | --- | --- | --- | --- | --- | --- | --- | --- | --- | --- | --- | --- | --- | --- | --- | --- | --- | --- | --- | --- | --- | --- | --- | --- | --- | --- | --- | --- | --- | --- | --- | --- | --- | --- | --- | --- | --- | --- | --- | --- | --- | --- | --- | --- | --- | --- | --- | --- | --- | --- | --- | --- | --- | --- | --- | --- | --- | --- | --- | --- | --- | --- | --- | --- | --- | --- | --- | --- | --- | --- | --- | --- | --- | --- | --- | --- | --- | --- | --- | --- | --- | --- | --- | --- | --- | --- | --- | --- | --- | --- | --- | --- | --- | --- | --- | --- | --- | --- | --- | --- | --- | --- | --- | --- | --- | --- | --- | --- | --- | --- | --- | --- | --- | --- | --- | --- | --- | --- | --- | --- | --- | --- | --- | --- | --- | --- | --- | --- | --- | --- | --- | --- | --- | --- | --- | --- | --- | --- | --- | --- | --- | --- | --- | --- | --- | --- | --- | --- | --- | --- | --- | --- | --- | --- | --- | --- | --- | --- | --- | --- | --- | --- | --- | --- | --- | --- | --- | --- | --- | --- | --- | --- | --- | --- | --- | --- | --- | --- | --- | --- | --- | --- | --- | --- | --- | --- | --- | --- | --- | --- | --- | --- | --- | --- | --- | --- | --- | --- | --- | --- | --- | --- | --- | --- | --- | --- | --- | --- | --- | --- | --- | --- | --- | --- | --- | --- | --- | --- | --- | --- | --- | --- | --- | --- | --- | --- | --- | --- | --- | --- | --- | --- | --- | --- | --- | --- | --- | --- | --- | --- | --- | --- | --- | --- | --- | --- | --- | --- | --- | --- | --- | --- | --- | --- | --- | --- | --- | --- | --- | --- | --- | --- | --- | --- | --- | --- | --- | --- | --- | --- | --- | --- | --- | --- | --- | --- | --- | --- | --- | --- | --- | --- | --- | --- | --- | --- | --- | --- | --- | --- | --- | --- | --- | --- | --- | --- | --- | --- | --- | --- | --- | --- | --- | --- | --- | --- | --- | --- | --- | --- | --- | --- | --- | --- | --- | --- | --- | --- | --- | --- | --- | --- | --- | --- | --- | --- | --- | --- | --- | --- | --- | --- | --- | --- | --- | --- | --- | --- | --- | --- | --- | --- | --- | --- | --- | --- | --- | --- | --- | --- | --- | --- | --- | --- | --- | --- | --- | --- | --- | --- | --- | --- | --- | --- | --- | --- | --- | --- | --- | --- | --- | --- | --- | --- | --- | --- | --- | --- | --- | --- | --- | --- | --- | --- | --- | --- | --- | --- | --- | --- | --- | --- | --- | --- | --- | --- | --- | --- | --- | --- | --- | --- | --- | --- | --- | --- | --- | --- | --- | --- | --- | --- | --- | --- | --- | --- | --- | --- | --- | --- | --- | --- | --- | --- | --- | --- | --- | --- | --- | --- | --- | --- | --- | --- | --- | --- | --- | --- | --- | --- | --- | --- | --- | --- | --- | --- | --- | --- | --- | --- | --- | --- | --- | --- | --- | --- | --- | --- | --- | --- | --- | --- | --- | --- | --- | --- | --- | --- | --- | --- | --- | --- | --- | --- | --- | --- | --- | --- | --- | --- | --- | --- | --- | --- | --- | --- | --- | --- | --- | --- | --- | --- | --- | --- | --- | --- | --- | --- | --- | --- | --- | --- | --- | --- | --- | --- | --- | --- | --- | --- | --- | --- | --- | --- | --- | --- | --- | --- | --- | --- | --- | --- | --- | --- | --- | --- | --- | --- | --- | --- | --- | --- | --- | --- | --- | --- | --- | --- | --- | --- | --- | --- | --- | --- | --- | --- | --- | --- | --- | --- | --- | --- | --- | --- | --- | --- | --- | --- | --- | --- | --- | --- | --- | --- | --- | --- | --- | --- | --- | --- | --- | --- | --- | --- | --- | --- | --- | --- | --- | --- | --- | --- | --- | --- | --- | --- | --- | --- | --- | --- | --- | --- | --- | --- | --- | --- | --- | --- | --- | --- | --- | --- | --- | --- | --- | --- | --- | --- | --- | --- | --- | --- | --- | --- | --- | --- | --- | --- | --- | --- | --- | --- | --- | --- | --- | --- | --- | --- | --- | --- | --- | --- | --- | --- | --- | --- | --- | --- | --- | --- | --- | --- | --- | --- | --- | --- | --- | --- | --- | --- | --- | --- | --- | --- | --- | --- | --- | --- | --- | --- | --- | --- | --- | --- | --- | --- | --- | --- | --- | --- | --- | --- | --- | --- | --- | --- | --- | --- | --- | --- | --- | --- | --- | --- | --- | --- | --- | --- | --- | --- | --- | --- | --- | --- | --- | --- | --- | --- | --- | --- | --- | --- | --- | --- | --- | --- | --- | --- | --- | --- | --- | --- | --- | --- | --- | --- | --- | --- | --- | --- | --- | --- | --- | --- | --- | --- | --- | --- | --- | --- | --- | --- | --- | --- | --- | --- | --- | --- | --- | --- | --- | --- | --- | --- | --- | --- | --- | --- | --- | --- | --- | --- | --- | --- | --- | --- | --- | --- | --- | --- | --- | --- | --- | --- | --- | --- | --- | --- | --- | --- | --- | --- | --- | --- | --- | --- | --- | --- | --- | --- | --- | --- | --- | --- | --- | --- | --- | --- | --- | --- | --- | --- | --- | --- | --- | --- | --- | --- | --- | --- | --- | --- | --- | --- | --- | --- | --- | --- | --- | --- | --- | --- | --- | --- | --- | --- | --- | --- | --- | --- | --- | --- | --- | --- | --- | --- | --- | --- | --- | --- | --- | --- | --- | --- | --- | --- | --- | --- | --- | --- | --- | --- | --- | --- | --- | --- | --- | --- | --- | --- | --- | --- | --- | --- | --- | --- | --- | --- | --- | --- | --- | --- | --- | --- | --- | --- | --- | --- | --- | --- | --- | --- | --- | --- | --- | --- | --- | --- | --- | --- | --- | --- | --- | --- | --- | --- | --- | --- | --- | --- | --- | --- | --- | --- | --- | --- | --- | --- | --- | --- | --- | --- | --- | --- | --- | --- | --- | --- | --- | --- | --- | --- | --- | --- | --- | --- | --- | --- | --- | --- | --- | --- | --- | --- | --- | --- | --- | --- | --- | --- | --- | --- | --- | --- | --- | --- | --- | --- | --- | --- | --- | --- | --- | --- | --- | --- | --- | --- | --- | --- | --- | --- | --- | --- | --- | --- | --- | --- | --- | --- | --- | --- | --- | --- | --- | --- | --- | --- | --- | --- | --- | --- | --- | --- | --- | --- | --- | --- | --- | --- | --- | --- | --- | --- | --- | --- | --- | --- | --- | --- | --- | --- | --- | --- | --- | --- | --- | --- | --- | --- | --- | --- | --- | --- | --- | --- | --- | --- | --- | --- | --- | --- | --- | --- | --- | --- | --- | --- | --- | --- | --- | --- | --- | --- | --- | --- | --- | --- | --- | --- | --- | --- | --- | --- | --- | --- | --- | --- | --- | --- | --- | --- | --- | --- | --- | --- | --- | --- | --- | --- | --- | --- | --- | --- | --- | --- | --- | --- | --- | --- | --- | --- | --- | --- | --- | --- | --- | --- | --- | --- | --- | --- | --- | --- | --- | --- | --- | --- | --- | --- | --- | --- | --- | --- | --- | --- | --- | --- | --- | --- | --- | --- | --- | --- | --- | --- | --- | --- | --- | --- | --- | --- | --- | --- | --- | --- | --- | --- | --- | --- | --- | --- | --- | --- | --- | --- | --- | --- | --- | --- | --- | --- | --- | --- | --- | --- | --- | --- | --- | --- | --- | --- | --- | --- | --- | --- | --- | --- | --- | --- | --- | --- | --- | --- | --- | --- | --- | --- | --- | --- | --- | --- | --- | --- | --- | --- | --- | --- | --- | --- | --- | --- | --- | --- | --- | --- | --- | --- | --- | --- | --- | --- | --- | --- | --- | --- | --- | --- | --- | --- | --- | --- | --- | --- | --- | --- | --- | --- | --- | --- | --- | --- | --- | --- | --- | --- | --- | --- | --- | --- | --- | --- | --- | --- | --- | --- | --- | --- | --- | --- | --- | --- | --- | --- | --- | --- | --- | --- | --- | --- | --- | --- | --- | --- | --- | --- | --- | --- | --- | --- | --- | --- | --- | --- | --- | --- | --- | --- | --- | --- | --- | --- | --- | --- | --- | --- | --- | --- | --- | --- | --- | --- | --- | --- | --- | --- | --- | --- | --- | --- | --- | --- | --- | --- | --- | --- | --- | --- | --- | --- | --- | --- | --- | --- | --- | --- | --- | --- | --- | --- | --- | --- | --- | --- | --- | --- | --- | --- | --- | --- | --- | --- | --- | --- | --- | --- | --- | --- | --- | --- | --- | --- | --- | --- | --- | --- | --- | --- | --- | --- | --- | --- | --- | --- | --- | --- | --- | --- | --- | --- | --- | --- | --- | --- | --- | --- | --- | --- | --- | --- | --- | --- | --- | --- | --- | --- | --- | --- | --- | --- | --- | --- | --- | --- | --- | --- | --- | --- | --- | --- | --- | --- | --- | --- | --- | --- | --- | --- | --- | --- | --- | --- | --- | --- | --- | --- | --- | --- | --- | --- | --- | --- | --- | --- | --- | --- | --- | --- | --- | --- | --- | --- | --- | --- | --- | --- | --- | --- | --- | --- | --- | --- | --- | --- | --- | --- | --- | --- | --- | --- | --- | --- | --- | --- | --- | --- | --- | --- | --- | --- | --- | --- | --- | --- | --- | --- | --- | --- | --- | --- | --- | --- | --- | --- | --- | --- | --- | --- | --- | --- | --- | --- | --- | --- | --- | --- | --- | --- | --- | --- | --- | --- | --- | --- | --- | --- | --- | --- | --- | --- | --- | --- | --- | --- | --- | --- | --- | --- | --- | --- | --- | --- | --- | --- | --- | --- | --- | --- | --- | --- | --- | --- | --- | --- | --- | --- | --- | --- | --- | --- | --- | --- | --- | --- | --- | --- | --- | --- | --- | --- | --- | --- | --- | --- | --- | --- | --- | --- | --- | --- | --- | --- | --- | --- | --- | --- | --- | --- | --- | --- | --- | --- | --- | --- | --- | --- | --- | --- | --- | --- | --- | --- | --- | --- | --- | --- | --- | --- | --- | --- | --- | --- | --- | --- | --- | --- | --- | --- | --- | --- | --- | --- | --- | --- | --- | --- | --- | --- | --- | --- | --- | --- | --- | --- | --- | --- | --- | --- | --- | --- | --- | --- | --- | --- | --- | --- | --- | --- | --- | --- | --- | --- | --- | --- | --- | --- | --- | --- | --- | --- | --- | --- | --- | --- | --- | --- | --- | --- | --- | --- | --- | --- | --- | --- | --- | --- | --- | --- | --- | --- | --- | --- | --- | --- | --- | --- | --- | --- | --- | --- | --- | --- | --- | --- | --- | --- | --- | --- | --- | --- | --- | --- | --- | --- | --- | --- | --- | --- | --- | --- | --- | --- | --- | --- | --- | --- | --- | --- | --- | --- | --- | --- | --- | --- | --- | --- | --- | --- | --- | --- | --- | --- | --- | --- | --- | --- | --- | --- | --- | --- | --- | --- | --- | --- | --- | --- | --- | --- | --- | --- | --- | --- | --- | --- | --- | --- | --- | --- | --- | --- | --- | --- | --- | --- | --- | --- | --- | --- | --- | --- | --- | --- | --- | --- | --- | --- | --- | --- | --- | --- | --- | --- | --- | --- | --- | --- | --- | --- | --- | --- | --- | --- | --- | --- | --- | --- | --- | --- | --- | --- | --- | --- | --- | --- | --- | --- | --- | --- | --- | --- | --- | --- | --- | --- | --- | --- | --- | --- | --- | --- | --- | --- | --- | --- | --- | --- | --- | --- | --- | --- | --- | --- | --- | --- | --- | --- | --- | --- | --- | --- | --- | --- | --- | --- | --- | --- | --- | --- | --- | --- | --- | --- | --- | --- | --- | --- | --- | --- | --- | --- | --- | --- | --- | --- | --- | --- | --- | --- | --- | --- | --- | --- | --- | --- | --- | --- | --- | --- | --- | --- | --- | --- | --- | --- | --- | --- | --- | --- | --- | --- | --- | --- | --- | --- | --- | --- | --- | --- | --- | --- | --- | --- | --- | --- | --- | --- | --- | --- | --- | --- | --- | --- | --- | --- | --- | --- | --- | --- | --- | --- | --- | --- | --- | --- | --- | --- | --- | --- | --- | --- | --- | --- | --- | --- | --- | --- | --- | --- | --- | --- | --- | --- | --- | --- | --- | --- | --- | --- | --- | --- | --- | --- | --- | --- | --- | --- | --- | --- | --- | --- | --- | --- | --- | --- | --- | --- | --- | --- | --- | --- | --- | --- | --- | --- | --- | --- | --- | --- | --- | --- | --- | --- | --- | --- | --- | --- | --- | --- | --- | --- | --- | --- | --- | --- | --- | --- | --- | --- | --- | --- | --- | --- | --- | --- | --- | --- | --- | --- | --- | --- | --- | --- | --- | --- | --- | --- | --- | --- | --- | --- | --- | --- | --- | --- | --- | --- | --- | --- | --- | --- | --- | --- | --- | --- | --- | --- | --- | --- | --- | --- | --- | --- | --- | --- | --- | --- | --- | --- | --- | --- | --- | --- | --- | --- | --- | --- | --- | --- | --- | --- | --- | --- | --- | --- | --- | --- | --- | --- | --- | --- | --- | --- | --- | --- | --- | --- | --- | --- | --- | --- | --- | --- | --- | --- | --- | --- | --- | --- | --- | --- | --- | --- | --- | --- | --- | --- | --- | --- | --- | --- | --- | --- | --- | --- | --- | --- | --- | --- | --- | --- | --- | --- | --- | --- | --- | --- | --- | --- | --- | --- | --- | --- | --- | --- | --- | --- | --- | --- | --- | --- | --- | --- | --- | --- | --- | --- | --- | --- | --- | --- | --- | --- | --- | --- | --- | --- | --- | --- | --- | --- | --- | --- | --- | --- | --- | --- | --- | --- | --- | --- | --- | --- | --- | --- | --- | --- | --- | --- | --- | --- | --- | --- | --- | --- | --- | --- | --- | --- | --- | --- | --- | --- | --- | --- | --- | --- | --- | --- | --- | --- | --- | --- | --- | --- | --- | --- | --- | --- | --- | --- | --- | --- | --- | --- | --- | --- | --- | --- | --- | --- | --- | --- | --- | --- | --- | --- | --- | --- | --- | --- | --- | --- | --- | --- | --- | --- | --- | --- | --- | --- | --- | --- | --- | --- | --- | --- | --- | --- | --- | --- | --- | --- | --- | --- | --- | --- | --- | --- | --- | --- | --- | --- | --- | --- | --- | --- | --- | --- | --- | --- | --- | --- | --- | --- | --- | --- | --- | --- | --- | --- | --- | --- | --- | --- | --- | --- | --- | --- | --- | --- | --- | --- | --- | --- | --- | --- | --- | --- | --- | --- | --- | --- | --- | --- | --- | --- | --- | --- | --- | --- | --- | --- | --- | --- | --- | --- | --- | --- | --- | --- | --- | --- | --- | --- | --- | --- | --- | --- | --- | --- | --- | --- | --- | --- | --- | --- | --- | --- | --- | --- | --- | --- | --- | --- | --- | --- | --- | --- | --- | --- | --- | --- | --- | --- | --- | --- | --- | --- | --- | --- | --- | --- | --- | --- | --- | --- | --- | --- | --- | --- | --- | --- | --- | --- | --- | --- | --- | --- | --- | --- | --- | --- | --- | --- | --- | --- | --- | --- | --- | --- | --- | --- | --- | --- | --- | --- | --- | --- | --- | --- | --- | --- | --- | --- | --- | --- | --- | --- | --- | --- | --- | --- | --- | --- | --- | --- | --- | --- | --- | --- | --- | --- | --- | --- | --- | --- | --- | --- | --- | --- | --- | --- | --- | --- | --- | --- | --- | --- | --- | --- | --- | --- | --- | --- | --- | --- | --- | --- | --- | --- | --- | --- | --- | --- | --- | --- | --- | --- | --- | --- | --- | --- | --- | --- | --- | --- | --- | --- | --- | --- | --- | --- | --- | --- | --- | --- | --- | --- | --- | --- | --- | --- | --- | --- | --- | --- | --- | --- | --- | --- | --- | --- | --- | --- | --- | --- | --- | --- | --- | --- | --- | --- | --- | --- | --- | --- | --- | --- | --- | --- | --- | --- | --- | --- | --- | --- | --- | --- | --- | --- | --- | --- | --- | --- | --- | --- | --- | --- | --- | --- | --- | --- | --- | --- | --- | --- | --- | --- | --- | --- | --- | --- | --- | --- | --- | --- | --- | --- | --- | --- | --- | --- | --- | --- | --- | --- | --- | --- | --- | --- | --- | --- | --- | --- | --- | --- | --- | --- | --- | --- | --- | --- | --- | --- | --- | --- | --- | --- | --- | --- | --- | --- | --- | --- | --- | --- | --- | --- | --- | --- | --- | --- | --- | --- | --- | --- | --- | --- | --- | --- | --- | --- | --- | --- | --- | --- | --- | --- | --- | --- | --- | --- | --- | --- | --- | --- | --- | --- | --- | --- | --- | --- | --- | --- | --- | --- | --- | --- | --- | --- | --- | --- | --- | --- | --- | --- | --- | --- | --- | --- | --- | --- | --- | --- | --- | --- | --- | --- | --- | --- | --- | --- | --- | --- | --- | --- | --- | --- | --- | --- | --- | --- | --- | --- | --- | --- | --- | --- | --- | --- | --- | --- | --- | --- | --- | --- | --- | --- | --- | --- | --- | --- | --- | --- | --- | --- | --- | --- | --- | --- | --- | --- | --- | --- | --- | --- | --- | --- | --- | --- | --- | --- | --- | --- | --- | --- | --- | --- | --- | --- | --- | --- | --- | --- | --- | --- | --- | --- | --- | --- | --- | --- | --- | --- | --- | --- | --- | --- | --- | --- | --- | --- | --- | --- | --- | --- | --- | --- | --- | --- | --- | --- | --- | --- | --- | --- | --- | --- | --- | --- | --- | --- | --- | --- | --- | --- | --- | --- | --- | --- | --- | --- | --- | --- | --- | --- | --- | --- | --- | --- | --- | --- | --- | --- | --- | --- | --- | --- | --- | --- | --- | --- | --- | --- | --- | --- | --- | --- | --- | --- | --- | --- | --- | --- | --- | --- | --- | --- | --- | --- | --- | --- | --- | --- | --- | --- | --- | --- | --- | --- | --- | --- | --- | --- | --- | --- | --- | --- | --- | --- | --- | --- | --- | --- | --- | --- | --- | --- | --- | --- | --- | --- | --- | --- | --- | --- | --- | --- | --- | --- | --- | --- | --- | --- | --- | --- | --- | --- | --- | --- | --- | --- | --- | --- | --- | --- | --- | --- | --- | --- | --- | --- | --- | --- | --- | --- | --- | --- | --- | --- | --- | --- | --- | --- | --- | --- | --- | --- | --- | --- | --- | --- | --- | --- | --- | --- | --- | --- | --- | --- | --- | --- | --- | --- | --- | --- | --- | --- | --- | --- | --- | --- | --- | --- | --- | --- | --- | --- | --- | --- | --- | --- | --- | --- | --- | --- | --- | --- | --- | --- | --- | --- | --- | --- | --- | --- | --- | --- | --- | --- | --- | --- | --- | --- | --- | --- | --- | --- | --- | --- | --- | --- | --- | --- | --- | --- | --- | --- | --- | --- | --- | --- | --- | --- | --- | --- | --- | --- | --- | --- | --- | --- | --- | --- | --- | --- | --- | --- | --- | --- | --- | --- | --- | --- | --- | --- | --- | --- | --- | --- | --- | --- | --- | --- | --- | --- | --- | --- | --- | --- | --- | --- | --- | --- | --- | --- | --- | --- | --- | --- | --- | --- | --- | --- | --- | --- | --- | --- | --- | --- | --- | --- | --- | --- | --- | --- | --- | --- | --- | --- | --- | --- | --- | --- | --- | --- | --- | --- | --- | --- | --- | --- | --- | --- | --- | --- | --- | --- | --- | --- | --- | --- | --- | --- | --- | --- | --- | --- | --- | --- | --- | --- | --- | --- | --- | --- | --- | --- | --- | --- | --- | --- | --- | --- | --- | --- | --- | --- | --- | --- | --- | --- | --- | --- | --- | --- | --- | --- | --- | --- | --- | --- | --- | --- | --- | --- | --- | --- | --- | --- | --- | --- | --- | --- | --- | --- | --- | --- | --- | --- | --- | --- | --- | --- | --- | --- | --- | --- | --- | --- | --- | --- | --- | --- | --- | --- | --- | --- | --- | --- | --- | --- | --- | --- | --- | --- | --- | --- | --- | --- | --- | --- | --- | --- | --- | --- | --- | --- | --- | --- | --- | --- | --- | --- | --- | --- | --- | --- | --- | --- | --- | --- | --- | --- | --- | --- | --- | --- | --- | --- | --- | --- | --- | --- | --- | --- | --- | --- | --- | --- | --- | --- | --- | --- | --- | --- | --- | --- | --- | --- | --- | --- | --- | --- | --- | --- | --- | --- | --- | --- | --- | --- | --- | --- | --- | --- | --- | --- | --- | --- | --- | --- | --- | --- | --- | --- | --- | --- | --- | --- | --- | --- | --- | --- | --- | --- | --- | --- | --- | --- | --- | --- | --- | --- | --- | --- | --- | --- | --- | --- | --- | --- | --- | --- | --- | --- | --- | --- | --- | --- | --- | --- | --- | --- | --- | --- | --- | --- | --- | --- | --- | --- | --- | --- | --- | --- | --- | --- | --- | --- | --- | --- | --- | --- | --- | --- | --- | --- | --- | --- | --- | --- | --- | --- | --- | --- | --- | --- | --- | --- | --- | --- | --- | --- | --- | --- | --- | --- | --- | --- | --- | --- | --- | --- | --- | --- | --- | --- | --- | --- | --- | --- | --- | --- | --- | --- | --- | --- | --- | --- | --- | --- | --- | --- | --- | --- | --- | --- | --- | --- | --- | --- | --- | --- | --- | --- | --- | --- | --- | --- | --- | --- | --- | --- | --- | --- | --- | --- | --- | --- | --- | --- | --- | --- | --- | --- | --- | --- | --- | --- | --- | --- | --- | --- | --- | --- | --- | --- | --- | --- | --- | --- | --- | --- | --- | --- | --- | --- | --- | --- | --- | --- | --- | --- | --- | --- | --- | --- | --- | --- | --- | --- | --- | --- | --- | --- | --- | --- | --- | --- | --- | --- | --- | --- | --- | --- | --- | --- | --- | --- | --- | --- | --- | --- | --- | --- | --- | --- | --- | --- | --- | --- | --- | --- | --- | --- | --- | --- | --- | --- | --- | --- | --- | --- | --- | --- | --- | --- | --- | --- | --- | --- | --- | --- | --- | --- | --- | --- | --- | --- | --- | --- | --- | --- | --- | --- | --- | --- | --- | --- | --- | --- | --- | --- | --- | --- | --- | --- | --- | --- | --- | --- | --- | --- | --- | --- | --- | --- | --- | --- | --- | --- | --- | --- | --- | --- | --- | --- | --- | --- | --- | --- | --- | --- | --- | --- | --- | --- | --- | --- | --- | --- | --- | --- | --- | --- | --- | --- | --- | --- | --- | --- | --- | --- | --- | --- | --- | --- | --- | --- | --- | --- | --- | --- | --- | --- | --- | --- | --- | --- | --- | --- | --- | --- | --- | --- | --- | --- | --- | --- | --- | --- | --- | --- | --- | --- | --- | --- | --- | --- | --- | --- | --- | --- | --- | --- | --- | --- | --- | --- | --- | --- | --- | --- | --- | --- | --- | --- | --- | --- | --- | --- | --- | --- | --- | --- | --- | --- | --- | --- | --- | --- | --- | --- | --- | --- | --- | --- | --- | --- | --- | --- | --- | --- | --- | --- | --- | --- | --- | --- | --- | --- | --- | --- | --- | --- | --- | --- | --- | --- | --- | --- | --- | --- | --- | --- | --- | --- | --- | --- | --- | --- | --- | --- | --- | --- | --- | --- | --- | --- | --- | --- | --- | --- | --- | --- | --- | --- | --- | --- | --- | --- | --- | --- | --- | --- | --- | --- | --- | --- | --- | --- | --- | --- | --- | --- | --- | --- | --- | --- | --- | --- | --- | --- | --- | --- | --- | --- | --- | --- | --- | --- | --- | --- | --- | --- | --- | --- | --- | --- | --- | --- | --- | --- | --- | --- | --- | --- | --- | --- | --- | --- | --- | --- | --- | --- | --- | --- | --- | --- | --- | --- | --- | --- | --- | --- | --- | --- | --- | --- | --- | --- | --- | --- | --- | --- | --- | --- | --- | --- | --- | --- | --- | --- | --- | --- | --- | --- | --- | --- | --- | --- | --- | --- | --- | --- | --- | --- | --- | --- | --- | --- | --- | --- | --- | --- | --- | --- | --- | --- | --- | --- | --- | --- | --- | --- | --- | --- | --- | --- | --- | --- | --- | --- | --- | --- | --- | --- | --- | --- | --- | --- | --- | --- | --- | --- | --- | --- | --- | --- | --- | --- | --- | --- | --- | --- | --- | --- | --- | --- | --- | --- | --- | --- | --- | --- | --- | --- | --- | --- | --- | --- | --- | --- | --- | --- | --- | --- | --- | --- | --- | --- | --- | --- | --- | --- | --- | --- | --- | --- | --- | --- | --- | --- | --- | --- | --- | --- | --- | --- | --- | --- | --- | --- | --- | --- | --- | --- | --- | --- | --- | --- | --- | --- | --- | --- | --- | --- | --- | --- | --- | --- | --- | --- | --- | --- | --- | --- | --- | --- | --- | --- | --- | --- | --- | --- | --- | --- | --- | --- | --- | --- | --- | --- | --- | --- | --- | --- | --- | --- | --- | --- | --- | --- | --- | --- | --- | --- | --- | --- | --- | --- | --- | --- | --- | --- | --- | --- | --- | --- | --- | --- | --- | --- | --- | --- | --- | --- | --- | --- | --- | --- | --- | --- | --- | --- | --- | --- | --- | --- | --- | --- | --- | --- | --- | --- | --- | --- | --- | --- | --- | --- | --- | --- | --- | --- | --- | --- | --- | --- | --- | --- | --- | --- | --- | --- | --- | --- | --- | --- | --- | --- | --- | --- | --- | --- | --- | --- | --- | --- | --- | --- | --- | --- | --- | --- | --- | --- | --- | --- | --- | --- | --- | --- | --- | --- | --- | --- | --- | --- | --- | --- | --- | --- | --- | --- | --- | --- | --- | --- | --- | --- | --- | --- | --- | --- | --- | --- | --- | --- | --- | --- | --- | --- | --- | --- | --- | --- | --- | --- | --- | --- | --- | --- | --- | --- | --- | --- | --- | --- | --- | --- | --- | --- | --- | --- | --- | --- | --- | --- | --- | --- | --- | --- | --- | --- | --- | --- | --- | --- | --- | --- | --- | --- | --- | --- | --- | --- | --- | --- | --- | --- | --- | --- | --- | --- | --- | --- | --- | --- | --- | --- | --- | --- | --- | --- | --- | --- | --- | --- | --- | --- | --- | --- | --- | --- | --- | --- | --- | --- | --- | --- | --- | --- | --- | --- | --- | --- | --- | --- | --- | --- | --- | --- | --- | --- | --- | --- | --- | --- | --- | --- | --- | --- | --- | --- | --- | --- | --- | --- | --- | --- | --- | --- | --- | --- | --- | --- | --- | --- | --- | --- | --- | --- | --- | --- | --- | --- | --- | --- | --- | --- | --- | --- | --- | --- | --- | --- | --- | --- | --- | --- | --- | --- | --- | --- | --- | --- | --- | --- | --- | --- | --- | --- | --- | --- | --- | --- | --- | --- | --- | --- | --- | --- | --- | --- | --- | --- | --- | --- | --- | --- | --- | --- | --- | --- | --- | --- | --- | --- | --- | --- | --- | --- | --- | --- | --- | --- | --- | --- | --- | --- | --- | --- | --- | --- | --- | --- | --- | --- | --- | --- | --- | --- | --- | --- | --- | --- | --- | --- | --- | --- | --- | --- | --- | --- | --- | --- | --- | --- | --- | --- | --- | --- | --- | --- | --- | --- | --- | --- | --- | --- | --- | --- | --- | --- | --- | --- | --- | --- | --- | --- | --- | --- | --- | --- | --- | --- | --- | --- | --- | --- | --- | --- | --- | --- | --- | --- | --- | --- | --- | --- | --- | --- | --- | --- | --- | --- | --- | --- | --- | --- | --- | --- | --- | --- | --- | --- | --- | --- | --- | --- | --- | --- | --- | --- | --- | --- | --- | --- | --- | --- | --- | --- | --- | --- | --- | --- | --- | --- | --- | --- | --- | --- | --- | --- | --- | --- | --- | --- | --- | --- | --- | --- | --- | --- | --- | --- | --- | --- | --- | --- | --- | --- | --- | --- | --- | --- | --- | --- | --- | --- | --- | --- | --- | --- | --- | --- | --- | --- | --- | --- | --- | --- | --- | --- | --- | --- | --- | --- | --- | --- | --- | --- | --- | --- | --- | --- | --- | --- | --- | --- | --- | --- | --- | --- | --- | --- | --- | --- | --- | --- | --- | --- | --- | --- | --- | --- | --- | --- | --- | --- | --- | --- | --- | --- | --- | --- | --- | --- | --- | --- | --- | --- | --- | --- | --- | --- | --- | --- | --- | --- | --- | --- | --- | --- | --- | --- | --- | --- | --- | --- | --- | --- | --- | --- | --- | --- | --- | --- | --- | --- | --- | --- | --- | --- | --- | --- | --- | --- | --- | --- | --- | --- | --- | --- | --- | --- | --- | --- | --- | --- | --- | --- | --- | --- | --- | --- | --- | --- | --- | --- | --- | --- | --- | --- | --- | --- | --- | --- | --- | --- | --- | --- | --- | --- | --- | --- | --- | --- | --- | --- | --- | --- | --- | --- | --- | --- | --- | --- | --- | --- | --- | --- | --- | --- | --- | --- | --- | --- | --- | --- | --- | --- | --- | --- | --- | --- | --- | --- | --- | --- | --- | --- | --- | --- | --- | --- | --- | --- | --- | --- | --- | --- | --- | --- | --- | --- | --- | --- | --- | --- | --- | --- | --- | --- | --- | --- | --- | --- | --- | --- | --- | --- | --- | --- | --- | --- | --- | --- | --- | --- | --- | --- | --- | --- | --- | --- | --- | --- | --- | --- | --- | --- | --- | --- | --- | --- | --- | --- | --- | --- | --- | --- | --- | --- | --- | --- | --- | --- | --- | --- | --- | --- | --- | --- | --- | --- | --- | --- | --- | --- | --- | --- | --- | --- | --- | --- | --- | --- | --- | --- | --- | --- | --- | --- | --- | --- | --- | --- | --- | --- | --- | --- | --- | --- | --- | --- | --- | --- | --- | --- | --- | --- | --- | --- | --- | --- | --- | --- | --- | --- | --- | --- | --- | --- | --- | --- | --- | --- | --- | --- | --- | --- | --- | --- | --- | --- | --- | --- | --- | --- | --- | --- | --- | --- | --- | --- | --- | --- | --- | --- | --- | --- | --- | --- | --- | --- | --- | --- | --- | --- | --- | --- | --- | --- | --- | --- | --- | --- | --- | --- | --- | --- | --- | --- | --- | --- | --- | --- | --- | --- | --- | --- | --- | --- | --- | --- | --- | --- | --- | --- | --- | --- | --- | --- | --- | --- | --- | --- | --- | --- | --- | --- | --- | --- | --- | --- | --- | --- | --- | --- | --- | --- | --- | --- | --- | --- | --- | --- | --- | --- | --- | --- | --- | --- | --- | --- | --- | --- | --- | --- | --- | --- | --- | --- | --- | --- | --- | --- | --- | --- | --- | --- | --- | --- | --- | --- | --- | --- | --- | --- | --- | --- | --- | --- | --- | --- | --- | --- | --- | --- | --- | --- | --- | --- | --- | --- | --- | --- | --- | --- | --- | --- | --- | --- | --- | --- | --- | --- | --- | --- | --- | --- | --- | --- | --- | --- | --- | --- | --- | --- | --- | --- | --- | --- | --- | --- | --- | --- | --- | --- | --- | --- | --- | --- | --- | --- | --- | --- | --- | --- | --- | --- | --- | --- | --- | --- | --- | --- | --- | --- | --- | --- | --- | --- | --- | --- | --- | --- | --- | --- | --- | --- | --- | --- | --- | --- | --- | --- | --- | --- | --- | --- | --- | --- | --- | --- | --- | --- | --- | --- | --- | --- | --- | --- | --- | --- | --- | --- | --- | --- | --- | --- | --- | --- | --- | --- | --- | --- | --- | --- | --- | --- | --- | --- | --- | --- | --- | --- | --- | --- | --- | --- | --- | --- | --- | --- | --- | --- | --- | --- | --- | --- | --- | --- | --- | --- | --- | --- | --- | --- | --- | --- | --- | --- | --- | --- | --- | --- | --- | --- | --- | --- | --- | --- | --- | --- | --- | --- | --- | --- | --- | --- | --- | --- | --- | --- | --- | --- | --- | --- | --- | --- | --- | --- | --- | --- | --- | --- | --- | --- | --- | --- | --- | --- | --- | --- | --- | --- | --- | --- | --- | --- | --- | --- | --- | --- | --- | --- | --- | --- | --- | --- | --- | --- | --- | --- | --- | --- | --- | --- | --- | --- | --- | --- | --- | --- | --- | --- | --- | --- | --- | --- | --- | --- | --- | --- | --- | --- | --- | --- | --- | --- | --- | --- | --- | --- | --- | --- | --- | --- | --- | --- | --- | --- | --- | --- | --- | --- | --- | --- | --- | --- | --- | --- | --- | --- | --- | --- | --- | --- | --- | --- | --- | --- | --- | --- | --- | --- | --- | --- | --- | --- | --- | --- | --- | --- | --- | --- | --- | --- | --- | --- | --- | --- | --- | --- | --- | --- | --- | --- | --- | --- | --- | --- | --- | --- | --- | --- | --- | --- | --- | --- | --- | --- | --- | --- | --- | --- | --- | --- | --- | --- | --- | --- | --- | --- | --- | --- | --- | --- | --- | --- | --- | --- | --- | --- | --- | --- | --- | --- | --- | --- | --- | --- | --- | --- | --- | --- | --- | --- | --- | --- | --- | --- | --- | --- | --- | --- | --- | --- | --- | --- | --- | --- | --- | --- | --- | --- | --- | --- | --- | --- | --- | --- | --- | --- | --- | --- | --- | --- | --- | --- | --- | --- | --- | --- | --- | --- | --- | --- | --- | --- | --- | --- | --- | --- | --- | --- | --- | --- | --- | --- | --- | --- | --- | --- | --- | --- | --- | --- | --- | --- | --- | --- | --- | --- | --- | --- | --- | --- | --- | --- | --- | --- | --- | --- | --- | --- | --- | --- | --- | --- | --- | --- | --- | --- | --- | --- | --- | --- | --- | --- | --- | --- | --- | --- | --- | --- | --- | --- | --- | --- | --- | --- | --- | --- | --- | --- | --- | --- | --- | --- | --- | --- | --- | --- | --- | --- | --- | --- | --- | --- | --- | --- | --- | --- | --- | --- | --- | --- | --- | --- | --- | --- | --- | --- | --- | --- | --- | --- | --- | --- | --- | --- | --- | --- | --- | --- | --- | --- | --- | --- | --- | --- | --- | --- | --- | --- | --- | --- | --- | --- | --- | --- | --- | --- | --- | --- | --- | --- | --- | --- | --- | --- | --- | --- | --- | --- | --- | --- | --- | --- | --- | --- | --- | --- | --- | --- | --- | --- | --- | --- | --- | --- | --- | --- | --- | --- | --- | --- | --- | --- | --- | --- | --- | --- | --- | --- | --- | --- | --- | --- | --- | --- | --- | --- | --- | --- | --- | --- | --- | --- | --- | --- | --- | --- | --- | --- | --- | --- | --- | --- | --- | --- | --- | --- | --- | --- | --- | --- | --- | --- | --- | --- | --- | --- | --- | --- | --- | --- | --- | --- | --- | --- | --- | --- | --- | --- | --- | --- | --- | --- | --- | --- | --- | --- | --- | --- | --- | --- | --- | --- | --- | --- | --- | --- | --- | --- | --- | --- | --- | --- | --- | --- | --- | --- | --- | --- | --- | --- | --- | --- | --- | --- | --- | --- | --- | --- | --- | --- | --- | --- | --- | --- | --- | --- | --- | --- | --- | --- | --- | --- | --- | --- | --- | --- | --- | --- | --- | --- | --- | --- | --- | --- | --- | --- | --- | --- | --- | --- | --- | --- | --- | --- | --- | --- | --- | --- | --- | --- | --- | --- | --- | --- | --- | --- | --- | --- | --- | --- | --- | --- | --- | --- | --- | --- | --- | --- | --- | --- | --- | --- | --- | --- | --- | --- | --- | --- | --- | --- | --- | --- | --- | --- | --- | --- | --- | --- | --- | --- | --- | --- | --- | --- | --- | --- | --- | --- | --- | --- | --- | --- | --- | --- | --- | --- | --- | --- | --- | --- | --- | --- | --- | --- | --- | --- | --- | --- | --- | --- | --- | --- | --- | --- | --- | --- | --- | --- | --- | --- | --- | --- | --- | --- | --- | --- | --- | --- | --- | --- | --- | --- | --- | --- | --- | --- | --- | --- | --- | --- | --- | --- | --- | --- | --- | --- | --- | --- | --- | --- | --- | --- | --- | --- | --- | --- | --- | --- | --- | --- | --- | --- | --- | --- | --- | --- | --- | --- | --- | --- | --- | --- | --- | --- | --- | --- | --- | --- | --- | --- | --- | --- | --- | --- | --- | --- | --- | --- | --- | --- | --- | --- | --- | --- | --- | --- | --- | --- | --- | --- | --- | --- | --- | --- | --- | --- | --- | --- | --- | --- | --- | --- | --- | --- | --- | --- | --- | --- | --- | --- | --- | --- | --- | --- | --- | --- | --- | --- | --- | --- | --- | --- | --- | --- | --- | --- | --- | --- | --- | --- | --- | --- | --- | --- | --- | --- | --- | --- | --- | --- | --- | --- | --- | --- | --- | --- | --- | --- | --- | --- | --- | --- | --- | --- | --- | --- | --- | --- | --- | --- | --- | --- | --- | --- | --- | --- | --- | --- | --- | --- | --- | --- | --- | --- | --- | --- | --- | --- | --- | --- | --- | --- | --- | --- | --- | --- | --- | --- | --- | --- | --- | --- | --- | --- | --- | --- | --- | --- | --- | --- | --- | --- | --- | --- | --- | --- | --- | --- | --- | --- | --- | --- | --- | --- | --- | --- | --- | --- | --- | --- | --- | --- | --- | --- | --- | --- | --- | --- | --- | --- | --- | --- | --- | --- | --- | --- | --- | --- | --- | --- | --- | --- | --- | --- | --- | --- | --- | --- | --- | --- | --- | --- | --- | --- | --- | --- | --- | --- | --- | --- | --- | --- | --- | --- | --- | --- | --- | --- | --- | --- | --- | --- | --- | --- | --- | --- | --- | --- | --- | --- | --- | --- | --- | --- | --- | --- | --- | --- | --- | --- | --- | --- | --- | --- | --- | --- | --- | --- | --- | --- | --- | --- | --- | --- | --- | --- | --- | --- | --- | --- | --- | --- | --- | --- | --- | --- | --- | --- | --- | --- | --- | --- | --- | --- | --- | --- | --- | --- | --- | --- | --- | --- | --- | --- | --- | --- | --- | --- | --- | --- | --- | --- | --- | --- | --- | --- | --- | --- | --- | --- | --- | --- | --- | --- | --- | --- | --- | --- | --- | --- | --- | --- | --- | --- | --- | --- | --- | --- | --- | --- | --- | --- | --- | --- | --- | --- | --- | --- | --- | --- | --- | --- | --- | --- | --- | --- | --- | --- | --- | --- | --- | --- | --- | --- | --- | --- | --- | --- | --- | --- | --- | --- | --- | --- | --- | --- | --- | --- | --- | --- | --- | --- | --- | --- | --- | --- | --- | --- | --- | --- | --- | --- | --- | --- | --- | --- | --- | --- | --- | --- | --- | --- | --- | --- | --- | --- | --- | --- | --- | --- | --- | --- | --- | --- | --- | --- | --- | --- | --- | --- | --- | --- | --- | --- | --- | --- | --- | --- | --- | --- | --- | --- | --- | --- | --- | --- | --- | --- | --- | --- | --- | --- | --- | --- | --- | --- | --- | --- | --- | --- | --- | --- | --- | --- | --- | --- | --- | --- | --- | --- | --- | --- | --- | --- | --- | --- | --- | --- | --- | --- | --- | --- | --- | --- | --- | --- | --- | --- | --- | --- | --- | --- | --- | --- | --- | --- | --- | --- | --- | --- | --- | --- | --- | --- | --- | --- | --- | --- | --- | --- | --- | --- | --- | --- | --- | --- | --- | --- | --- | --- | --- | --- | --- | --- | --- | --- | --- | --- | --- | --- | --- | --- | --- | --- | --- | --- | --- | --- | --- | --- | --- | --- | --- | --- | --- | --- | --- | --- | --- | --- | --- | --- | --- | --- | --- | --- | --- | --- | --- | --- | --- | --- | --- | --- | --- | --- | --- | --- | --- | --- | --- | --- | --- | --- | --- | --- | --- | --- | --- | --- | --- | --- | --- | --- | --- | --- | --- | --- | --- | --- | --- | --- | --- | --- | --- | --- | --- | --- | --- | --- | --- | --- | --- | --- | --- | --- | --- | --- | --- | --- | --- | --- | --- | --- | --- | --- | --- | --- | --- | --- | --- | --- | --- | --- | --- | --- | --- | --- | --- | --- | --- | --- | --- | --- | --- | --- | --- | --- | --- | --- | --- | --- | --- | --- | --- | --- | --- | --- | --- | --- | --- | --- | --- | --- | --- | --- | --- | --- | --- | --- | --- | --- | --- | --- | --- | --- | --- | --- | --- | --- | --- | --- | --- | --- | --- | --- | --- | --- | --- | --- | --- | --- | --- | --- | --- | --- | --- | --- | --- | --- | --- | --- | --- | --- | --- | --- | --- | --- | --- | --- | --- | --- | --- | --- | --- | --- | --- | --- | --- | --- | --- | --- | --- | --- | --- | --- | --- | --- | --- | --- | --- | --- | --- | --- | --- | --- | --- | --- | --- | --- | --- | --- | --- | --- | --- | --- | --- | --- | --- | --- | --- | --- | --- | --- | --- | --- | --- | --- | --- | --- | --- | --- | --- | --- | --- | --- | --- | --- | --- | --- | --- | --- | --- | --- | --- | --- | --- | --- | --- | --- | --- | --- | --- | --- | --- | --- | --- | --- | --- | --- | --- | --- | --- | --- | --- | --- | --- | --- | --- | --- | --- | --- | --- | --- | --- | --- | --- | --- | --- | --- | --- | --- | --- | --- | --- | --- | --- | --- | --- | --- | --- | --- | --- | --- | --- | --- | --- | --- | --- | --- | --- | --- | --- | --- | --- | --- | --- | --- | --- | --- | --- | --- | --- | --- | --- | --- | --- | --- | --- | --- | --- | --- | --- | --- | --- | --- | --- | --- | --- | --- | --- | --- | --- | --- | --- | --- | --- | --- | --- | --- | --- | --- | --- | --- | --- | --- | --- | --- | --- | --- | --- | --- | --- | --- | --- | --- | --- | --- | --- | --- | --- | --- | --- | --- | --- | --- | --- | --- | --- | --- | --- | --- | --- | --- | --- | --- | --- | --- | --- | --- | --- | --- | --- | --- | --- | --- | --- | --- | --- | --- | --- | --- | --- | --- | --- | --- | --- | --- | --- | --- | --- | --- | --- | --- | --- | --- | --- | --- | --- | --- | --- | --- | --- | --- | --- | --- | --- | --- | --- | --- | --- | --- | --- | --- | --- | --- | --- | --- | --- | --- | --- | --- | --- | --- | --- | --- | --- | --- | --- | --- | --- | --- | --- | --- | --- | --- | --- | --- | --- | --- | --- | --- | --- | --- | --- | --- | --- | --- | --- | --- | --- | --- | --- | --- | --- | --- | --- | --- | --- | --- | --- | --- | --- | --- | --- | --- | --- | --- | --- | --- | --- | --- | --- | --- | --- | --- | --- | --- | --- | --- | --- | --- | --- | --- | --- | --- | --- | --- | --- | --- | --- | --- | --- | --- | --- | --- | --- | --- | --- | --- | --- | --- | --- | --- | --- | --- | --- | --- | --- | --- | --- | --- | --- | --- | --- | --- | --- | --- | --- | --- | --- | --- | --- | --- | --- | --- | --- | --- | --- | --- | --- | --- | --- | --- | --- | --- | --- | --- | --- | --- | --- | --- | --- | --- | --- | --- | --- | --- | --- | --- | --- | --- | --- | --- | --- | --- | --- | --- | --- | --- | --- | --- | --- | --- | --- | --- | --- | --- | --- | --- | --- | --- | --- | --- | --- | --- | --- | --- | --- | --- | --- | --- | --- | --- | --- | --- | --- | --- | --- | --- | --- | --- | --- | --- | --- | --- | --- | --- | --- | --- | --- | --- | --- | --- | --- | --- | --- | --- | --- | --- | --- | --- | --- | --- | --- | --- | --- | --- | --- | --- | --- | --- | --- | --- | --- | --- | --- | --- | --- | --- | --- | --- | --- | --- | --- | --- | --- | --- | --- | --- | --- | --- | --- | --- | --- | --- | --- | --- | --- | --- | --- | --- | --- | --- | --- | --- | --- | --- | --- | --- | --- | --- | --- | --- | --- | --- | --- | --- | --- | --- | --- | --- | --- | --- | --- | --- | --- | --- | --- | --- | --- | --- | --- | --- | --- | --- | --- | --- | --- | --- | --- | --- | --- | --- | --- | --- | --- | --- | --- | --- | --- | --- | --- | --- | --- | --- | --- | --- | --- | --- | --- | --- | --- | --- | --- | --- | --- | --- | --- | --- | --- | --- | --- | --- | --- | --- | --- | --- | --- | --- | --- | --- | --- | --- | --- | --- | --- | --- | --- | --- | --- | --- | --- | --- | --- | --- | --- | --- | --- | --- | --- | --- | --- | --- | --- | --- | --- | --- | --- | --- | --- | --- | --- | --- | --- | --- | --- | --- | --- | --- | --- | --- | --- | --- | --- | --- | --- | --- | --- | --- | --- | --- | --- | --- | --- | --- | --- | --- | --- | --- | --- | --- | --- | --- | --- | --- | --- | --- | --- | --- | --- | --- | --- | --- | --- | --- | --- | --- | --- | --- | --- | --- | --- | --- | --- | --- | --- | --- | --- | --- | --- | --- | --- | --- | --- | --- | --- | --- | --- | --- | --- | --- | --- | --- | --- | --- | --- | --- | --- | --- | --- | --- | --- | --- | --- | --- | --- | --- | --- | --- | --- | --- | --- | --- | --- | --- | --- | --- | --- | --- | --- | --- | --- | --- | --- | --- | --- | --- | --- | --- | --- | --- | --- | --- | --- | --- | --- | --- | --- | --- | --- | --- | --- | --- | --- | --- | --- | --- | --- | --- | --- | --- | --- | --- | --- | --- | --- | --- | --- | --- | --- | --- | --- | --- | --- | --- | --- | --- | --- | --- | --- | --- | --- | --- | --- | --- | --- | --- | --- | --- | --- | --- | --- | --- | --- | --- | --- | --- | --- | --- | --- | --- | --- | --- | --- | --- | --- | --- | --- | --- | --- | --- | --- | --- | --- | --- | --- | --- | --- | --- | --- | --- | --- | --- | --- | --- | --- | --- | --- | --- | --- | --- | --- | --- | --- | --- | --- | --- | --- | --- | --- | --- | --- | --- | --- | --- | --- | --- | --- | --- | --- | --- | --- | --- | --- | --- | --- | --- | --- | --- | --- | --- | --- | --- | --- | --- | --- | --- | --- | --- | --- | --- | --- | --- | --- | --- | --- | --- | --- | --- | --- | --- | --- | --- | --- | --- | --- | --- | --- | --- | --- | --- | --- | --- | --- | --- | --- | --- | --- | --- | --- | --- | --- | --- | --- | --- | --- | --- | --- | --- | --- | --- | --- | --- | --- | --- | --- | --- | --- | --- | --- | --- | --- | --- | --- | --- | --- | --- | --- | --- | --- | --- | --- | --- | --- | --- | --- | --- | --- | --- | --- | --- | --- | --- | --- | --- | --- | --- | --- | --- | --- | --- | --- | --- | --- | --- | --- | --- | --- | --- | --- | --- | --- | --- | --- | --- | --- | --- | --- | --- | --- | --- | --- | --- | --- | --- | --- | --- | --- | --- | --- | --- | --- | --- | --- | --- | --- | --- | --- | --- | --- | --- | --- | --- | --- | --- | --- | --- | --- | --- | --- | --- | --- | --- | --- | --- | --- | --- | --- | --- | --- | --- | --- | --- | --- | --- | --- | --- | --- | --- | --- | --- | --- | --- | --- | --- | --- | --- | --- | --- | --- | --- | --- | --- | --- | --- | --- | --- | --- | --- | --- | --- | --- | --- | --- | --- | --- | --- | --- | --- | --- | --- | --- | --- | --- | --- | --- | --- | --- | --- | --- | --- | --- | --- | --- | --- | --- | --- | --- | --- | --- | --- | --- | --- | --- | --- | --- | --- | --- | --- | --- | --- | --- | --- | --- | --- | --- | --- | --- | --- | --- | --- | --- | --- | --- | --- | --- | --- | --- | --- | --- | --- | --- | --- | --- | --- | --- | --- | --- | --- | --- | --- | --- | --- | --- | --- | --- | --- | --- | --- | --- | --- | --- | --- | --- | --- | --- | --- | --- | --- | --- | --- | --- | --- | --- | --- | --- | --- | --- | --- | --- | --- | --- | --- | --- | --- | --- | --- | --- | --- | --- | --- | --- | --- | --- | --- | --- | --- | --- | --- | --- | --- | --- | --- | --- | --- | --- | --- | --- | --- | --- | --- | --- | --- | --- | --- | --- | --- | --- | --- | --- | --- | --- | --- | --- | --- | --- | --- | --- | --- | --- | --- | --- | --- | --- | --- | --- | --- | --- | --- | --- | --- | --- | --- | --- | --- | --- | --- | --- | --- | --- | --- | --- | --- | --- | --- | --- | --- | --- | --- | --- | --- | --- | --- | --- | --- | --- | --- | --- | --- | --- | --- | --- | --- | --- | --- | --- | --- | --- | --- | --- | --- | --- | --- | --- | --- | --- | --- | --- | --- | --- | --- | --- | --- | --- | --- | --- | --- | --- | --- | --- | --- | --- | --- | --- | --- | --- | --- | --- | --- | --- | --- | --- | --- | --- | --- | --- | --- | --- | --- | --- | --- | --- | --- | --- | --- | --- | --- | --- | --- | --- | --- | --- | --- | --- | --- | --- | --- | --- | --- | --- | --- | --- | --- | --- | --- | --- | --- | --- | --- | --- | --- | --- | --- | --- | --- | --- | --- | --- | --- | --- | --- | --- | --- | --- | --- | --- | --- | --- | --- | --- | --- | --- | --- | --- | --- | --- | --- | --- | --- | --- | --- | --- | --- | --- | --- | --- | --- | --- | --- | --- | --- | --- | --- | --- | --- | --- | --- | --- | --- | --- | --- | --- | --- | --- | --- | --- | --- | --- | --- | --- | --- | --- | --- | --- | --- | --- | --- | --- | --- | --- | --- | --- | --- | --- | --- | --- | --- | --- | --- | --- | --- | --- | --- | --- | --- | --- | --- | --- | --- | --- | --- | --- | --- | --- | --- | --- | --- | --- | --- | --- | --- | --- | --- | --- | --- | --- | --- | --- | --- | --- | --- | --- | --- | --- | --- | --- | --- | --- | --- | --- | --- | --- | --- | --- | --- | --- | --- | --- | --- | --- | --- | --- | --- | --- | --- | --- | --- | --- | --- | --- | --- | --- | --- | --- | --- | --- | --- | --- | --- | --- | --- | --- | --- | --- | --- | --- | --- | --- | --- | --- | --- | --- | --- | --- | --- | --- | --- | --- | --- | --- | --- | --- | --- | --- | --- | --- | --- | --- | --- | --- | --- | --- | --- | --- | --- | --- | --- | --- | --- | --- | --- | --- | --- | --- | --- | --- | --- | --- | --- | --- | --- | --- | --- | --- | --- | --- | --- | --- | --- | --- | --- | --- | --- | --- | --- | --- | --- | --- | --- | --- | --- | --- | --- | --- | --- | --- | --- | --- | --- | --- | --- | --- | --- | --- | --- | --- | --- | --- | --- | --- | --- | --- | --- | --- | --- | --- | --- | --- | --- | --- | --- | --- | --- | --- | --- | --- | --- | --- | --- | --- | --- | --- | --- | --- | --- | --- | --- | --- | --- | --- | --- | --- | --- | --- | --- | --- | --- | --- | --- | --- | --- | --- | --- | --- | --- | --- | --- | --- | --- | --- | --- | --- | --- | --- | --- | --- | --- | --- | --- | --- | --- | --- | --- | --- | --- | --- | --- | --- | --- | --- | --- | --- | --- | --- | --- | --- | --- | --- | --- | --- | --- | --- | --- | --- | --- | --- | --- | --- | --- | --- | --- | --- | --- | --- | --- | --- | --- | --- | --- | --- | --- | --- | --- | --- | --- | --- | --- | --- | --- | --- | --- | --- | --- | --- | --- | --- | --- | --- | --- | --- | --- | --- | --- | --- | --- | --- | --- | --- | --- | --- | --- | --- | --- | --- | --- | --- | --- | --- | --- | --- | --- | --- | --- | --- | --- | --- | --- | --- | --- | --- | --- | --- | --- | --- | --- | --- | --- | --- | --- | --- | --- | --- | --- | --- | --- | --- | --- | --- | --- | --- | --- | --- | --- | --- | --- | --- | --- | --- | --- | --- | --- | --- | --- | --- | --- | --- | --- | --- | --- | --- | --- | --- | --- | --- | --- | --- | --- | --- | --- | --- | --- | --- | --- | --- | --- | --- | --- | --- | --- | --- | --- | --- | --- | --- | --- | --- | --- | --- | --- | --- | --- | --- | --- | --- | --- | --- | --- | --- | --- | --- | --- | --- | --- | --- | --- | --- | --- | --- | --- | --- | --- | --- | --- | --- | --- | --- | --- | --- | --- | --- | --- | --- | --- | --- | --- | --- | --- | --- | --- | --- | --- | --- | --- | --- | --- | --- | --- | --- | --- | --- | --- | --- | --- | --- | --- | --- | --- | --- | --- | --- | --- | --- | --- | --- | --- | --- | --- | --- | --- | --- | --- | --- | --- | --- | --- | --- | --- | --- | --- | --- | --- | --- | --- | --- | --- | --- | --- | --- | --- | --- | --- | --- | --- | --- | --- | --- | --- | --- | --- | --- | --- | --- | --- | --- | --- | --- | --- | --- | --- | --- | --- | --- | --- | --- | --- | --- | --- | --- | --- | --- | --- | --- | --- | --- | --- | --- | --- | --- | --- | --- | --- | --- | --- | --- | --- | --- | --- | --- | --- | --- | --- | --- | --- | --- | --- | --- | --- | --- | --- | --- | --- | --- | --- | --- | --- | --- | --- | --- | --- | --- | --- | --- | --- | --- | --- | --- | --- | --- | --- | --- | --- | --- | --- | --- | --- | --- | --- | --- | --- | --- | --- | --- | --- | --- | --- | --- | --- | --- | --- | --- | --- | --- | --- | --- | --- | --- | --- | --- | --- | --- | --- | --- | --- | --- | --- | --- | --- | --- | --- | --- | --- | --- | --- | --- | --- | --- | --- | --- | --- | --- | --- | --- | --- | --- | --- | --- | --- | --- | --- | --- | --- | --- | --- | --- | --- | --- | --- | --- | --- | --- | --- | --- | --- | --- | --- | --- | --- | --- | --- | --- | --- | --- | --- | --- | --- | --- | --- | --- | --- | --- | --- | --- | --- | --- | --- | --- | --- | --- | --- | --- | --- | --- | --- | --- | --- | --- | --- | --- | --- | --- | --- | --- | --- | --- | --- | --- | --- | --- | --- | --- | --- | --- | --- | --- | --- | --- | --- | --- | --- | --- | --- | --- | --- | --- | --- | --- | --- | --- | --- | --- | --- | --- | --- | --- | --- | --- | --- | --- | --- | --- | --- | --- | --- | --- | --- | --- | --- | --- | --- | --- | --- | --- | --- | --- | --- | --- | --- | --- | --- | --- | --- | --- | --- | --- | --- | --- | --- | --- | --- | --- | --- | --- | --- | --- | --- | --- | --- | --- | --- | --- | --- | --- | --- | --- | --- | --- | --- | --- | --- | --- | --- | --- | --- | --- | --- | --- | --- | --- | --- | --- | --- | --- | --- | --- | --- | --- | --- | --- | --- | --- | --- | --- | --- | --- | --- | --- | --- | --- | --- | --- | --- | --- | --- | --- | --- | --- | --- | --- | --- | --- | --- | --- | --- | --- | --- | --- | --- | --- | --- | --- | --- | --- | --- | --- | --- | --- | --- | --- | --- | --- | --- | --- | --- | --- | --- | --- | --- | --- | --- | --- | --- | --- | --- | --- | --- | --- | --- | --- | --- | --- | --- | --- | --- | --- | --- | --- | --- | --- | --- | --- | --- | --- | --- | --- | --- | --- | --- | --- | --- | --- | --- | --- | --- | --- | --- | --- | --- | --- | --- | --- | --- | --- | --- | --- | --- | --- | --- | --- | --- | --- | --- | --- | --- | --- | --- | --- | --- | --- | --- | --- | --- | --- | --- | --- | --- | --- | --- | --- | --- | --- | --- | --- | --- | --- | --- | --- | --- | --- | --- | --- | --- | --- | --- | --- | --- | --- | --- | --- | --- | --- | --- | --- | --- | --- | --- | --- | --- | --- | --- | --- | --- | --- | --- | --- | --- | --- | --- | --- | --- | --- | --- | --- | --- | --- | --- | --- | --- | --- | --- | --- | --- | --- | --- | --- | --- | --- | --- | --- | --- | --- | --- | --- | --- | --- | --- | --- | --- | --- | --- | --- | --- | --- | --- | --- | --- | --- | --- | --- | --- | --- | --- | --- | --- | --- | --- | --- | --- | --- | --- | --- | --- | --- | --- | --- | --- | --- | --- | --- | --- | --- | --- | --- | --- | --- | --- | --- | --- | --- | --- | --- | --- | --- | --- | --- | --- | --- | --- | --- | --- | --- | --- | --- | --- | --- | --- | --- | --- | --- | --- | --- | --- | --- | --- | --- | --- | --- | --- | --- | --- | --- | --- | --- | --- | --- | --- | --- | --- | --- | --- | --- | --- | --- | --- | --- | --- | --- | --- | --- | --- | --- | --- | --- | --- | --- | --- | --- | --- | --- | --- | --- | --- | --- | --- | --- | --- | --- | --- | --- | --- | --- | --- | --- | --- | --- | --- | --- | --- | --- | --- | --- | --- | --- | --- | --- | --- | --- | --- | --- | --- | --- | --- | --- | --- | --- | --- | --- | --- | --- | --- | --- | --- | --- | --- | --- | --- | --- | --- | --- | --- | --- | --- | --- | --- | --- | --- | --- | --- | --- | --- | --- | --- | --- | --- | --- | --- | --- | --- | --- | --- | --- | --- | --- | --- | --- | --- | --- | --- | --- | --- | --- | --- | --- | --- | --- | --- | --- | --- | --- | --- | --- | --- | --- | --- | --- | --- | --- | --- | --- | --- | --- | --- | --- | --- | --- | --- | --- | --- | --- | --- | --- | --- | --- | --- | --- | --- | --- | --- | --- | --- | --- | --- | --- | --- | --- | --- | --- | --- | --- | --- | --- | --- | --- | --- | --- | --- | --- | --- | --- | --- | --- | --- | --- | --- | --- | --- | --- | --- | --- | --- | --- | --- | --- | --- | --- | --- | --- | --- | --- | --- | --- | --- | --- | --- | --- | --- | --- | --- | --- | --- | --- | --- | --- | --- | --- | --- | --- | --- | --- | --- | --- | --- | --- | --- | --- | --- | --- | --- | --- | --- | --- | --- | --- | --- | --- | --- | --- | --- | --- | --- | --- | --- | --- | --- | --- | --- | --- | --- | --- | --- | --- | --- | --- | --- | --- | --- | --- | --- | --- | --- | --- | --- | --- | --- | --- | --- | --- | --- | --- | --- | --- | --- | --- | --- | --- | --- | --- | --- | --- | --- | --- | --- | --- | --- | --- | --- | --- | --- | --- | --- | --- | --- | --- | --- | --- | --- | --- | --- | --- | --- | --- | --- | --- | --- | --- | --- | --- | --- | --- | --- | --- | --- | --- | --- | --- | --- | --- | --- | --- | --- | --- | --- | --- | --- | --- | --- | --- | --- | --- | --- | --- | --- | --- | --- | --- | --- | --- | --- | --- | --- | --- | --- | --- | --- | --- | --- | --- | --- | --- | --- | --- | --- | --- | --- | --- | --- | --- | --- | --- | --- | --- | --- | --- | --- | --- | --- | --- | --- | --- | --- | --- | --- | --- | --- | --- | --- | --- | --- | --- | --- | --- | --- | --- | --- | --- | --- | --- | --- | --- | --- | --- | --- | --- | --- | --- | --- | --- | --- | --- | --- | --- | --- | --- | --- | --- | --- | --- | --- | --- | --- | --- | --- | --- | --- | --- | --- | --- | --- | --- | --- | --- | --- | --- | --- | --- | --- | --- | --- | --- | --- | --- | --- | --- | --- | --- | --- | --- | --- | --- | --- | --- | --- | --- | --- | --- | --- | --- | --- | --- | --- | --- | --- | --- | --- | --- | --- | --- | --- | --- | --- | --- | --- | --- | --- | --- | --- | --- | --- | --- | --- | --- | --- | --- | --- | --- | --- | --- | --- | --- | --- | --- | --- | --- | --- | --- | --- | --- | --- | --- | --- | --- | --- | --- | --- | --- | --- | --- | --- | --- | --- | --- | --- | --- | --- | --- | --- | --- | --- | --- | --- | --- | --- | --- | --- | --- | --- | --- | --- | --- | --- | --- | --- | --- | --- | --- | --- | --- | --- | --- | --- | --- | --- | --- | --- | --- | --- | --- | --- | --- | --- | --- | --- | --- | --- | --- | --- | --- | --- | --- | --- | --- | --- | --- | --- | --- | --- | --- | --- | --- | --- | --- | --- | --- | --- | --- | --- | --- | --- | --- | --- | --- | --- | --- | --- | --- | --- | --- | --- | --- | --- | --- | --- | --- | --- | --- | --- | --- | --- | --- | --- | --- | --- | --- | --- | --- | --- | --- | --- | --- | --- | --- | --- | --- | --- | --- | --- | --- | --- | --- | --- | --- | --- | --- | --- | --- | --- | --- | --- | --- | --- | --- | --- | --- | --- | --- | --- | --- | --- | --- | --- | --- | --- | --- | --- | --- | --- | --- | --- | --- | --- | --- | --- | --- | --- | --- | --- | --- | --- | --- | --- | --- | --- | --- | --- | --- | --- | --- | --- | --- | --- | --- | --- | --- | --- | --- | --- | --- | --- | --- | --- | --- | --- | --- | --- | --- | --- | --- | --- | --- | --- | --- | --- | --- | --- | --- | --- | --- | --- |

**Table S2.** Principal component analysis (PCA) for copper-responsive proteins in *Citrus sinensis* leaves

| **Spot**  **No.** | **Protein identity** | **Variables** | |  |  |  |  |  |
| --- | --- | --- | --- | --- | --- | --- | --- | --- |
|  |  | **PC1** | **PC2** | **PC3** | **PC4** | **PC5** | **PC6** | **PC7** |
| **Photosynthesis, carbohydrate and energy metabolism** | |  |  |  |  |  |  |  |
| S19 | Chlorophyll a-b binding protein 8,chloroplastic | 0.552 | -0.623 | -0.373 | -0.012 | 0.208 | -0.204 | -0.133 |
| S41 | Protease Do-like 1, chloroplastic | 0.227 | 0.432 | 0.742 | -0.105 | -0.328 | 0.171 | 0.218 |
| S13 | PsbP domain-containing protein 3, chloroplastic | 0.859 | 0.236 | 0.016 | -0.344 | 0.126 | -0.019 | 0.004 |
| S2 | 29 kDa ribonucleoprotein A, chloroplastic; Ribonucleoprotein At2g37220, chloroplastic | 0.697 | 0.446 | -0.194 | 0.028 | -0.094 | 0.106 | 0.137 |
| S32 | 29 kDa ribonucleoprotein A, chloroplastic; Ribonucleoprotein At2g37220, chloroplastic | 0.955 | 0.071 | 0.060 | -0.090 | 0.023 | 0.160 | 0.136 |
| S17 | Oxygen-evolving enhancer protein 1, chloroplastic | -0.853 | -0.078 | 0.187 | -0.046 | 0.265 | 0.172 | 0.102 |
| S3 | Carbonic anhydrase, chloroplastic | 0.039 | -0.771 | -0.193 | 0.335 | -0.077 | 0.260 | 0.347 |
| S11 | Rubisco subunit binding-protein alpha subunit, chloroplast, putative, expressed; Chaperonin 60 subunit alpha 1, chloroplastic | 0.783 | -0.110 | 0.371 | -0.221 | 0.251 | -0.072 | -0.115 |
| S9 | Ribulose bisphosphate carboxylase/oxygenase activase 1, chloroplastic | 0.655 | 0.197 | 0.132 | -0.403 | -0.449 | -0.316 | -0.164 |
| S14 | Ribulose bisphosphate carboxylase/oxygenase activase 1, chloroplastic | 0.747 | 0.423 | 0.038 | 0.034 | -0.390 | -0.270 | 0.060 |
| S4 | Ribulose bisphosphate carboxylase/oxygenase activase 1, chloroplastic | -0.097 | 0.509 | 0.357 | 0.598 | 0.304 | -0.331 | 0.003 |
| S10 | Ribulose bisphosphate carboxylase/oxygenase activase 1, chloroplastic | -0.045 | -0.859 | 0.347 | -0.233 | 0.152 | -0.191 | 0.013 |
| S21 | Phosphoribulokinase, chloroplastic | 0.859 | -0.423 | 0.137 | -0.019 | -0.139 | 0.069 | -0.029 |
| S33 | Sedoheptulose-1,7-bisphosphatase, chloroplastic | 0.824 | -0.329 | -0.066 | 0.187 | 0.015 | -0.036 | -0.038 |
| S44 | Malate dehydrogenase, mitochondrial | -0.581 | 0.238 | -0.311 | -0.288 | 0.211 | -0.110 | 0.562 |
| S45 | Malate dehydrogenase, mitochondrial | -0.501 | 0.585 | -0.503 | 0.285 | 0.014 | -0.035 | -0.141 |
| S30 | Enolase | 0.832 | 0.179 | 0.408 | 0.175 | 0.025 | 0.142 | -0.203 |
| S36 | Enolase | -0.696 | -0.049 | 0.399 | -0.363 | 0.189 | 0.306 | -0.264 |
| S43 | Dihydrolipoyllysine-residue succinyltransferase component of 2-oxoglutarate dehydrogenase complex 2, mitochondrial | 0.805 | 0.248 | 0.056 | 0.444 | 0.081 | 0.216 | 0.175 |
| **Antioxidation and detoxification** | |  |  |  |  |  |  |  |
| S1 | 2-Cys peroxiredoxin BAS1, chloroplastic | 0.255 | 0.643 | -0.445 | 0.055 | 0.464 | -0.172 | -0.233 |
| S20 | Cysteine synthase, chloroplastic/chromoplastic | -0.414 | 0.474 | 0.733 | -0.070 | -0.172 | -0.132 | 0.049 |
| S39 | Cysteine synthase | 0.873 | -0.176 | 0.010 | -0.080 | -0.136 | 0.365 | 0.106 |
| S34 | L-ascorbate peroxidase 1, cytosolic | 0.821 | -0.102 | 0.139 | -0.035 | -0.069 | -0.212 | -0.231 |
| S24 | Glutathione peroxidase (Fragment) | 0.754 | -0.153 | 0.036 | -0.150 | 0.514 | 0.110 | 0.005 |
| **Chaperones and folding catalysts** | |  |  |  |  |  |  |  |
| S16 | Luminal-binding protein 5 | -0.790 | -0.317 | 0.387 | 0.205 | -0.073 | 0.071 | -0.079 |
| S8 | Peptidyl-prolylcis-transisomerase CYP37, chloroplastic | 0.733 | 0.105 | -0.260 | -0.254 | 0.198 | 0.192 | 0.284 |
| S26 | Chaperonin CPN60-1, mitochondrial, putative, expressed | 0.696 | -0.575 | 0.255 | 0.179 | 0.044 | -0.157 | 0.132 |
| **Signal transduction** | |  |  |  |  |  |  |  |
| S35 | Major allergen Pru ar 1 (Major pollen allergen Bet v 1-D/H; Major pollen allergen Bet v 1-A) | -0.157 | -0.160 | 0.788 | 0.014 | 0.144 | -0.382 | 0.310 |
| S7 | 14-3-3 protein 6 | 0.322 | 0.628 | 0.462 | -0.018 | -0.020 | 0.399 | -0.075 |
| **Cellular transport** | |  |  |  |  |  |  |  |
| S5 | Ferritin-3, chloroplastic | 0.796 | 0.256 | 0.278 | 0.063 | 0.259 | -0.262 | 0.163 |
| **Nucleic acid metabolism** | |  |  |  |  |  |  |  |
| S42 | RuvB-like helicase 1 | 0.750 | -0.113 | -0.057 | 0.547 | -0.275 | 0.036 | -0.031 |
| **Others** | |  |  |  |  |  |  |  |
| S27 |  | 0.347 | 0.702 | 0.364 | 0.176 | 0.406 | 0.222 | -0.068 |
| S28 |  | 0.564 | 0.541 | -0.341 | -0.458 | -0.073 | -0.024 | 0.033 |
| S31 |  | 0.912 | -0.034 | 0.048 | -0.002 | 0.133 | -0.088 | 0.036 |
| S23 | Anthranilate N-methyltransferase | 0.927 | 0.069 | -0.282 | 0.018 | -0.011 | -0.161 | 0.132 |
| S37 | S-adenosyl-L-homocysteine hydrolase (adenosylhomocysteinase) | 0.873 | -0.216 | -0.190 | 0.034 | -0.078 | 0.157 | -0.139 |
| S38 | Dihydroflavonol-4-reductase | 0.442 | -0.713 | 0.326 | -0.060 | 0.189 | 0.093 | -0.169 |
|  |  |  |  |  |  |  |  |  |
| **Eigen value** | | 16.957 | 6.434 | 4.336 | 2.163 | 1.865 | 1.506 | 1.186 |
| **Variation percent (%)** | | 45.831 | 17.389 | 11.719 | 5.846 | 5.039 | 4.071 | 3.205 |

**Table S3.** Principal component analysis (PCA) for copper-responsive proteins in *Citrus grandis* leaves

| Spot  No. | Protein identity | **Variables** | |  |  |  |  |  |  |
| --- | --- | --- | --- | --- | --- | --- | --- | --- | --- |
|  |  | PC1 | PC2 | PC3 | PC4 | PC5 | PC6 | PC7 | PC8 |
| **Photosynthesis, carbohydrate and energy metabolism** | |  |  |  |  |  |  |  |  |
| G1 | 29 kDa ribonucleoprotein A, chloroplastic; Ribonucleoprotein At2g37220, chloroplastic | -0.261 | 0.680 | -0.525 | 0.092 | -0.019 | 0.047 | 0.238 | 0.082 |
| G30 | Photosystem II stability/assembly factor HCF136, chloroplast, putative | -0.375 | 0.676 | -0.197 | -0.346 | 0.297 | -0.135 | -0.195 | 0.172 |
| G31 | Photosystem II stability/assembly factor HCF136, chloroplast, putative | -0.381 | 0.318 | 0.654 | 0.438 | -0.159 | -0.171 | 0.085 | -0.192 |
| G10 | Ferredoxin--NADP reductase, leaf-type isozyme, chloroplastic | 0.120 | -0.801 | 0.167 | -0.057 | 0.229 | 0.339 | 0.063 | 0.206 |
| G42 | Ferredoxin--NADP reductase, leaf-type isozyme, chloroplastic | 0.810 | 0.547 | -0.063 | 0.050 | -0.109 | -0.024 | -0.022 | 0.005 |
| G8 | RuBisCO subunit binding-protein alpha subunit, chloroplast, putative, expressed; Chaperonin 60 subunit alpha 1, chloroplastic | -0.646 | -0.130 | 0.621 | 0.085 | -0.174 | 0.218 | 0.008 | -0.088 |
| G9 | Ribulose bisphosphate carboxylase/oxygenase activase 1, chloroplastic | 0.894 | -0.158 | 0.022 | 0.245 | 0.105 | -0.236 | -0.064 | -0.160 |
| G6 | Sedoheptulose-1,7-bisphosphatase, chloroplastic | 0.561 | 0.593 | -0.102 | -0.104 | 0.368 | -0.082 | 0.091 | -0.349 |
| G38 | Glyceraldehyde-3-phosphate dehydrogenase B, chloroplastic | 0.763 | 0.501 | -0.001 | -0.075 | 0.107 | -0.006 | 0.186 | 0.304 |
| G29 | Triosephosphate isomerase, cytosolic (Fragment) | 0.756 | -0.315 | -0.155 | 0.204 | -0.231 | -0.237 | 0.340 | 0.027 |
| G35 | Triosephosphate isomerase, cytosolic | 0.444 | -0.653 | 0.031 | -0.088 | 0.106 | 0.096 | 0.264 | 0.330 |
| G41 | Triosephosphate isomerase, cytosolic | 0.642 | 0.527 | 0.087 | 0.389 | -0.126 | 0.310 | -0.134 | -0.025 |
| G4 | Probable 6-phosphogluconolactonase 4, chloroplastic | 0.442 | 0.500 | 0.504 | 0.081 | -0.336 | 0.205 | -0.319 | 0.095 |
| G36 | Fructose-1,6-bisphosphatase, cytosolic | 0.710 | 0.470 | 0.280 | -0.310 | 0.250 | 0.057 | -0.066 | 0.145 |
| G17 | Malate dehydrogenase [NADP], chloroplastic | 0.252 | -0.523 | 0.437 | 0.477 | 0.406 | 0.049 | 0.124 | 0.025 |
| G37 | Malate dehydrogenase, cytoplasmic | 0.847 | 0.245 | 0.012 | 0.087 | 0.204 | -0.009 | -0.017 | -0.305 |
| G39 | Malate dehydrogenase, cytoplasmic | 0.294 | 0.858 | -0.008 | 0.115 | 0.036 | 0.270 | -0.110 | 0.038 |
| G18 | ATP synthase subunit beta, mitochondrial | -0.739 | 0.048 | 0.477 | -0.016 | -0.184 | -0.145 | 0.315 | 0.167 |
| G23 | ATP synthase gamma chain, chloroplastic | -0.703 | 0.098 | 0.604 | -0.216 | 0.095 | -0.107 | 0.231 | -0.061 |
| G26 | Bis(5'-adenosyl)-triphosphatase | -0.685 | 0.521 | -0.100 | 0.343 | -0.159 | 0.084 | -0.186 | -0.147 |
| G24 | Glucose-1-phosphate adenylyltransferase small subunit 2, chloroplastic | 0.243 | 0.566 | 0.627 | -0.006 | -0.148 | -0.101 | -0.353 | 0.139 |
| G25 | Glucose-1-phosphate adenylyltransferase small subunit 2, chloroplastic | 0.665 | 0.502 | -0.132 | -0.449 | 0.005 | 0.238 | 0.048 | 0.074 |
| **Antioxidation and detoxification** | |  |  |  |  |  |  |  |  |
| G40 | Glutathione S-transferase | 0.302 | 0.654 | 0.577 | -0.087 | -0.124 | 0.187 | -0.059 | 0.098 |
| G34 | Glutathione S-transferase DHAR1, mitochondrial | 0.409 | -0.471 | -0.128 | 0.453 | 0.383 | 0.181 | -0.424 | 0.064 |
| G21 | Copper/zinc superoxide dismutase (Fragment) | -0.619 | 0.476 | -0.469 | 0.135 | 0.032 | 0.316 | 0.066 | 0.036 |
| G33 | Manganese superoxide dismutase (Fragment) | -0.288 | 0.706 | -0.254 | -0.033 | 0.059 | 0.477 | 0.249 | -0.193 |
| G16 | Quinone oxidoreductase-like protein At1g23740, chloroplastic | 0.835 | -0.166 | 0.307 | 0.311 | 0.200 | 0.120 | -0.112 | 0.002 |
| **Chaperones and folding catalysts** | |  |  |  |  |  |  |  |  |
| G15 | Probable protein disulfide-isomerase A6 | 0.009 | 0.725 | 0.329 | -0.156 | 0.105 | -0.491 | -0.192 | 0.215 |
| G12 | 20 kDa chaperonin, chloroplastic | -0.200 | -0.221 | 0.913 | 0.005 | -0.100 | -0.080 | -0.036 | -0.172 |
| G11 | Heat shock cognate 70 kDa protein 2 | 0.379 | -0.554 | -0.257 | -0.531 | -0.239 | 0.260 | -0.184 | -0.055 |
| G19 | Chaperonin CPN60-1, mitochondrial, putative, expressed | -0.460 | -0.216 | 0.662 | 0.085 | 0.027 | 0.398 | 0.282 | 0.183 |
| **Signal transduction** | |  |  |  |  |  |  |  |  |
| G3 | Calreticulin-1 | 0.401 | -0.654 | 0.092 | -0.480 | -0.309 | 0.087 | -0.001 | -0.216 |
| G27 | Major allergen Pru ar 1 (Major pollen allergen Bet v 1-D/H; Major pollen allergen Bet v 1-A) | 0.361 | 0.548 | -0.231 | 0.233 | -0.538 | -0.199 | 0.001 | 0.166 |
| G5 | 14-3-3 protein 7 (14-3-3-like protein GF14 epsilon) | -0.278 | 0.793 | -0.075 | -0.017 | 0.427 | -0.020 | 0.181 | -0.178 |
| G22 | Annexin D1 | 0.227 | 0.405 | 0.703 | 0.004 | 0.371 | 0.003 | 0.358 | 0.015 |
| **Cellular transport** | |  |  |  |  |  |  |  |  |
| G28 | Ferritin-2, chloroplastic | -0.699 | -0.025 | -0.089 | -0.125 | 0.376 | -0.372 | -0.258 | 0.121 |
| **Cell wall and cytoskeleton** | |  |  |  |  |  |  |  |  |
| G7 | Tubulin beta-6 chain | 0.810 | 0.351 | 0.031 | -0.174 | -0.070 | -0.021 | 0.380 | 0.015 |
| G2 | Endochitinase 1 | -0.287 | -0.494 | -0.638 | 0.452 | 0.100 | 0.024 | 0.018 | 0.183 |
| **Stress response** | |  |  |  |  |  |  |  |  |
| G14 | Abscisic stress-ripening protein 1-like | -0.636 | 0.624 | -0.393 | -0.107 | -0.045 | 0.050 | 0.119 | -0.047 |
| **Others** | |  |  |  |  |  |  |  |  |
| G20 |  | -0.563 | 0.664 | -0.094 | 0.335 | -0.025 | 0.135 | -0.056 | 0.184 |
| G32 | S-adenosyl-L-homocysteine hydrolase (adenosylhomocysteinase) | 0.651 | 0.087 | -0.317 | 0.379 | -0.232 | -0.277 | 0.395 | 0.041 |
|  |  |  |  |  |  |  |  |  |  |
| **Eigen value** | | 12.503 | 10.866 | 6.158 | 2.774 | 2.123 | 1.832 | 1.765 | 1.033 |
| **Variation percent (%)** | | 30.496 | 26.503 | 15.019 | 6.765 | 5.178 | 4.467 | 4.305 | 2.520 |

**Table S4.** Specific primer pairs used for qRT-PCR analysis

| Spot No. | Genes | Accession No. | Forward primers (5´→3´) | Reverse primers (5´→3´) |
| --- | --- | --- | --- | --- |
| G3 | Calreticulin-1 | Cs3g15060.3 | CTCTAACTCTCCTCATTATCTTCC | GTCTTGTCCTTGTTGCTGAA |
| G9 | Ribulose bisphosphate carboxylase/oxygenase activase 1, chloroplastic | Cs7g31800.3 | TCAAGATTACTGCTGAAGTC | TGGCTGATGTAGTCATAAGA |
| G10 | Ferredoxin--NADP reductase, leaf-type isozyme, chloroplastic | Cs1g25510.4 | CAAGTCCACTTCGCTTCCAA | ATCTGCCAATGTAAGGTGTCTT |
| G11 | Heat shock cognate 70 kDa protein 2 | Cs7g29010.1 | AGGTTGATTGGTAGGAGATT | TGTGAGTCATTGAAGTAAGC |
| G14 | Abscisic stress-ripening protein 1-like | Cs3g21500.1 | ACTGATTACTCTGAGACTAC | TATCTCCTCCTCTATCTTGT |
| G26 | Bis(5'-adenosyl)-triphosphatase | Cs9g13060.1 | AGGCAAGTGTTCTATTCAAC | GGTCTCATCAGCAGTAAGAT |
| G29 | Triosephosphate isomerase, cytosolic (Fragment) | Cs5g16495.1 | TTGCTCAGGTTCAGGAAGTC | TCTGGTATTTGTCTAGGATGGG |
| G33 | Manganese superoxide dismutase (Fragment) | Cs7g29850.1 | GTTCTCTGGAAGCATTGATAC | GACATTCTTGTACTGTAGGTAG |
| G34 | Glutathione S-transferase DHAR1, mitochondrial | Cs7g28340.4 | CCTTCTCTTACCAATCCTCCT | CTACAGCAGTGACCTTCTCT |
| G35 | Triosephosphate isomerase | Cs7g32500.1 | AATGGAACACCTGAAGAAGT | CAGAATGACCAAGAATGACC |
| S2 | 29 kDa ribonucleoprotein A, chloroplastic | Cs6g11900.1 | TATCTTCCTCCTCTTCTCAC | ATTACCTCAACCATCTCAAC |
| S5 | Ferritin-3,chloroplastic | Cs6g09150.2 | AGTCTAATGCTTCTCTTGTG | ATTGATCTGCTCGTTGATAG |
| S9 | Ribulose bisphosphate carboxylase/oxygenase activase 1, chloroplastic | Cs7g31800.3 | TCAAGATTACTGCTGAAGTC | TGGCTGATGTAGTCATAAGA |
| S16 | Luminal-binding protein 5 | Cs5g01840.2 | GAATCAGGCTGCTGTCAATC | GGCTGTCTCCTTCATCTTAGT |
| S17 | Oxygen-evolving enhancer protein 1, chloroplastic | Cs1g23450.1 | TAGAACCAACTTCCTTCACT | CAGCATAGTCAATTCCATCC |
| S23 | Anthranilate N-methyltransferase | Cs5g24940.1 | CTACTCTCTTGCTCCTGTTT | GCTTGACATTCCATTGTTGA |
| S24 | Glutathione peroxidase (Fragment) | Cs5g03830.1 | CCAGGAGACAATGAACAGATTC | GGCATAACGCTCAACAACAT |
| S30 | Enolase | Cs6g15540.1 | CAAGCATATTGCCGAACTCTC | TTAATCACAGCCTTCAGATGGT |
| S32 | Chloroplast 29 kDa ribonucleoprotein (Fragment) | Cs7g01430.1 | ACAGCAAGCACCGAAGAATC | CGACTCCTTCCAGTGTACTTATC |
| S33 | Sedoheptulose-1,7-bisphosphatase, chloroplastic | Cs7g31640.4 | CACACGAGTTCCTTCTTCTTGA | ATTGACATCTGGCACCATTCC |
| S37 | S-adenosyl-L-homocysteine hydrolase | Orange1.1t01892.1 | GTTGATCCACGAGGGAGTCA | TCTTCGGAAACACCAACTAATC |
| S43 | Dihydrolipoyllysine-residue succinyltransferase component of 2-oxoglutarate dehydrogenase complex 2, mitochondrial | Cs2g21190.3 | GAAGCAACCTGGAGATAGAGT | AGCAACACCTTCACCTGATT |
|  | *Actin* | Cs1g05000.1 | AGAACTATGAACTGCCTGATGGC | GCTTGGAGCAAGTGCTGTGATT |
|  | *U4/U6 small nuclear ribonucleoprotein PRP31 (PRPF31)* | Cs7g08440.1 | ACTCATGGGAACGGCTGGTGGTC | TCGGCAGGCACGCATCCTTAGAG |
